# Red-Light-Driven Ru(II) Photocatalysts Enable Dual Oxidation of NADH and Glutathione in Living Cells

**DOI:** 10.1021/acs.inorgchem.6c03885

**Published:** 2026-10-27

**Authors:** Mikel Gabino-García, María Gil-Moles, M. Concepción Gimeno

**Affiliations:** Departamento de Química Inorgánica, Instituto de Síntesis Química y Catálisis Homogénea (ISQCH), CSIC-Universidad de Zaragoza, C. Pedro Cerbuna 12, 50009 Zaragoza, Spain

## Abstract

Intracellular photocatalysis has emerged as a promising strategy to disrupt cancer redox homeostasis, yet most photoredox systems remain limited by oxygen dependence and deactivation by intracellular thiols, particularly glutathione (GSH). Here we report PEGylated Ru­(II) terpyridine complexes that function as visible-light photocatalysts, and catalytically oxidize both NADH and GSH through complementary Type I and Type II pathways. The complexes exhibit broad visible-light absorption, aqueous stability, and activity in biologically relevant media. Stern–Volmer quenching experiments and electrochemical studies are consistent with excited-state electron transfer from NADH and GSH, while ROS studies reveal singlet oxygen, superoxide, and hydrogen peroxide during catalysis. Unexpectedly, glutathione enhances rather than suppresses photocatalytic activity, supporting its participation in catalyst turnover. NADH oxidation is maintained in oxygen-limited solution. In cancer cells, red-light irradiation disrupts the intracellular NAD^+^/NADH balance, depletes glutathione, and amplifies oxidative stress, resulting in selective photocytotoxicity. Comparative studies further reveal that intracellular performance depends not only on catalytic efficiency in solution but also on catalyst photostability and accessibility to cellular redox pools. Together, these findings establish terpyridine-based Ru­(II) photocatalysts as an effective strategy for disrupting intracellular redox balance and provide new insight into glutathione-assisted photocatalysis in biological systems.

## Graphical abstract

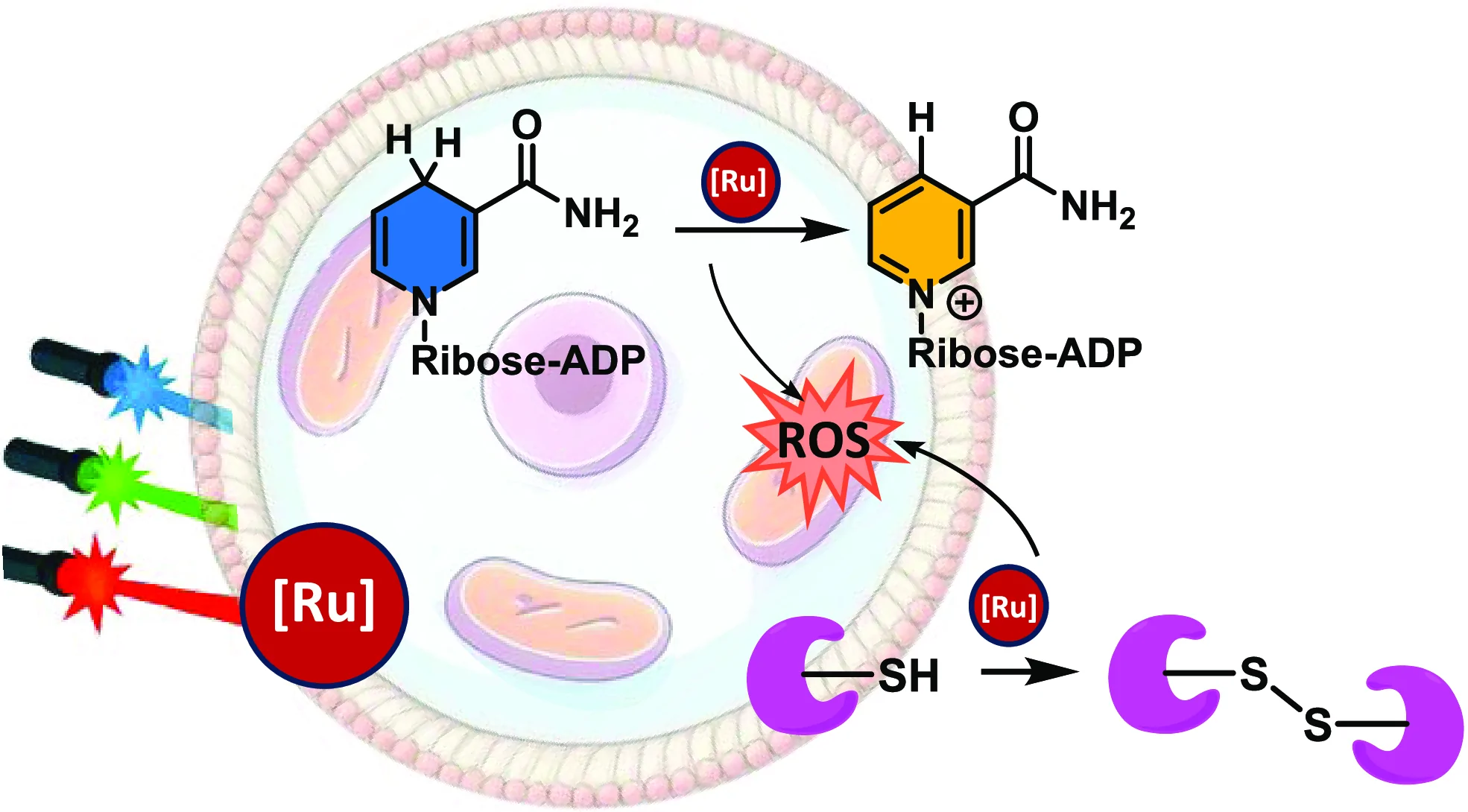

## Introduction

Cancer cells depend on tightly regulated redox networks to sustain metabolic plasticity and resist oxidative stress. Central to this balance are the NAD^+^/NADH and GSH/GSSG couples, which together support energy production, reductive biosynthesis, and antioxidant defense.^1–3^ Perturbation of these redox buffers represents an attractive strategy for anticancer therapy because even modest shifts in intracellular redox balance can compromise metabolic resilience and sensitize tumor cells to oxidative damage.

Photodynamic therapy (PDT) generates reactive oxygen species (ROS) upon light activation. However, its efficacy is limited by oxygen dependence, stoichiometric ROS production, and rapid quenching by intracellular thiols such as glutathione.^4^ As a result, strategies capable of amplifying oxidative stress through catalytic rather than stoichiometric mechanisms are of considerable interest.

Photocatalytic cancer therapy (PCT) has recently emerged as an alternative paradigm in which light-activated catalysts operate in redox cycles, enabling sustained perturbation of cellular metabolism under irradiation. In principle, catalytic oxidation of reduced cofactors such as NADH could disrupt metabolic electron flow, while simultaneous depletion of glutathione would compromise the primary intracellular antioxidant buffer. Developing catalysts capable of simultaneously oxidizing both NADH and GSH in the intracellular environment therefore remains a major challenge.^5–13^

Transition-metal complexes are attractive platforms for intracellular photocatalysis because of their long-lived excited states, tunable redox properties, and compatibility with visible-light activation. Among them, Ir­(III) complexes have dominated the field,^14,15^ and, far fewer Ru­(II) photocatalysts,^16–19^ have been reported to promote light-driven NADH oxidation, primarily in solution with limited examples in living cells.^14,18^ Although catalytic oxidation of NADH can disrupt metabolic electron flow, its biological impact may be limited because the intracellular glutathione (GSH/GSSG) system efficiently buffers oxidative stress and restores redox homeostasis. Consequently, simultaneous catalytic oxidation of both NADH and GSH represents a more effective strategy to induce sustained redox imbalance by coupling metabolic disruption with depletion of the major intracellular antioxidant.

Achieving such dual redox catalysis, however, remains highly challenging. Glutathione not only scavenges reactive oxygen species but also coordinates metal centers and quenches excited states, frequently leading to catalyst deactivation. Although glutathione has traditionally been regarded as a major obstacle to Ru photoredox catalysis because it quenches excited states and scavenges reactive oxygen species, only one recent mechanistic study has examined GSH, together with NADH oxidation, with blue light as a productive electron donor for Ru photocatalysts in solution.^20^ Whether this process can be exploited to sustain intracellular photocatalysis while simultaneously perturbing glutathione homeostasis has remained unexplored.

Ru­(II) polypyridyl complexes are well-established light-responsive agents for photodynamic therapy^21–24^ and photoactivated chemotherapy (PACT),^25,26^ owing to their rich excited-state landscapes, photostability, and compatibility with visible-light activation. Their therapeutic activity, however, typically relies on ROS generation or photoinduced ligand release, and thus remains constrained by blue or green excitation, limited tissue penetration, and oxygen dependence, which severely compromises efficacy in hypoxic tumors.^27^ Although π-extended diimine ligands have enabled red-shifted absorption,^28–30^ terpyridine-based Ru­(II) complexes remain comparatively underexplored, despite offering structural rigidity, electronic tunability, and favorable photochemical properties.^31–33^

Successful intracellular photocatalysis depends not only on intrinsic catalytic efficiency but also on catalyst stability, aqueous compatibility, and access to intracellular redox pools. To address these requirements, we incorporated PEG substituents into the terpyridine scaffold to enhance water solubility, improve compatibility with biological media, and minimize aggregation under physiological conditions, while allowing us to evaluate how these properties influence intracellular photocatalytic performance. This design provides a platform to examine whether photocatalytic activity toward NADH and GSH can be translated from solution to the intracellular environment.

Here we report PEGylated Ru­(II) terpyridine complexes that catalytically oxidize both NADH and GSH under blue, green, and red-light irradiation. The distinctive feature of this system is not NADH photooxidation or ROS generation per se, but the combination of dual NADH/GSH redox photocatalysis with the productive participation of GSH, which enhances rather than suppresses catalytic activity. This behavior allows efficient photocatalysis to be maintained under oxygen-limited solution conditions and extends to 600 nm irradiation. Importantly, the photocatalytic behavior translates into living cancer cells, where red-light irradiation perturbs both the NAD^+^/NADH and GSH/GSSG redox pools and enhances oxidative stress. Comparison of two closely related complexes further reveals that intracellular performance depends not only on catalytic efficiency in solution but also on catalyst photostability and accessibility to cellular redox environments. Together, these findings distinguish this system from previously reported metal-based photocatalysts focused primarily on NADH oxidation or ROS generation and establish GSH-assisted dual redox photocatalysis as a strategy for perturbing intracellular redox homeostasis.

## Results and Discussion

### Synthesis

Ligand **L** was synthesized by coupling terpyridine carboxylic acid with a PEGylated amino–alkyne derivative. Subsequent reaction with RuCl_3_ afforded the bis­(terpyridine)-Ru­(II) **1** and terpyridine–bipyridine Ru­(II) **2** complexes (Scheme 1). These complexes show excellent solubility in both organic solvents (CH_2_Cl_2_, CHCl_3_, CH_3_OH, CH_3_CN) and biological media. Full synthetic procedures and characterization (NMR, HR-MS, FT-IR and analytical data) are provided in the ESI (Figures S1–S22). The spectroscopic and analytical data are fully consistent with the proposed structures. The FT-IR spectra of complexes **1** and **2** display the characteristic amide C=O stretching bands at 1650 cm^–1^ in both cases, further supporting the presence of the carbonyl-containing functionality. It is important to note that under the reaction conditions, complex **2** partially hydrolyzed, cleaving the alkyne and yielding a hydroxyl group.

**Scheme 1:**
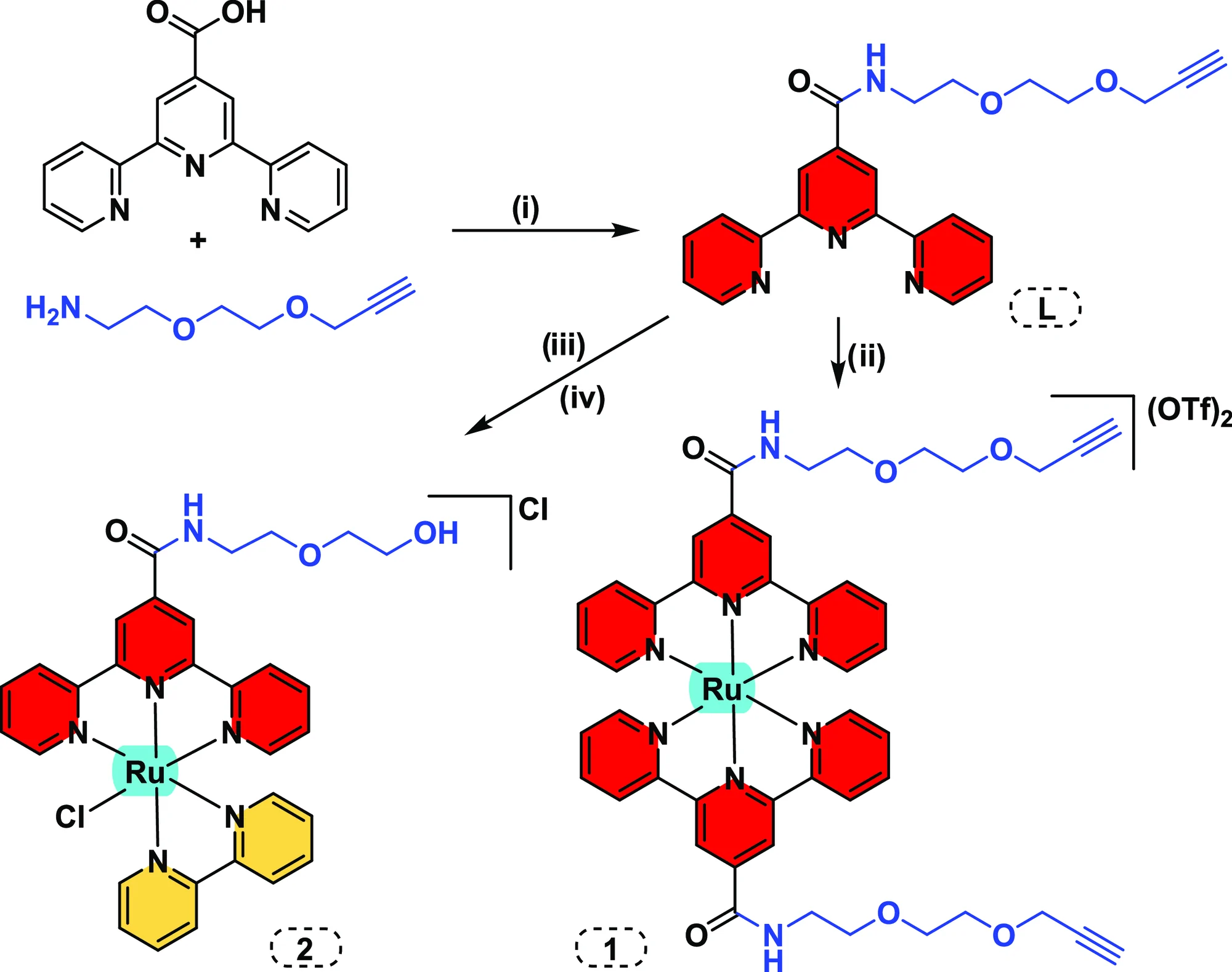
Synthesis of the Ligand **L** and Complexes **1** and **2** ^a^

a (i) DIPEA, HATU, DMF; (ii) RuCl_3_·*x*H_2_O, *N*-ethylmorpholine, 80 °C; (iii) RuCl_3_·*x*H_2_O, EtOH, 80 °C; (iv) 2,2′-bipyridine, LiCl, Et_3_N, EtOH/H_2_O, 88 °C.

### Photophysical Properties, Stability, Photoaquation, and Singlet Oxygen Production

Before investigating the photocatalytic activity of the complexes, we first evaluated whether they possess the photophysical and chemical properties required to operate under biologically relevant conditions. The UV–Vis spectra of complexes **1** and **2** recorded in DMEM display the characteristic absorption profile of Ru­(II)–Terpy systems. In the high-energy region (230–320 nm), both compounds exhibit well-defied, structured bands with three maxima (**1**: 233, 272, 314 nm; **2**: 233, 280, 319 nm), which are assigned to intraligand charge-transfer (ILCT) and ligand-to-ligand charge-transfer (LLCT). These features closely resemble those reported for related complexes,^17,34,35^ indicating that the electronic structures of the complexes are largely preserved in the biological medium. At lower energies, both complexes show a broad band spanning approximately 390 to 620 nm, with maxima at 487 nm (**1**) and 495 nm (**2**), characteristic of metal-to-ligand charge-transfer transition (MLCT) (Figure S23). These broad absorption profiles cover a large portion of the visible spectrum and make both complexes suitable candidates for visible-light photocatalysis.

Both complexes remain largely stable in the dark over 48 h, with the high-energy region remaining essentially unchanged and only minor intensity variations observed in the MLCT region. While complex **1** retains its MLCT profile over time, complex **2** displays a slight blue shift of this band, attributable to slow hydrolysis. Photostability studies under 470 nm irradiation reveal the same trend: complex **1** remains photostable, whereas **2** undergoes accelerated hydrolysis (see SI Figures S24–S25). To further elucidate this behavior, ^1^H NMR studies in D_2_O were conducted in the dark and under 470 nm light (see Figure 1A and SI Figures S26–28). These experiments corroborate the initial findings: complex **1** showed no detectable spectral changes, whereas complex **2** exhibits progressive shifts consistent with Cl^–^ substitution by D_2_O. Notably, hydrolysis of **2** is markedly enhanced under irradiation (dark *K*_tot_ = 4.78 × 10^–5^ s^–1^; 470 nm *K*_tot_ = 1.89 × 10^–3^ s^–1^) (Figure 1B, SI Figures S29–S31, Table S1), confirming its pronounced photolability. To investigate chloride hydrolysis, HR-MS analysis was performed before and after irradiation at 470 nm. Before irradiation, the species [M–Cl]^+^ was detected (calcd *m*/*z* 657.0950; found 657.0953; SI Figure S32), whereas after irradiation a new species at *m*/*z* 639.1293 was observed (calcd 639.1289), assigned to [M+H_2_O–HCl]^+^. The appearance of this species following irradiation provides additional evidence for photoinduced chloride hydrolysis, consistent with the changes observed by UV–Vis and NMR spectroscopy.

**Figure 1:**
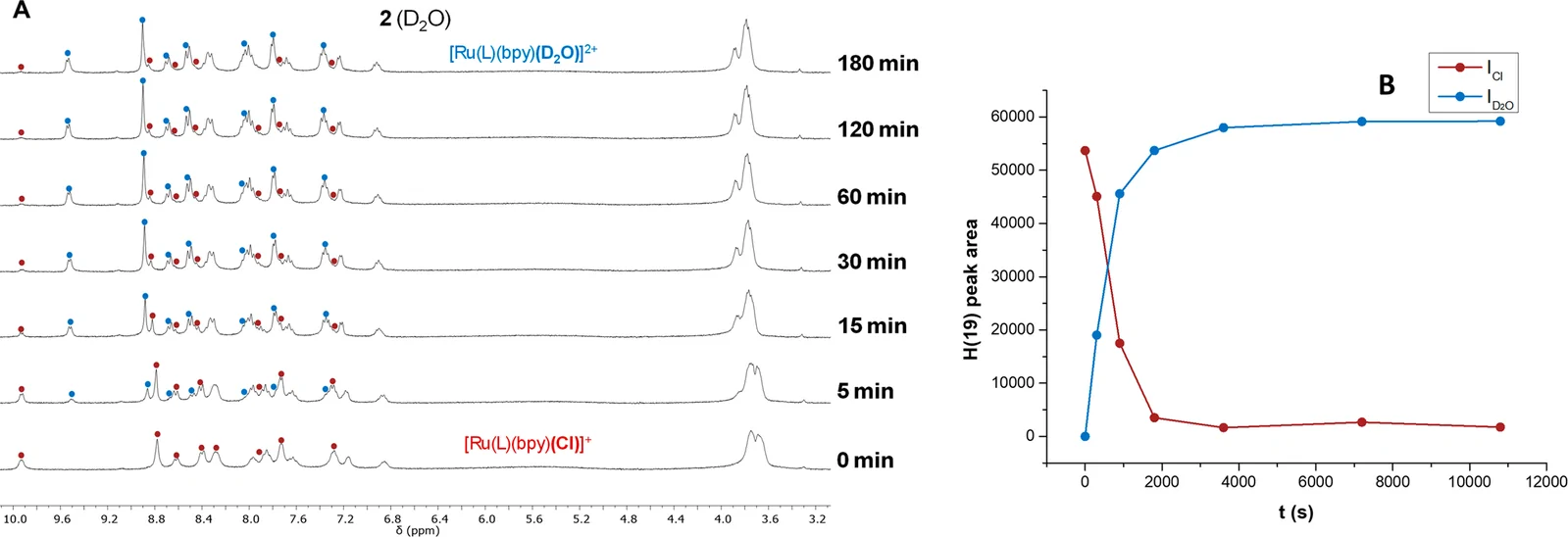
(A) Time evolution of ^1^H NMR of complex **2** during the hydrolysis of the Cl^–^ ligand upon dissolution in D_2_O and irradiation at 470 nm up to 3 h. Peaks corresponding to species [**2**-Cl]^+^ and [**2**-D_2_O]^2+^ are marked in red and blue, respectively. (B) Plot of peak area of H (19) vs time for species [**2**-Cl]^+^ (red) and [**2**-D_2_O]^2+^ (blue).

Having established the visible-light absorption and stability of the complexes, we next investigated their excited-state properties. Complex **1** was essentially nonemissive, whereas both the free ligand **L** and complex **2** exhibited measurable luminescence (Figure 2). In DMSO, **L** displays excitation maxima at 350 and 450 nm, giving rise to an emission band centered at 480 nm. Upon coordination to Ru­(II), the excitation profile shifts to longer wavelengths, with complex **2** exhibiting excitation maxima at 500 nm in DMSO and 480 nm in PBS, together with near-infrared emission centered at 770 and 735 nm, respectively (Figure 2; Figures S33–S34; Table S2). These features are consistent with excitation involving ligand-centered transitions and Ru→terpyridine MLCT states. The free ligand exhibits an emission lifetime of 5.79 ns in DMSO, whereas coordination to Ru­(II) significantly extends the excited-state lifetime to 48.82 ns in DMSO and 17.89 ns in PBS. Although shorter than those typically reported for classical Ru­(II) polypyridyl phosphors, these values are in good agreement with related Ru­(II) terpyridine complexes and are attributed to emission from a ^3^MLCT excited state that is efficiently deactivated through thermally accessible metal-centered (^3^MC) states.^36^

**Figure 2:**
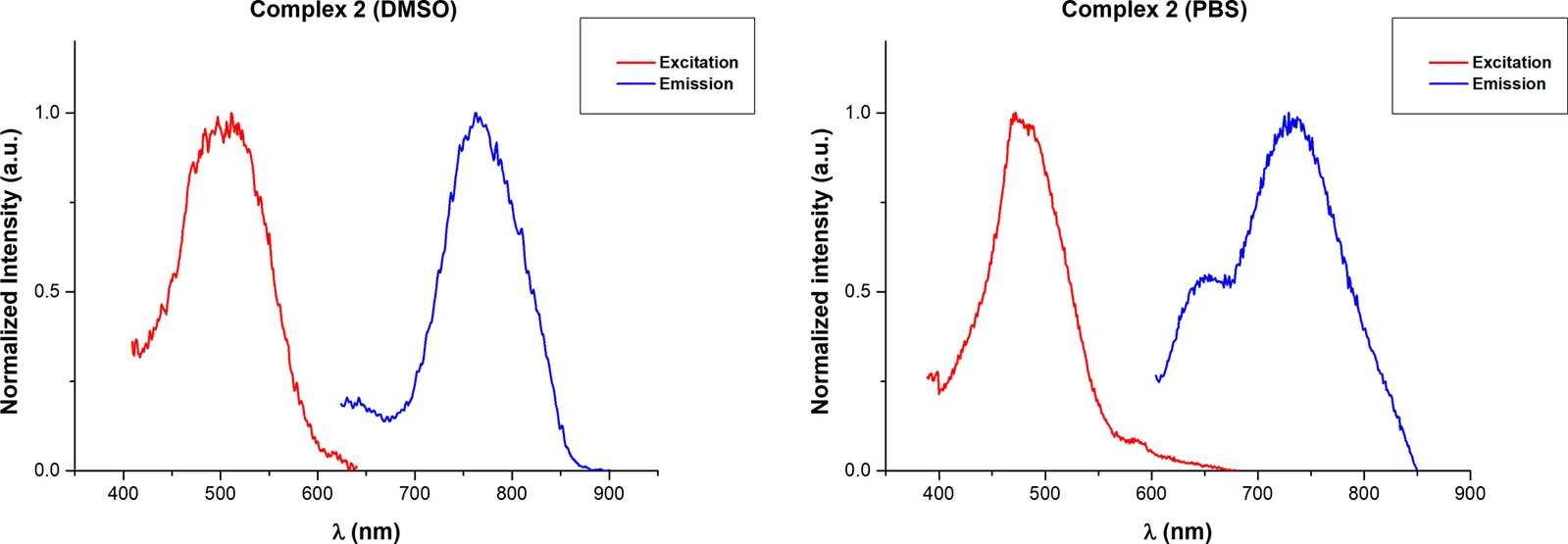
Normalized excitation and emission spectra of complex **2** in DMSO and PBS (30 μM) at 298 K.

The ability of these complexes to generate singlet oxygen was also evaluated using 9,10-diphenylanthracene (DPA) as a chemical trapping probe for ^1^O_2_, with [Ru­(bpy)_3_]^2+^ as the reference photosensitizer for the determination of the singlet-oxygen quantum yield (Φ_Δ_).^37–40^ As shown in Figure 3 and Figure S35, complex **1** generated ^1^O_2_ with a quantum yield of 5.7%, whereas complex **2** exhibited a significantly lower quantum yield of 0.6%. These results demonstrate that both complexes are capable of generating singlet oxygen, although with relatively low quantum yields.

**Figure 3:**
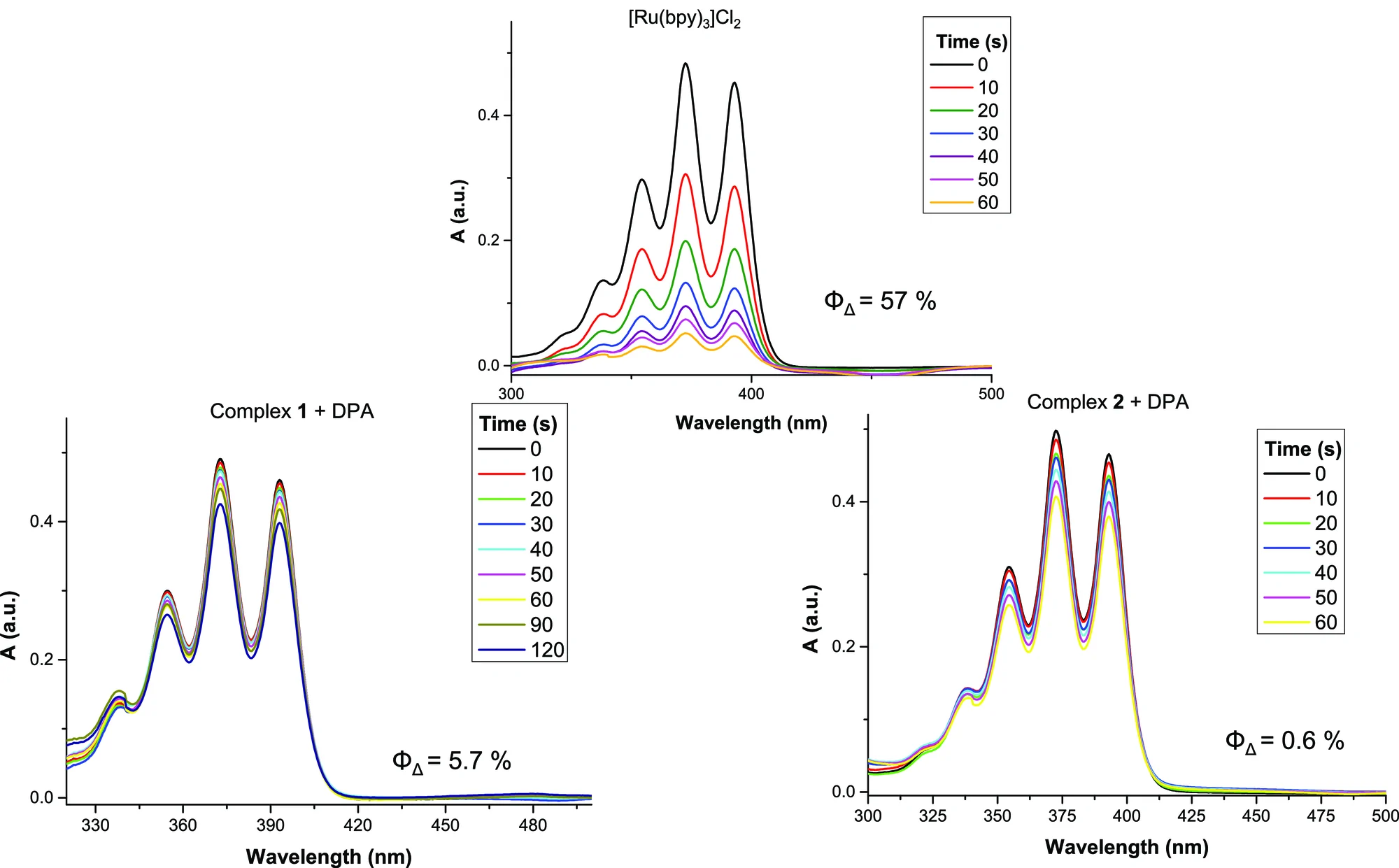
Singlet oxygen detection by indirect method using DPA.

### Excited-State Interaction with NADH and Glutathione

To determine whether photocatalytic oxidation is initiated by direct interaction of biological reductants with the excited Ru­(II) center, Stern–Volmer emission quenching experiments were performed using complex **2** in PBS. Progressive addition of either NADH or glutathione (GSH) resulted in efficient quenching of the Ru emission, yielding linear Stern–Volmer plots (*I*_0_/*I* vs [Q], where [Q] is the concentration of NADH or GSH, see Figure 4 and Figures S36–S38). The corresponding apparent bimolecular quenching constants were 3.34 × 10^8^ M^–1^ s^–1^ for NADH and 2.86 × 10^8^ M^–1^ s^–1^ for GSH, demonstrating that both substrates efficiently interact with the excited state of the catalyst. These observations are consistent with quenching of the excited state and support the interaction of both NADH and GSH with the photoexcited complex, although steady-state Stern–Volmer analysis alone does not establish the nature of the quenching mechanism. The similar quenching efficiencies further suggest that glutathione, traditionally regarded as a major deactivating agent for photocatalysts, may instead participate productively in catalyst turnover under the reaction conditions.^9^

**Figure 4:**
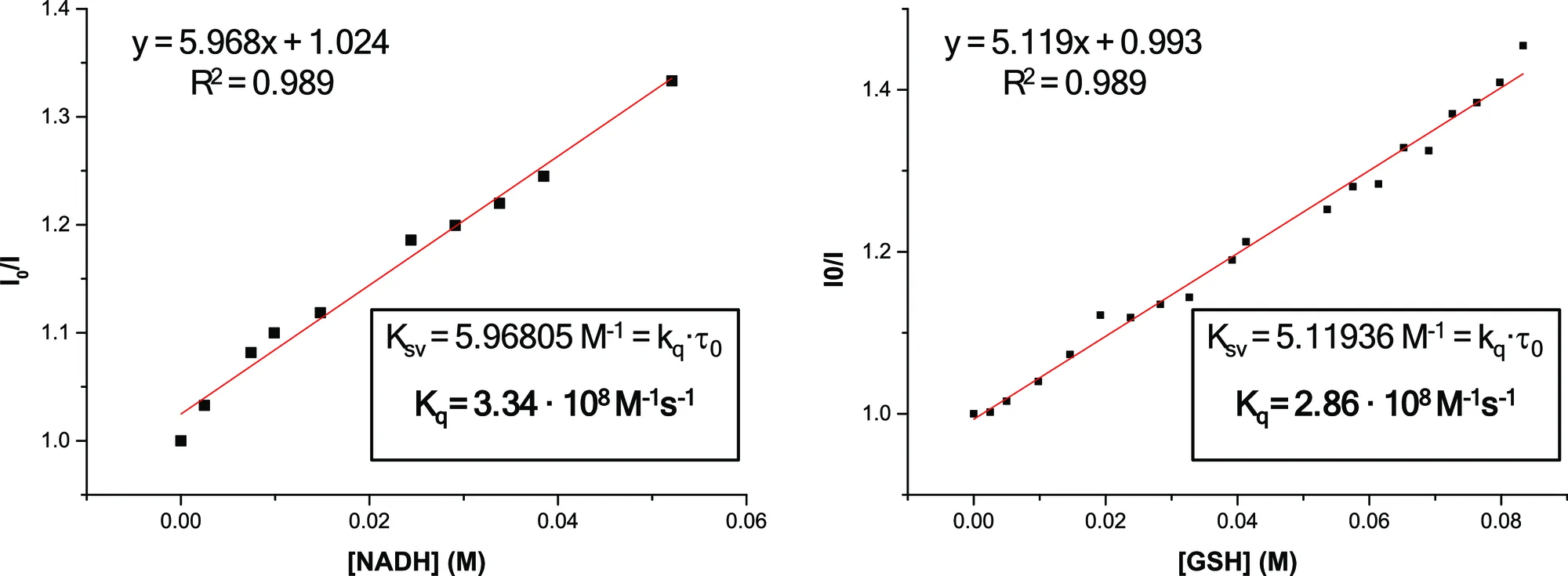
Stern–Volmer linear plot for NADH (left) and GSH (right). The corresponding apparent *k*_q_ value, estimated from *K*_SV_/τ_0_ assuming a dynamic-quenching model, has also been included.

### Electrochemical Properties

The suitability of the ruthenium complexes to participate in the proposed photoredox processes was assessed by cyclic voltammetry (SI Figure S39). Both complexes display well-defined [RuLL′]^+3^/[RuLL′]^+2^ couples at *E*_1/2_ = +1.093 V for **1** and +0.609 V for **2** (L = Terpy, L′ = Terpy or bpy). The reversibility of the redox processes indicates stable interconversion between oxidation states. Importantly, both complexes also display the one-electron reduction process assigned to the [RuLL′]^+2^/[RuLL′]^+^ couple, demonstrating that the reduced state involved in the proposed photocatalytic cycle is electrochemically accessible.^41,42^ For complex **1**, this process is irreversible, with *E*_red_ = −1.381 V, whereas complex **2** shows a reversible process with and *E*_red_ = −1.809 V and *E*_ox_ = −1.739 (*E*_1/2_ = −1.774 V). A second reduction process, tentatively assigned as ligand-centered, is observed at *E*_red_ = −1.870 V for **1** and *E*_red_ = −2.119 V and *E*_ox_ = −1.999 V (*E*_1/2_ = 2.059 V) for **2**.

To evaluate whether formation of the reduced [RuLL′]^+^ state is energetically feasible following photoexcitation, the excited-state reduction potential was estimated for complex **2**, the emissive complex. From an excited-state energy of *E*_00_ = 2.03 eV and the ground-state reduction potential, an excited-state reduction potential E*_red_([RuLL′]^+2*^/[RuLL′]^+^) = +0.221 V (SI eq S7) was obtained. Comparison with the oxidation potentials of NADH (−0.32 V) and GSH (−0.40 V) indicates that electron transfer from either substrate to photoexcited complex **2** is thermodynamically favorable. The corresponding free-energy, calculated according to Δ*G*_PET_ = −*F*[E*_red_(acceptor) – *E*_ox_(donor)], are approximately −52 and −60 kJ mol^–1^ for NADH and GSH, respectively (SI eq S8). Thus, the electrochemical data provide experimental support for the energetic feasibility of the proposed reductive electron-transfer pathway and for the accessibility of the [Ru]^+^ state, although they do not constitute direct spectroscopic observation of this intermediate under photocatalytic conditions.^41^

In addition, the HOMO and LUMO energies were calculated as −5.982 and −3.436 eV for **1**, and −5.512 and −3.007 eV for **2**, corresponding to HOMO–LUMO gaps of 2.546 and 2.505 eV, respectively (SI eqs S9–S10). These values are consistent with the electronic characteristics of the complexes and complement the electrochemical and photophysical data.^43^

### NADH Photo-Oxidation

The photocatalytic activity of complexes **1** and **2** toward NADH oxidation was evaluated under physiologically relevant conditions in PBS (pH 7.4). Because efficient photocatalysts for biological applications should ideally operate under long-wavelength irradiation, catalytic activity was investigated under blue (470 nm), green (525 nm), and red (600 nm) light. Whereas blue light provides efficient excitation of the Ru­(II) chromophore, green and red light offer improved tissue penetration and lower phototoxicity, making them particularly attractive for biomedical applications. NADH oxidation was monitored by UV–Vis spectroscopy through the decrease of the characteristic NADH absorption band at 340 nm accompanied by the growth of the NAD^+^ band at 260 nm (Figure 5). No spectral changes were observed in the dark, confirming that the reaction is strictly photoactivated (Figure S40). Upon irradiation, both complexes efficiently catalyzed the oxidation of NADH, with significantly higher activity under 470 nm than under 525 nm irradiation. In all cases, complex **2** consistently outperformed complex **1**, providing higher TON, TOF, and conversion values (Table 1 and Figures S41–S46). Although catalytic efficiency decreased with increasing wavelength, complex **2** remained active under red-light irradiation, producing measurable NADH oxidation at 600 nm (Table 1 and Figure S47). While the catalytic activity under these conditions is modest, the ability to promote photocatalysis under red light is noteworthy given its superior tissue penetration and lower intrinsic phototoxicity.

**Figure 5:**
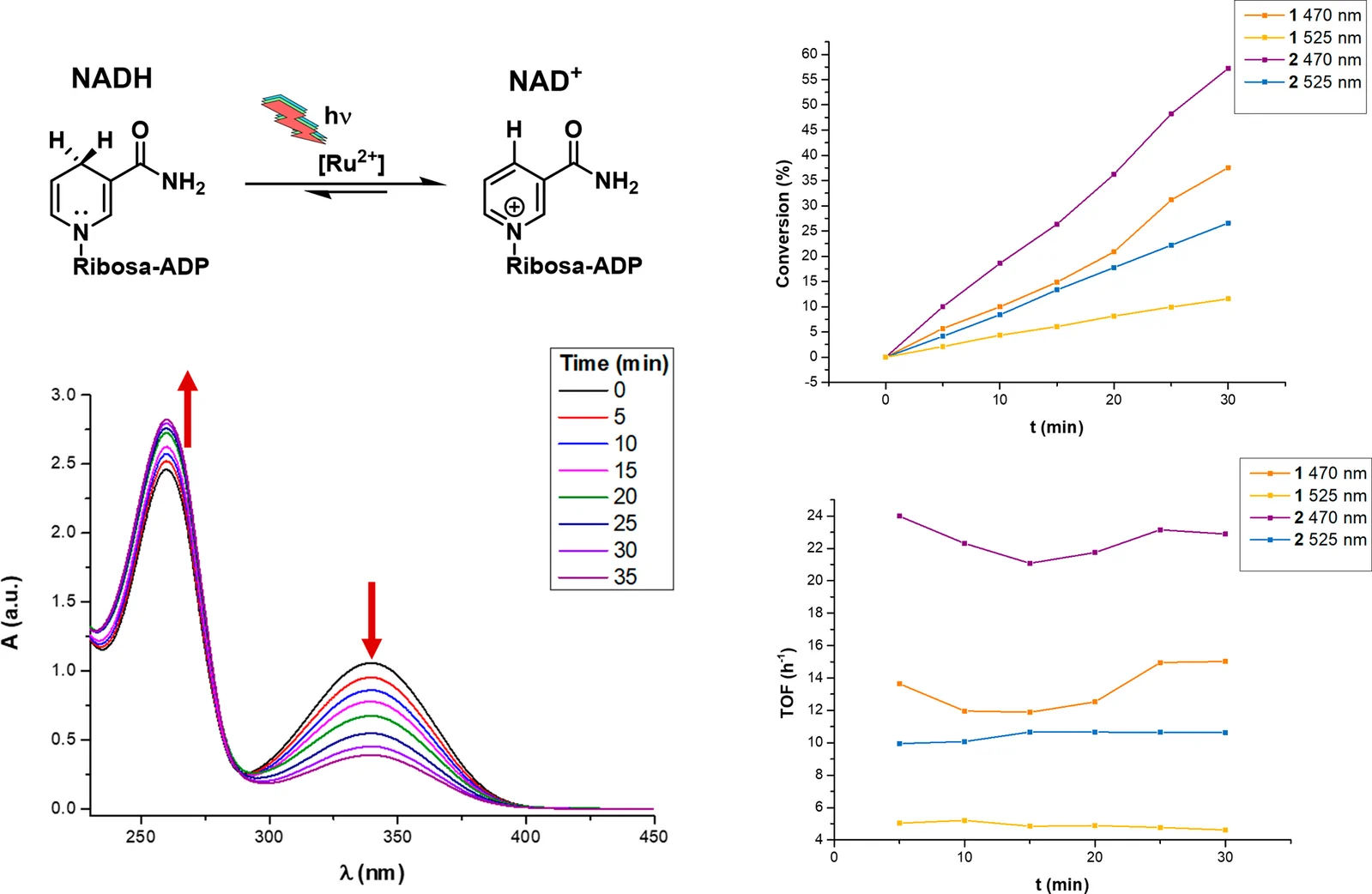
Time-resolved UV–vis spectrum of NADH in the presence of catalyst **2** (10 mM; 5 mol %) under 470 nm irradiation. Compared plots of conversion and TOF for complexes **1** and **2** (10 mM; 5 mol %) under irradiation at 470 and 525 nm.

**Table 1:**
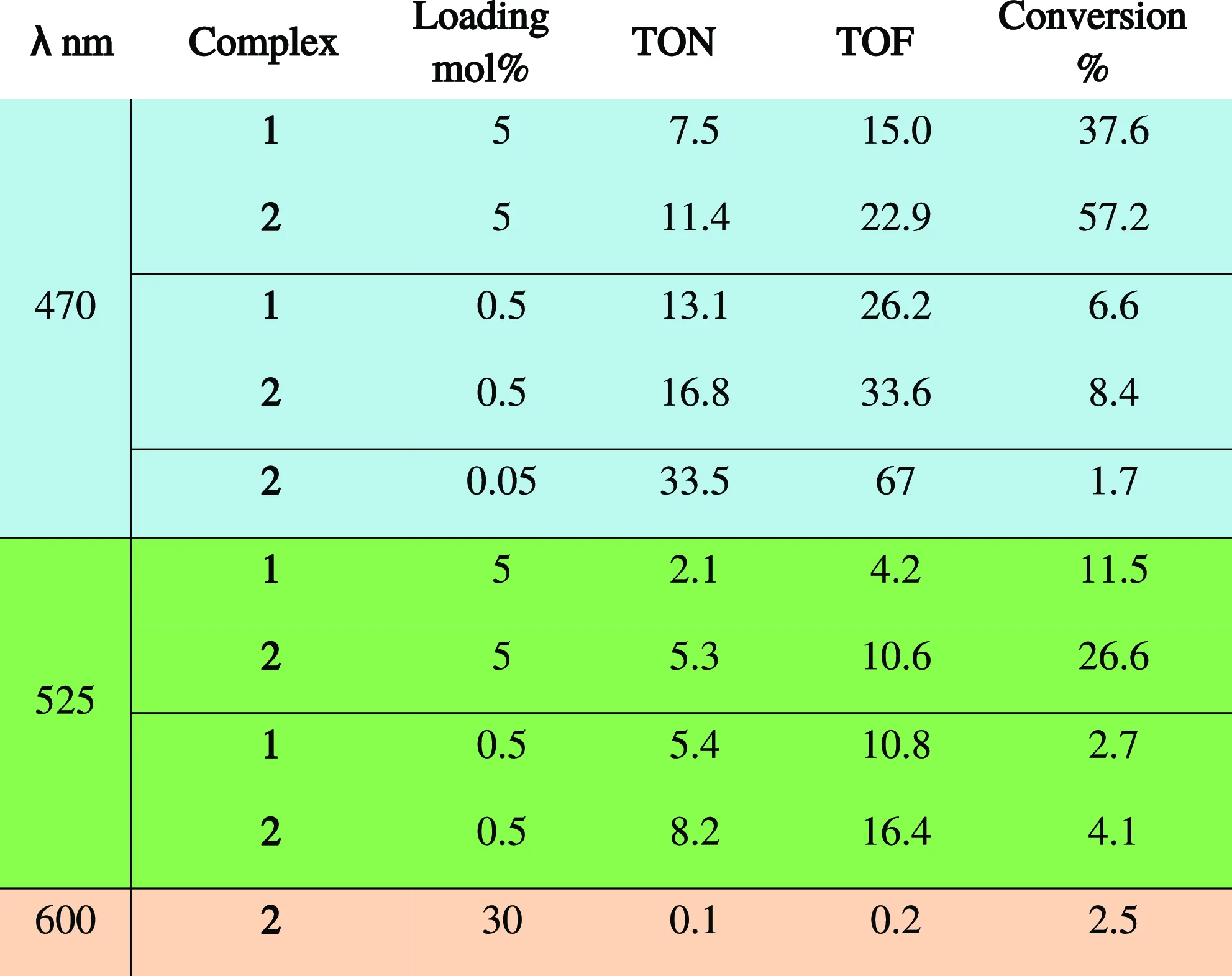
TON, TOF (h^–1^) and Conversion Values for Catalysts **1** and **2** after 30 min at Different Wavelengths and Catalyst Loadings

Because complex **2** exhibited the highest catalytic activity, subsequent experiments focused on this complex. Catalyst loading studies demonstrated that efficient turnover was maintained even at concentrations as low as 0.05 mol %, affording maximum TON and TOF values of 209.9 and 91.7 h^–1^, respectively (Figures S45 and S46). Furthermore, prolonged irradiation resulted in almost quantitative conversion (>95%) of NADH into NAD^+^ under both blue and green light (Figures S48 and S49). Control experiments confirmed that neither light alone nor the free ligand induced detectable NADH oxidation (Figures S50 and S51).

To gain mechanistic insight into the catalytic process, the reaction was monitored by ^1^H NMR under 470 nm irradiation. Progressive disappearance of the characteristic NADH resonances together with the appearance of the corresponding NAD^+^ signals confirmed the photocatalytic conversion of the reduced cofactor (Figure 6 and Figure S52). Importantly, no resonances attributable to Ru–hydride intermediates were detected throughout the reaction. Repeating the experiment under deoxygenated conditions in DMSO-*d*_6_ likewise failed to reveal hydride formation, although catalytic activity was reduced (Figure S53). These observations indicate that NADH oxidation proceeds through a photoredox electron-transfer mechanism rather than through hydride transfer and further demonstrate that the catalytic cycle retains measurable activity under oxygen-limited solution conditions.

**Figure 6:**
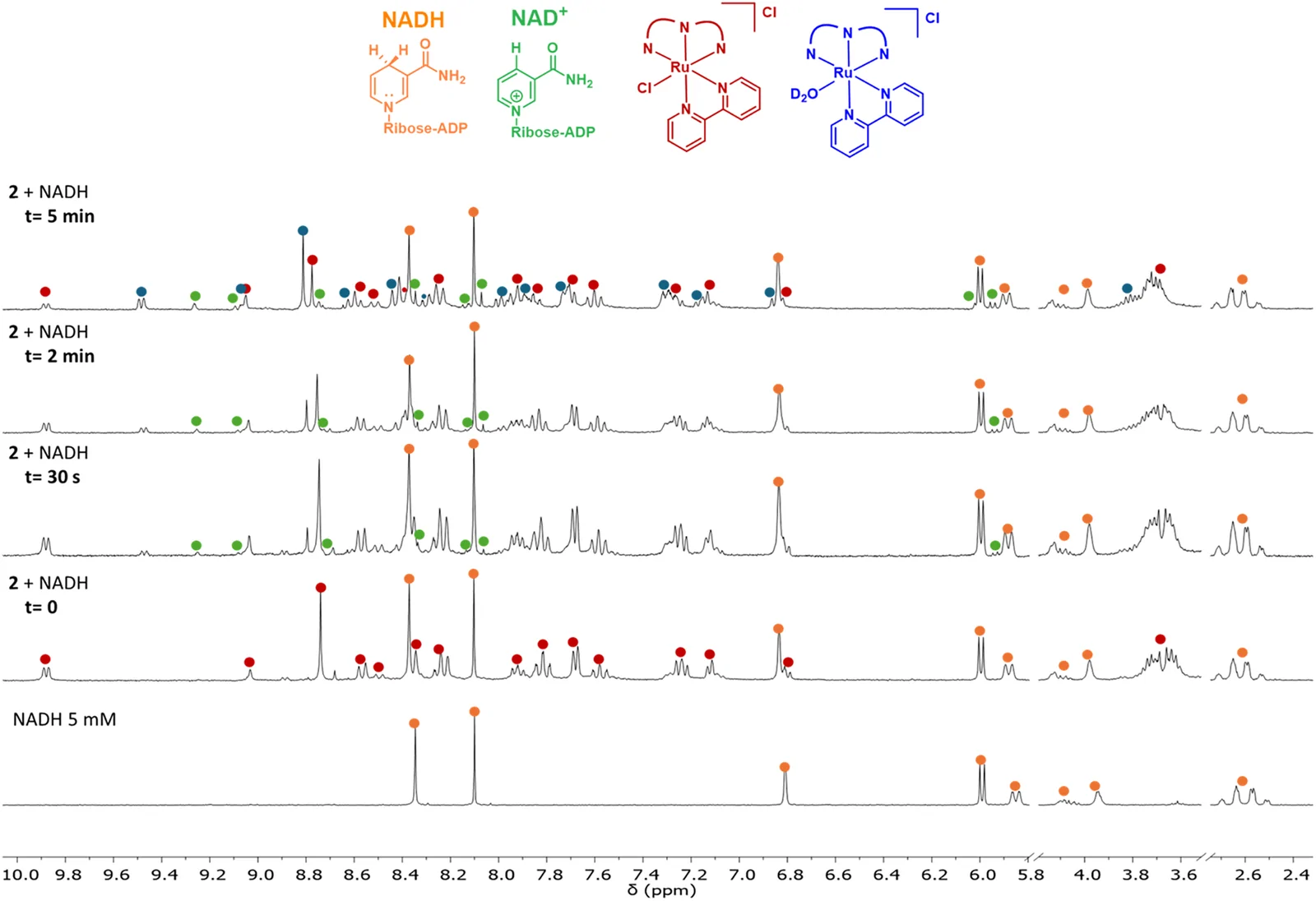
^1^H NMR in D_2_O: NADH (orange), NAD^+^ (green), [**2**-Cl]^+^ (red) and [**2**-D_2_O]^2+^ (blue).

### Reactive Oxygen Species Involved in Photocatalytic NADH Oxidation

To elucidate the mechanism of NADH photooxidation, we next investigated the involvement of reactive oxygen species (ROS) in the catalytic cycle. Hydrogen peroxide formation was first monitored during irradiation and showed a clear time-dependent increase throughout the reaction (Figure S54), providing direct evidence for continuous ROS generation during photocatalysis.

The role of molecular oxygen was subsequently examined by performing the reaction in deoxygenated PBS. Although catalytic activity decreased significantly under these conditions, NADH oxidation was not completely suppressed (Figure 7 and Figures S55–S58), indicating that oxygen plays an important, but not exclusive, role in the catalytic cycle. These observations are consistent with the reduced catalytic activity observed in the deoxygenated ^1^H NMR experiments and demonstrate that the photocatalytic system remains operative, albeit less efficiently, under oxygen-limited solution conditions.

**Figure 7:**
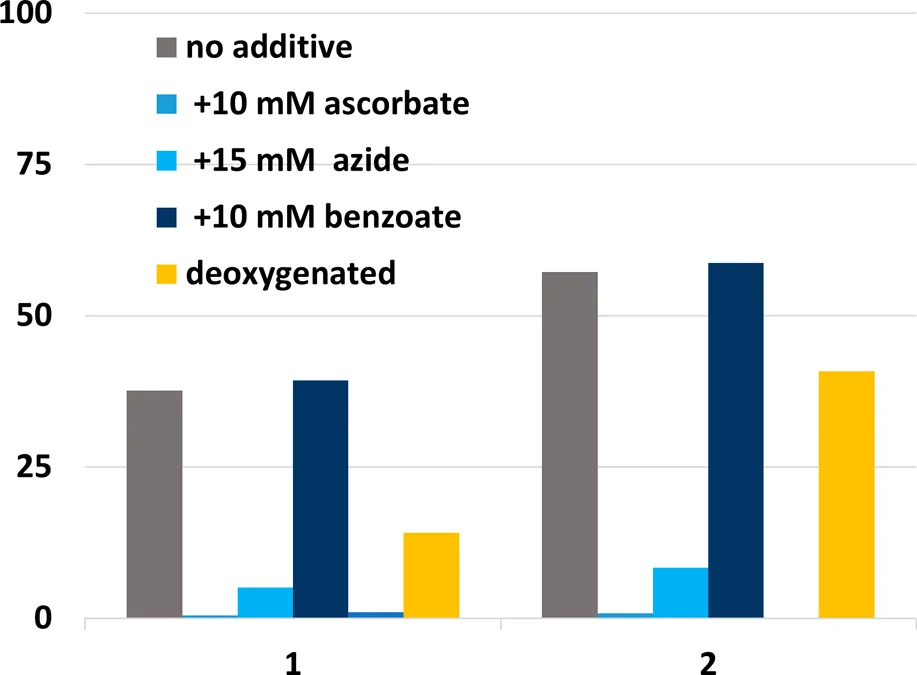
Percentage conversion after 30 min of NADH photooxidation in the presence of ROS scavengers and under deoxygenated media (λ = 470 nm and 5 mol %).

To identify the ROS involved, photocatalysis was carried out in the presence of selective scavengers under 470 nm irradiation (Figure 7 and Figures S59–S70). Addition of ascorbate, a broad-spectrum ROS scavenger, completely inhibited NADH oxidation, confirming that ROS are essential for catalysis. In contrast, quenching of singlet oxygen with sodium azide resulted in only a partial decrease in catalytic activity, indicating that ^1^O_2_ contributes to, but is not solely responsible for, the oxidation process. Furthermore, as mentioned, both **1** and **2** are capable of generating a limited amount of ^1^O_2_ (Φ_Δ_ = 5.7 and 0.6% respectively). The addition of sodium benzoate, a hydroxyl radical scavenger, had no measurable effect, excluding a significant contribution from hydroxyl radicals.

The generation of superoxide was further investigated using dihydroethidium (DHE) in the presence and absence of superoxide dismutase (SOD). In the absence of NADH, irradiation of either complex produced only a weak DHE response. In contrast, a pronounced increase in DHE fluorescence was observed in the presence of NADH, indicating that DHE oxidation is strongly enhanced during NADH photocatalysis. DHE can be oxidized by O_2_^•–^ to form a fluorescent product; however, other oxidizing species can also induce DHE oxidation.^44–46^ For this reason, SOD was included as a control, as it selectively scavenges superoxide through its dismutation to H_2_O_2_ and O_2_. Both complexes produced a strong, time-dependent increase in fluorescence intensity (Figure 8 and SI Figures S71 and S72), indicating continuous oxidation of DHE. In the presence of SOD, the fluorescence intensity decreased markedly, supporting the involvement of superoxide in the observed DHE oxidation. Taken together, the NADH-dependent enhancement of DHE oxidation and its marked suppression by SOD strongly support the generation of superoxide during NADH photocatalysis.

**Figure 8:**
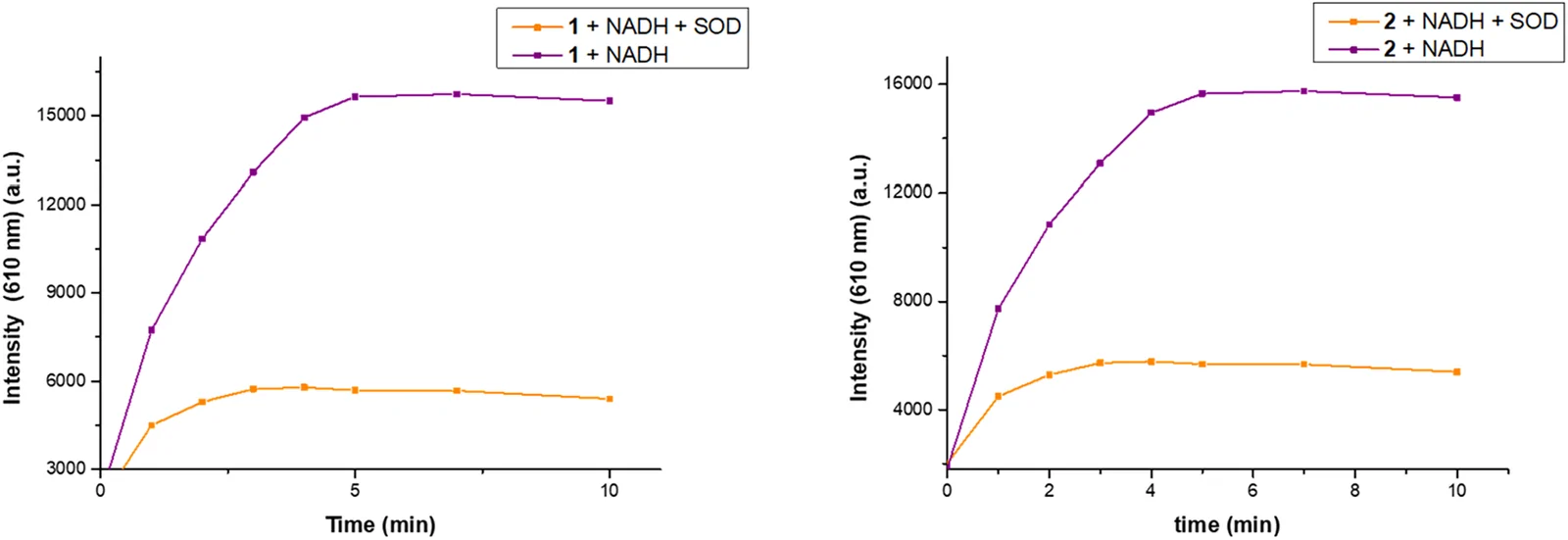
Detection of superoxide generation by DHE in presence and absence of SOD.

Taken together, these experiments reveal that molecular oxygen participates in catalyst turnover through the generation of reactive oxygen species, including superoxide, hydrogen peroxide, and, to a lesser extent, singlet oxygen. The complete suppression of catalysis by ascorbate, together with the detection of multiple ROS, indicates that photocatalytic NADH oxidation proceeds through complementary electron-transfer and energy-transfer pathways rather than through a single photochemical process.

Based on these observations and previous literature reports,^19^ we propose the catalytic mechanism shown in Figure 9. Following photoexcitation, the Ru­(II) complex accepts an electron from NADH to generate the reduced Ru­(I) intermediate. In the presence of oxygen, this species is reoxidized through electron transfer to molecular oxygen, producing superoxide and subsequently hydrogen peroxide, while energy transfer from the excited state also generates singlet oxygen. Together, these complementary pathways regenerate the catalyst and sustain photocatalytic oxidation of NADH under normoxic conditions. Although the precise regeneration pathway under oxygen-limited conditions remains to be fully established, the observed catalytic activity demonstrates that the system retains measurable photocatalytic turnover even when molecular oxygen is significantly reduced. It is important to highlight that complex **2** exhibits the highest catalytic activity, while displaying only a limited contribution from ^1^O_2_ generation (Φ_Δ_ = 0.6%). This finding further supports the hypothesis that NADH oxidation proceeds predominantly through an electron-transfer process between the photoexcited catalyst and NADH, rather than through a pathway involving ^1^O_2_.This interpretation is further supported by the interaction between NADH and the photoexcited complexes, as evidenced by the Stern–Volmer studies.

**Figure 9:**
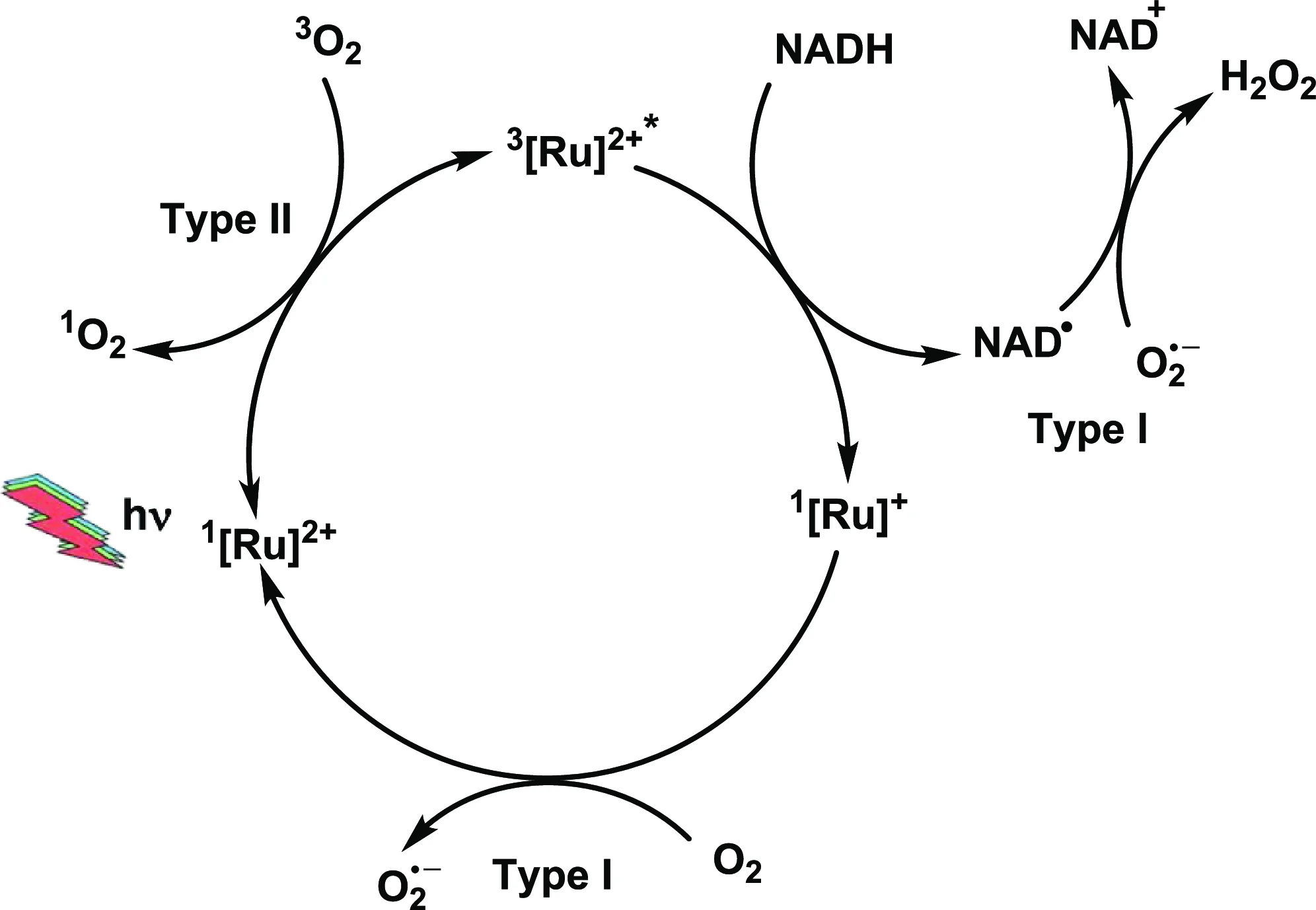
Proposed mechanism of photooxidation of NADH.

### Catalyst Robustness and the Cooperative Role of Glutathione

Successful intracellular photocatalysis requires catalysts that remain active in the presence of the high concentrations of proteins and thiols found in biological environments. We therefore investigated the catalytic performance of complex **2** in the presence of bovine serum albumin (BSA), the major serum protein, and glutathione (GSH), the most abundant intracellular thiol.

The addition of BSA had no measurable effect on NADH photooxidation (Figure 10A and Figures S73–S76), indicating that protein binding does not significantly interfere with catalyst activity. In striking contrast, the presence of GSH enhanced rather than inhibited photocatalysis. Instead of promoting catalyst deactivation through thiol coordination or excited-state quenching, glutathione increased the efficiency of NADH oxidation, demonstrating that the catalyst remains fully operational under thiol-rich conditions (see SI Figures S77–S80).

**Figure 10:**
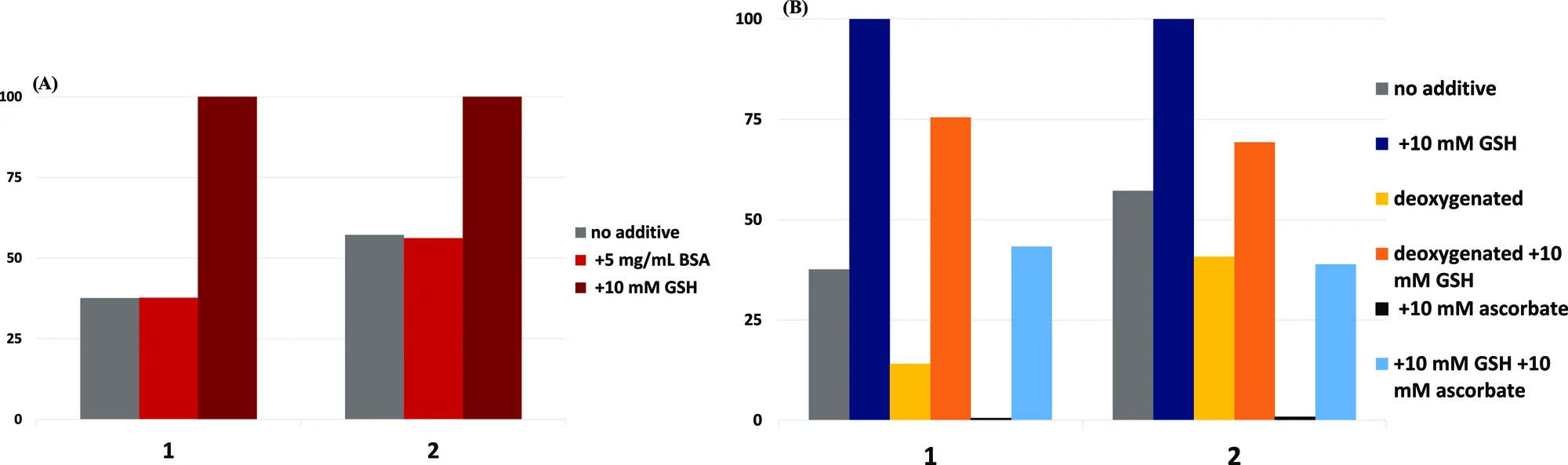
(A) Percentage conversion after 30 min of NADH photo-oxidation in the presence of BSA and GSH (λ = 470 nm and 5 mol %) (B) percentage conversion after 30 min of NADH photo-oxidation in the presence GSH, deoxygenated media and ascorbate (λ = 470 nm and 5 mol %).

To investigate the origin of this unexpected behavior, additional experiments were performed in the presence of GSH under oxygen-limited solution conditions and in the presence of both GSH and ascorbate, a broad-spectrum ROS scavenger (Figure 10B). Remarkably, in the presence of GSH the catalytic activity observed under oxygen-limited conditions exceeded that measured under normoxic conditions. Furthermore, efficient NADH oxidation was maintained even in the simultaneous presence of GSH and ascorbate, where catalytic activity was comparable to that observed under standard reaction conditions.

These observations indicate that glutathione is not simply tolerated by the catalyst but actively contributes to the photocatalytic cycle. We propose that GSH participates in reductive quenching of the excited Ru­(II) state, providing an alternative pathway for catalyst turnover that partially compensates for the reduced availability of molecular oxygen. Such a mechanism explains both the enhanced catalytic activity in the presence of GSH and the persistence of photocatalysis under oxygen-limited conditions.

Based on these results, the catalytic pathway shown in Figure 11 is proposed. In this mechanism, glutathione functions as a productive redox partner rather than a catalyst poison, promoting regeneration of the active photocatalyst while simultaneously undergoing oxidation. This cooperative behavior highlights the robustness of the catalytic system in biologically relevant media and reveals an unexpected role for glutathione in sustaining intracellular photoredox catalysis.

**Figure 11:**
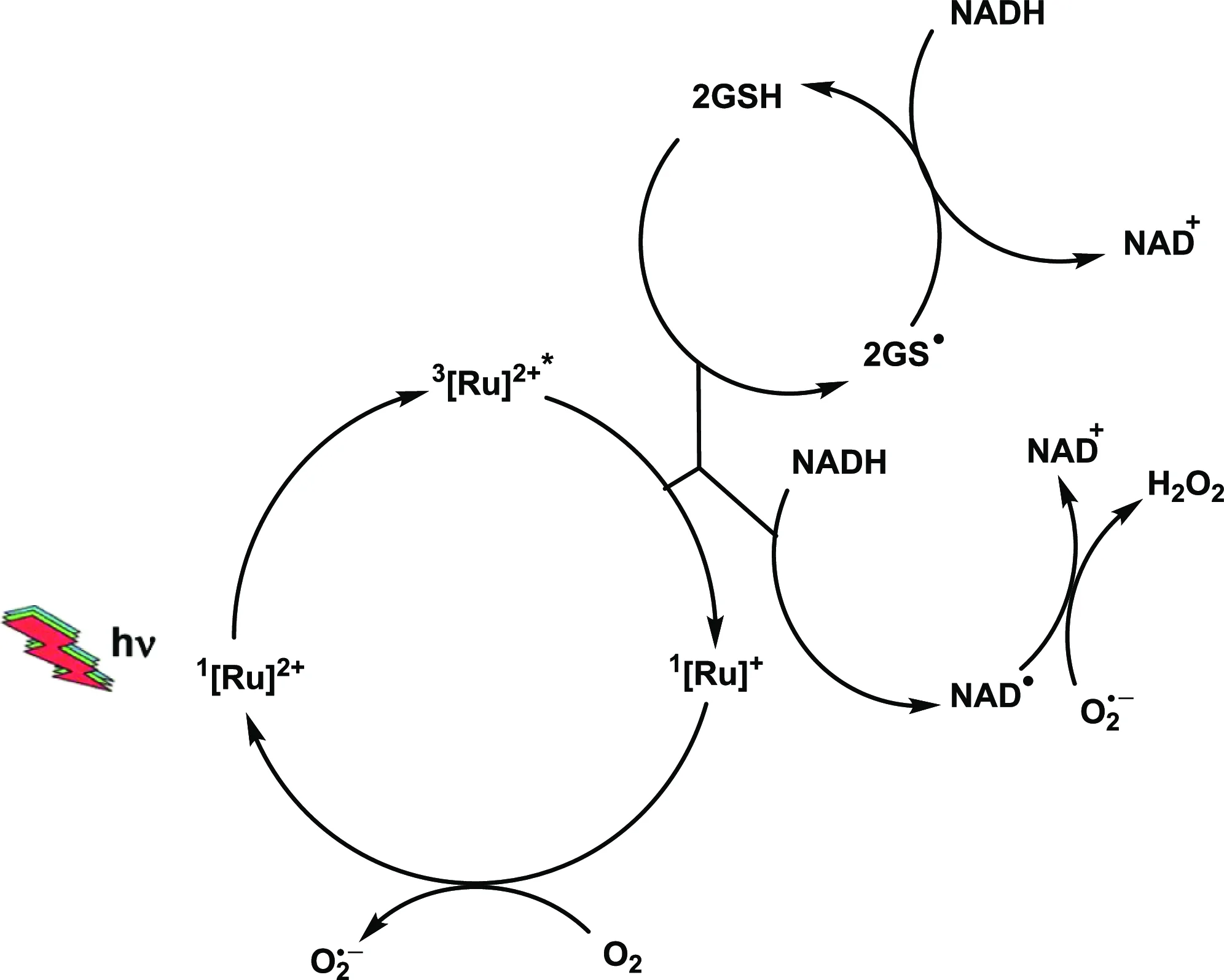
Proposed mechanism of photooxidation of NADH in the presence of GSH.

Together, these findings overturn the conventional view of glutathione as merely a deactivating agent for photocatalysts and instead demonstrate that, under appropriate conditions, it can sustain photocatalytic turnover by participating directly in the catalytic cycle.

### Photocatalytic Oxidation of Glutathione

The cooperative role of glutathione suggested by the previous experiments prompted us to investigate whether GSH is itself a photocatalytic substrate. Accordingly, the oxidation of glutathione (GSH) to glutathione disulfide (GSSG) was evaluated in PBS (pH 7.4) under blue (470 nm), green (525 nm), and red (600 nm) irradiation using complexes **1** and **2**.

GSH oxidation was monitored using Ellman’s reagent (DTNB), which reacts selectively with reduced glutathione to generate 5-thio-2-nitrobenzoate (TNB), allowing GSH consumption to be followed through the decrease of the characteristic absorption band at 412 nm (Figure 12 and Figures S81–S91). No changes were observed in the absence of light, confirming that the oxidation process is strictly photocatalytic (Figure S81).

**Figure 12:**
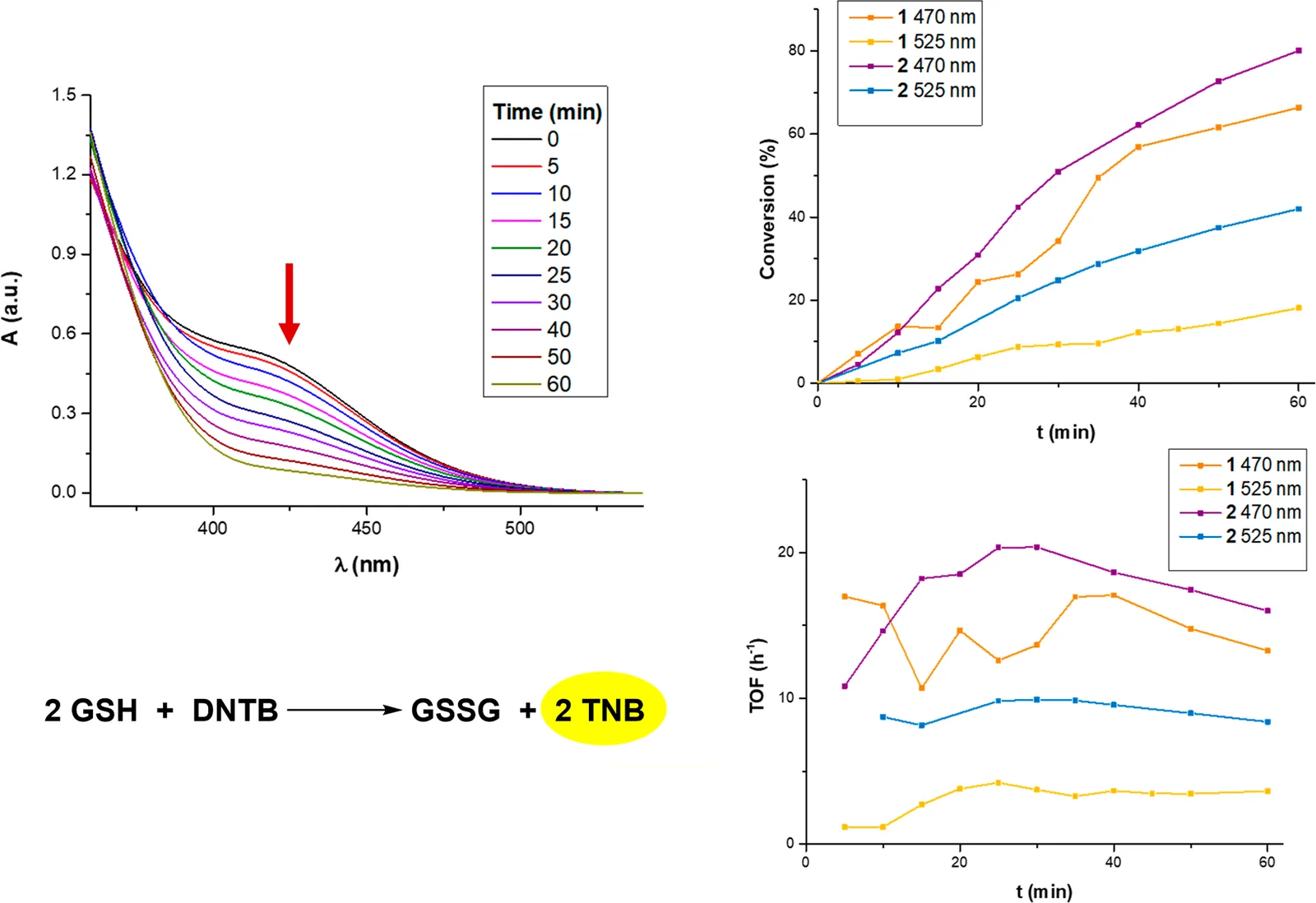
Time-resolved UV–vis spectra of GSH photo-oxidation in the presence of catalyst **2** (10 mM; 5 mol %) under 470 nm irradiation. Comparative plots of conversion and TOF for complexes **1** and **2** (20 mM; 5 mol %) under irradiation at 470 and 525 nm.

Upon irradiation, both complexes efficiently promoted GSH oxidation, with the highest catalytic activity observed under blue light and progressively lower activity under green and red irradiation. Throughout the study, complex **2** consistently exhibited slightly higher catalytic activity than complex **1**, in agreement with its superior performance in NADH oxidation (Table 2 and Figures S82–S85). Importantly, both complexes remained catalytically active under red-light irradiation, demonstrating that visible-light-driven GSH oxidation can also be achieved at wavelengths with greater tissue penetration and reduced intrinsic phototoxicity (S86 and S87).

**Table 2:**
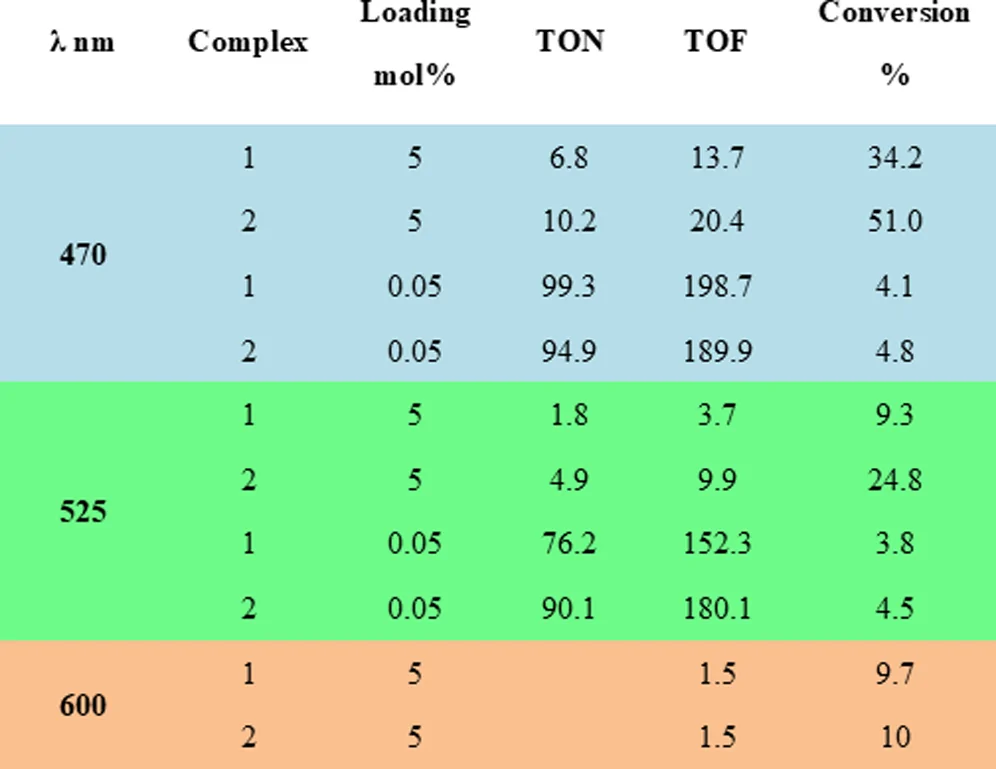
TON, TOF (h^–1^) and Conversion Values of Catalysts **1** and **2** after 30 min (470 and 525 nm) or 80 min (600 nm) of Irradiation at Different Wavelengths and Catalyst Loadings

Catalytic turnover was maintained even at catalyst loadings as low as 0.05 mol %, affording high TON and TOF values under both blue and green irradiation (Table 2). Furthermore, prolonged irradiation resulted in almost quantitative conversion (>95%) of GSH into GSSG under 470 nm irradiation (Figures S88 and S89). Control experiments confirmed that neither irradiation alone nor the free ligand promoted detectable GSH oxidation (Figures S90 and S91).

Together, these results demonstrate that the Ru­(II) complexes efficiently catalyze the oxidation of both major intracellular reducing agents, NADH and glutathione. Combined with the mechanistic studies described above, these findings support the proposal that glutathione acts as a productive substrate within the photocatalytic cycle rather than merely as a catalyst-deactivating thiol.

### Influence of Molecular Oxygen on GSH Photocatalysis and Proposed Mechanism

To further investigate the role of molecular oxygen in GSH photooxidation, catalytic experiments were performed under oxygen-limited solution conditions. As observed for NADH oxidation, reducing oxygen availability significantly reduced the catalytic efficiency but did not completely suppress GSH oxidation (Figures S92–S95). These results demonstrate that molecular oxygen is an important component of the catalytic cycle while also indicating that photocatalytic turnover can be maintained, albeit less efficiently, when oxygen availability is substantially reduced. Furthermore, as mentioned, both complexes are capable of generating ^1^O_2_, but only to a limited extent (**1** Φ_Δ_ = 5.7% and **2** Φ_Δ_ = 0.6%). Unfortunately, the generation of superoxide could not be confirmed under these conditions (SI Figures S96 and S97), as GSH can scavenge reactive oxygen species and may therefore interfere with the detection of O_2_^•–^.^47^ In addition, GS^•^ can react with DHE, giving rise to secondary oxidation products.^48^ Together with the reduced catalytic efficiency under oxygen limited conditions and the modest production of ^1^O_2_ the data support the hypothesis that GSH photooxidation proceeds through the same general photoredox pathway established for NADH oxidation.

Based on these observations, the mechanism shown in Figure 13 is proposed. Following photoexcitation of the Ru­(II) complex, glutathione is proposed to undergo reductive quenching of the excited state to generate the reduced Ru­(I) intermediate together with a glutathione-derived radical. Subsequent electron transfer from Ru­(I) to molecular oxygen would regenerate the ground-state catalyst while producing superoxide, which could be further converted into additional reactive oxygen species. In this case, complex **2** was again the more active catalyst, exhibiting the highest catalytic efficiency for both GSH and NADH photooxidation, despite its lower ^1^O_2_ generation. The consistency of this behavior across both substrates, together with the limited contribution of ^1^O_2_ generation, further supports the involvement of a common photoredox pathway in these reactions.

**Figure 13:**
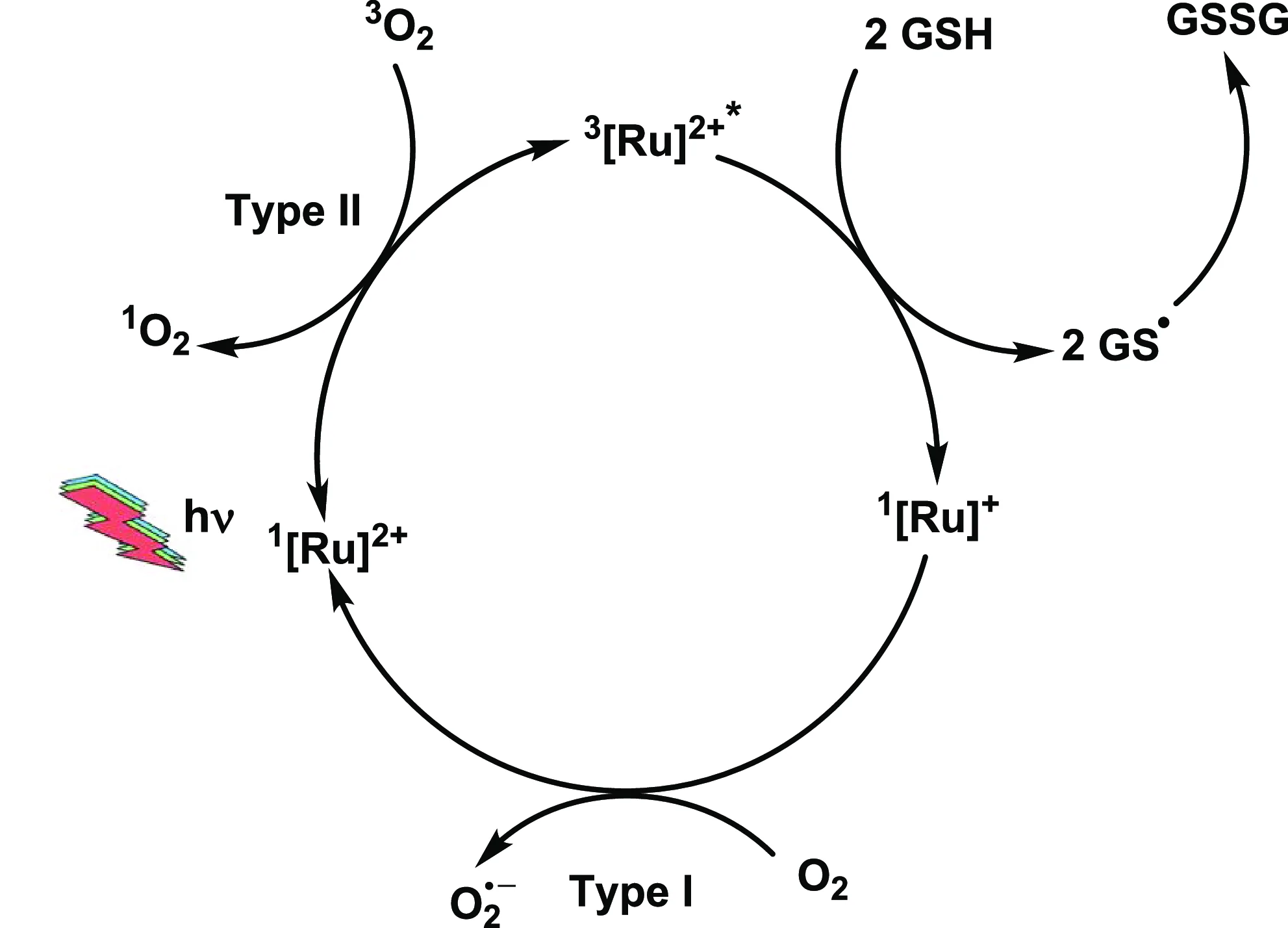
Proposed mechanism of photooxidation of GSH.

Having established the photocatalytic behavior of the complexes in solution, we next investigated whether these properties translate into intracellular activity by examining physicochemical parameters that govern biological performance, including lipophilicity, cellular uptake, and photocytotoxicity.

### DNA Binding Studies

To assess whether direct DNA interaction contributes to the biological activity of the complexes, the binding of the free ligand **L** and complexes **1** and **2** to calf thymus DNA (ct-DNA) was investigated by UV–Vis titration. Because terpyridine derivatives are known to interact with nucleic acids, these experiments were performed to distinguish between a DNA-targeting mechanism and the photocatalytic redox mechanism proposed.

All three compounds exhibited hypochromic changes upon addition of ct-DNA, indicating interaction with DNA. However, marked differences in binding affinity were observed. The free ligand **L** displayed the strongest interaction (*K*_b_ = 7.50 × 10^5^ M^–1^), whereas complexes **1** and **2** bound more weakly (*K*_b_ = 3.43 × 10^4^ and 2.28 × 10^5^ M^–1^, respectively), indicating that coordination to Ru­(II) significantly reduces the DNA-binding ability of the terpyridine scaffold, most likely as a consequence of the decreased molecular planarity.

Because complex **2** undergoes photoaccelerated aquation, generating a potentially coordinatively unsaturated Ru­(II) center, we further examined whether irradiation promotes covalent interaction with DNA. UV–Vis titration performed under 470 nm irradiation revealed no significant change in the binding mode, with a binding constant (*K*_b_ = 6.18 × 10^5^ M^–1^) remaining consistent with intercalative association rather than covalent coordination (SI Figures S98–S99). However, the spectral changes did not provide evidence for the formation of a covalent Ru–DNA adduct.

To further investigate the binding mode using an orthogonal method, ethidium bromide (EB) displacement experiments were performed. Both complexes **1** and **2** displaced DNA-bound EB, with apparent binding constants (*K*_app_) of 5.23 × 10^7^ and 2.30 × 10^7^ M^–1^, respectively (SI Figures S100 and S101). The free ligand also displaced EB, although less efficiently (*K*_app_ = 4.48 × 10^5^ M^–1^). These results provide additional evidence supporting an intercalative contribution to the interaction of the compounds with DNA. Interestingly, although the ligand exhibited a higher binding constant in the UV–Vis titration, it was less efficient in displacing EB. This difference may reflect the contribution of additional factors, including the charge of the compounds and electrostatic interactions with the negatively charged DNA backbone.

Overall, the combined UV–Vis titration and EB displacement experiments support DNA interaction with an intercalative contribution, while no evidence for covalent DNA binding was observed.^49^

### Lipophilicity, Cellular Uptake, and Photocytotoxicity

Having established the photocatalytic performance of the complexes in solution, we next investigated whether these properties translate into biological activity. Because intracellular photocatalysis depends not only on catalytic efficiency but also on cellular accessibility, we first evaluated the lipophilicity and uptake of the two complexes. Both complexes exhibited strongly negative water–octanol partition coefficients (log *P* ≈ −2), reflecting the high hydrophilicity imparted by the PEG substituents (eq S13). Although PEGylation improves aqueous solubility and compatibility with biological media, such low lipophilicity is expected to limit passive membrane permeation. Consistent with this expectation, ICP–OES measurements in MCF-7 cells (100 μM, 24 h) revealed low intracellular accumulation for both compounds. Complexes **1** and **2** reached intracellular concentrations of 3.100 ± 0.036 and 2.174 ± 0.046 nmol per 7 × 10^6^ cells, corresponding to uptake efficiencies of only 0.34% and 0.24%, respectively. Nevertheless, because photocatalysts operate catalytically rather than stoichiometrically, even these low intracellular concentrations may be sufficient to perturb cellular redox homeostasis.

Despite their similarly low uptake, the two complexes displayed strikingly different biological behavior (Table 3). Under dark conditions, both compounds were essentially nontoxic (IC_50_ ≥ 200 μM) toward MCF-7, MDA-MB-231, and A549 cells. Upon irradiation, however, only complex **1** exhibited significant photocytotoxicity. Under blue light (470 nm, 10 min), moderate activity was observed in MCF-7 cells (IC_50_ = 82 μM), whereas complex **2** remained inactive. Because blue light itself induces measurable phototoxicity, subsequent studies were performed using green (525 nm, 30 min) and red (600 nm, 1 h) irradiation. Under these conditions, complex **1** displayed progressively enhanced activity, reaching IC_50_ values of 35.7 μM under green light and 29–43 μM under red light across the three tumor cell lines. The corresponding phototoxicity indices confirmed efficient light activation, with PI values exceeding 7 in MCF-7 cells and approximately 5 in MDA-MB-231 and A549 cells. In contrast, complex **2** remained essentially nonphototoxic under all irradiation conditions. To assess the selectivity of the complexes toward nontumor cells, NHDF-Ad fibroblasts were employed to determine whether, in addition to their light-dependent activation, the complexes also exhibit a favorable selectivity profile toward nontumor cells. Their cytotoxicity was evaluated under both dark conditions and upon irradiation at 600 nm. The results obtained are highly promising, as neither complex exhibited detectable cytotoxicity in fibroblasts, either in the dark or upon irradiation at 600 nm. These findings suggest that both complexes display a favorable safety profile toward nontumor cells under the experimental conditions investigated.

**Table 3:**
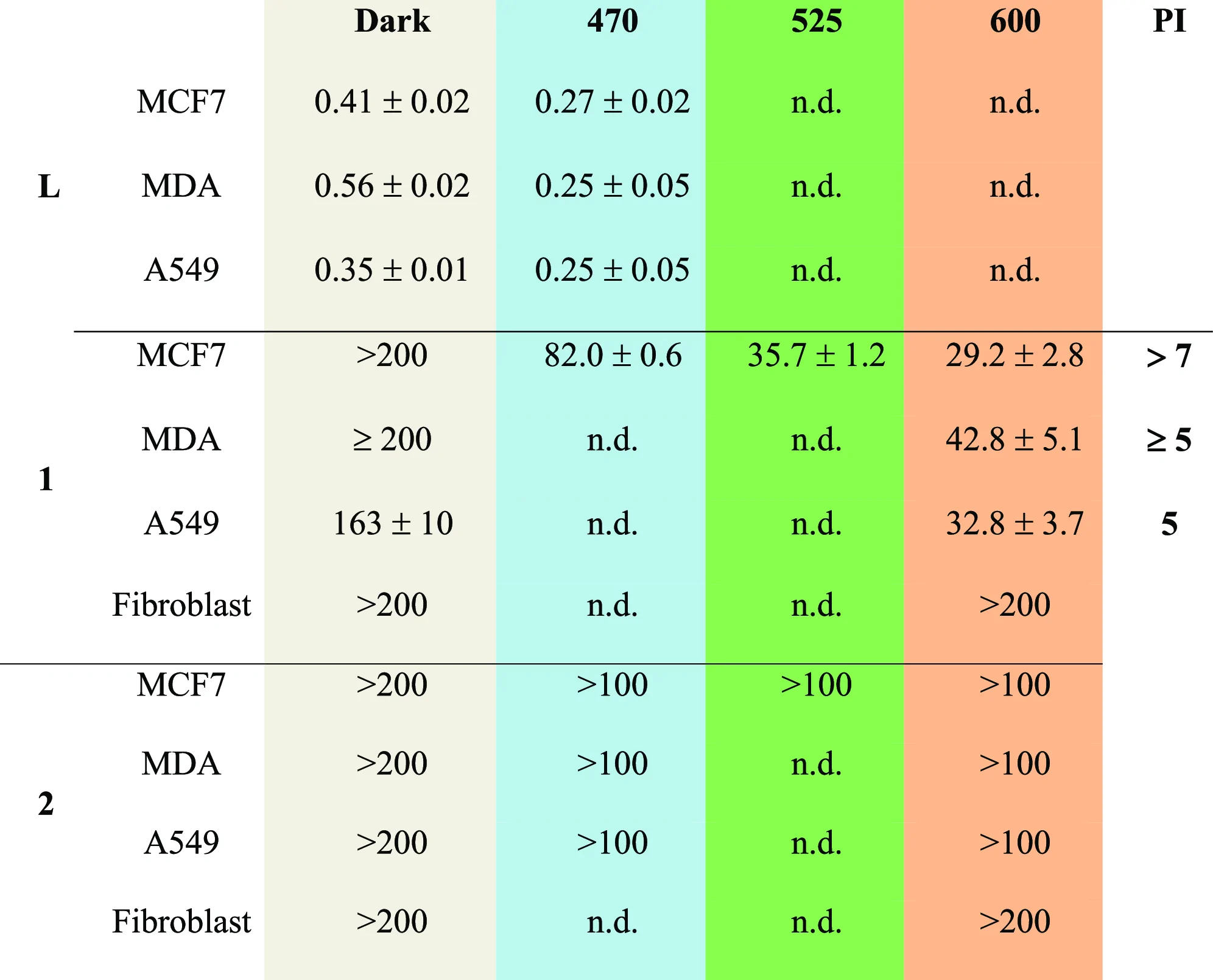
IC_50_ Values (μM) of **L**, **1**, **2** in Dark and under Irradiation of 470, 525, and 600 nm^a^

a n.d. = not determined.

The divergent behavior of the two complexes is particularly revealing. Although complex **2** is the more efficient photocatalyst in the cell-free oxidation of NADH and glutathione, this superior catalytic activity is not translated into cellular photocytotoxicity. These results demonstrate that intracellular photocatalytic performance is governed not only by catalytic efficiency measured in solution but also by factors such as photostability, intracellular accessibility, and productive interaction with cellular redox environments. Consequently, catalytic activity determined in simplified model systems does not necessarily predict biological performance. This unexpected discrepancy prompted us to investigate the intracellular consequences of photocatalysis in greater detail in order to identify the factors responsible for the markedly different biological activities of the two complexes.

#### Disruption of Intracellular Redox Homeostasis

The solution studies demonstrated that complex 1 efficiently catalyzes the oxidation of both NADH and glutathione under visible-light irradiation. We next investigated whether these photocatalytic processes also occur in living cells by quantifying the intracellular NAD^+^/NADH and GSH/GSSG redox couples in MCF-7 cells following red-light irradiation (600 nm).

### Intracellular Disruption of the NAD^+^/NADH Balance

The intracellular concentrations of NADH, NAD^+^, and their ratio are summarized in Table 4 (Figure S102 and eqs S14–S15). Irradiation alone produced no significant changes in the cellular NAD^+^/NADH balance. In contrast, treatment with complex **1** induced a clear shift toward the oxidized state, an effect that was further enhanced upon red-light irradiation. While the NAD^+^/NADH ratio increased from 0.50 in untreated cells to 0.86 in the dark in the presence of complex **1**, photoactivation further increased this value to 1.09. These results demonstrate that the photocatalytic activity observed in solution is translated into living cells, producing a measurable perturbation of intracellular NADH-dependent redox homeostasis.

**Table 4:** Intracellular Concentrations (pmol per 10^6^ Cells)^a^

	*C*_Dark_	*C*_600_	1_Dark_	1_600_
NADH	185 ± 15	177 ± 19	204 ± 20	190 ± 34
NAD^+^	92 ± 13	97 ± 13	175 ± 16	206 ± 25
$\frac{{\mathrm{NAD}}^{+}}{\mathrm{NADH}}$	0.50 ± 0.08	0.55 ± 0.09	0.86 ± 0.12	1.09 ± 0.23

a C = control; **1** = complex **1** (2 × IC_50_).

### Intracellular GSH Depletion

Because complex **1** efficiently oxidized glutathione in solution, we next examined whether intracellular glutathione was similarly affected (Figure S103 and eq S16). As summarized in Table 5, irradiation of cells treated with complex **1** produced a pronounced depletion of reduced glutathione accompanied by a marked increase in the GSSG/GSH ratio. Whereas untreated cells displayed a GSSG/GSH ratio of 0.66 following irradiation, treatment with complex **1** increased this value to 4.76, reflecting extensive oxidation of the major intracellular antioxidant pool. Importantly, this effect was achieved using only 2 × IC_50_ of the photocatalyst, demonstrating efficient intracellular photocatalytic turnover despite the relatively low cellular uptake of the complex.

**Table 5:** Intracellular Concentrations (nmol per 10^6^ Cells)^a^

	*C*_Dark_	*C*_600_	1_Dark_	1_600_
GSSG	9.99 ± 0.54	7.31 ± 0.01	4.82 ± 0.41	6.72 ± 1.04
GSH	19.07 ± 0.89	11.08 ± 1.13	7.43 ± 0.71	1.41 ± 0.15
$\frac{\mathrm{GSSG}}{\mathrm{GSH}}$	0.52 ± 0.04	0.66 ± 0.07	0.65 ± 0.08	4.76 ± 0.89

a C = control; **1** = complex **1** (2 × IC_50_).

The simultaneous oxidation of the NADH and glutathione redox couples provides direct evidence that the photocatalytic mechanism established in solution remains operative in living cells. By perturbing both metabolic reducing power and antioxidant capacity, complex **1** effectively rewires intracellular redox homeostasis, creating conditions that strongly favor oxidative stress and ultimately sensitize cancer cells to photocatalytic cell death.

Considering the substantially larger changes observed in the intracellular NAD^+^/NADH and GSH/GSSG pools under complex and light conditions, compared with the corresponding dark and light-only controls, demonstrate that the redox perturbation is predominantly photoinduced.

### Intracellular ROS Generation and Mitochondrial Dysfunction

To further elucidate the origin of the different biological activities of complexes **1** and **2**, intracellular ROS production and mitochondrial membrane potential (ΔΨ_m_) were analyzed by flow cytometry under dark conditions and following red-light irradiation (600 nm, 1 h). Neither complex induced significant mitochondrial depolarization in the dark (Figure 14). Upon irradiation, however, complex **1** produced a marked loss of mitochondrial membrane potential, with approximately 54% of the cell population displaying ΔΨ_m_ depolarization. In contrast, complex **2** had no measurable effect on mitochondrial integrity under either condition. These results demonstrate that only complex **1** is capable of triggering mitochondrial dysfunction following photoactivation.

**Figure 14:**
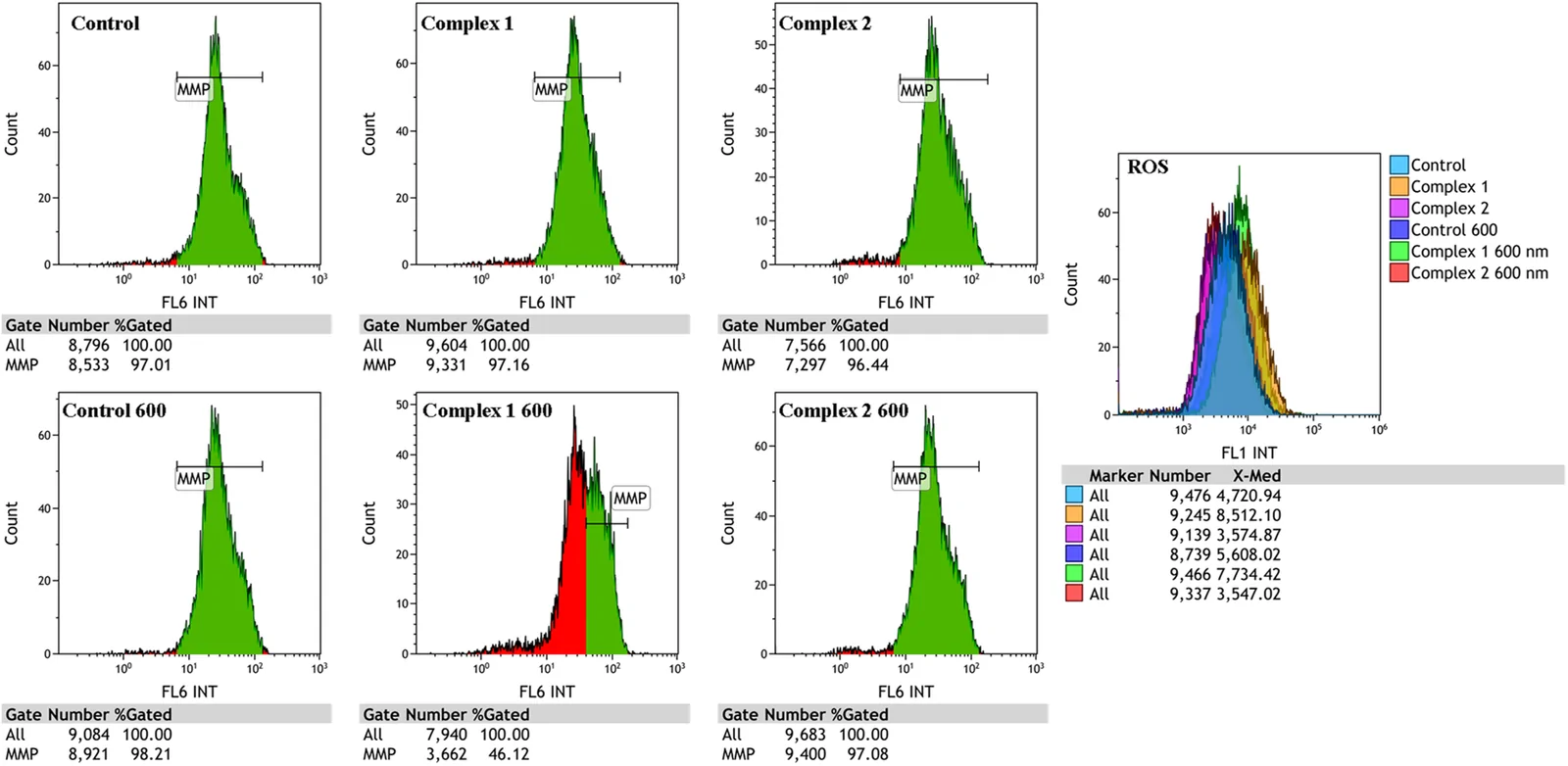
Flow cytometry experiments to determine mitochondrial ΔΨ and ROS.

A similar trend was observed for intracellular ROS production. Complex **2** failed to induce detectable ROS generation either in the dark or upon irradiation, whereas complex **1** produced measurable ROS under dark conditions that increased substantially following red-light exposure (Figure 14). This behavior closely parallels the intracellular redox measurements described above, where complex **1** produced a progressive disruption of both the NAD^+^/NADH and GSH/GSSG redox couples, while complex **2** showed no detectable biological activity.

Taken together, these results establish a clear relationship between intracellular photocatalysis and biological response. The enhanced ROS production induced by complex **1** provides a mechanistic explanation for its ability to disrupt mitochondrial function, perturb intracellular redox homeostasis, and ultimately induce photocytotoxicity. Conversely, despite exhibiting superior catalytic activity in cell-free assays, complex **2** fails to generate sufficient intracellular oxidative stress to trigger these downstream biological effects. These findings further demonstrate that efficient photocatalysis in solution alone is insufficient to predict intracellular performance and emphasize the importance of productive access to cellular redox environments.

### Mechanism of the Intracellular Photocatalysis

Collectively, the spectroscopic, photocatalytic, and biological studies support a unified mechanism in which Ru­(II)-mediated photocatalysis disrupts intracellular redox homeostasis through complementary photodynamic (PDT) and photocatalytic (PCT) pathways (Figure 15). Upon visible-light excitation, the Ru­(II) complex undergoes electron transfer with intracellular reducing agents, initiating a catalytic cycle that promotes the oxidation of both NADH and glutathione while simultaneously generating reactive oxygen species through Type I and Type II (^1^O_2_ Φ_Δ_ = 5.7 (**1**) and 0.6% (**2**)) photochemical processes. The resulting depletion of the two principal intracellular redox buffers, together with continuous ROS production, amplifies oxidative stress, ultimately leading to mitochondrial dysfunction and photocytotoxicity. An important mechanistic finding of this work is that glutathione does not simply act as a competing reductant or catalyst poison. Instead, the experimental evidence supports its participation in reductive quenching of the excited Ru­(II) state, thereby promoting catalyst turnover while undergoing oxidation to GSSG. This cooperative behavior explains the enhanced catalytic activity observed in the presence of GSH and the persistence of photocatalysis under oxygen-limited solution conditions. Comparison of complexes **1** and **2** further demonstrates that efficient photocatalysis in homogeneous solution is not, by itself, sufficient to achieve intracellular activity. Although complex **2** exhibits superior catalytic performance toward NADH and glutathione oxidation in solution, it fails to produce measurable intracellular redox perturbation or photocytotoxicity. In contrast, complex **1**, despite its lower intrinsic catalytic activity, efficiently disrupts the intracellular NAD^+^/NADH and GSH/GSSG redox couples, generates ROS, induces mitochondrial depolarization, and ultimately produces selective photocytotoxicity. These observations demonstrate that intracellular photocatalytic performance is governed not only by catalytic efficiency but also by photostability and productive access to cellular redox environments. The proposed mechanism is summarized in Figure 15, which integrates the solution photochemistry with the intracellular biological response and illustrates how complementary PDT and photocatalytic pathways cooperate to achieve efficient disruption of cancer cell redox homeostasis.

**Figure 15:**
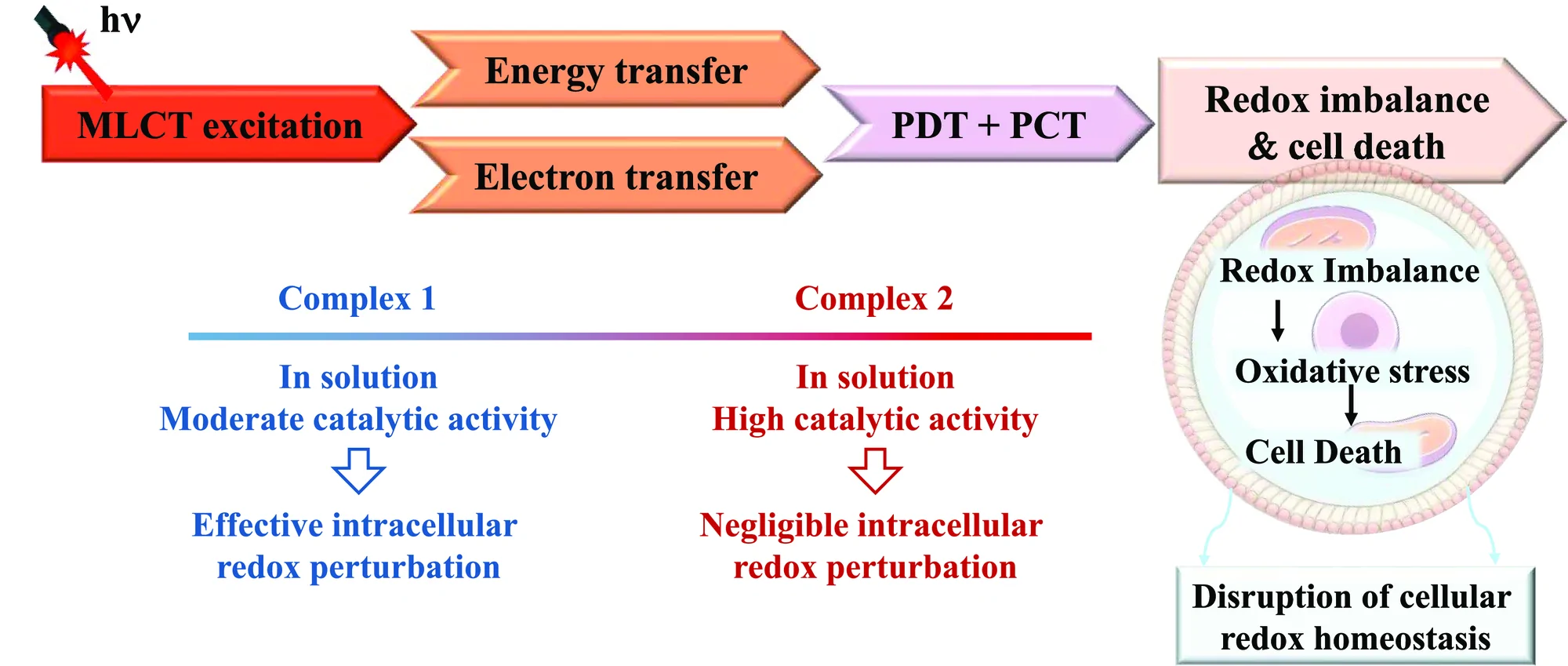
Proposed mechanism for intracellular Ru­(II)-mediated photocatalytic disruption of cancer redox homeostasis, and the distinct intracellular behaviors of complexes **1** and **2**.

## Conclusions

This work demonstrates that PEGylated Ru­(II) terpyridine complexes function as visible-light-activated intracellular photocatalysts capable of simultaneously perturbing the two principal intracellular redox buffers, NADH and glutathione. Upon photoexcitation, the Ru­(II) complexes undergo photoredox processes consistent with the involvement of both electron- and energy-transfer pathways, promoting catalytic oxidation of NADH and GSH together with the generation of reactive oxygen species. The combined depletion of metabolic reducing power and antioxidant capacity results in amplified oxidative stress and disruption of intracellular redox homeostasis.

A key mechanistic finding of this study is that glutathione does not simply act as a competing thiol or catalyst poison. Instead, the experimental evidence supports its participation in reductive quenching of the excited Ru­(II) state, thereby promoting catalyst turnover and sustaining photocatalytic activity under oxygen-limited solution conditions. This cooperative role of glutathione broadens the operational scope of Ru­(II)-based photoredox catalysis in biologically relevant environments.

The intracellular studies further demonstrate that red-light activation of complex **1** produces simultaneous disruption of the NAD^+^/NADH and GSH/GSSG redox couples, accompanied by ROS generation, mitochondrial membrane depolarization, and selective photocytotoxicity, despite the low cellular uptake of the catalyst. Importantly, neither complex showed detectable cytotoxicity in nontumoral fibroblasts under dark or irradiated conditions (IC_50_ > 200 μM), supporting preferential photocytotoxicity toward cancer cells under the conditions investigated. Comparison of complexes **1** and **2** further reveals that superior catalytic activity measured in homogeneous solution does not necessarily translate into greater intracellular activity. Instead, effective intracellular photocatalysis depends on a combination of catalytic efficiency, photostability, and productive access to intracellular redox-active environments.

Overall, this work establishes Ru­(II) photocatalysis as a promising strategy for intracellular redox modulation and provides general design principles for the development of next-generation metal-based photocatalysts. More broadly, it demonstrates that combining photodynamic and photocatalytic pathways offers an effective approach to amplifying oxidative stress through complementary mechanisms, opening new opportunities for light-activated therapies based on catalytic control of cellular redox homeostasis.

## Experimental Section

### General Procedures

All reactions were carried out under an argon atmosphere using standard Schlenk techniques. Solvents were dried and purified by standard procedures prior to use. Unless otherwise stated, reagents were obtained from commercial sources, Merck or BLD, and used without further purification. Reaction progress was monitored by thin-layer chromatography (TLC). Products were purified by column chromatography on aluminum oxide using the appropriate solvent mixtures as eluents. NMR spectra were recorded on Bruker ARX300, AV300 or AV400 spectrometers. ^1^H NMR spectra were recorded at 300 or 400 MHz; ^13^C­{^1^H}-APT NMR spectra were recorded at 75 or 101 MHz; ^19^F­{^1^H} NMR spectra were recorded at 282 MHz. A Bruker MicroToF-Q spectrometer was used for High-resolution mass spectra-ESI (HRMS-ESI) equipped with an API-ESI source and a QToF mass analyzer, both allowing a maximum error in the measurement of 5 ppm. FT/IR spectra were recorded on a Jasco FT/IR-6600 spectrometer. UV–vis spectra were recorded with JASCO V-780 spectrophotometer. Solutions were irradiated using Kessil PR160L-470 nm (137 mW/cm^2^, blue light), PR160L-525 nm (137 mW/cm^2^, green light) and a homemade 600 nm LED array (16.25 mW/cm^2^, red light). Doses can be calculated at each time using the following equation: Dose (J/cm^2^) = Power Density (mW/cm^2^) × time (s) × 0.001. Luminescence spectra and lifetime measurements were recorded with a FluoTime300 PicoQuant spectrometer. Quantum yields were measured by the absolute method using a Hamamatsu Quantaurus-QY C11347 compact one-box absolute quantum yield measurement system. Cyclic voltammetry spectra were recorded with a PalmSens 4 potentiostat.

### Synthesis and Characterization

#### Synthesis of L

2,2′:6′,2″-terpyridine-4′-carboxylic acid (300 mg, 1.08 mmol) and DIPEA (*N*,*N*-Diisopropylethylamine) (0.47 mL, 2.7 mmol) were dissolved in dry DMF (5 mL). To this solution HATU (Hexafluorophosphate Azabenzotriazole Tetramethyl Uronium) (616 mg, 1.62 mmol) was added and was left to stir under argon atmosphere for 30 min. To the resulting brown suspension, a 2 M solution of propargyl-PEG2-amine (172 μL, 1.2 mmol) in dry methanol was added dropwise and the resulting orange solution was left to stir overnight at room temperature and under argon atmosphere. Then, the reaction mixture was quenched with water (150 mL) and was left to stand for 15 min to allow the fine precipitate to aggregate and settle, facilitating subsequent filtration. The solid was washed with water (10 mL × 3) and with a *N*-pentane/ether (5:1) mixture (70 mL). The obtained product was a brown and moist solid, which was dried under vacuum for 24 h. The crude product was purified by column chromatography on aluminum oxide (Al_2_O_3_) using a gradient of DCM and MeOH (from 100:0 to 98:2) and the progress was monitored by TLC (*R*_f(DCM/MeOH99:1)_ = 0.5). The desired fraction was collected, and the solvent was removed under vacuum to afford **L** as a white solid (250.7 mg, 57.7% yield). ^1^H NMR (400 MHz, MeOD) δ (ppm): 8.79 (s, 2H, Tpy 5), 8.72 (ddd, *J =* 4.8, 1.8, 0.9 Hz, 2H, Tpy 1), 8.65 (dt_pseudo_, *J =* 8.0, 1.1 Hz, 2H, Tpy 4), 8.01 (td_pseudo_, *J =* 7.8, 1.8 Hz, 2H, Tpy 3), 7.50 (ddd, *J =* 7.5, 4.8, 1.2 Hz, 2H, Tpy 2), 4.23 (d, *J =* 2.4 Hz, 2H, PEG 10), 3.79–3.71 (m, 6H, PEG 7–9), 3.71–3.64 (m, 2H, PEG 6), 2.83 (t, *J =* 2.4 Hz, 1H, alkyne 11). ^13^C­{^1^H}-APT NMR (101 MHz, MeOD) δ (ppm): 168.5 (C=O d), 157.7 (Tpy a/b) 156.7 (Tpy a/b), 150.3 (Tpy 1), 145.6 (Tpy c), 138.7 (Tpy 3), 125.7 (Tpy 2), 122.7 (Tpy 4), 119.7 (Tpy 5), 76.0 (alkyne e), 71.1, 70.4, 70.1 (PEG 7,8,9), 59.1 (PEG 10), 41.1 (PEG 6). HRMS (ESI^+^): *m*/*z* [M+H]^+^ calc. for [C_23_H_23_N_4_O_3_]^+^ 403.1765; found 403.1767. *m*/*z* [M+Na]^+^ calc. for [C_23_H_22_N_4_NaO_3_]^+^ 425.1584; found 425.1586. Anal. Calcd for C_23_H_22_N_4_O_3_ (%): C, 68.64; H, 5.51; N, 13.92; Found: C, 68.14; H, 5.56; N, 13.68.

#### Synthesis of Complex **1**

**L** (177 mg, 0.44 mmol) was dissolved in deoxygenated dry EtOH (20 mL), the solution was heated to reflux (80 °C) and left to stir under argon atmosphere until complete dissolution. Then RuCl_3_·*x*H_2_O (55.6 mg, 0.22 mmol) and 5 drops of *N*-ethylmorpholine were added to the solution and the reaction was refluxed (80 °C) overnight under an argon atmosphere. Subsequently, the reaction mixture was cooled to room temperature, and the solvent was removed under vacuo. The resulting product was purified by silica column chromatography. The column was first eluted with EtOH to remove an initial yellow band, which was discarded. Then, the eluent was switched to MeCN/H_2_O/saturated NaNO_3_ (aq) (20:3:1) and the desired fraction was eluted as a red band. The obtained fraction was evaporated to dryness, and the resulting red solid was dissolved in DCM and filtered through a pad of Celite. The filtrate was then dried over anhydrous MgSO_4_, filtered and concentrated under reduced pressure. Precipitation with ether afforded [Ru­(L)_2_]­(Cl)_2_ as a red solid (121.3 mg; 56.5% yield). [Ru­(L)_2_]­(Cl)_2_ (120.5 mg, 0.123 mmol) and Ag­(OTf) (63.4 mg, 0.246 mmol) were dissolved in DCM (15 mL) and the reaction mixture was stirred at room temperature for 3 h in the absence of light. Upon completion, the crude was filtered through a Celite pad and washed with MeOH. The filtrate was then evaporated to dryness under reduced pressure, and the resulting solid was recovered by adding ether and scraping it from the flask walls, as it adhered strongly upon drying. The obtained solid was then dried under vacuum for several days to afford [Ru­(L)_2_]­(OTf)_2_ as a crystalline red solid (106.7 mg; 71.8% yield). ^1^H NMR (300 MHz, MeOD) δ (ppm): 9.35 (s, 4H, Tpy 5), 8.77 (d, *J =* 8.2 Hz, 4H, Tpy 4), 8.03 (tt_pseudo_, *J =* 7.9, 1.4 Hz, 4H, Tpy 3), 7.50 (d, *J =* 5.5 Hz, 4H, Tpy 1), 7.28 (ddd, *J =* 7.3, 5.6, 1.3 Hz, 4H, Tpy 2), 4.24 (d, *J =* 2.4 Hz, 2H, PEG 10), 3.95–3.75 (m, 14H, PEG 7–9), 3.73–3.67 (m, 2H, PEG 6), 2.87 (t, *J =* 2.4 Hz, 1H, alkyne 11). ^13^C­{^1^H}-APT NMR (75 MHz, MeOD) δ (ppm): 166.3 (Tpy C=O d), 166.3 (Tpy C=O d′), 159.1 (Tpy a), 156.9 (Tpy b), 153.4 (Tpy 1), 143.0 (Tpy c), 142.9 (Tpy c′), 139.7 (Tpy 3), 129.2 (Tpy 2), 126.1 (Tpy 4), 122.9 (Tpy 5), 122.9 (Tpy 5′), 76.1 (PEG alkyne 11), 73.6 (PEG 6), 71.2 (PEG 7–9), 70.4 (PEG 7’-9’), 70.4 (PEG 7–9), 70.3 (PEG e), 62,3 (PEG 7–9), 59.2 (PEG 10), 49.0 (q, MeOD), 41.7 (PEG 7–9), 41.6 (PEG 7′–9′). ^19^F­{^1^H} NMR (282 MHz, MeOD) δ (ppm): −80.1. HRMS (ESI^+^): *m*/*z* [M]^2+^ calc for [C_46_H_44_N_8_O_6_Ru]^2+^ 453.1208; found 453.1221. Anal. Calcd for C_48_H_44_F_6_N_8_O_12_RuS_2_ (%): C, 47.88; H, 3.68; N, 9.31; S, 5.33; found: C, 47.40; H, 4.18; N, 9.51; S, 5.03.

#### Synthesis of Complex **2**

**L** (150 mg, 0.373 mmol) was dissolved in EtOH (8 mL) and the mixture was refluxed (80 °C) until complete dissolution. RuCl_3_·*x*H_2_O (94.1 mg, 0.373 mmol) was then added, and the reaction mixture was refluxed (80 °C) overnight. Upon completion, the reaction was allowed to cool to room temperature and filtered under vacuum. The solid was washed successively with EtOH, water and diethyl ether (3 × 20 mL each), affording [Ru­(L)­(Cl)_3_] as a brown solid (148.1 mg, 65% yield). [Ru­(L)­(Cl)_3_] (148.1 mg, 0.243 mmol) was dissolved in a deoxygenated EtOH/H_2_O 3:1 mixture and the solution was refluxed (80 °C) until complete dissolution. LiCl (103 mg, 2.43 mmol), Et_3_N (101 mL, 0.729 mmol) and 2,2′-bipyridine (42 mg, 0.267 mmol) were then added and the reaction mixture was refluxed (80 °C) overnight under an argon atmosphere. Upon completion, the reaction crude was filtered through an aluminum oxide (Al_2_O_3_) plug and washed with EtOH, and the obtained solution was dried by rotary evaporation. The resulting solid was further purified by chromatography column on aluminum oxide (Al_2_O_3_) using a gradient of a mixture of DCM and MeOH (100:0 to 94:6). The desired purple band (*R*_f(DCM/MeOH95:5)_ = 0.2) was collected and the solvent removed by rotary evaporation. Precipitation with Et_2_O from DCM afforded [Ru­(L)­(bipy)­(Cl)]­(Cl) as a purple solid (54 mg, 32.1% yield).

^1^H NMR (300 MHz, MeOD) δ (ppm): 10.22 (d, *J =* 4.9 Hz, 1H, Bipy 19), 9.04 (s, 2H, Tpy 5), 8.80 (d, *J =* 8.2 Hz, 1H, Bpy 16), 8.61 (d, *J =* 8.1 Hz, 2H, Tpy 4), 8.51 (d, *J =* 8.1 Hz, 1H, Bpy 15), 8.36 (dd, *J =* 7.9, 1.6 Hz, 1H, Bpy 17), 8.04 (ddd, *J =* 7.4, 5.7, 1.4 Hz, 1H, Bpy 18), 7.97 (dd, *J =* 7.9, 1.5 Hz, 2H, Tpy 3), 7.75 (ddd, *J =* 8.1, 7.8, 1.5 Hz, 1H, Bpy 14), 7.72 (dd, *J =* 5.6, 1.4 Hz, 2H, Tpy 1), 7.38 (ddd, *J =* 7.5, 5.6, 1.3 Hz, 2H, Tpy 2), 7.33 (dd, *J =* 5.9, 1.5 Hz, 1H, Bpy 12), 7.03 (ddd, *J =* 7.3, 5.8, 1.4 Hz, 1H, Bpy 13), 3.73 – 3.67 (m, 6H, PEG 7–9), (m, 2H, PEG 6). ^13^C­{^1^H}­APT NMR (75 MHz, MeOD) δ (ppm): 166.8 (Tpy C=O d), 160.0 (Bipy f), 159.9 (Tpy a,b), 157.4 (Bipy e), 153.6 (Bipy 19), 153.3 (Tpy 1), 152.8 (Bipy 12), 140.6 (Tpy c), 138.6 (Tpy 3), 138.4 (Bipy 17), 137.2 (Bipy 14), 128.8 (Tpy 2), 128.2 (Tpy 18), 127.5 (Bipy 13), 125.1 (Tpy 4), 124.8 (Bipy 16), 124.7 (Bipy 15), 121.5 (Tpy 5), 73.5 (PEG 6), 70.4 (PEG 7–9), 62.3 (PEG 7–9), 41.6 (PEG 7–9). HRMS (ESI^+^): *m*/*z* [M]^+^ calc for [C_30_H_28_N_6_O_3_Ru]^+^ 657.0950; found 657.0953. Anal. Calc. for C_30_H_28_Cl_2_N_6_O_3_Ru (%): C, 52.03; H, 4.08; N, 12.13; found: C, 52.42; H, 3.99; N, 12.43.

#### NADH Photooxidation

Catalysts **1** and **2** (catalyst loading = 10, 5, 0.5, and 0.05 mol %) were mixed with NADH (200 μM) in PBS. The catalytic reactions were monitored by UV–vis in the dark and under 470, 525, or 600 nm light irradiation. Characteristic band of NADH = 340 nm.

#### GSH Photooxidation

Catalyst **1** and **2** (20 μM; 5 mol %) were mixed with GSH (400 μM) in 7 mL PBS. At different irradiation times (at 470 nm, 525 or 600 nm), 250 μL of the catalytic reaction solution was added into 2250 μL PBS, and then 10 μL of DTNB (5,5′-dithiobis­(2-nitrobenzoic acid)) (10 mg/mL) were added. The absorbance of this solution was measured by UV–vis spectroscopy at different times, monitoring the band at 412 nm.

#### Cell Culture

The human breast cancer MCF7, breast cancer triple negative MDA-MB-231, lung carcinoma A549 and NHDF-Ad fibroblasts cell lines were cultured in high glucose Dulbecco’s modified Eagle’s medium (DMEM) supplemented with 5% fetal bovine serum (FBS) (20% for fibroblasts) as well as 100 μg/mL penicillin/streptomycin at 37 °C under a humidified atmosphere of 95% air and 5% CO_2_.

#### Cellular Uptake

Cellular uptake was determined by measuring the ruthenium content inside the cells with inductively coupled plasma optical emission spectroscopy (ICP-OES). Measurements were recorded using a Thermo Scientific i CAP PRO XP Duo spectrometer. Samples were prepared as follows: MCF7 cells were seeded in 75 cm^2^ cell culture flasks (three flasks per condition) and incubated until approximately 90% confluence was reached. Before adding the compounds, the culture medium was removed and then 10 mL of culture medium containing 100 μM of **1** and **2** were added. For the control, 10 mL of compound-free culture medium were added. Cells were incubated with each complex (**1** or **2**) (100 μM) or without complex (control) for 24 h at 37 °C. Subsequently, cells were detached with 3 mL of trypsin and incubated for 10 min, after which 7 mL of culture medium were added. Cells were collected and counted, obtaining cell densities of approximately 1 × 10^6^ cells mL^–1^. Samples were centrifuged, the supernatant was removed, and the pellets were washed once with PBS. The samples were then digested for 24 h with 1 mL of concentrated nitric acid. Once digested, 9 mL of ultrapure water were added, and ICP-OES measurements were performed in triplicate for each of the samples.

#### Cytotoxicity and Photocytotoxicity Assay

The MTT assay was used to determine cell viability as an indicator for cells sensitivity to the complexes. Exponentially growing cells MCF-7, MDA-MB-231, A549 and NHDF-Ad, were seeded at a density of approximately 7 × 10^3^ cells per well in 96-well flat-bottomed microplates and incubated for 24 h. The complexes were dissolved in H_2_O and then diluted with culture medium to obtain the final concentrations ranging from 200 μM to 0.4 μM in quadruplicate. Cells were incubated with compounds for 48 h at 37 °C. After 24 h of incubation with the complexes, duplicates of each concentration were irradiated with a LED lamp of 470 nm with a light dose of 15.1 J/cm^2^ for 10 min, 525 nm with a light dose of 45.3 J/cm^2^ for 30 min and 600 nm with a light dose of 58.5 J/cm^2^ for 60 min. The cells were cultured for a total of 48 h. Ten μL of MTT (5 mg/mL) were added to each well and plates were incubated for 2 h at 37 °C. Then, medium was discarded and DMSO (100 μL per well) was added to dissolve the formazan precipitates in the plates. A 96-well FLUOstar Omega (BMG LABTECH) was used to determine the optical density at 550 nm, and the IC_50_ was calculated. Each experiment was repeated in at least three independent analyses.

#### NAD^+^/NADH and GSH/GSSG Assays

Intracellular NAD^+^/NADH quantities were measured using a NAD^+^/NADH assay kit (HY-K0313, MedChemExpress, NJ, USA) following the manufactureŕs instructions. Intracellular GSSG/GSH quantities were measured using a GSH/GSSG assay kit (HY-K0311, MedChemExpress, NJ, USA) following the manufactureŕs instructions.

#### Flow Cytometry Studies: ROS and Mitochondrial Membrane Potential

200,000 cells (MCF7/mL) were seeded in flat-bottom 12-well plates (1 mL/well) in complete medium and allowed to attach for 24 h. A solution of the complexes **1** and **2** were added at concentration of 200 μM and MCF7 cells were cultured for a total of 48 h. A duplicate was irradiated with a 600 nm LED at a dose of 58.5 J/cm^2^ for 60 min after 24 h of incubation and left to incubate for another 24 h. The cells were then deadhered with 200 μL of trypsin and resuspended in 1 mL of media. This cell suspension has been used for all flow cytometry measurements that have been carried out at cytometer SA3800 Sony (cell cycle) and cytometer GALLIOS Beckman Coulter. Commercial kits were used for all flow cytometry studies and in all cases, they were used as indicated in the manufacturer’s instructions. ROS production: CellROX Green and CellROX Orange Flow Cytometry Assay Kits (molecular probes by life technologies, catalogue number C10492). Mitochondrial potential: MitoStep Flow Cytometry Mitochondrial Membrane Potential Assay (immunostep, reference: MITO-100T).

## Supplementary Material



## Notes

Additional experimental details including materials and methods, synthesis and characterization, NMR spectra, HRMS (ESI+) spectra, stability and photostability, UV–vis spectra, optical properties, Stern-volmer emission quenching studies, photocatalysis, DNA binding studies, NADH photooxidation, GSH photooxidation and biological studies (PDF)
