# Supplementary material for: Red-Light-Driven Ru(II) Photocatalysts Enable Dual Oxidation of NADH and Glutathione in Living Cells
Source: Inorg Chem. 2026 Oct 27;65(44):25912–28. doi: 10.1021/acs.inorgchem.6c03885 (PMC13753733; doi:10.1021/acs.inorgchem.6c03885)
Supplement: Supplementary file 1 [file ic6c03885_si_001.pdf]

Electronic Supplementary Information

Red-Light-Driven Ru(II) Photocatalysts Enable Dual Oxidation of NADH and Glutathione in Living Cells

Mikel Gabino-García, María Gil-Moles,* and M. Concepción Gimeno*
Departamento de Química Inorgánica. Instituto de Síntesis Química y Catálisis Homogénea (ISQCH), CSIC-Universidad de Zaragoza, C. Pedro Cerbuna 12, 50009 Zaragoza, Spain.
E-mail: gimeno@unizar.es, m.gilmoles@unizar.es

Table of contents

1. Materials and Methods: S3
   1.1. Synthesis and characterization: S3
   1.2. NMR spectra: ^{1}H NMR, ^{13}C{^{1}H}-APT NMR, ^{19}F{^{1}H} NMR, COSY, HSQC and HMBC S5
   1.3. HRMS (ESI+) spectra S14
   1.4. FT/IR SPECTRA S15
2. Stability and photostability S16
   2.1. UV-Vis spectra S16
   2.2. ^{1}H NMR spectra S18
      2.2.1. Hydrolysis of complex 2: rate and equilibrium constant calculation S19
3. Optical properties S21
4. Electrochemical properties S26
   4.1. Cyclic voltammetry S26
   4.2. Excited state redox potential determination S27
   4.3. Determination of free energy for the photoinduced oxidation (ΔG) S27
   4.4. Determination of the HOMO-LUMO energy gap S28
5. Photocatalysis S28
   5.1. Stern-volmer: emission quenching S24
   5.2. NADH photooxidation S28
      5.2.1. Kinetic study S28
      5.2.2. Mechanistic studies S36
   5.3. GSH photooxidation S48
      5.3.1. Kinetic study S48
      5.3.2. Mechanistic studies S53
6. Biological studies S56

6.1. DNA Binding S56
6.1.1. UV-vis S56
6.1.2. Competitive ethidium bromide (EB) fluorescence displacement assay S57
6.2. Lipophilicity assay S59
6.3. Cell culture S59
6.4. Cellular uptake S59
6.5. Cytotoxicity and photocytotoxicity assay S60
6.6. NAD^+/NADH assay S60
6.7. GSH/GSSG assay S61
6.8. Flow cytometry studies: ROS and mitochondrial membrane potential S63
7. REFERENCES S63

1. Materials and Methods:

Instrumentation. NMR spectra were recorded on Bruker ARX300, AV300 or AV400 spectrometers. ^{1}H NMR spectra were recorded at 300 or 400 MHz; $^{13}C\{^{1}H\}$-APT NMR spectra were recorded at 75 or 101 MHz; $^{19}F\{^{1}H\}$ NMR spectra were recorded at 282 MHz. A Bruker MicroToF-Q spectrometer was used for High-resolution mass spectra-ESI (HRMS-ESI) equipped with an API-ESI source and a QToF mass analyzer, both allowing a maximum error in the measurement of 5 ppm. FT/IR spectra were recorded on a Jasco FT/IR-6600 spectrometer. Materials: The starting materials used in this work have been purchased from Sigma Aldrich or BLD and used as received.

1.1. Synthesis and characterization:

Synthesis of L: 2,2':6',2''-terpyridine-4'-carboxylic acid (300 mg, 1.08 mmol) and DIPEA (N,N-Diisopropylethylamine) (0.47 mL, 2.7 mmol) were dissolved in dry DMF (5 mL). To this solution HATU (Hexafluorophosphate Azabenzotriazole Tetramethyl Uronium) (616 mg, 1.62 mmol) was added and was left to stir under argon atmosphere for 30 min. To the resulting brown suspension, a 2 M solution of propargyl-PEG2-amine (172 µL, 1.2 mmol) in dry methanol was added dropwise and the resulting orange solution was left to stir overnight at room temperature and under argon atmosphere. Then, the reaction mixture was quenched with water (150 mL) and was left to stand for 15 min to allow the fine precipitate to aggregate and settle, facilitating subsequent filtration. The solid was washed with water (10 mL x 3) and with a N-pentane:ether (5:1) mixture (70 mL). The obtained product was a brown and moist solid, which was dried under vacuum for 24 h. The crude product was purified by column chromatography on aluminum oxide (Al_2O_3) using a gradient of DCM and MeOH (from 100:0 to 98:2) and the progress was monitored by TLC ($R_{f\ (DCM:MeOH\ 99:1)}$ = 0.5). The desired fraction was collected, and the solvent was removed under vacuum to afford **L** as a white solid (250.7 mg, 57.7% yield).

^{1}H NMR (400 MHz, MeOD) δ (ppm): 8.79 (s, 2H, Tpy 5), 8.72 (ddd, J = 4.8, 1.8, 0.9 Hz, 2H, Tpy 1), 8.65 (dt_{pseudo}, J = 8.0, 1.1 Hz, 2H, Tpy 4), 8.01 (td_{pseudo}, J = 7.8, 1.8 Hz, 2H, Tpy 3), 7.50 (ddd, J = 7.5, 4.8, 1.2 Hz, 2H, Tpy 2), 4.23 (d, J = 2.4 Hz, 2H, PEG 10), 3.79 – 3.71 (m, 6H, PEG 7-9), 3.71 – 3.64 (m, 2H, PEG 6), 2.83 (t, J = 2.4 Hz, 1H, alkyne 11). $^{13}C\{^{1}H\}$-APT NMR (101 MHz, MeOD) δ (ppm): 168.5 (C=O d), 157.7 (Tpy a/b) 156.7 (Tpy a/b), 150.3 (Tpy 1), 145.6 (Tpy c), 138.7 (Tpy 3), 125.7 (Tpy 2), 122.7 (Tpy 4), 119.7 (Tpy 5), 76.0 (alkyne e), 71.1, 70.4, 70.1 (PEG 7,8,9), 59.1 (PEG 10), 41.1 (PEG 6). HRMS (ESI^+): m/z $[M+H]^+$ calc. for $[C_{23}H_{23}N_4O_3]^+$ 403.1765; found 403.1767. m/z $[M+Na]^+$ calc. for $[C_{23}H_{22}N_4NaO_3]^+$ 425.1584; found 425.1586. Anal. Calcd. for $C_{23}H_{22}N_4O_3$ (%): C, 68.64; H, 5.51; N, 13.92; Found: C, 68.14; H, 5.56; N, 13.68.

Synthesis of complex 1: L (177 mg, 0.44 mmol) was dissolved in deoxygenated dry EtOH (20 mL), the solution was heated to reflux (80 °C) and left to stir under argon atmosphere until complete dissolution. Then $RuCl_3 \cdot x\ H_2O$ (55.6 mg, 0.22 mmol) and 5 drops of N-ethylmorpholine were added to the solution and the reaction was refluxed (80 °C) overnight under an argon atmosphere. Subsequently, the reaction mixture was cooled to room temperature, and the solvent was removed under vacuo. The resulting product was purified by silica column chromatography. The column was first eluted with EtOH to remove an initial

yellow band, which was discarded. Then, the eluent was switched to MeCN:H_2O:saturated $NaNO_3$ (aq) (20:3:1) and the desired fraction was eluted as a red band. The obtained fraction was evaporated to dryness, and the resulting red solid was dissolved in DCM and filtered through a pad of Celite®. The filtrate was then dried over anhydrous $MgSO_4$, filtered and concentrated under reduced pressure. Precipitation with ether afforded $[Ru(L)_2](Cl)_2$ as a red solid (121.3 mg; 56.5% yield). $[Ru(L)_2](Cl)_2$ (120.5 mg, 0.123 mmol) and Ag(OTf) (63.4 mg, 0.246 mmol) were dissolved in DCM (15 mL) and the reaction mixture was stirred at room temperature for 3 hours in the absence of light. Upon completion, the crude was filtered through a Celite® pad and washed with MeOH. The filtrate was then evaporated to dryness under reduced pressure, and the resulting solid was recovered by adding ether and scraping it from the flask walls, as it adhered strongly upon drying. The obtained solid was then dried under vacuum for several days to afford $[Ru(L)_2](OTf)_2$ as a crystalline red solid (106.7 mg; 71.8% yield).

^1H NMR (300 MHz, MeOD) δ (ppm): 9.35 (s, 4H, Tpy 5), 8.77 (d, J = 8.2 Hz, 4H, Tpy 4), 8.03 (tt_{pseudo}, J = 7.9, 1.4 Hz, 4H, Tpy 3), 7.50 (d, J = 5.5 Hz, 4H, Tpy 1), 7.28 (ddd, J = 7.3, 5.6, 1.3 Hz, 4H, Tpy 2), 4.24 (d, J = 2.4 Hz, 2H, PEG 10), 3.95 – 3.75 (m, 14H, PEG 7-9), 3.73 – 3.67 (m, 2H, PEG 6), 2.87 (t, J = 2.4 Hz, 1H, alkyne 11). $^{13}C\{^1H\}$-APT NMR (75 MHz, MeOD) δ (ppm): 166.3 (Tpy C=O d), 166.3 (Tpy C=O d'), 159.1 (Tpy a), 156.9 (Tpy b), 153.4 (Tpy 1), 143.0 (Tpy c), 142.9 (Tpy c'), 139.7 (Tpy 3), 129.2 (Tpy 2), 126.1 (Tpy 4), 122.9 (Tpy 5), 122.9 (Tpy 5'), 76.1 (PEG alkyne 11), 73.6 (PEG 6), 71.2 (PEG 7-9), 70.4 (PEG 7'-9'), 70.4 (PEG 7-9), 70.3 (PEG e), 62,3 (PEG 7-9), 59.2 (PEG 10), 49.0 (q, MeOD), 41.7 (PEG 7-9), 41.6 (PEG 7'-9'). $^{19}F\{^1H\}$ NMR (282 MHz, MeOD) δ (ppm): -80.1. HRMS (ESI^+): m/z $[M]^{2+}$ calc for $[C_{46}H_{44}N_8O_6Ru]^{2+}$ 453.1208; found 453.1221. Anal. Calcd. for $C_{48}H_{44}F_6N_8O_{12}RuS_2$ (%): C, 47.88; H, 3.68; N, 9.31; S, 5.33; found: C, 47.40; H, 4.18; N, 9.51; S, 5.03.

Synthesis of complex 2: **L** (150 mg, 0.373 mmol) was dissolved in EtOH (8 mL) and the mixture was refluxed (80 ˚C) until complete dissolution. $RuCl_3 \cdot x\ H_2O$ (94.1 mg, 0.373 mmol) was then added, and the reaction mixture was refluxed (80 ˚C) overnight. Upon completion, the reaction was allowed to cool to room temperature and filtered under vacuum. The solid was washed successively with EtOH, water and diethyl ether (3 x 20 mL each), affording $[Ru(L)(Cl)_3]$ as a brown solid (148.1 mg, 65% yield). $[Ru(L)(Cl)_3]$ (148.1 mg, 0.243 mmol) was dissolved in a deoxygenated EtOH:H_2O 3:1 mixture and the solution was refluxed (80 ˚C) until complete dissolution. LiCl (103 mg, 2.43 mmol, 10 eq.), Et_3N (101 mL, 0.729 mmol) and 2,2'-bipyridine (42 mg, 0.267 mmol) were then added and the reaction mixture was refluxed (80 ˚C) overnight under an argon atmosphere. Upon completion, the reaction crude was filtered through an aluminum oxide (Al_2O_3) plug and washed with EtOH, and the obtained solution was dried by rotary evaporation. The resulting solid was further purified by chromatography column on aluminum oxide (Al_2O_3) using a gradient of a mixture of DCM and MeOH (100:0 to 94:6). The desired purple band ($Rf_{DCM:MeOH\ 95:5}$ = 0.2) was collected and the solvent removed by rotary evaporation. Precipitation with Et_2O from DCM afforded [Ru(L)(bipy)(Cl)](Cl) as a purple solid (54 mg, 32.1% yield).

^1H NMR (300 MHz, MeOD) δ (ppm): 10.22 (d, J = 4.9 Hz, 1H, Bipy 19), 9.04 (s, 2H, Tpy 5), 8.80 (d, J = 8.2 Hz, 1H, Bpy 16), 8.61 (d, J = 8.1 Hz, 2H, Tpy 4), 8.51 (d, J = 8.1 Hz, 1H,

Bpy 15), 8.36 (dd, $J = 7.9$, 1.6 Hz, 1H, Bpy 17), 8.04 (ddd, $J = 7.4$, 5.7, 1.4 Hz, 1H, Bpy 18), 7.97 (dd, $J = 7.9$, 1.5 Hz, 2H, Tpy 3), 7.75 (ddd, $J = 8.1$, 7.8, 1.5 Hz, 1H, Bpy 14), 7.72 (dd, $J = 5.6$, 1.4 Hz, 2H, Tpy 1), 7.38 (ddd, $J = 7.5$, 5.6, 1.3 Hz, 2H, Tpy 2), 7.33 (dd, $J = 5.9$, 1.5 Hz, 1H, Bpy 12), 7.03 (ddd, $J = 7.3$, 5.8, 1.4 Hz, 1H, Bpy 13), 3.73 – 3.67 (m, 6H, PEG 7-9), (m, 2H, PEG 6). $^{13}C\{^1H\}$APT NMR (75 MHz, MeOD) δ (ppm): 166.8 (Tpy C=O d), 160.0 (Bipy f), 159.9 (Tpy a,b), 157.4 (Bipy e), 153.6 (Bipy 19), 153.3 (Tpy 1), 152.8 (Bipy 12), 140.6 (Tpy c), 138.6 (Tpy 3), 138.4 (Bipy 17), 137.2 (Bipy 14), 128.8 (Tpy 2), 128.2 (Tpy 18), 127.5 (Bipy 13), 125.1 (Tpy 4), 124.8 (Bipy 16), 124.7 (Bipy 15), 121.5 (Tpy 5), 73.5 (PEG 6), 70.4 (PEG 7-9), 62.3 (PEG 7-9), 41.6 (PEG 7-9). HRMS (ESI$^+$): m/z [M]$^+$ calc for $[C_{30}H_{28}N_6O_3Ru]^+$ 657.0950; found 657.0953. Anal. Calc. for $C_{30}H_{28}Cl_2N_6O_3Ru$ (%): C, 52.03; H, 4.08; N, 12.13; found: C, 52.42; H, 3.99; N, 12.43.

1.2. NMR spectra: ^{1}H NMR, $^{13}C\{^1H\}$-APT NMR, $^{19}F\{^1H\}$ NMR, COSY, HSQC and HMBC

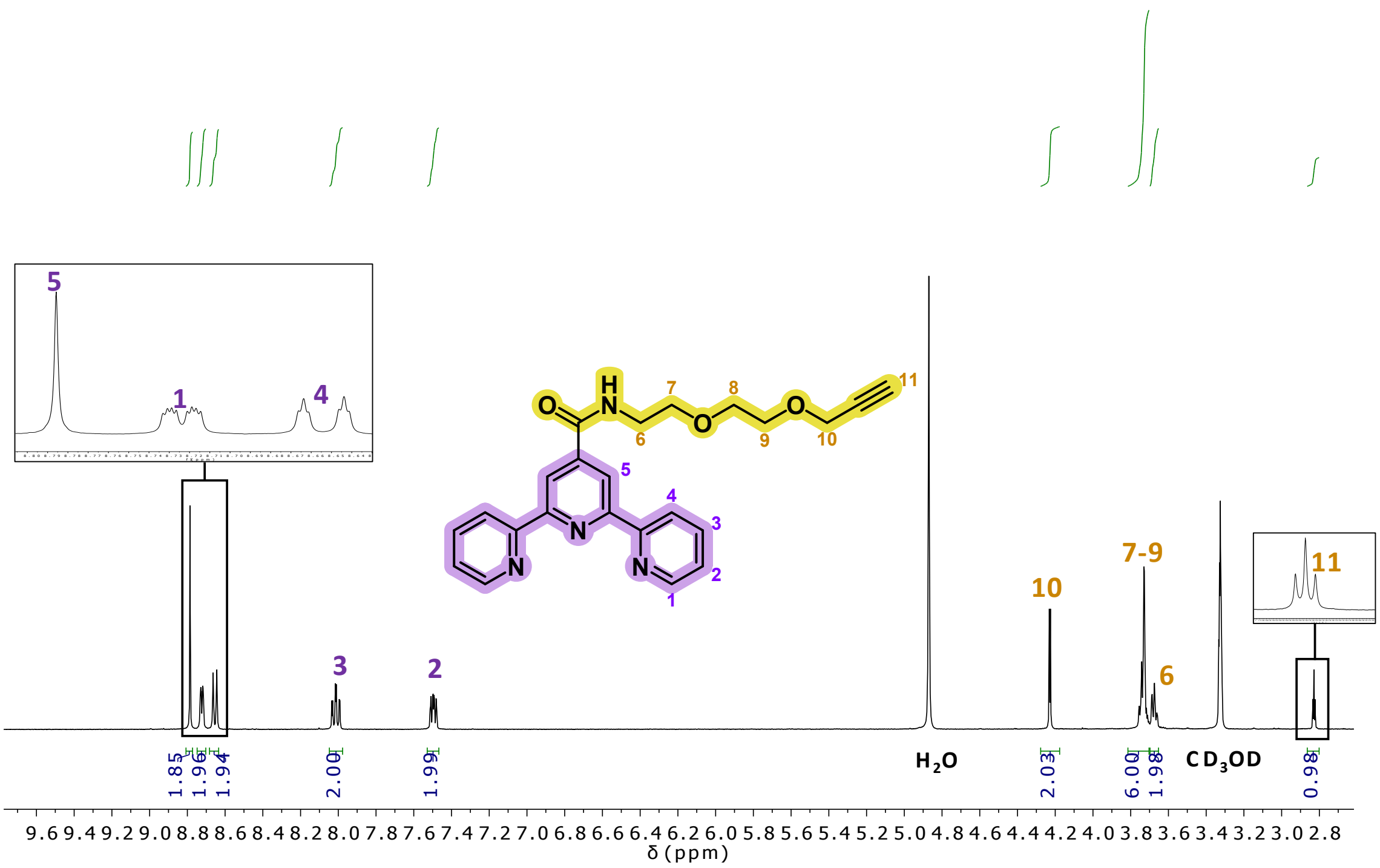

Figure S1. ^{1}H NMR spectrum of **L** (400 MHz, MeOD) δ (ppm).

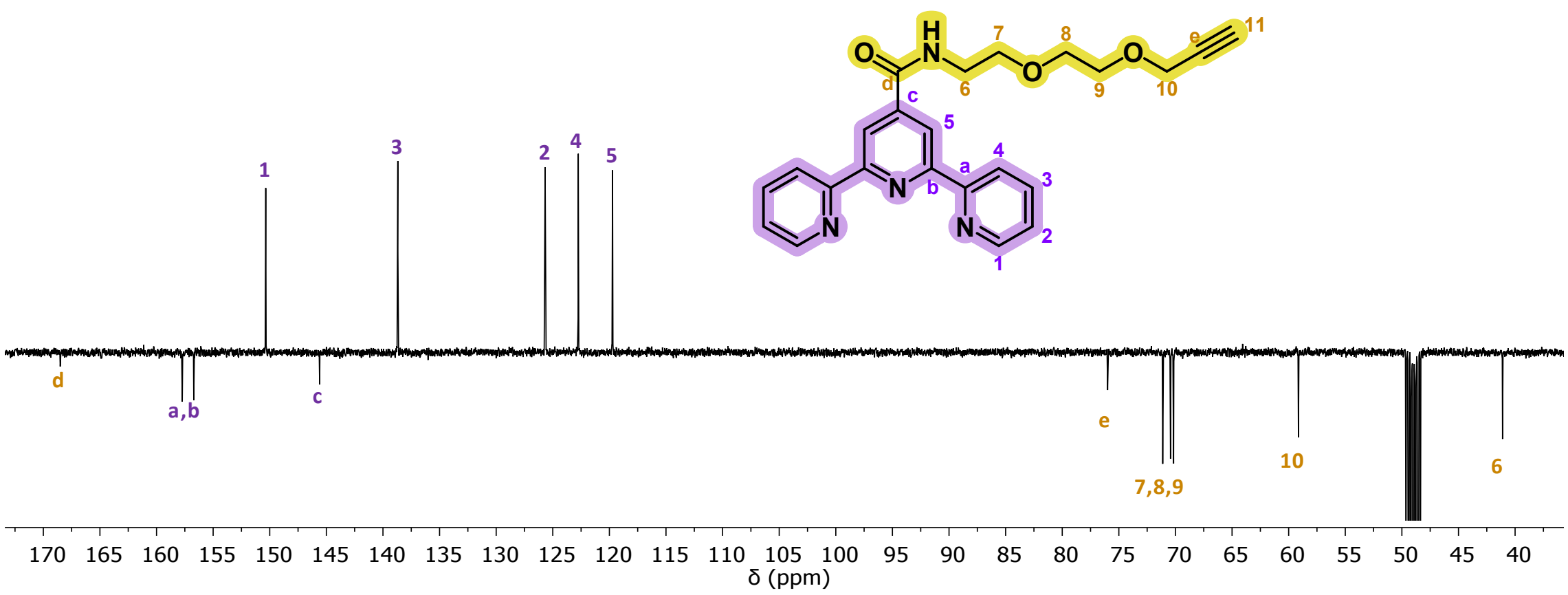

Figure S2. $^{13}C\{^{1}H\}$-APT NMR spectrum of **L** (101 MHz, MeOD) δ (ppm).

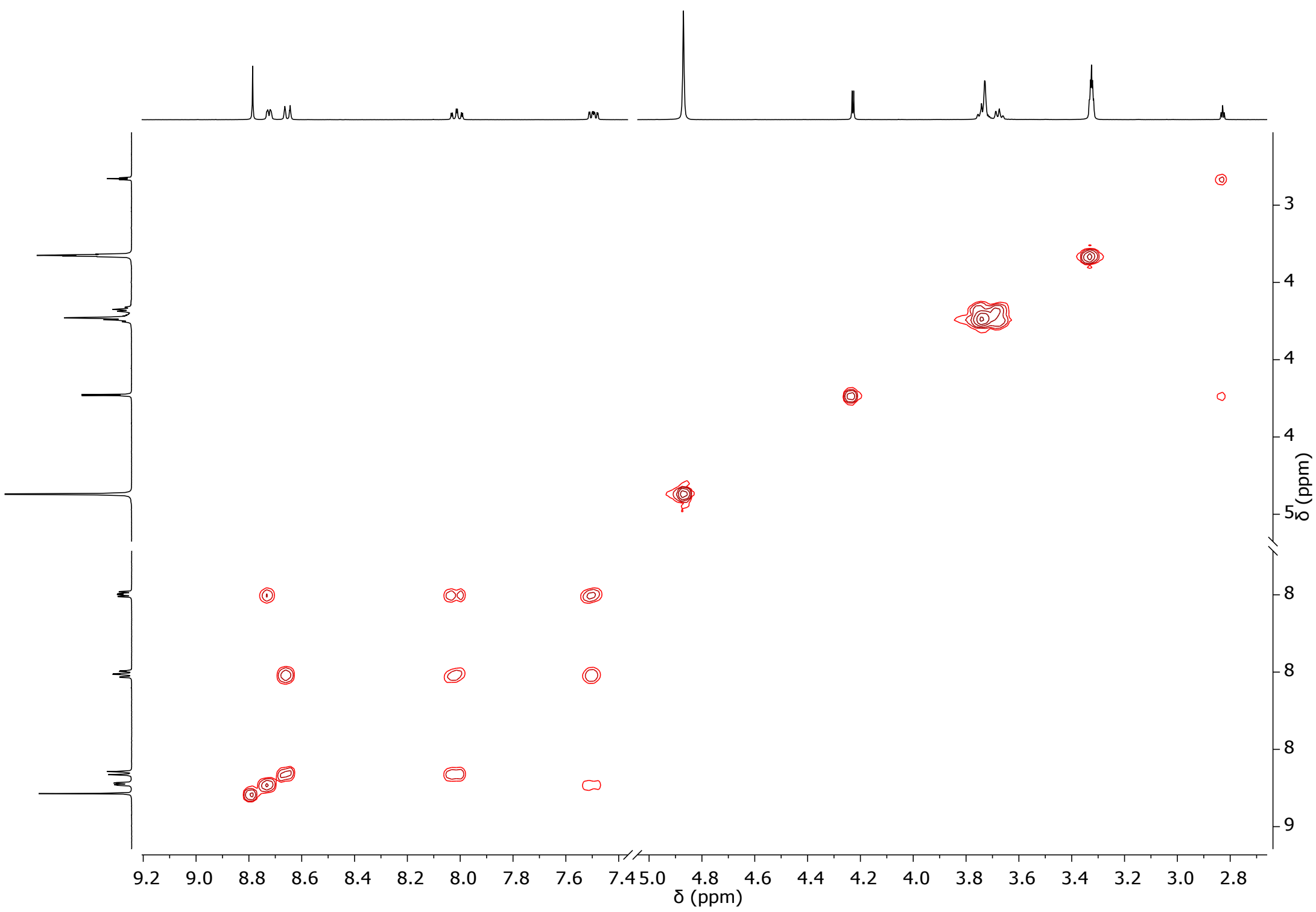

Figure S3. 2D ^{1}H-^{1}H COSY NMR spectrum of **L**.

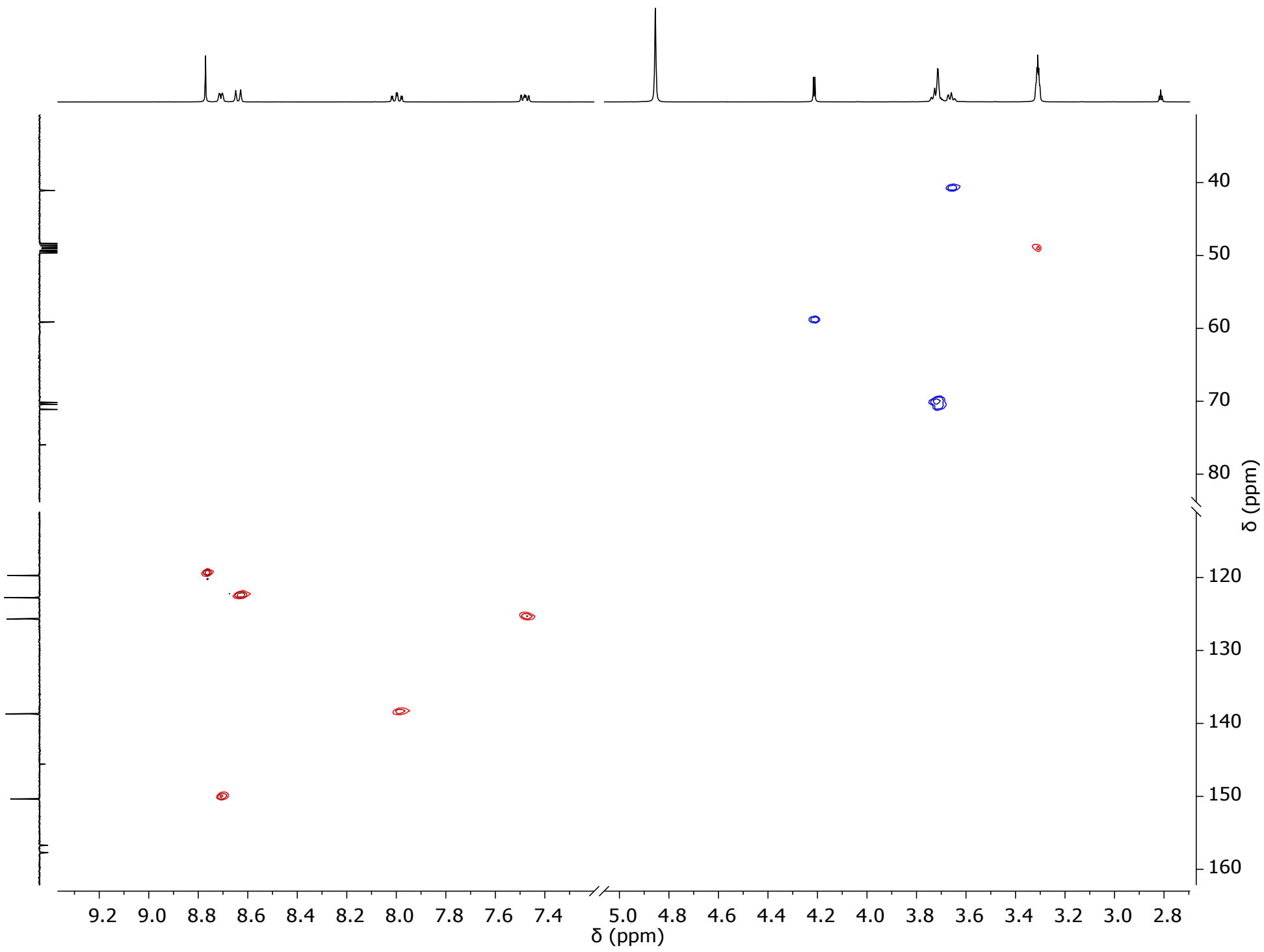

Figure S4. 2D ^{1}H-^{13}C HSQC NMR spectrum of **L**.

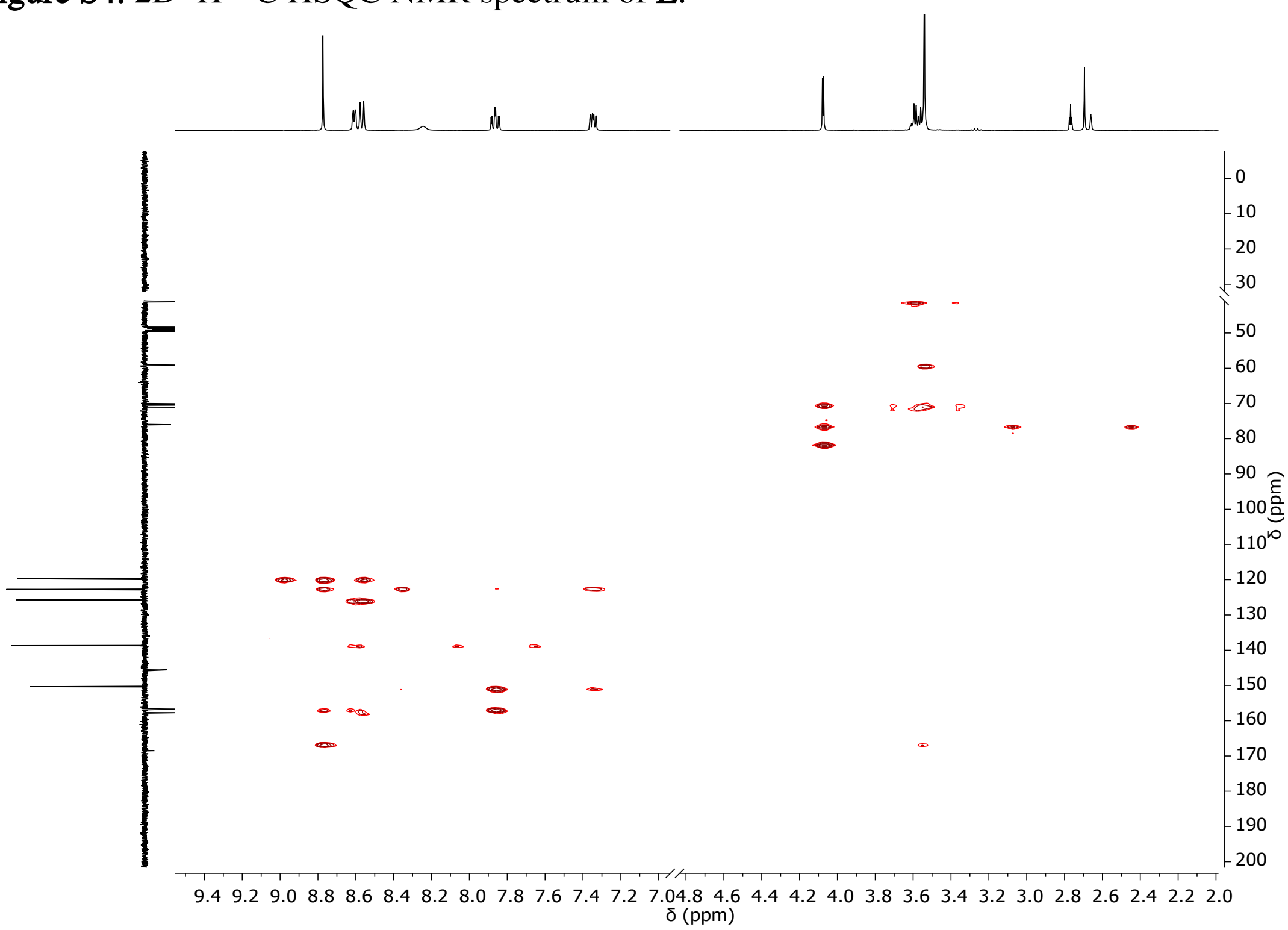

Figure S5. 2D ^{1}H-^{13}C HMBC NMR spectrum of **L**.

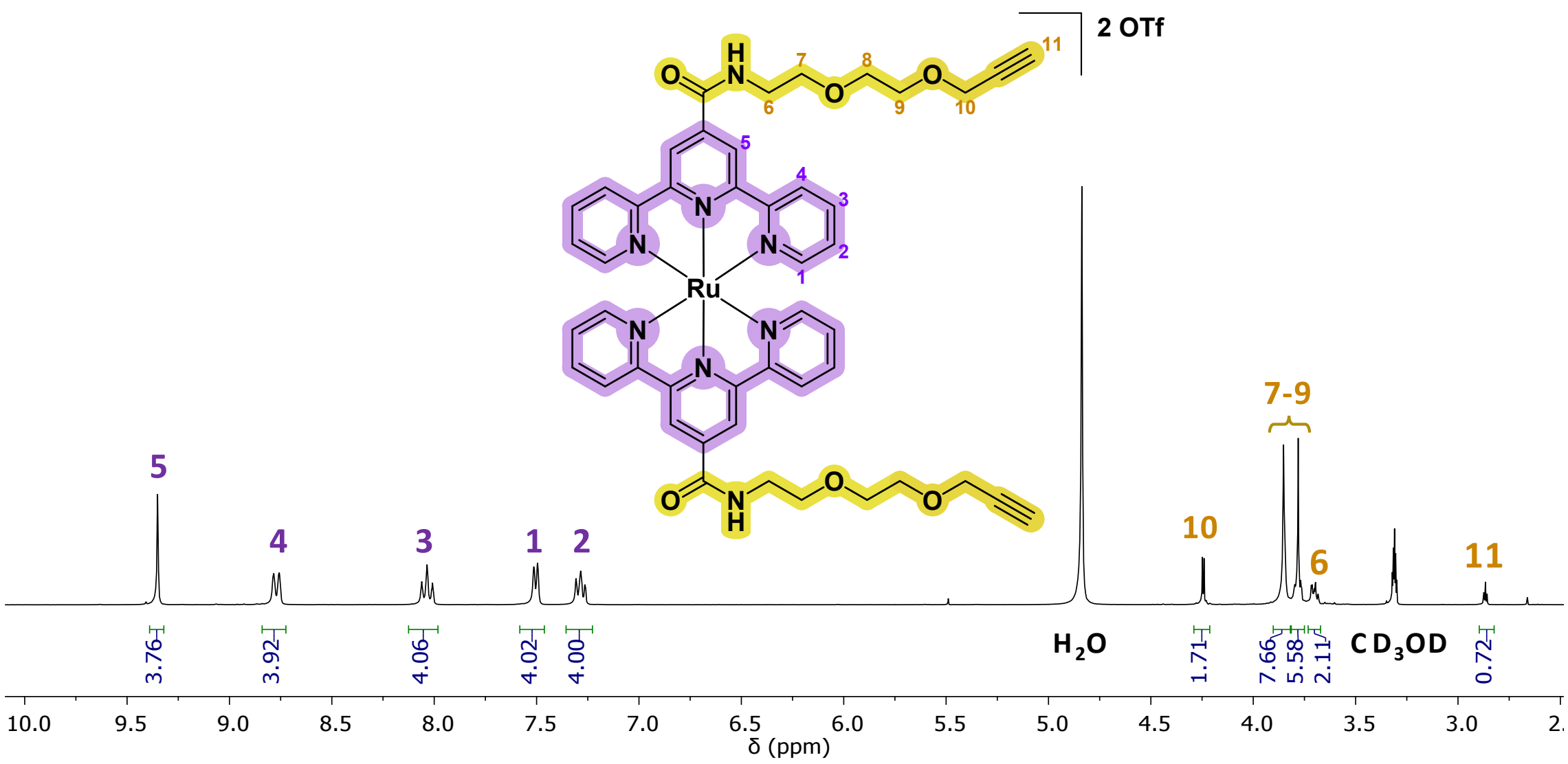

Figure S6. ^{1}H NMR spectrum of complex **1** (300 MHz, MeOD) δ (ppm).

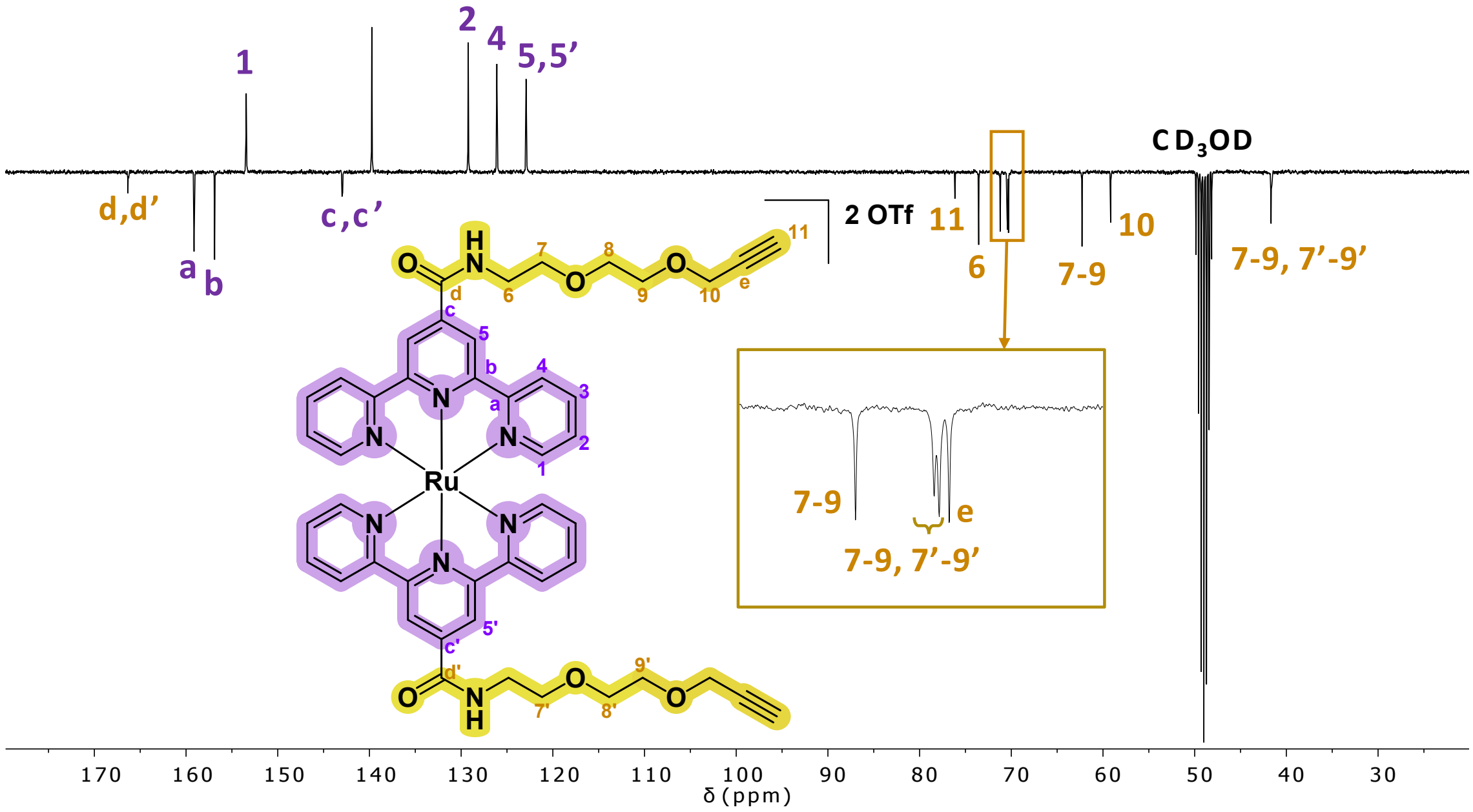

Figure S7. $^{13}C\{^{1}H\}$-APT NMR spectrum of complex **1** (75 MHz, MeOD) δ (ppm).

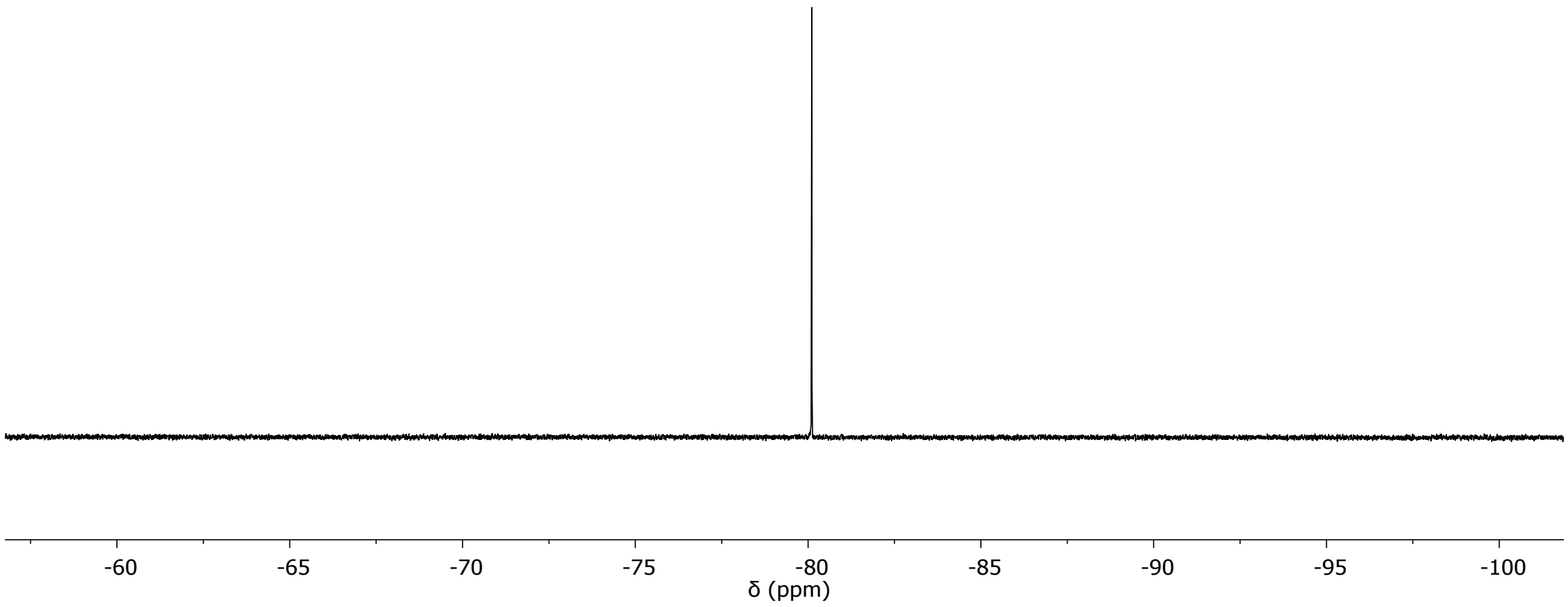

Figure S8. $^{19}F\{^{1}H\}$ NMR spectrum of complex **1** (282 MHz, MeOD) δ (ppm).

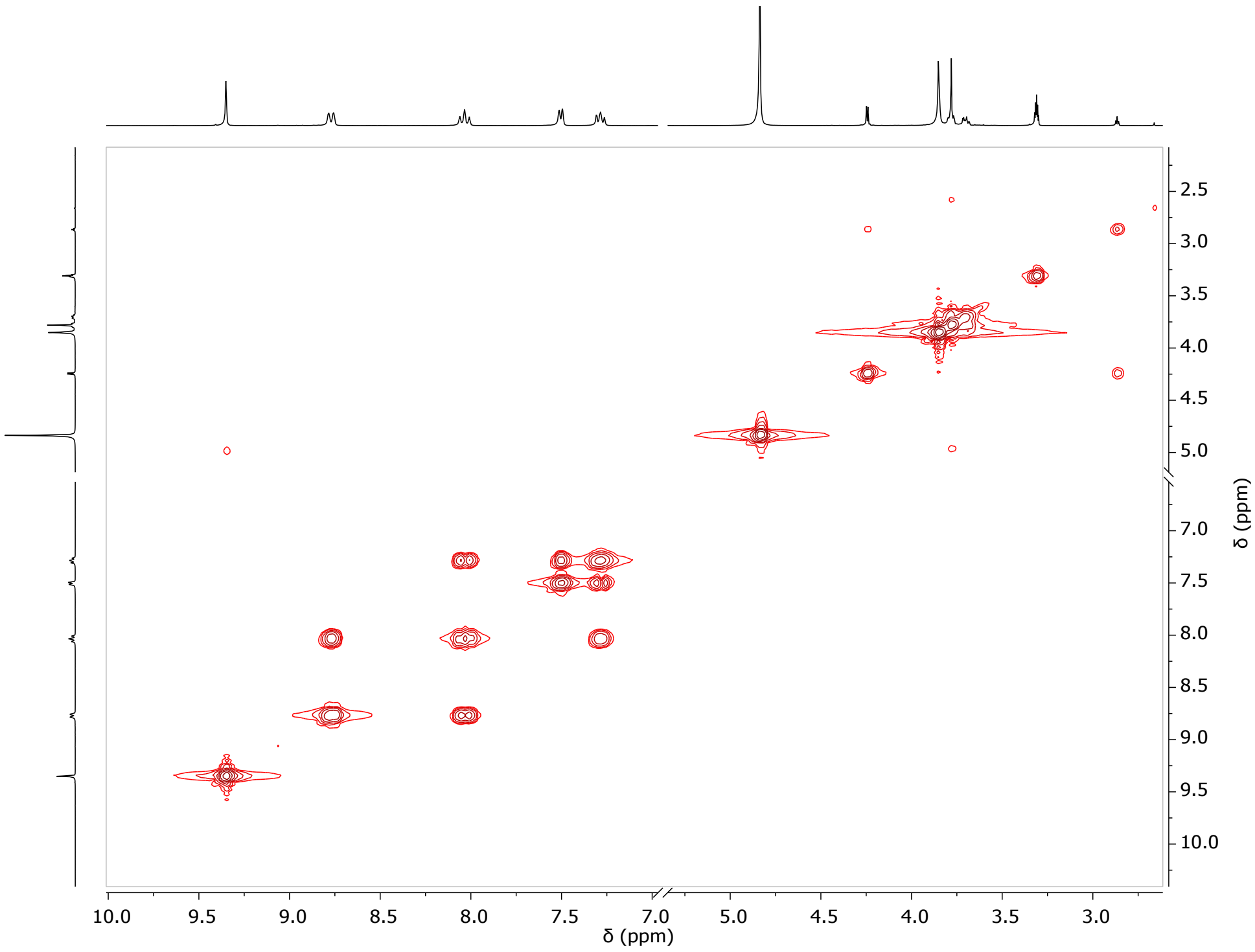

Figure S9. 2D ^{1}H-^{1}H COSY NMR spectrum of complex **1**.

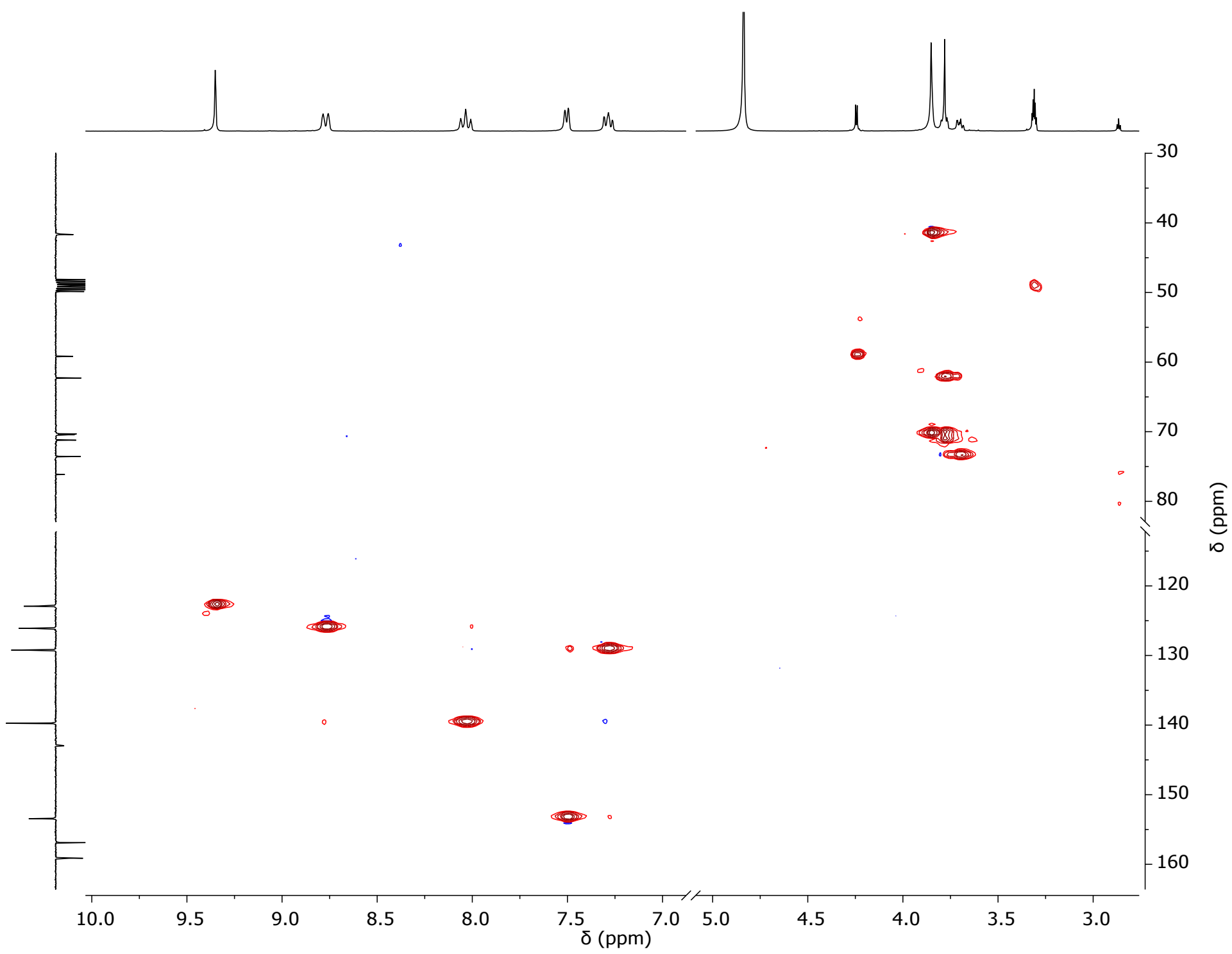

Figure S10. 2D ^{1}H-^{13}C HSQC NMR spectrum of complex **1**.

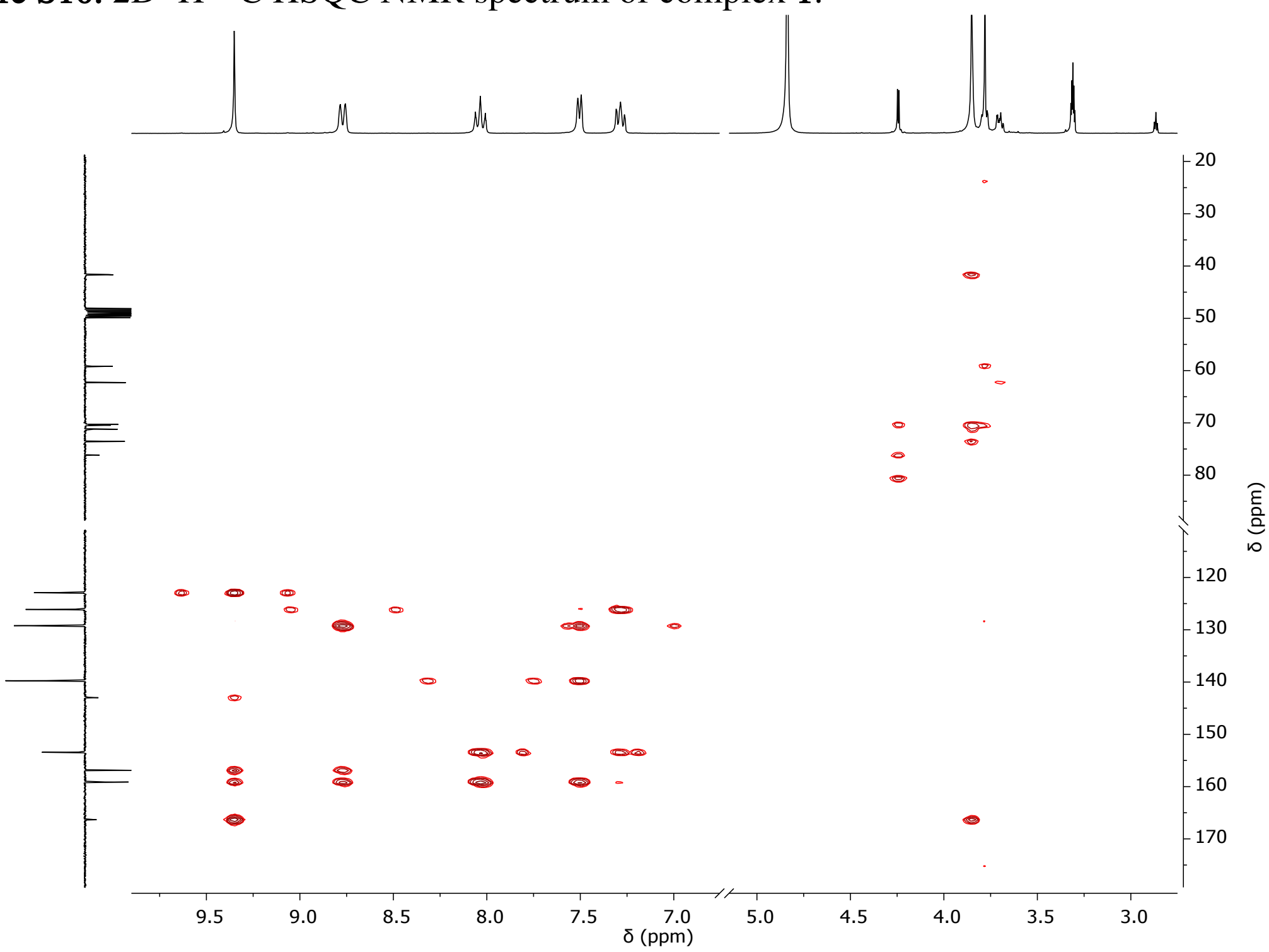

Figure S11. 2D ^{1}H-^{13}C HMBC NMR spectrum of complex **1**.

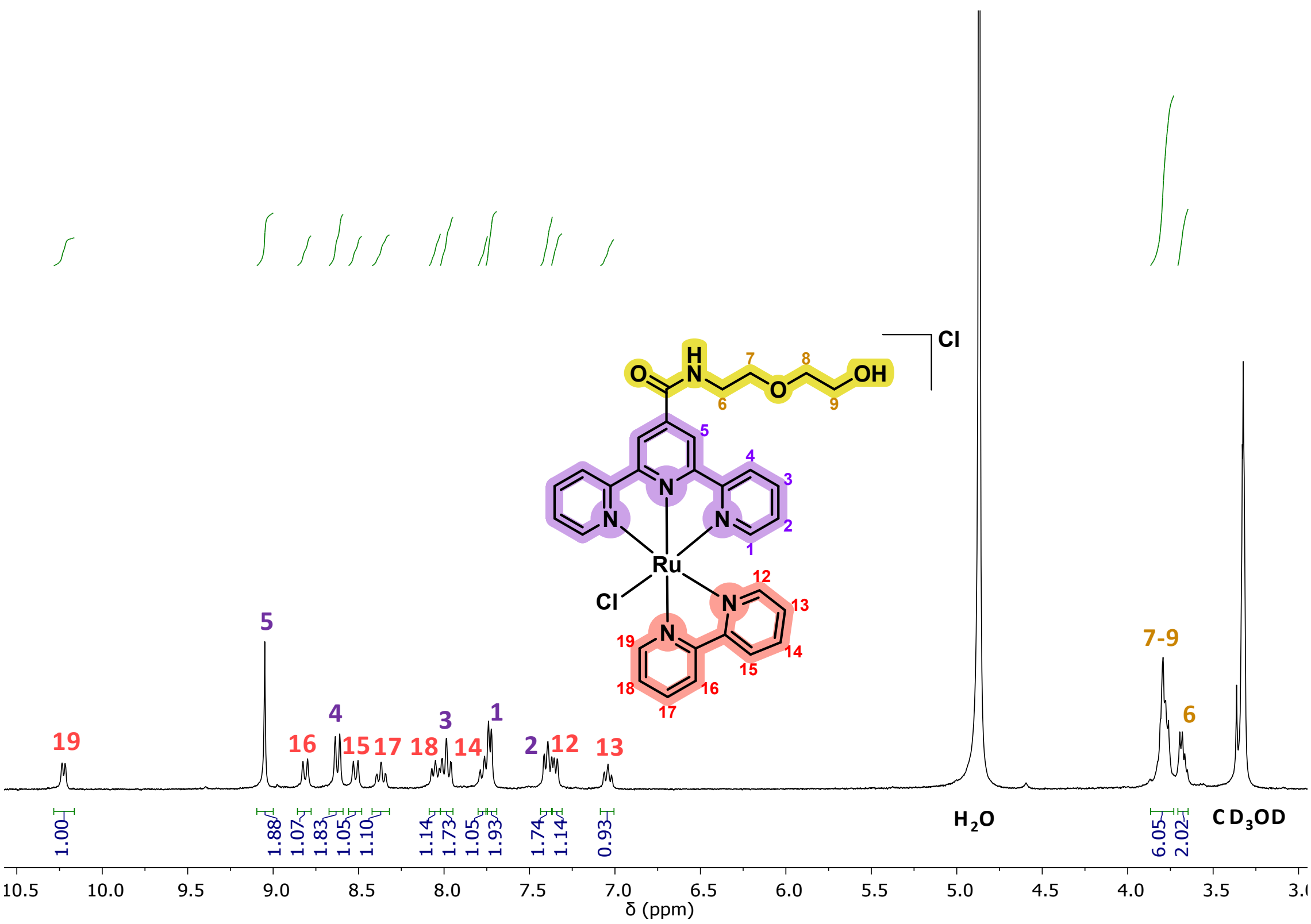

Figure S12. ^{1}H NMR spectrum of complex **2** (300 MHz, MeOD) δ (ppm).

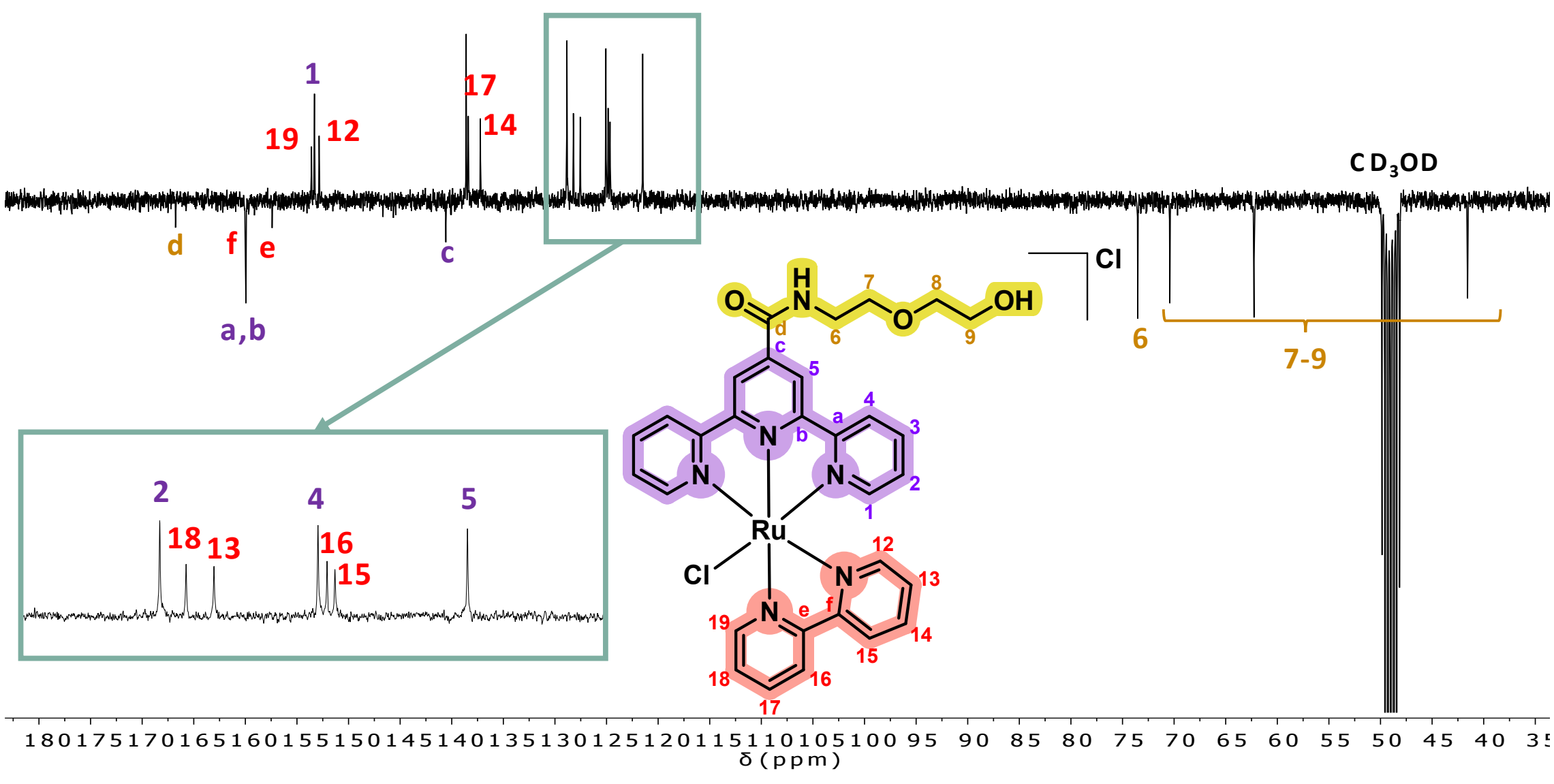

Figure S13. ^{13}C{^{1}H} APT NMR spectrum of complex **2** (75 MHz, MeOD) δ (ppm).

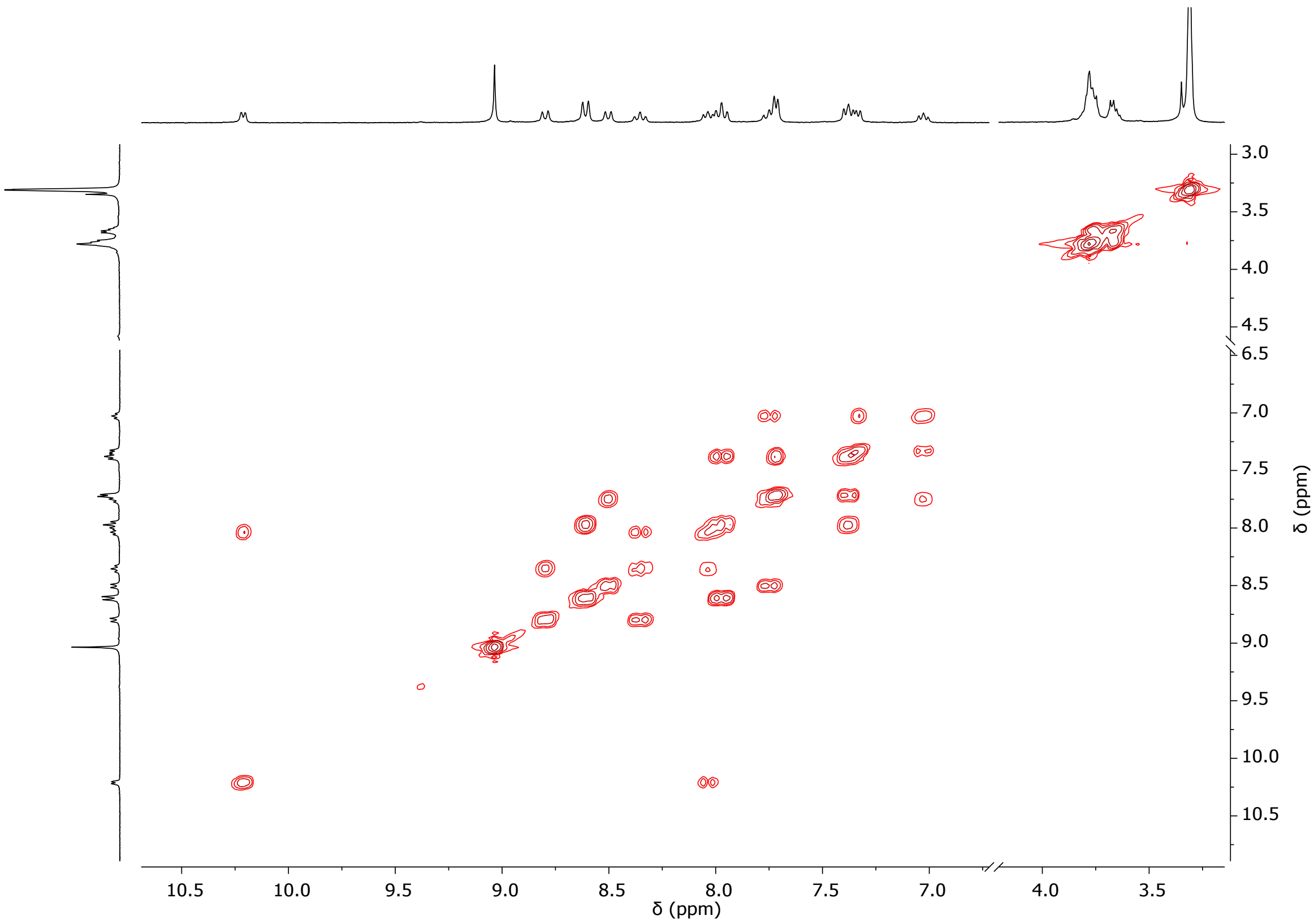

Figure S14. 2D ^{1}H-^{1}H COSY NMR spectrum of complex **2**.

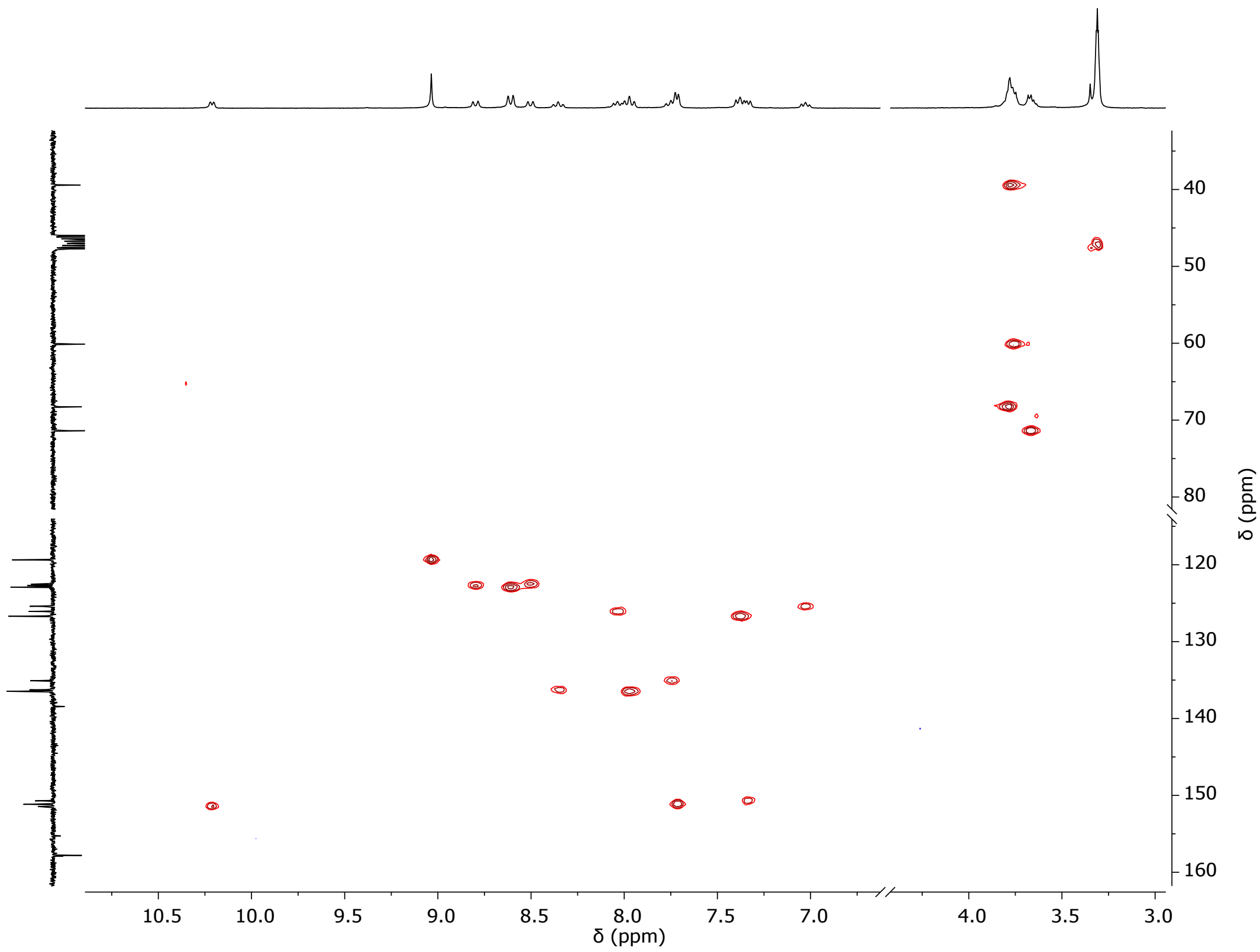

Figure S15. 2D ^{1}H-^{13}C HSQC NMR spectrum of complex **2**.

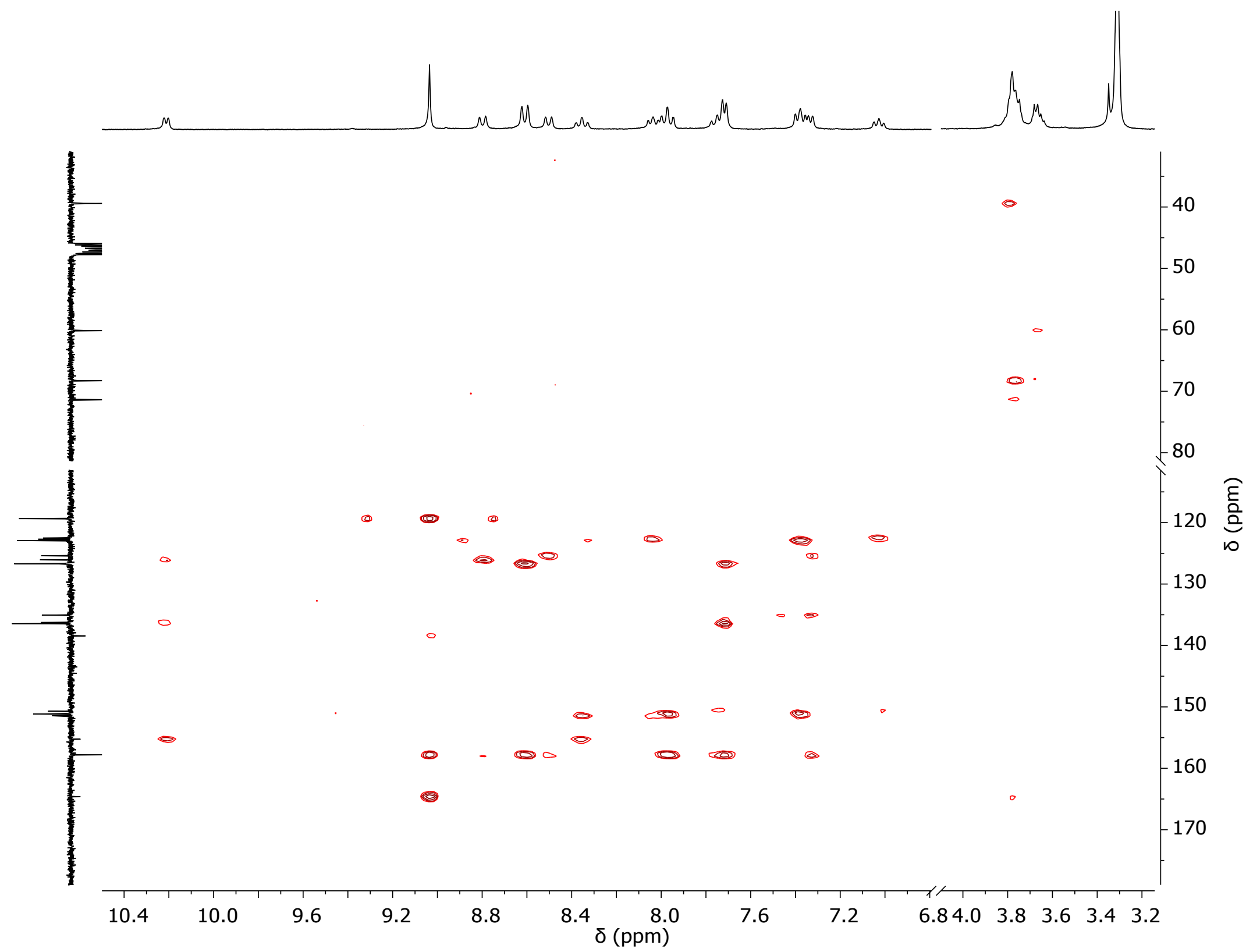

Figure S16. 2D ^{1}H-^{13}C HMBC NMR spectrum of complex **2**.

1.3. HRMS (ESI+) spectra

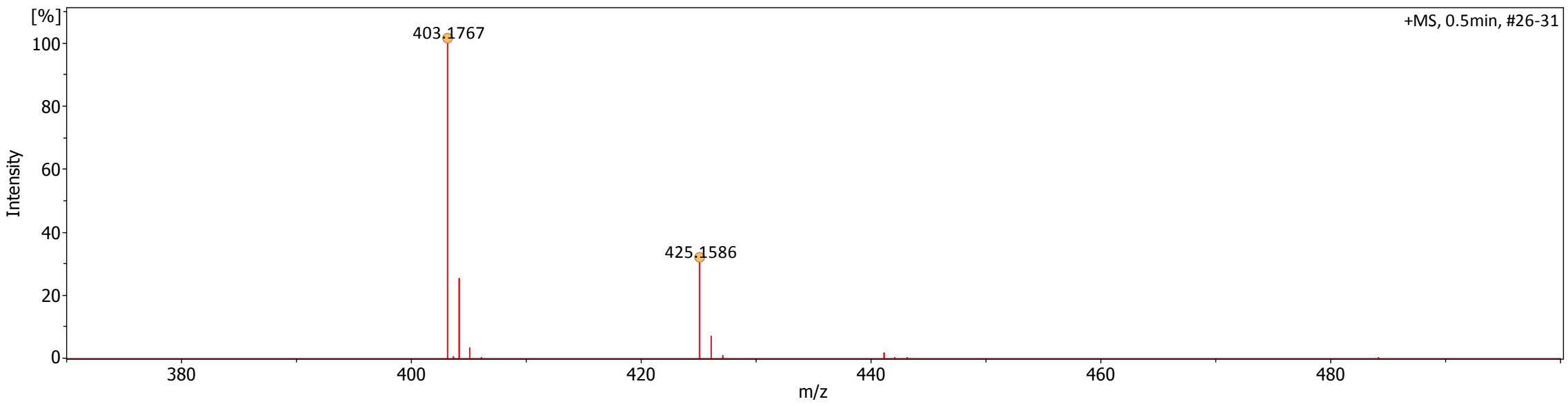

Figure S17. HRMS (ESI^+) spectrum: m/z $[M+H]^+$ and m/z $[M+Na]^+$ of ligand **L**.

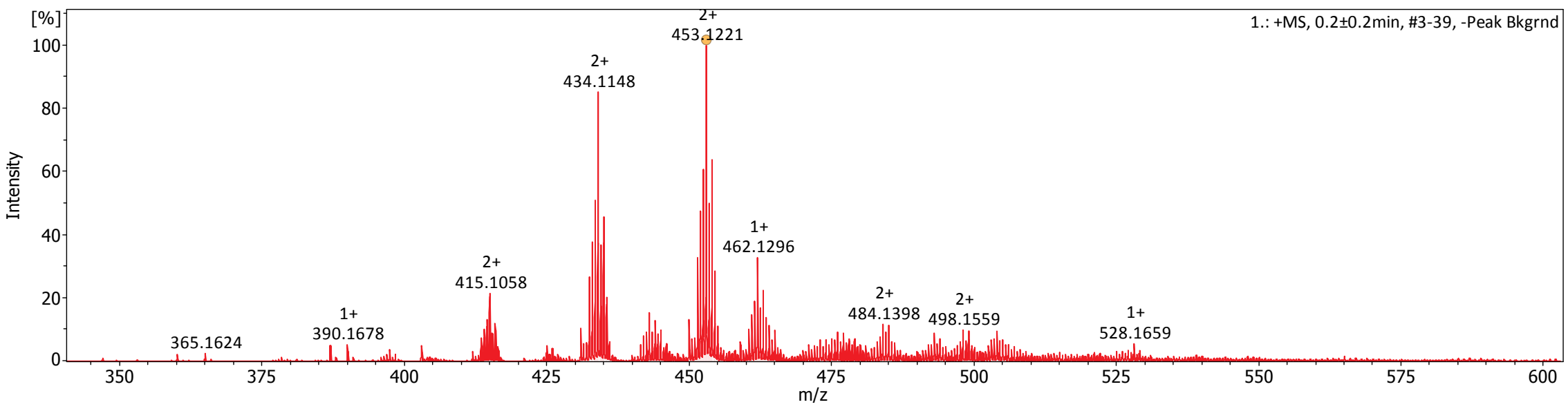

Figure S18. HRMS (ESI^+) spectrum: m/z $[M]^{2+}$ of complex **1**.

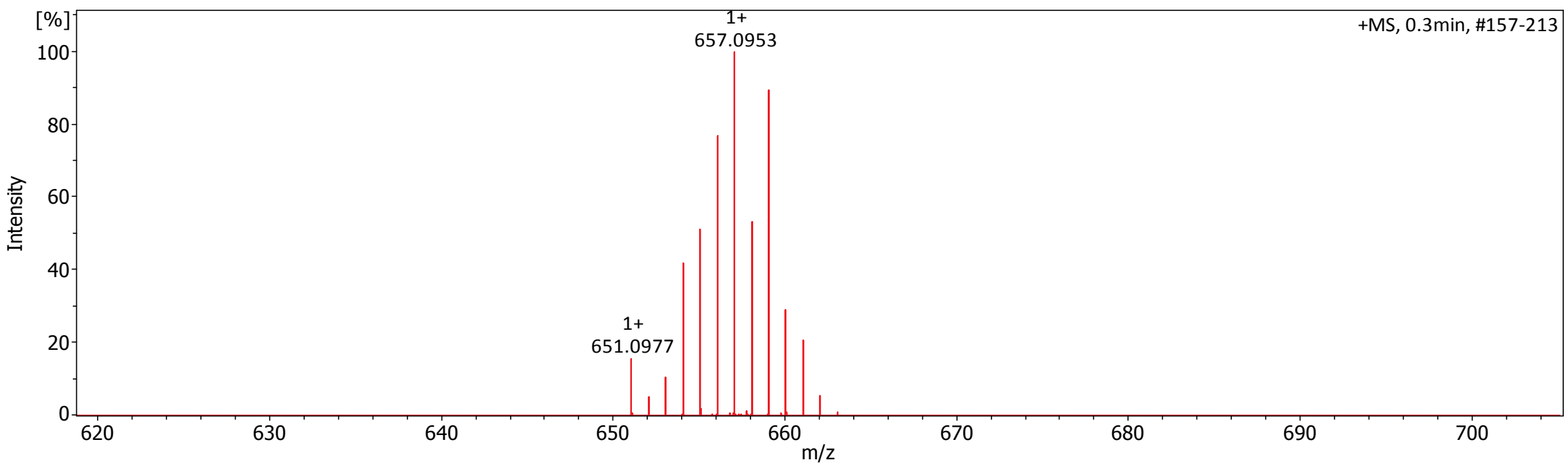

Figure S19. HRMS (ESI^+) spectrum: m/z $[M]^+$ of complex **2**.

1.4. FT/IR SPECTRA

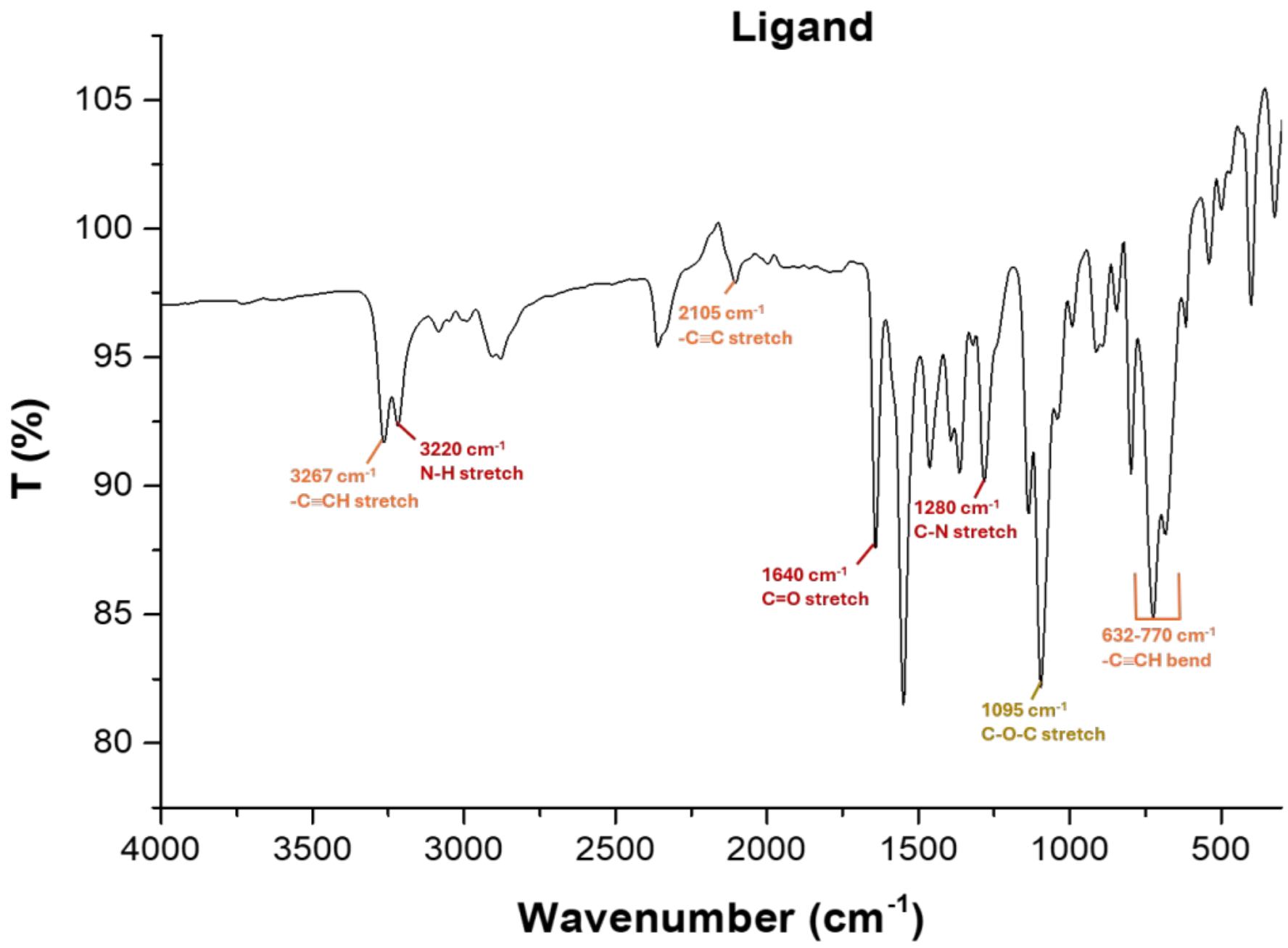

Figure S20. FT/IR spectrum of **L** recorded in solid state in the range of 4000-400 cm^{-1}

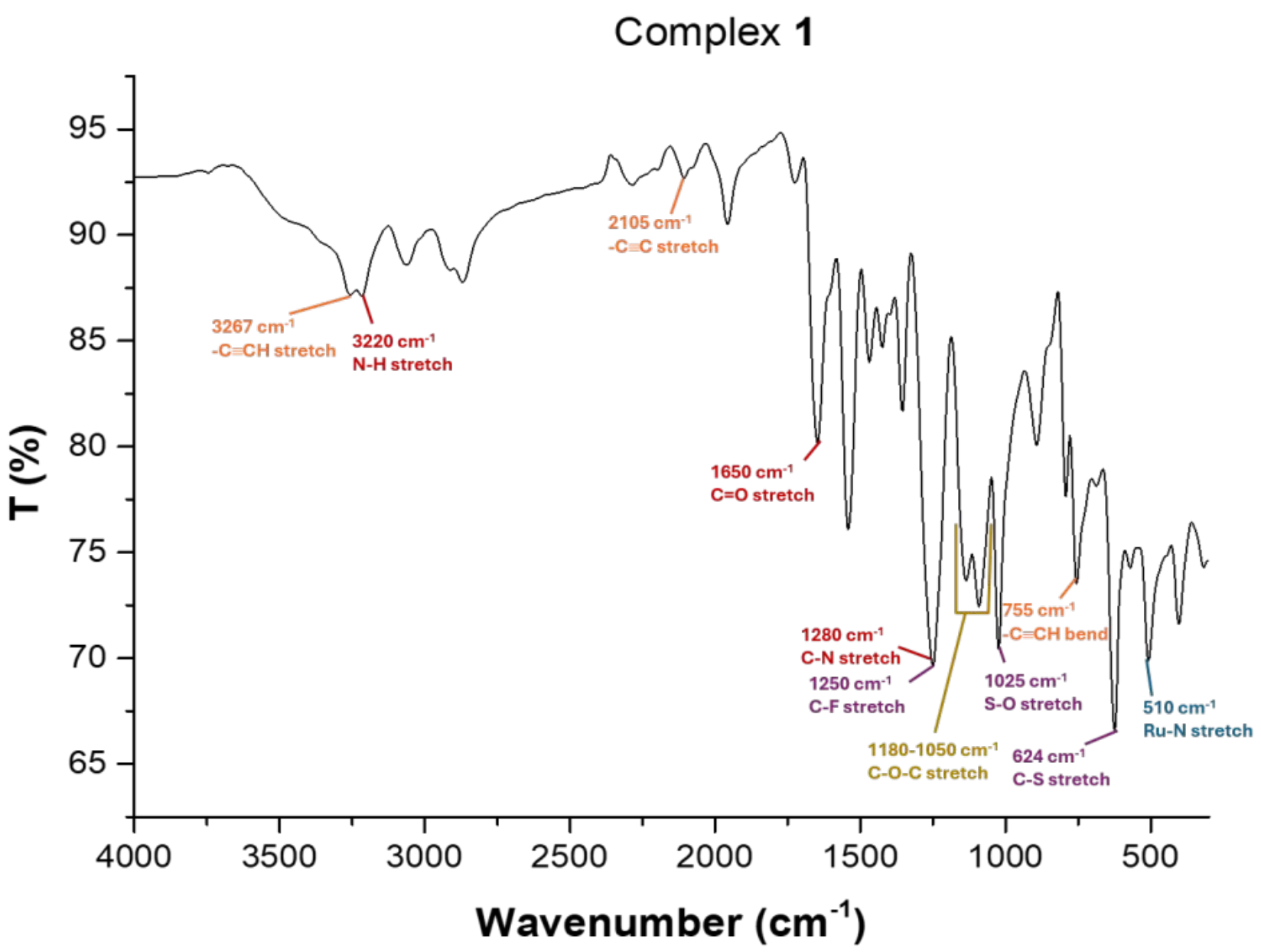

Figure S21. FT/IR spectrum of **1** recorded in solid state in the range of 4000-400 cm^{-1}

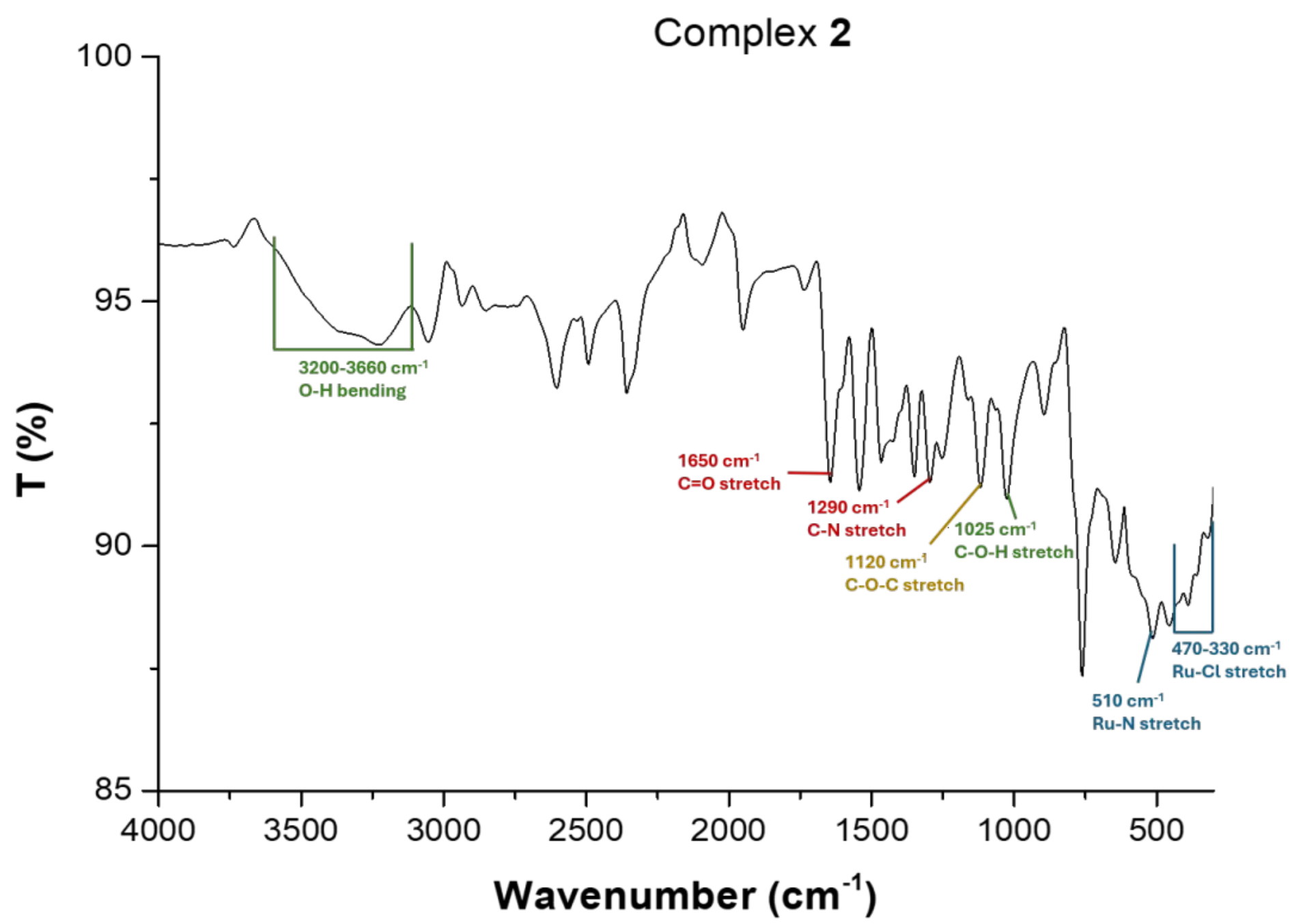

Figure S22. FT/IR spectrum of **2** recorded in solid state in the range of 4000-400 cm^{-1}

2. Stability and photostability

Instrumentation: UV-Vis spectra were recorded with JASCO V-780 spectrophotometer. ^1H NMR spectra were recorded on Bruker ARX300 or AV300 spectrometers. Solutions were irradiated using a Kessil PR160L-470 nm (137 mW/cm^2) (blue light). Doses can be calculated at each time using the following equation: Dose (J/cm^2) = Power Density (mW/cm^2) x time (s) x 0.001.

2.1. UV-Vis spectra

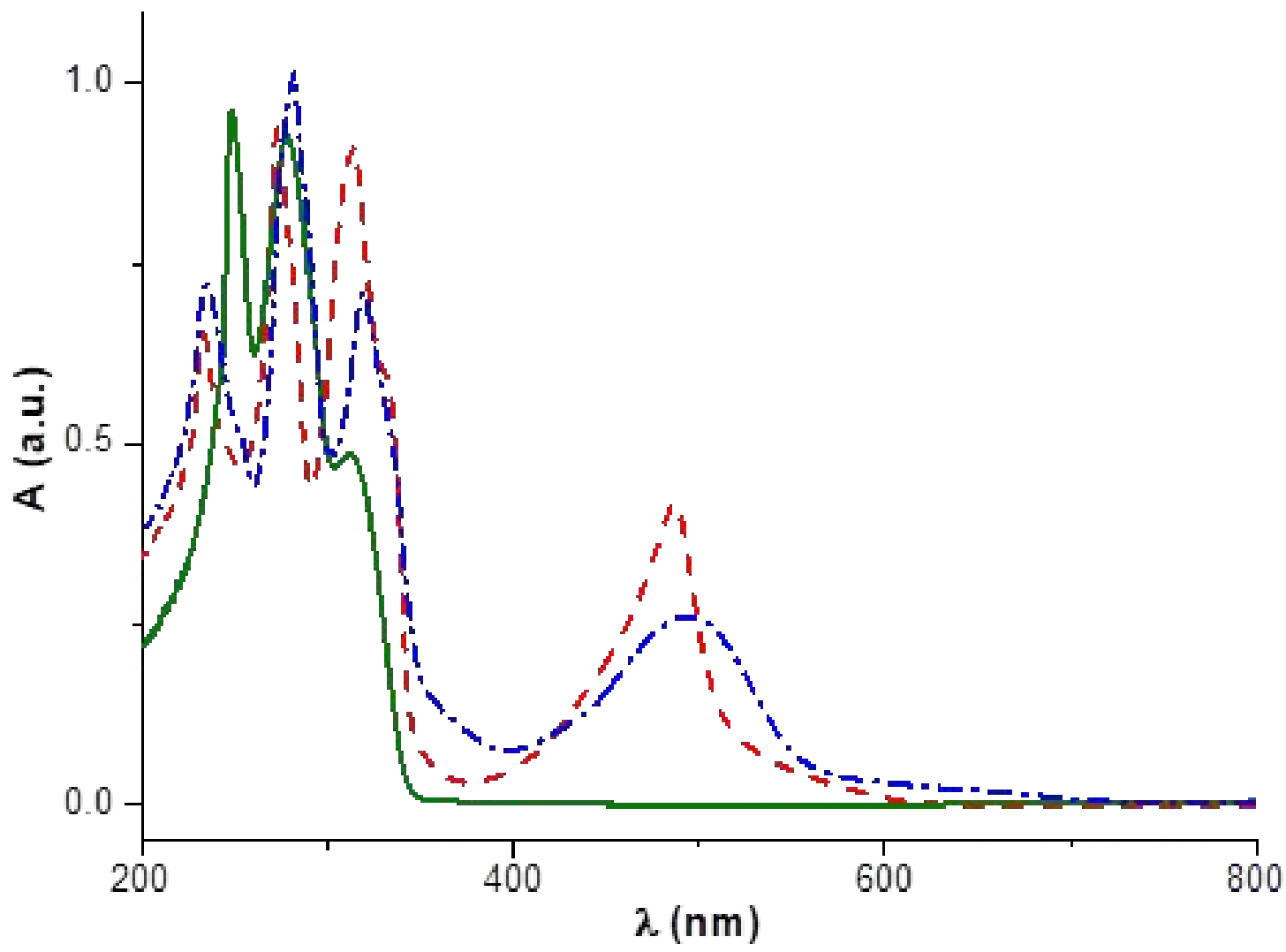

Figure S23. UV-Vis absorption spectra of **L** (DMEM:DMSO; 50:50, 50 μM, green), **1** (DMEM, 30 μM, red) and **2** (DMEM, 30 μM, blue).

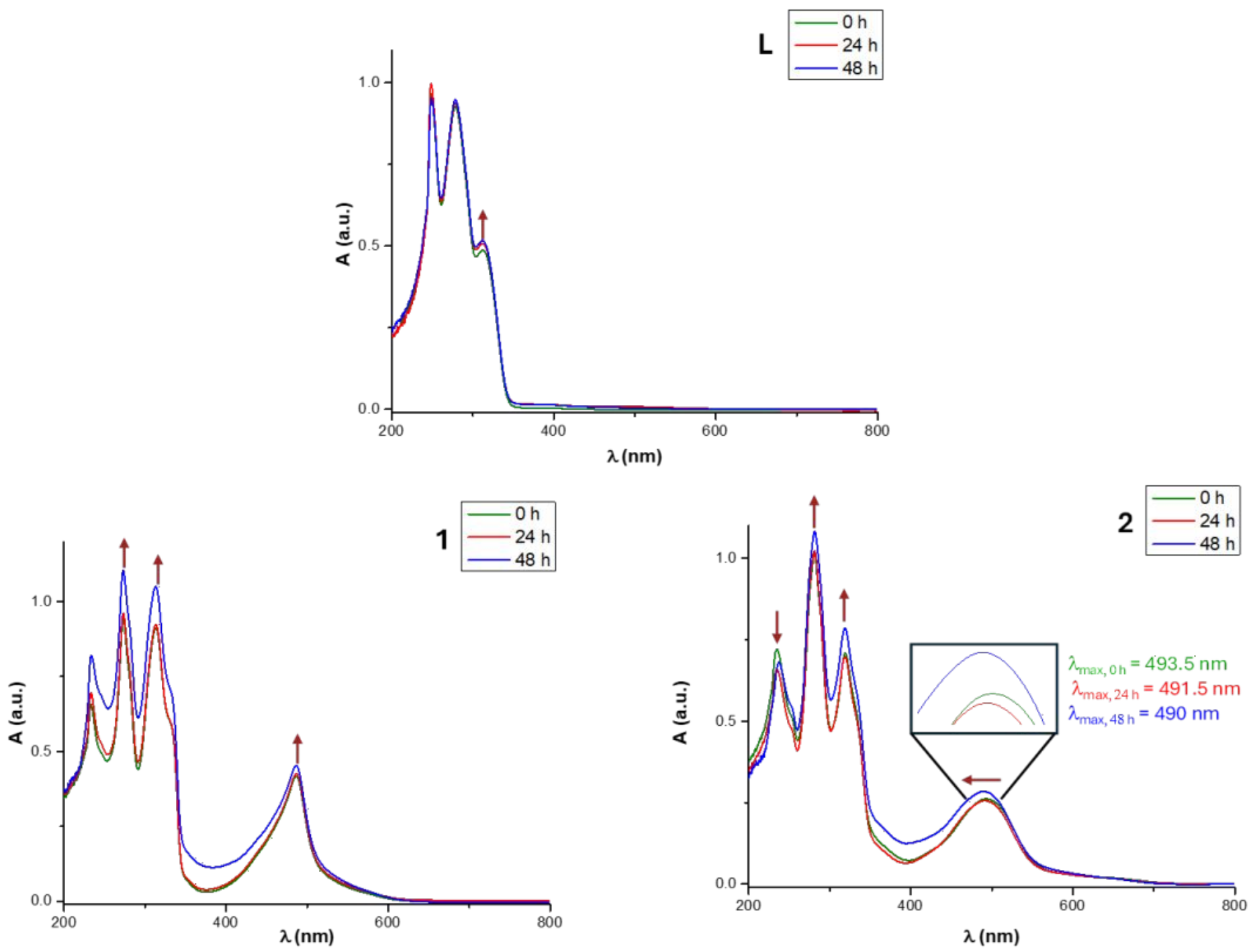

Figure S24. Stability of **L**, **1** and **2** in dark. UV-vis absorption spectra at 0, 24 and 48 h of **L** (DMEM:DMSO; 50:50, 50 µM), **1** (DMEM, 30 µM) and **2** (DMEM, 30 µM). Spectral changes marked with arrows.

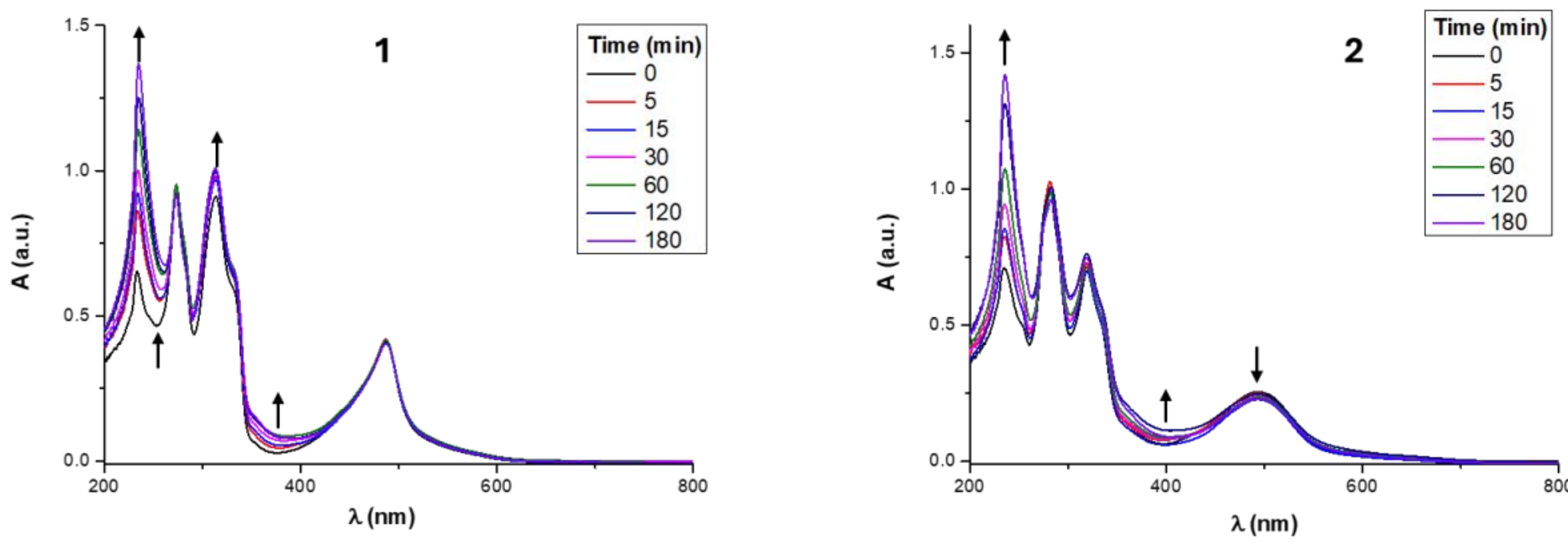

Figure S25. Photostability of complexes **1** and **2** under irradiation at 470 nm. UV-vis spectra of **1** and **2** (30 µM, DMEM) at different times up to 3 hours. Spectral changes are indicated by arrows.

2.2. ^{1}H NMR spectra

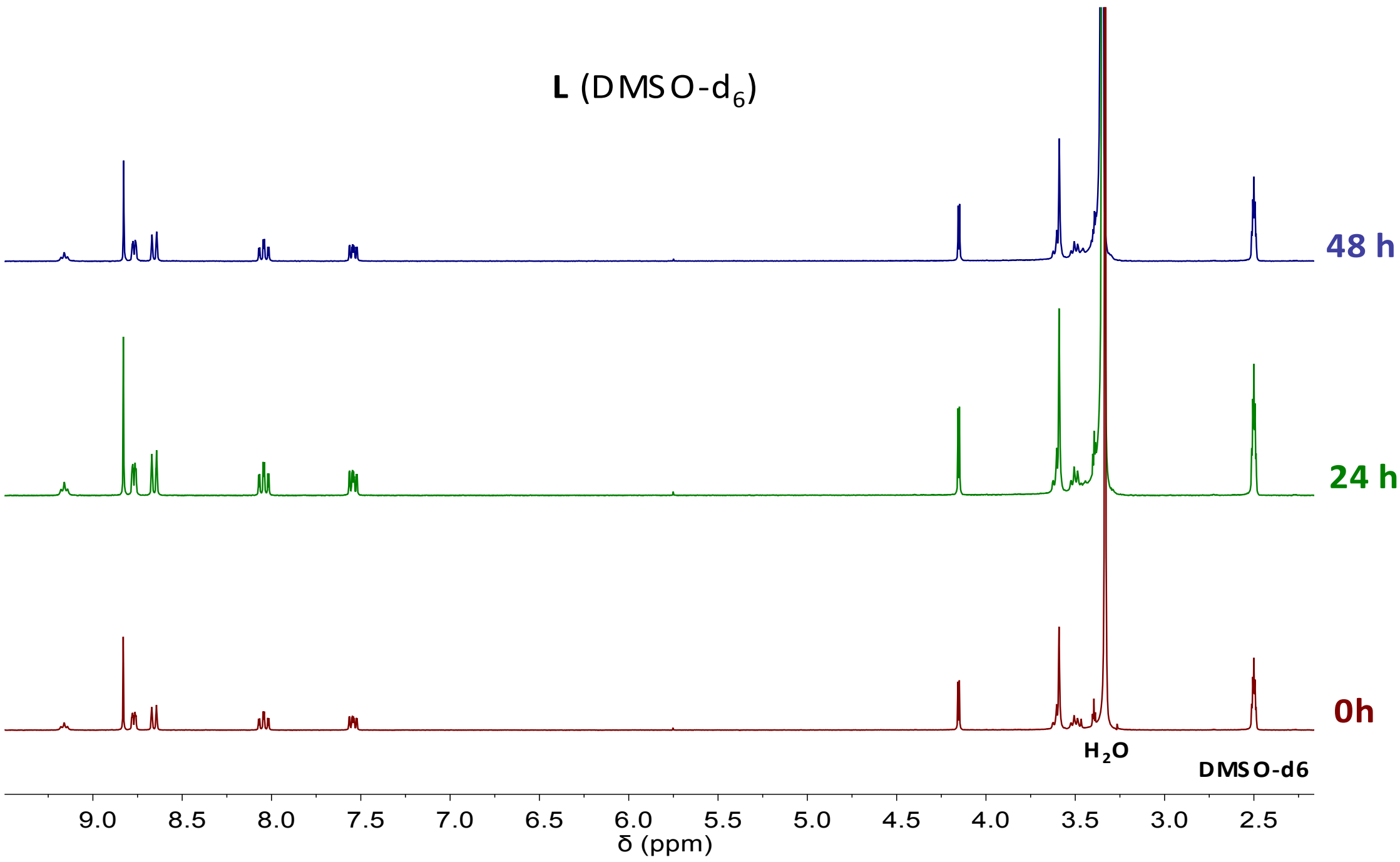

Figure S26. Stability of **L** in dark. ^{1}H NMR spectra of **L** in DMSO-d_6 at 0, 24 and 48 h.

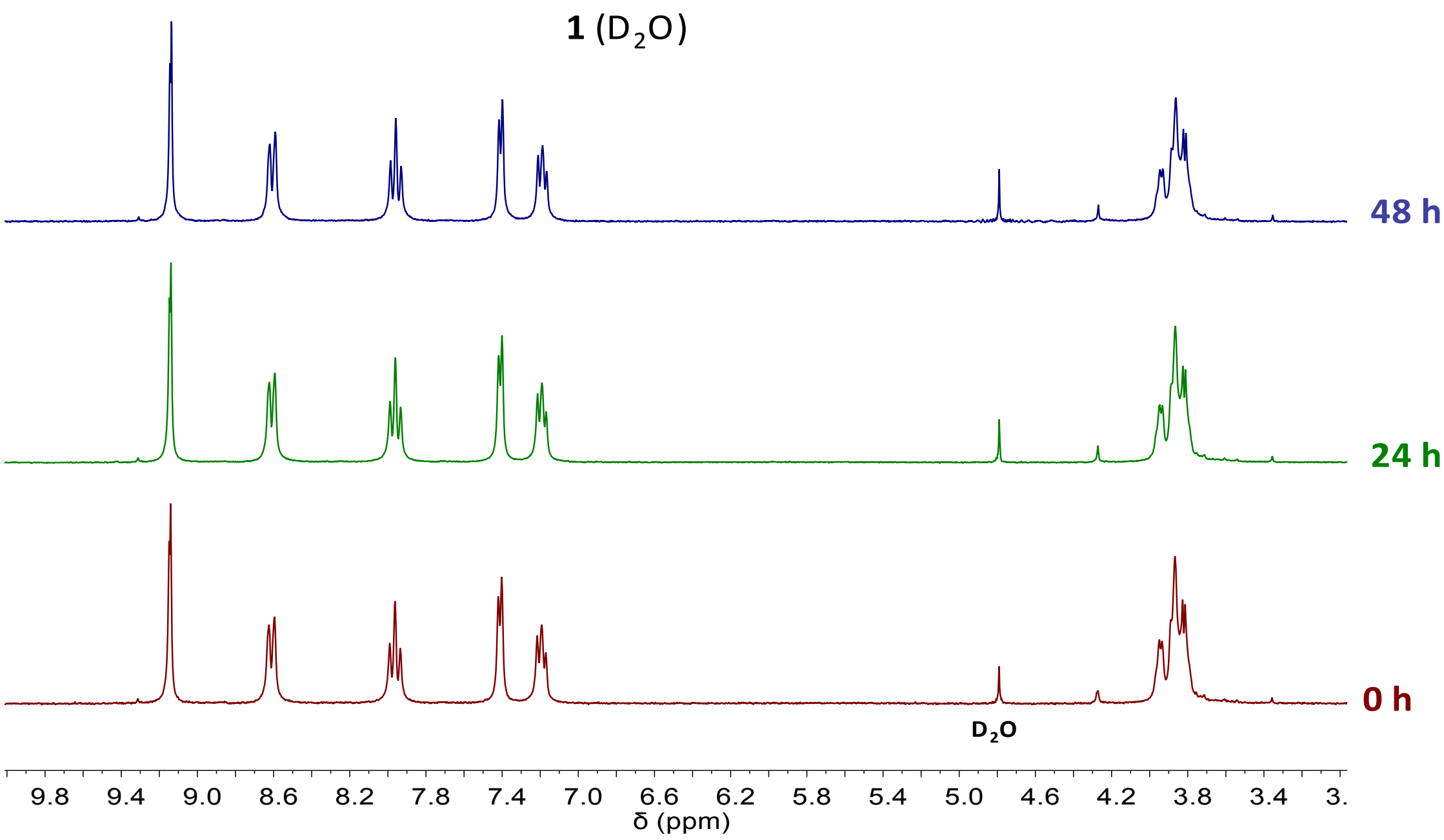

Figure S27. Stability of complex **1**. ^{1}H NMR spectra of complex **1** in D_2O at 0, 24 and 48 h.

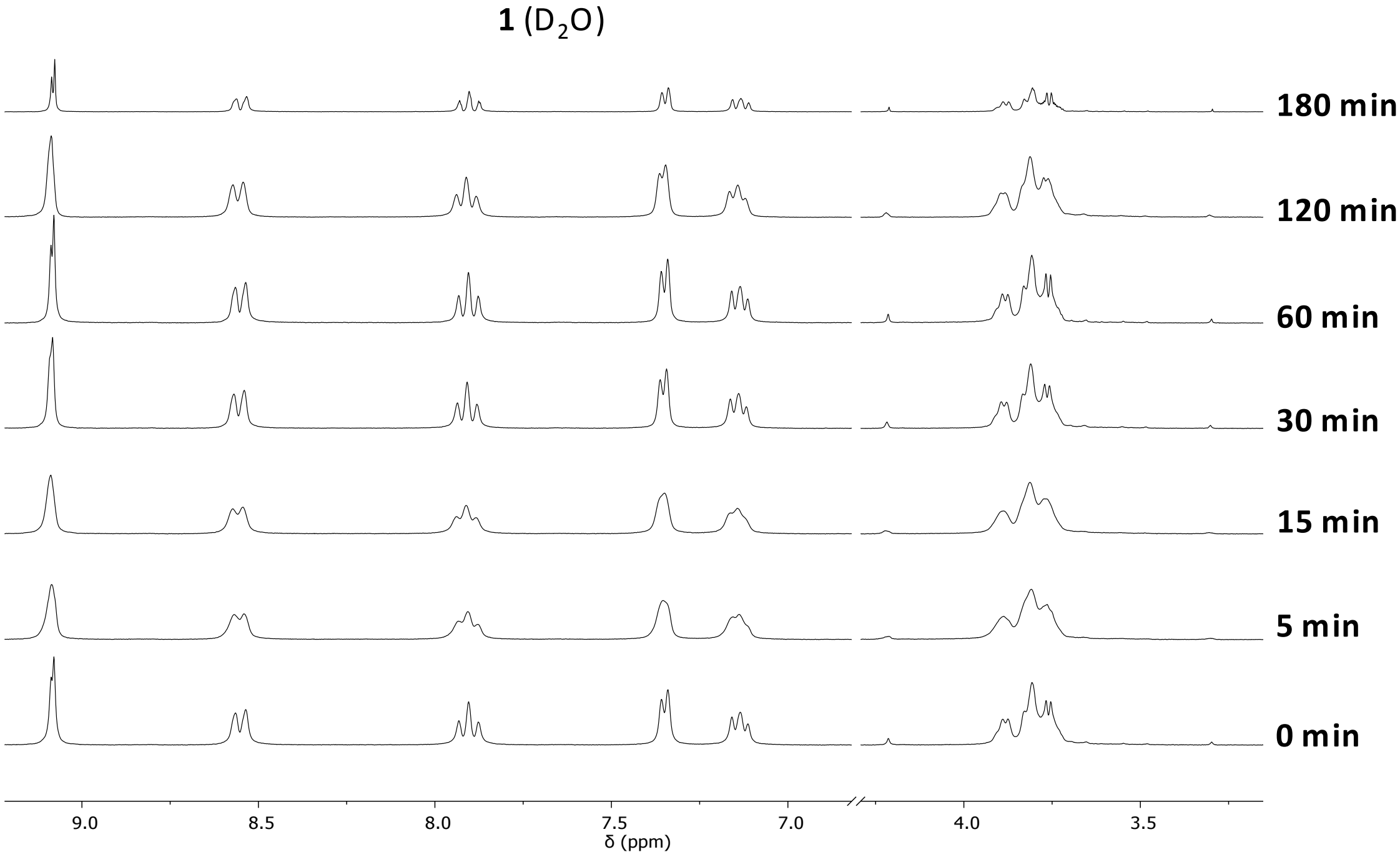

Figure S28. Photostability of complex **1** under irradiation at 470 nm. ^{1}H NMR spectra of **1** in D_2O at different times up to 3 hours.

2.2.1. Hydrolysis of complex 2: rate and equilibrium constant calculation

To monitor the substitution of the chloride ligand by a molecule of solvent (D_2O) Time-dependent ^{1}H NMR spectra were recorded (see Figure S26). The signal corresponding to bipyridine proton 19 (resonance at 9.93 ppm for [**2**-Cl]$^+$ and at 9.54 ppm for aqua complex [**2**-D_2O]$^{2+}$) was integrated for both species at each time point (Figure S27). The integrals were normalized to the total signal intensity, yielding mole fractions of both complexes. The evolution of the molar fraction of **[2-Cl]$^+$** was analyzed according to a reversible substitution first-order model shown in **Scheme S1**.

$$[2\text{-Cl}]^+ \underset{k_{-1}}{\overset{k_1}{\rightleftharpoons}} [2\text{-D}_2\text{O}]^{2+} + \text{Cl}^-$$

Scheme S1. Ligand exchange equilibrium for complex **2**

To calculate the kinetic parameters, data obtained were fitted to **Eq. S1**:

$$\ln\left(x_{Cl}(t) - x_{Cl,\,eq}\right) = \ln\left(x_{Cl,0} - x_{Cl,\,eq}\right) - (k_1 + k_{-1})\cdot t \qquad \textbf{(Eq. S1)}$$

Where $x_{Cl}(t)$ is the mole fraction of species **[2-Cl]$^+$** at time t and $x_{Cl,\,eq}$ the mole fraction upon reaching equilibrium (obtained from spectrum at the end of reaction (at 24 h in dark and 1 h in irradiated). The slope of the linear fit of the equation (Figure S28) gives k_{tot} according to **Eq. S2**

$$m = -\,k_{tot} = -\,(k_1 + k_{-1}) \qquad \textbf{(Eq. S2)}$$

The equilibrium constant k_{e} was calculated from the spectrum at the end of reaction (**Eq. S3**)

$$k_e = \frac{x_{Cl,\,eq}}{x_{D2O,\,eq}} = \frac{k_1}{k_{-1}} \qquad \textbf{(Eq. S3)}$$

Finally, the individualized constants and the half-life of the reaction were obtained from **Eq. S4** and **S5**, respectively.[1]

$$k_{-1} = \frac{k_{tot}}{1+k_e}; \; k_1 = \frac{k_e}{1+k_e} k_{tot} \qquad \text{(Eq. S4)}$$

$$t_{1/2} = \frac{\ln(2)}{k_{tot}} \qquad \text{(Eq. S5)}$$

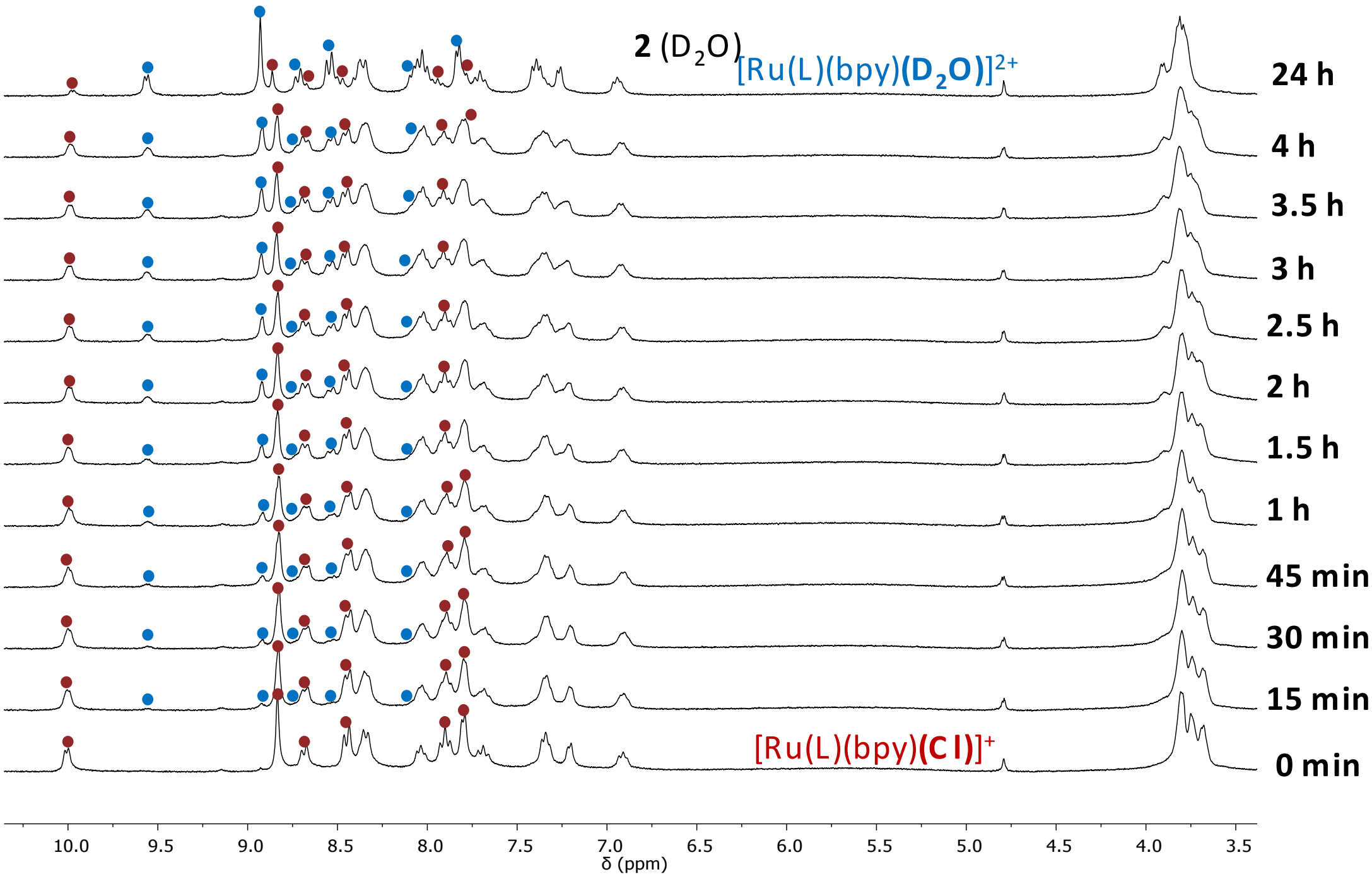

Figure S29. Time evolution of ^{1}H NMR of complex **2** during the hydrolysis of the Cl^- ligand upon dissolution in D_2O up to 48 h. Peaks corresponding to species [**2**-Cl]$^+$ and [**2**-D_2O]$^{2+}$ are marked in red and blue, respectively.

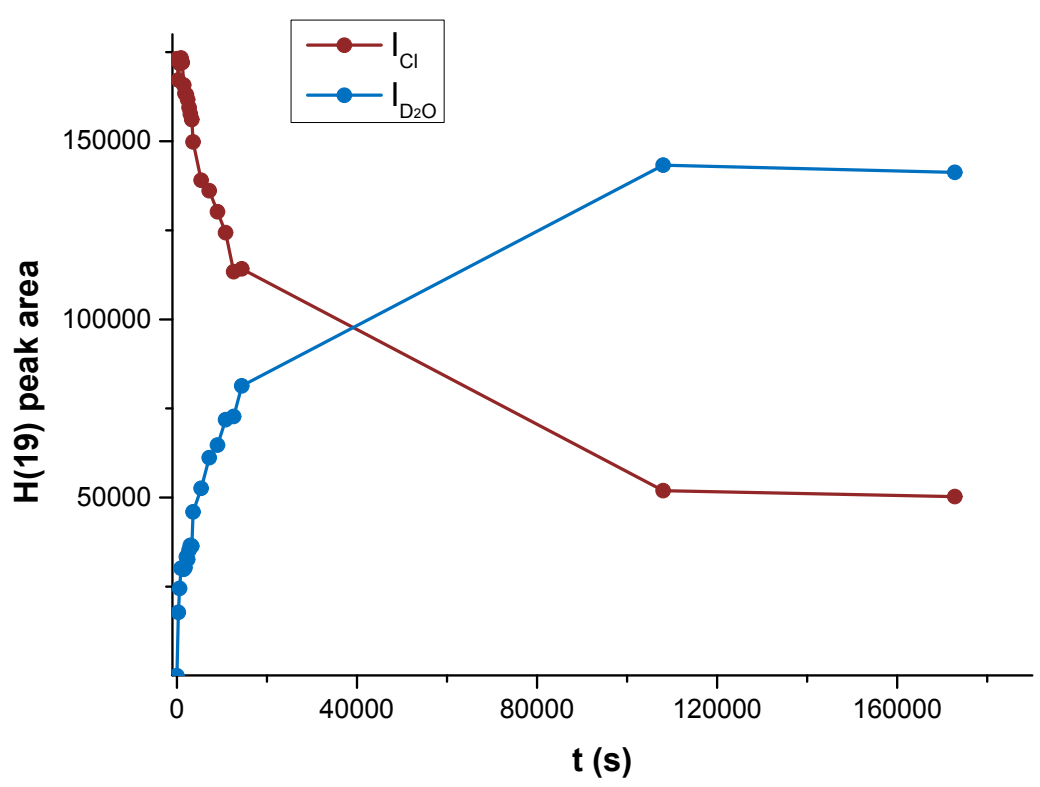

Figure S30. Peak area of H (19) vs time for species [**2**-Cl]$^+$ (red) and [**2**-D_2O]$^{2+}$ (blue) in dark.

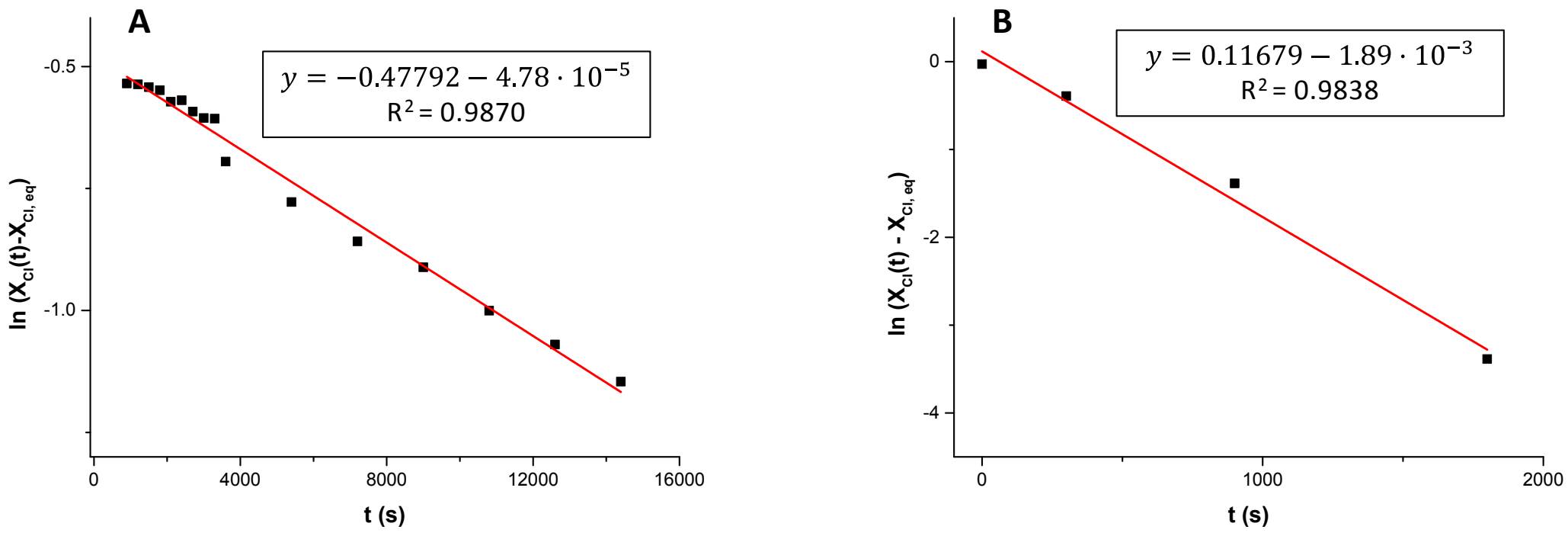

Figure S31. Linear fit of $\ln\left(x_{Cl,0} - x_{Cl,\,eq}\right)$ vs t in dark (A) and under irradiation at 470 nm (B).

Table S1. Calculated kinetic parameters for hydrolysis of complex **2** in dark and under irradiation at 470 nm.

	k_e	k_{tot}	k_1	k_{-1}	$t_{1/2}$
**Dark**	2.76	$4.78 \cdot 10^{-5}$	$3.51 \cdot 10^{-5}$	$1.27 \cdot 10^{-5}$	241.4 min
**Irradiated at 470 nm**	35.29	$1.89 \cdot 10^{-3}$	$1.84 \cdot 10^{-5}$	$5.21 \cdot 10^{-5}$	6.11 min

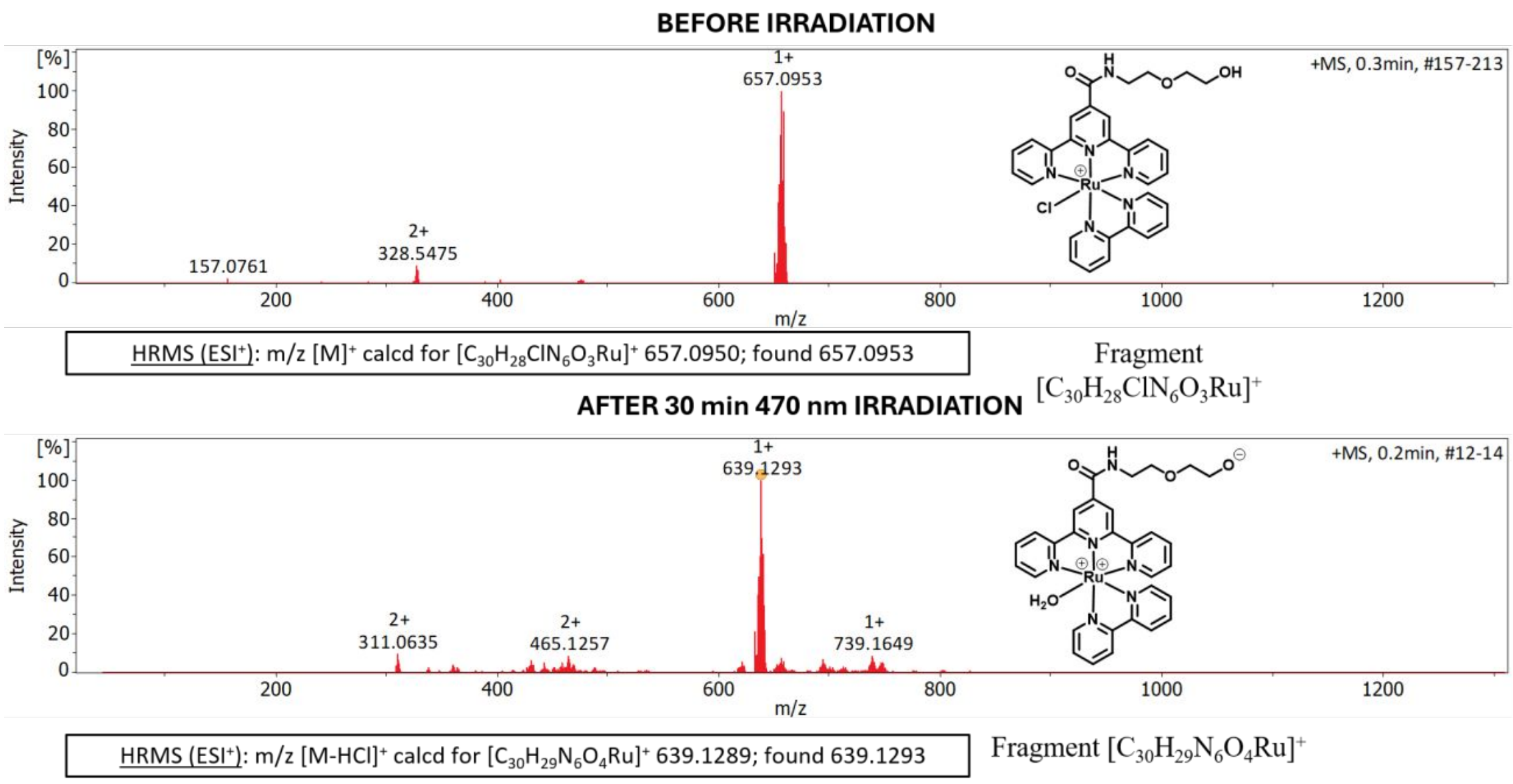

Figure S32. HRMS of complex **2** in H_2O before and after irradiation at 470 nm for 30 min. Structures of the found molecular ions are shown in each corresponding spectrum.

3. Optical properties

Instrumentation: Luminescence spectra and lifetime measurements were recorded with a FluoTime300 PicoQuant spectrometer. Quantum yields were measured by the absolute method using a Hamamatsu Quantaurus-QY C11347 compact one-box absolute quantum yield measurement system.

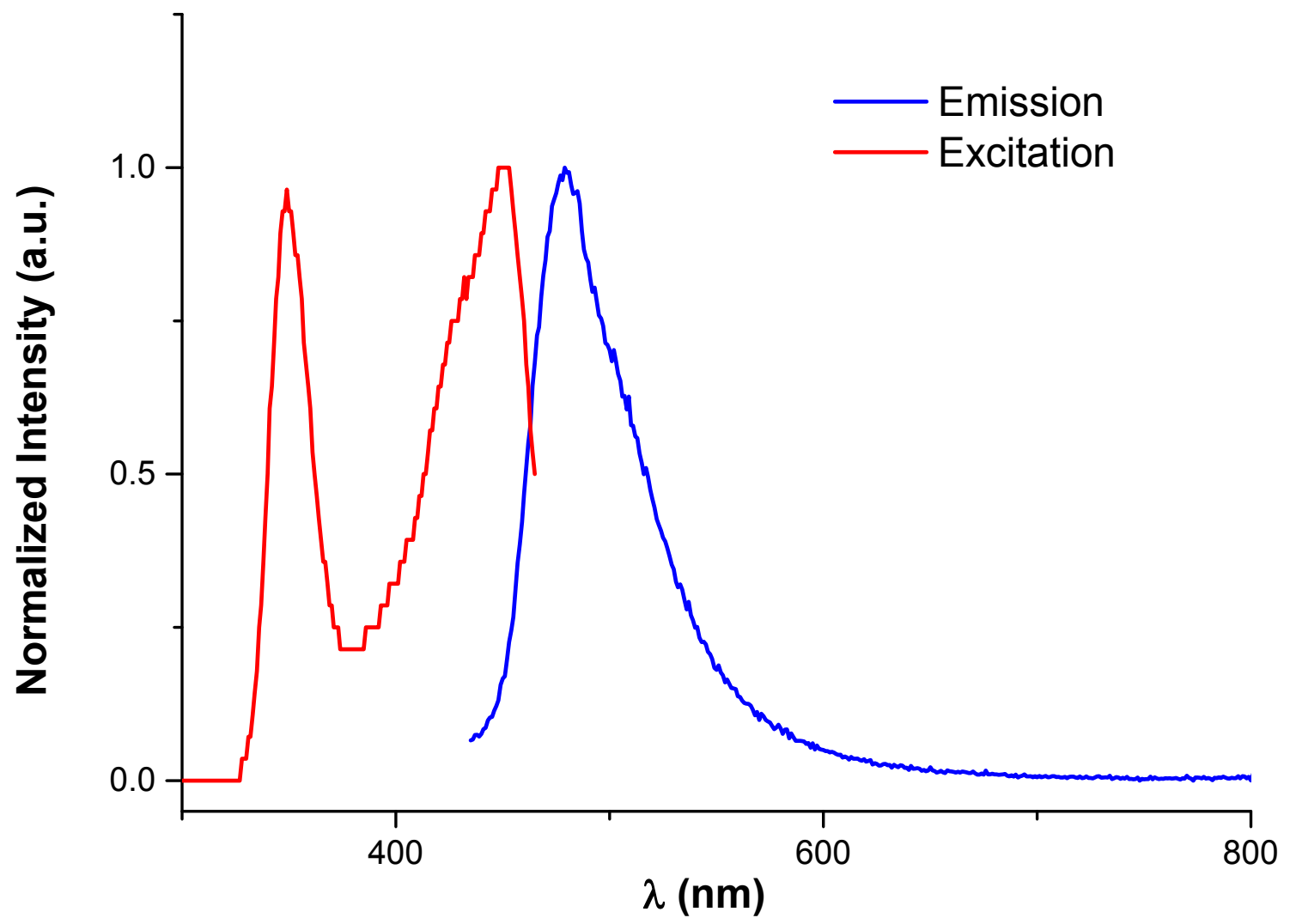

Figure S33. Normalized excitation (λ_{em} = 480 nm) and emission (λ_{ex} = 350 nm) spectra of **L** in DMSO (50 μM) at 298 K.

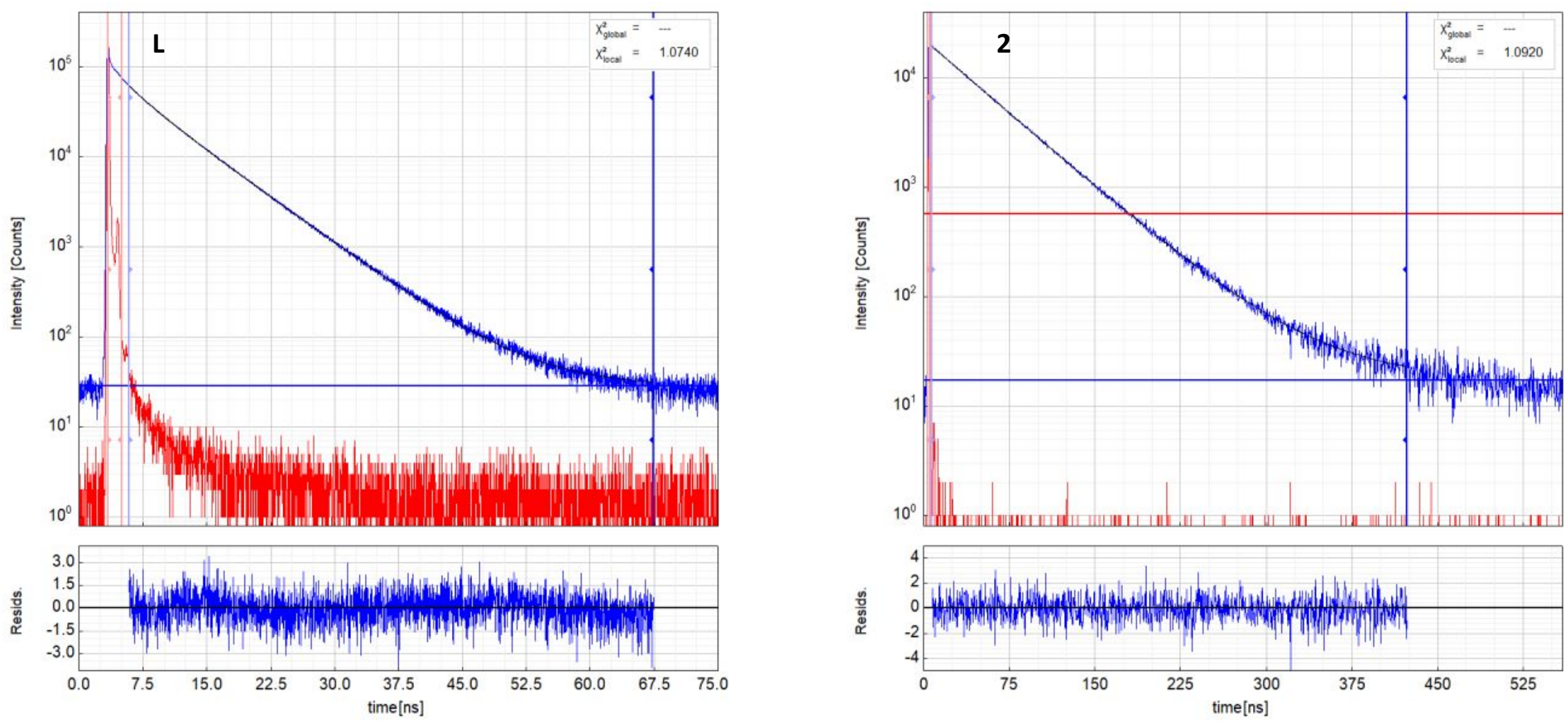

Figure S34. Lifetime fitting curves of **L** (left) and complex **2** (right).

Table S2. Photophysical data of **L**, **1**, and **2** measured in DMSO at 298 K.

	λ_{em} (nm)	λ_{ex} (nm)	τ (ns)	ϕ
**L**	480	350, 450	5.79	< 1%
**1**	n.d.	n.d.	n.d.	n.d.
**2**	770	420, 610, 700	48.82	1.2%

3.1.1.1. Singlet Oxygen Generation Quantum Yield Determination using DPA

Air-saturated acetonitrile solutions containing DPA (9,10-diphenylanthracene) (50 μM) and the corresponding photosensitizer (Complex **1** (30 μM), complex **2** (30 μM) or **[Ru(bpy)$_3$]Cl$_2$** (13.5 μM)) were prepared in a quartz cuvette. The solutions were irradiated at 470 nm, and the consumption of DPA was monitored by recording UV-vis spectra at different irradiation times and monitoring the decrease of the characteristic band at 373 nm.[2]

The singlet oxygen quantum yield (ϕ) of complexes **1** and **2** was determined by the following equation:

$$\Phi_{\Delta \mathrm{SAMPLE}} = \Phi_{\Delta \mathrm{REF}} \cdot \frac{k_{\mathrm{SAMPLE}}}{k_{\mathrm{REF}}} \cdot \frac{1\text{-}10^{-A(ref)}}{1\text{-}10^{-A(sample)}} \qquad \textbf{(Eq. S6)}$$

Where $\Phi_{\Delta REF}$ is the singlet oxygen quantum yield of $[Ru(bpy)_3]Cl_2$ (0.57), k is the slope obtained from plotting $\ln(A_t/A_0)$ versus irradiation time in seconds for each complex (Figure S35) and A is the absorbance of the complex at 470 nm.

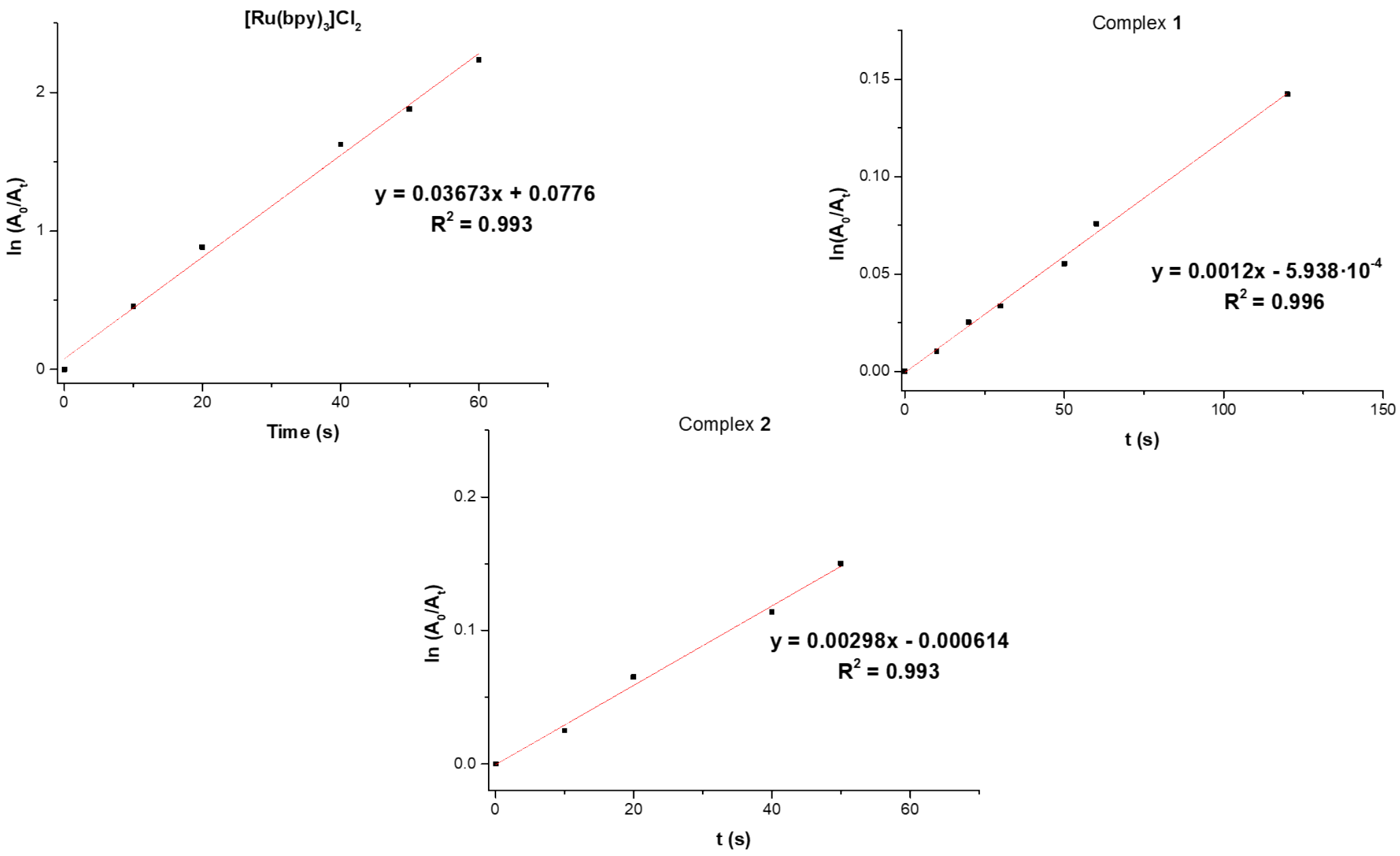

Figure S35. Plot of $\ln(A_t/A_0)$ versus irradiation time (s) for each complex and the reference $[Ru(bpy)_3]Cl_2$.

4. Stern-volmer: emission quenching

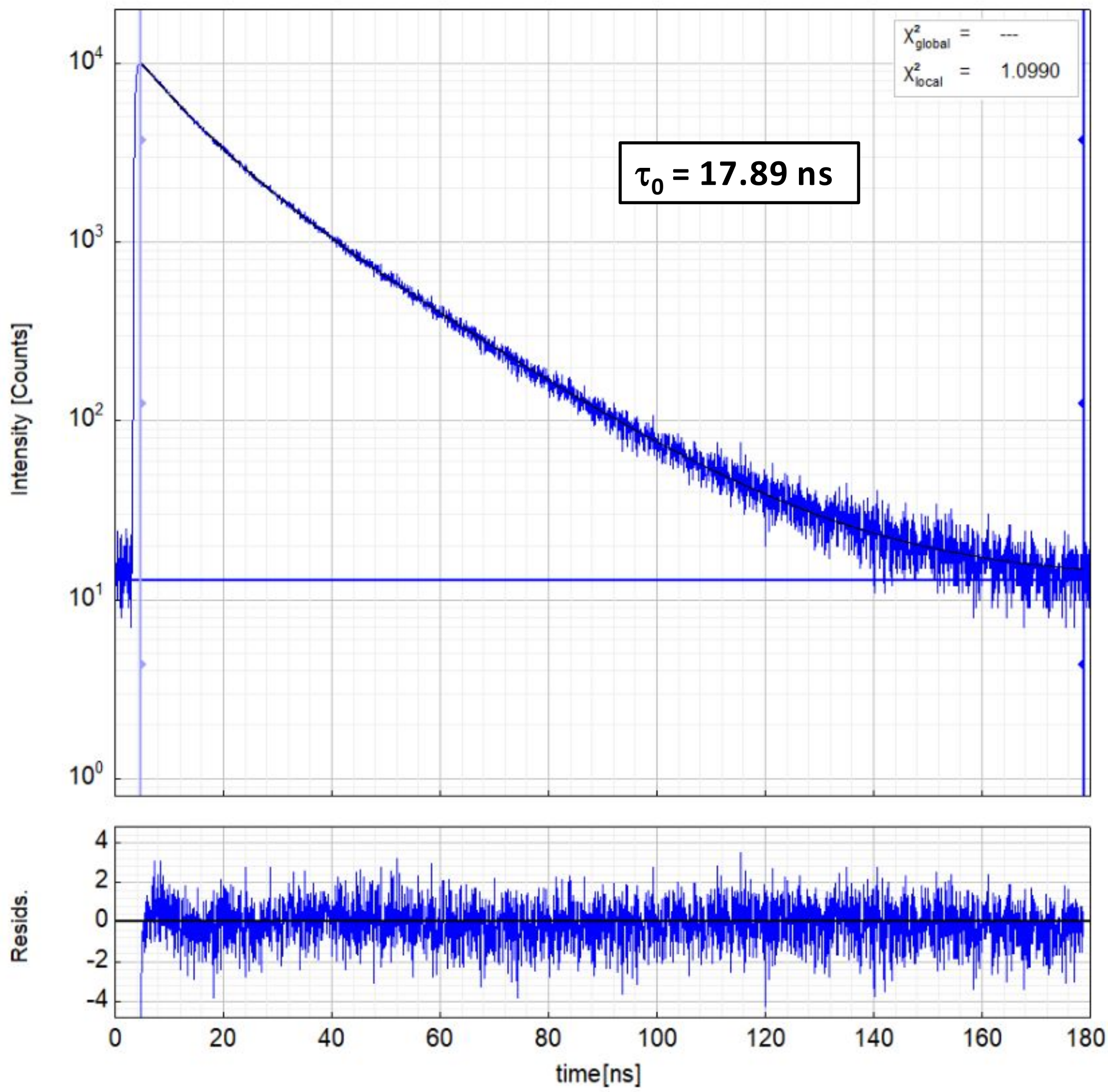

Figure S36. Complex **2** (20 μM) lifetime decay fitting in PBS.

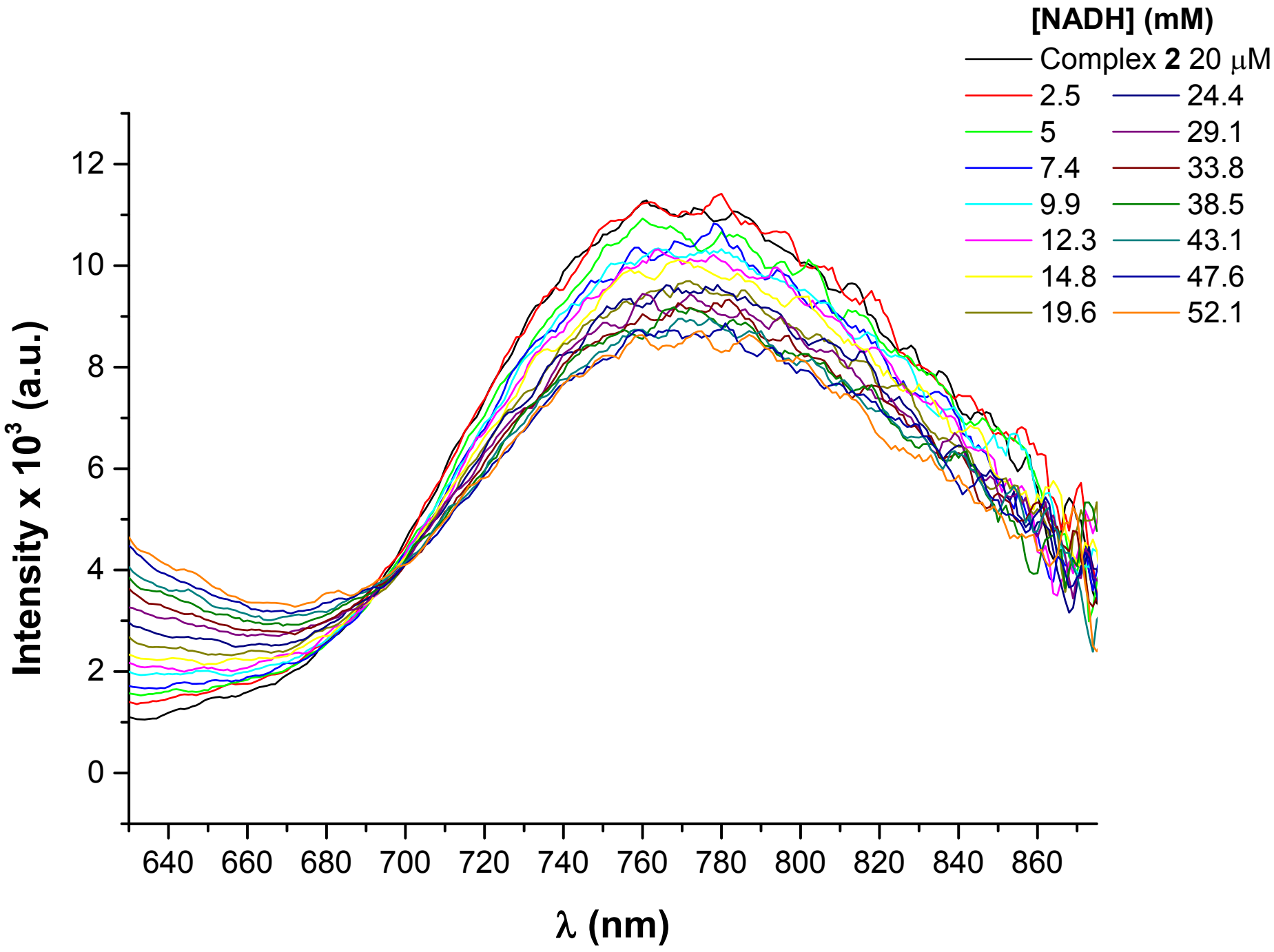

Figure S37. Complex **2** (20 µM) emission spectra upon addition of increasing amounts of NADH.

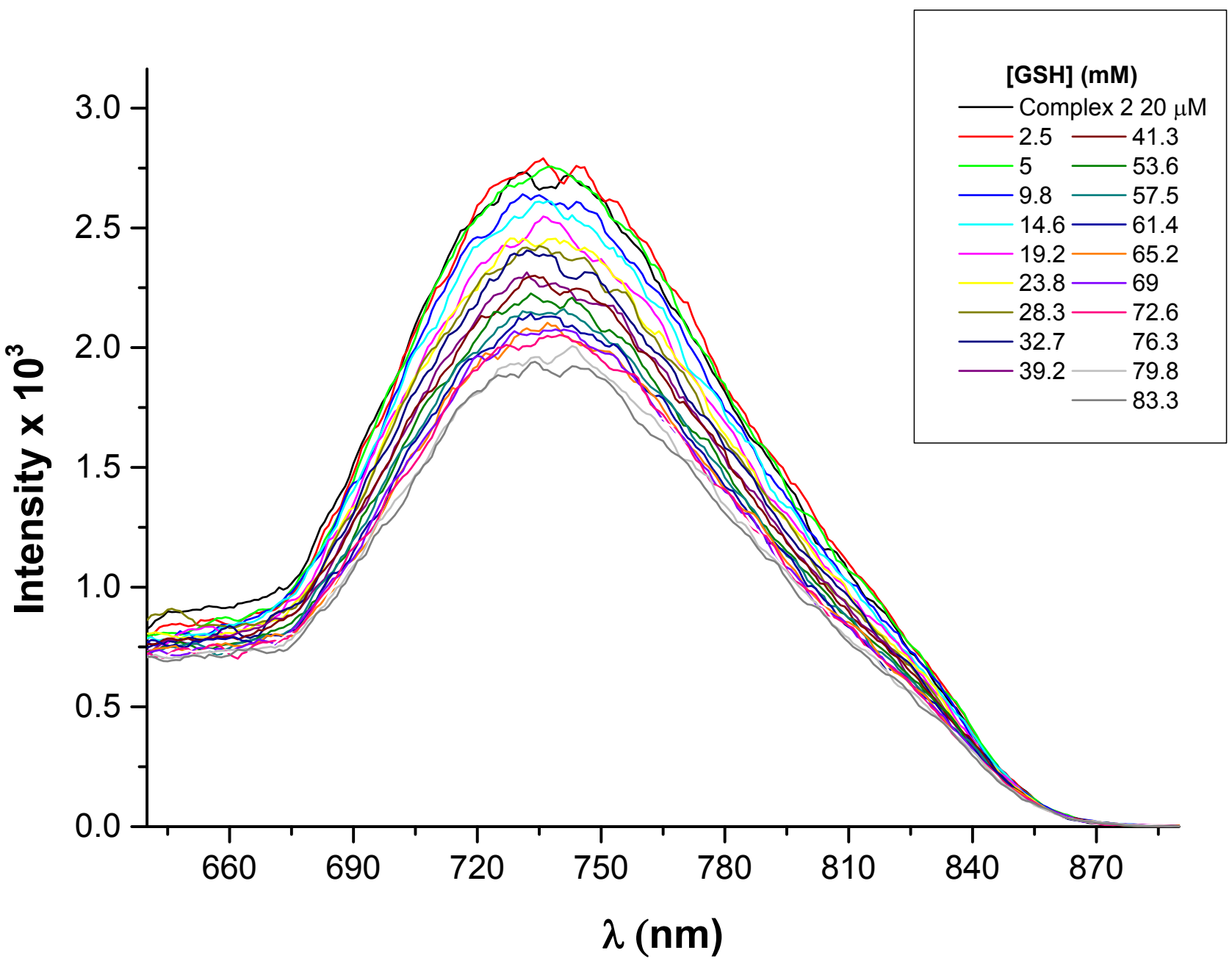

Figure S38. Complex **2** (20 µM) emission spectra upon addition of increasing amounts of GSH.

5. Electrochemical properties

5.1. Cyclic voltammetry

Instrumentation: cyclic voltammetry spectra were recorded with a PalmSens 4 potentiostat. NADH/NAD^+ oxidation potential vs Fc^+/Fc (- 0.32 V) was obtained from the oxidation potential of NADH/NAD^+ vs NHE (+ 0.32 V) and that of Fc^+/Fc (+0.64 V). GSH/GSSG oxidation potential vs Fc^+/Fc (- 0.40 V) was obtained from the oxidation potential of GSH/GSSG vs NHE (+ 0.24 V) and that of Fc^+/Fc (+ 0.64 V). Observed redox potentials are according to those previously described.[3-6]

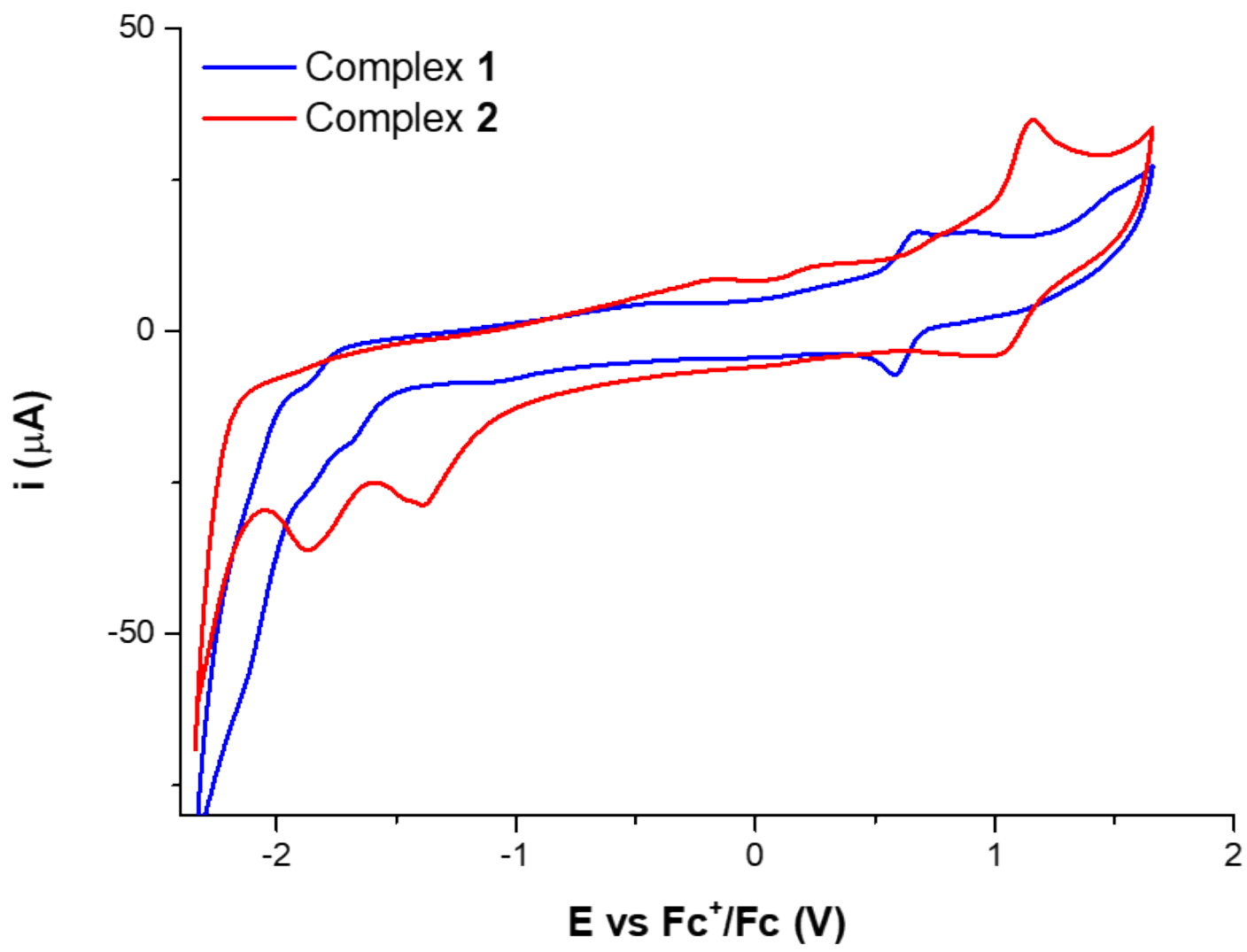

Figure S39. Cyclic voltammogram of complex **1** (500 mM, in blue) and complex **2** (500 mM, in red) at a glassy carbon electrode in DCM/0.1 M $TBAPF_6$ with a scan rate of 100 mV/s.

5.2. Excited state redox potential determination

The excited state redox potential of complex **2** was calculated using the following equation.[7]

$$E^*_{red}(Ru^{+2*}/Ru^+) = E_{red}(Ru^{+2}/Ru^+) + E_{0,0} \quad \textbf{(Eq. S7)}$$

Where $E_{0,0}$ is the excited state energy (in eV), which can be calculated by finding the midpoint between the absorption and emission maxima, in wavelength ($\lambda_{0,0}$) and converting it to energy ($E_{0,0}$=1240 / $\lambda_{0,0}$).

For complex **2**: $E_{0,0}$ = + 2.03 eV, and $E^*_{red}(Ru^{II*}/Ru^{I})$ = + 0.221 V

5.3. Determination of free energy for the photoinduced oxidation (ΔG)

The free energy of the photooxidations was determined using the following equation.[7]

$$\Delta G = -\mathcal{F}[E^*_{red}(Ru^{+2*}/Ru^+) - E_{ox}(sub/sub^{\cdot+})] \quad \textbf{(Eq. S8)}$$

Where $E_{ox}(sub/sub^{\cdot+})$ is the oxidation potential of the substrates (GSH or NADH) and $\mathcal{F}$ is the Faraday constant (96.49 kJ $V^{-1}mol^{-1}$).

For complex **2**:

ΔG (NADH photooxidation) = - 52.20 kJ mol^{-1}

ΔG (GSH photooxidation) = - 59.98 kJ mol $^{-1}$

5.4. Determination of the HOMO-LUMO energy gap

The HOMO and LUMO orbital energies for both complexes was determined using the following equations.[8-10]

$$E_{HOMO} = -(E_{onset(ox)} + 5.1\ eV \quad \textbf{(Eq. S9)}$$

$$E_{LUMO} = E_{HOMO} + E_g\ (eV) \quad \textbf{(Eq. S10)}$$

Where 5.1 eV corresponds to the energy of the Fc/Fc^+ redox couple relative to the vacuum level, and E_g is the bandgap energy in eV (E_g (eV)= 1240/$\lambda_{MLCT(UV\text{-}Vis)}$ (nm)).

For complex **1**: E_{HOMO} = - 5.982 eV; E_{LUMO} = - 3.436 eV and $\Delta E_{H\text{-}L}$ = 2.546 V

For complex **2**: E_{HOMO} = - 5.512 eV; E_{LUMO} = - 3.007 eV and $\Delta E_{H\text{-}L}$ = 2.505 V

6. Photocatalysis

6.1. NADH photooxidation

Instrumentation: UV-Vis spectra were recorded with JASCO V-780 spectrophotometer. Solutions were irradiated using Kessil PR160L-470 nm (137 mW/cm^2, blue light), PR160L-525 nm (137 mW/cm^2, green light) and a homemade 600 nm LED array (16.25 mW/cm^2, red light). Doses can be calculated at each time using the following equation: Dose (J/cm^2) = Power Density (mW/cm^2) x time (s) x 0.001.

Experimental procedure: Catalysts **1** and **2** (catalyst load = 10, 5, 0.5 and 0.05 mol%) were mixed with NADH (200 µM) in PBS. The catalytic reactions were monitored by UV-vis in the dark and under 470, 525 or 600 nm light irradiation. Characteristic band of NADH = 340 nm.

6.1.1. Kinetic study

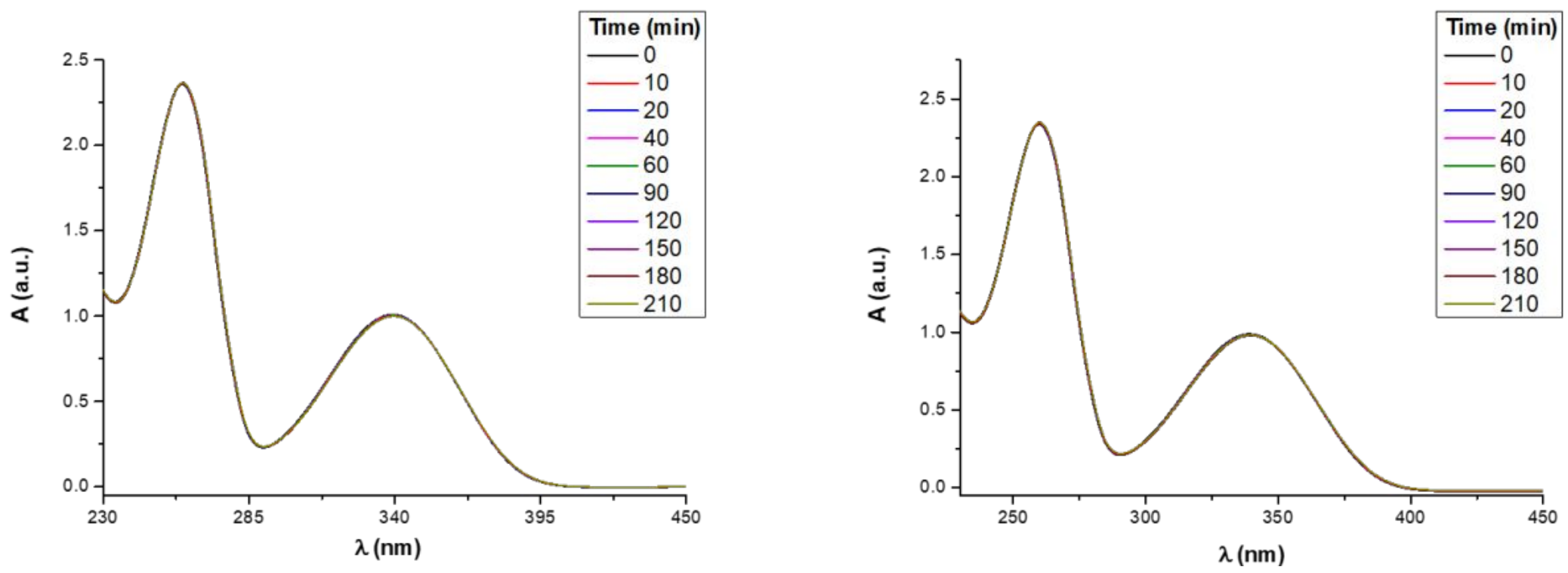

Figure S40. UV-vis spectrum of NADH in presence of **1** (20 µM, 10 mol%, right) and **2** (20 µM, 10 mol%, left) in dark.

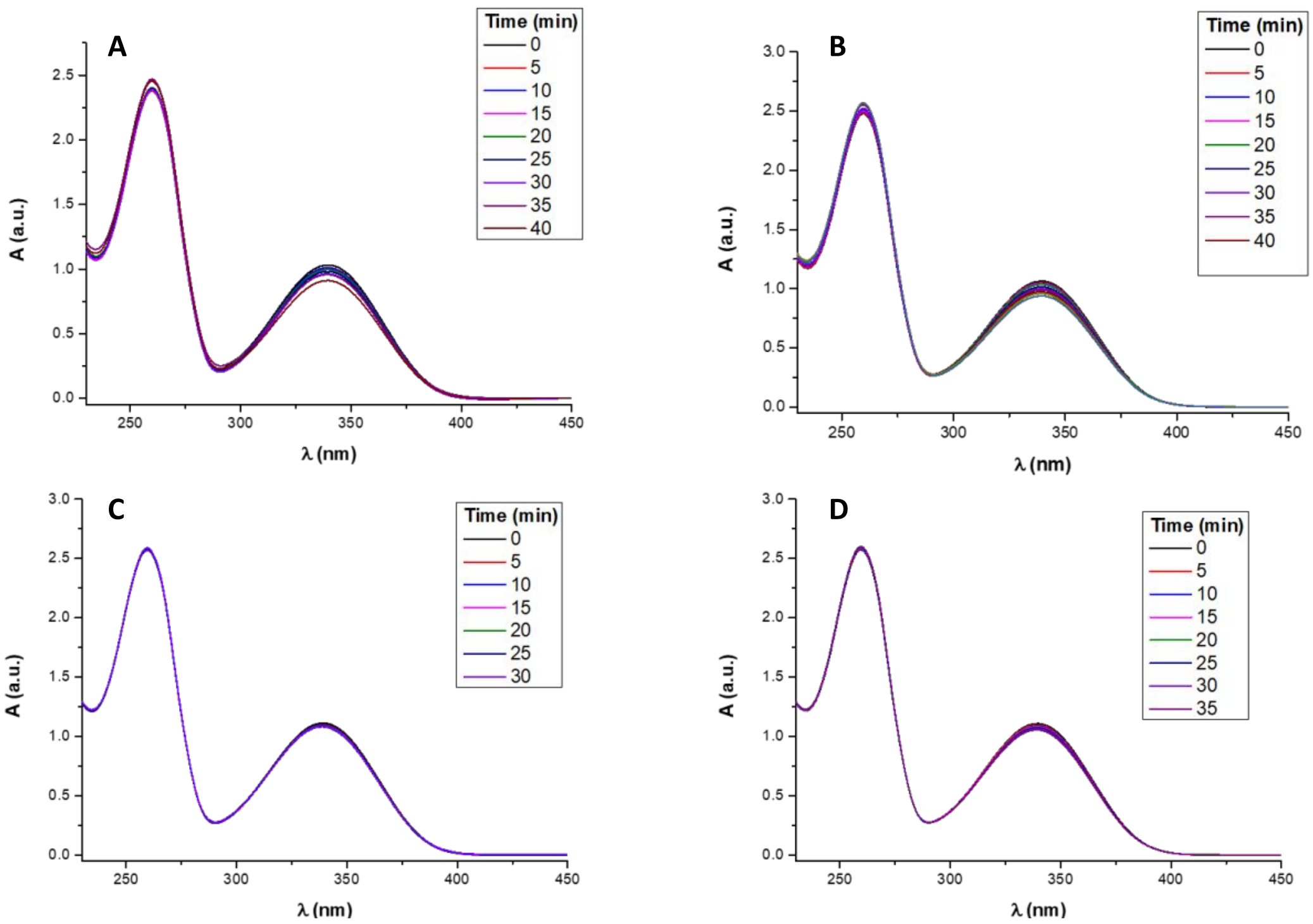

Figure S41. UV-vis spectrum of NADH in presence of catalyst (1 μM; 0.5 mol%) in different conditions: **A** complex **1** under 470 nm irradiation, **B** complex **2** under 470 nm irradiation, **C** complex **1** under 525 nm irradiation, **D** complex **2** under 525 nm irradiation.

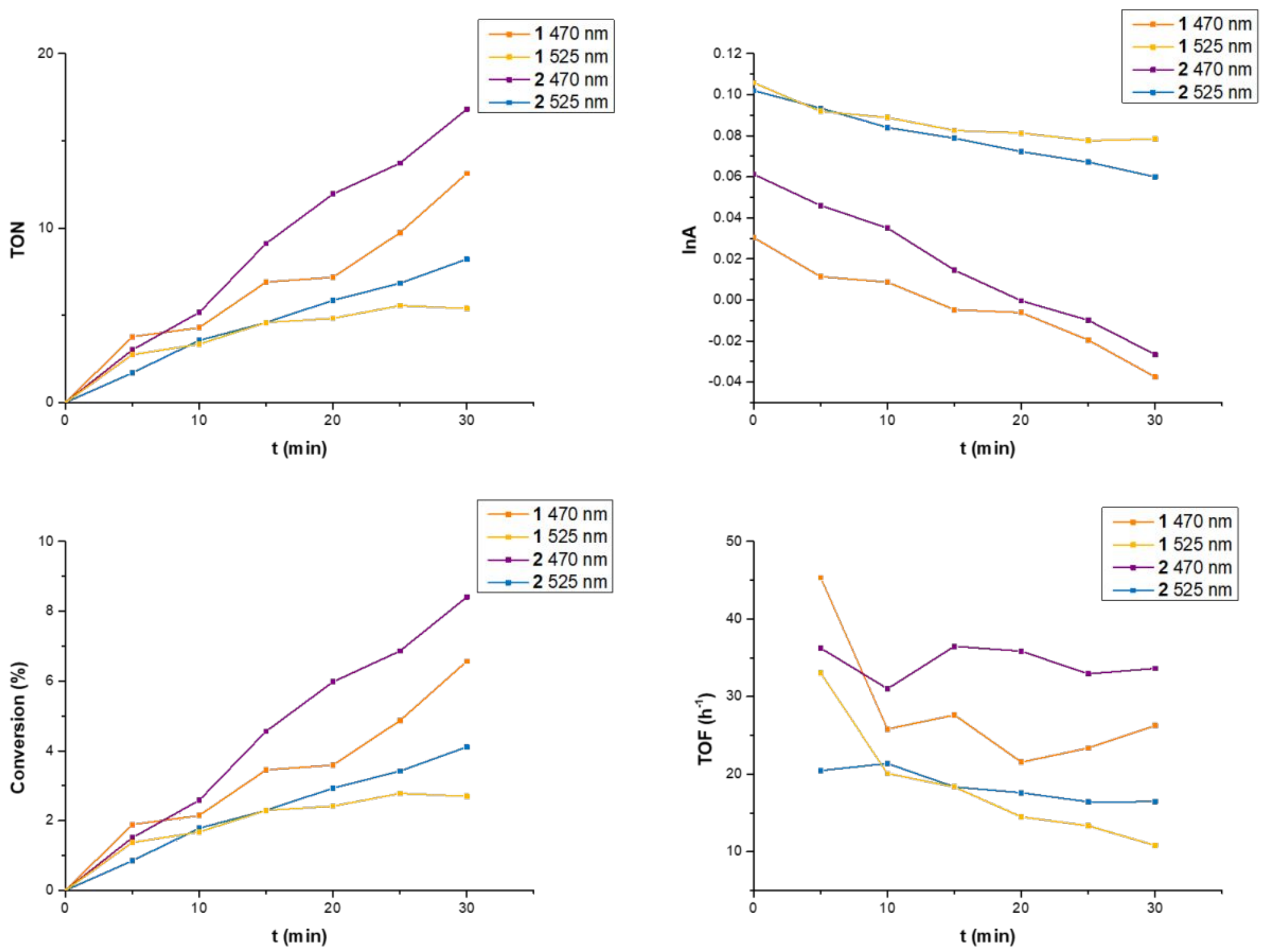

Figure S42. Compared plots of TON, lnA, conversion and TOF for complexes **1** and **2** (1 μM; 0.5 mol%) under irradiation at 470 and 525 nm.

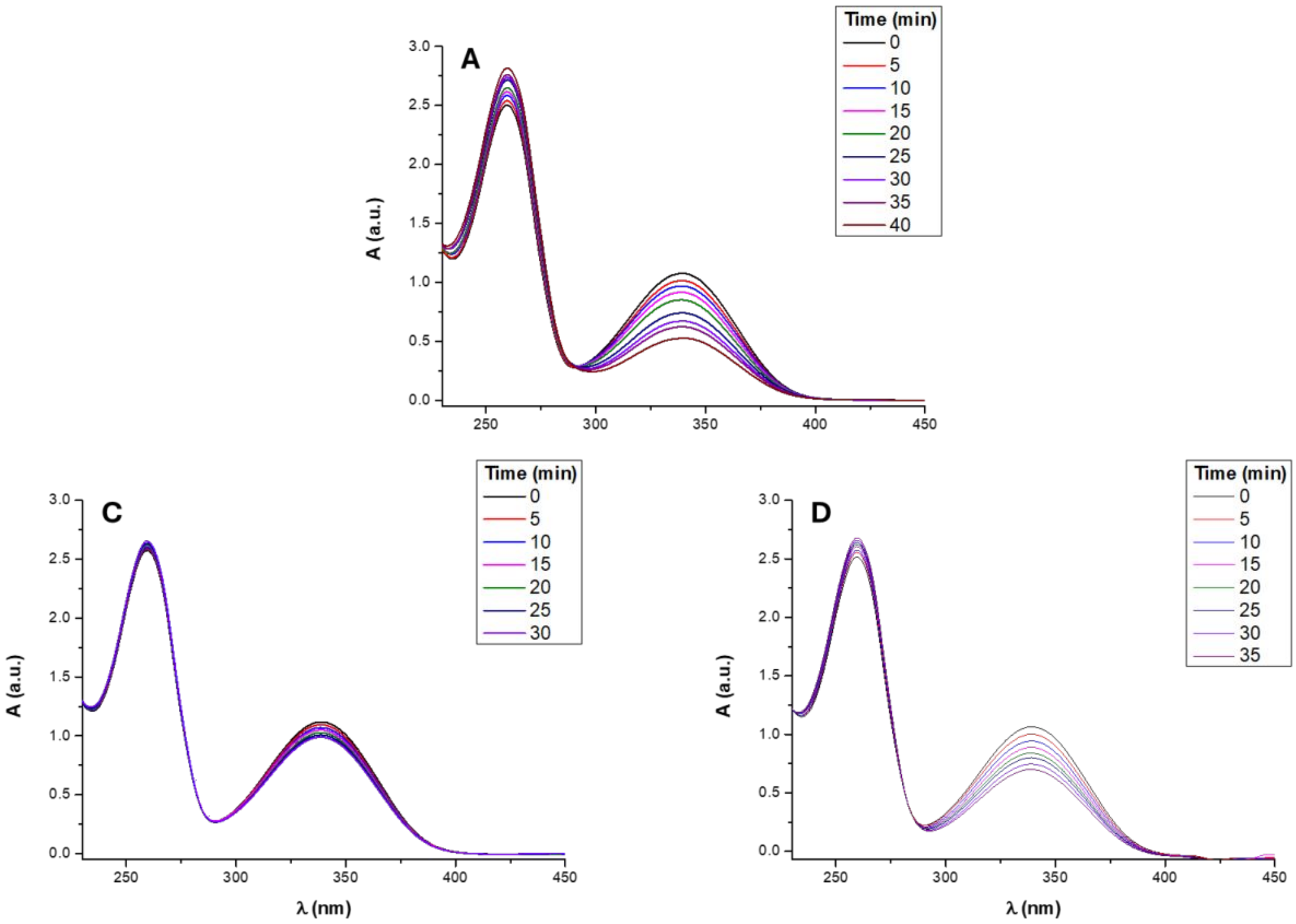

Figure S43. UV-vis spectrum of NADH in presence of catalyst (10 µM; 5 mol%) in different conditions: **A** complex **1** under 470 nm irradiation, **C** complex **1** under 525 nm irradiation, **D** complex **2** under 525 nm irradiation.

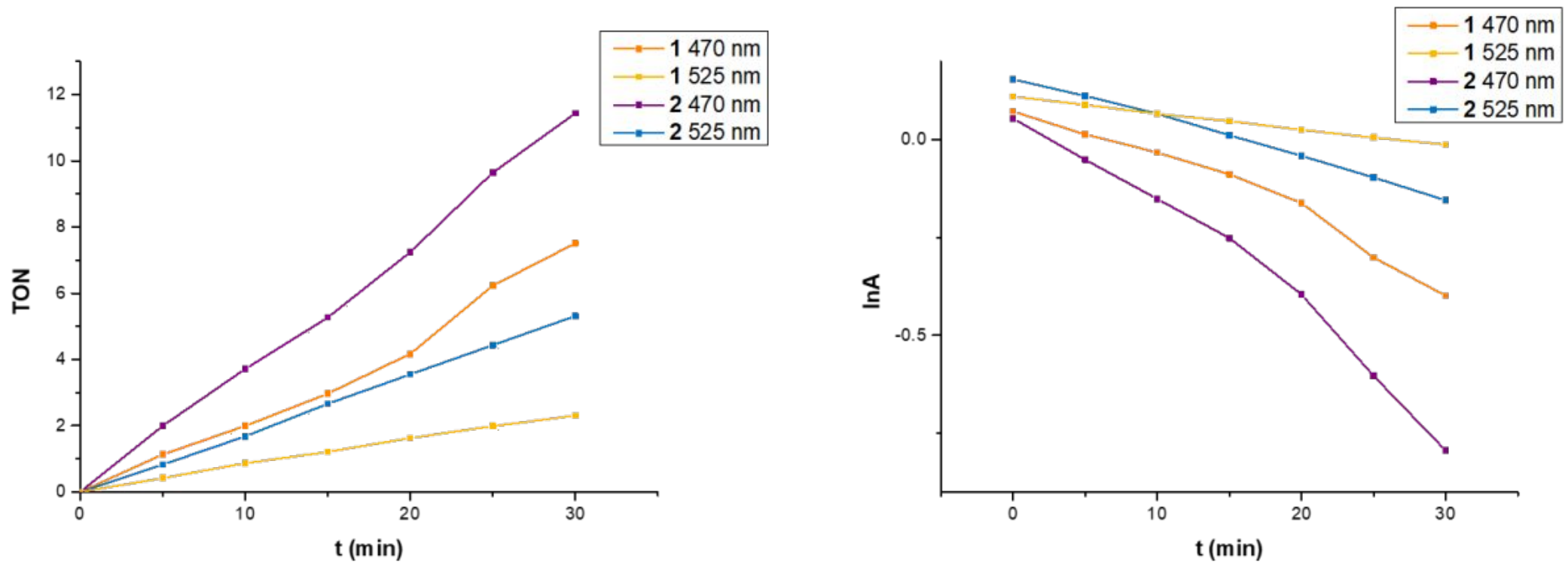

Figure S44. Compared plots of TON and lnA for complexes **1** and **2** (10 µM; 5 mol%) under irradiation at 470 and 525 nm.

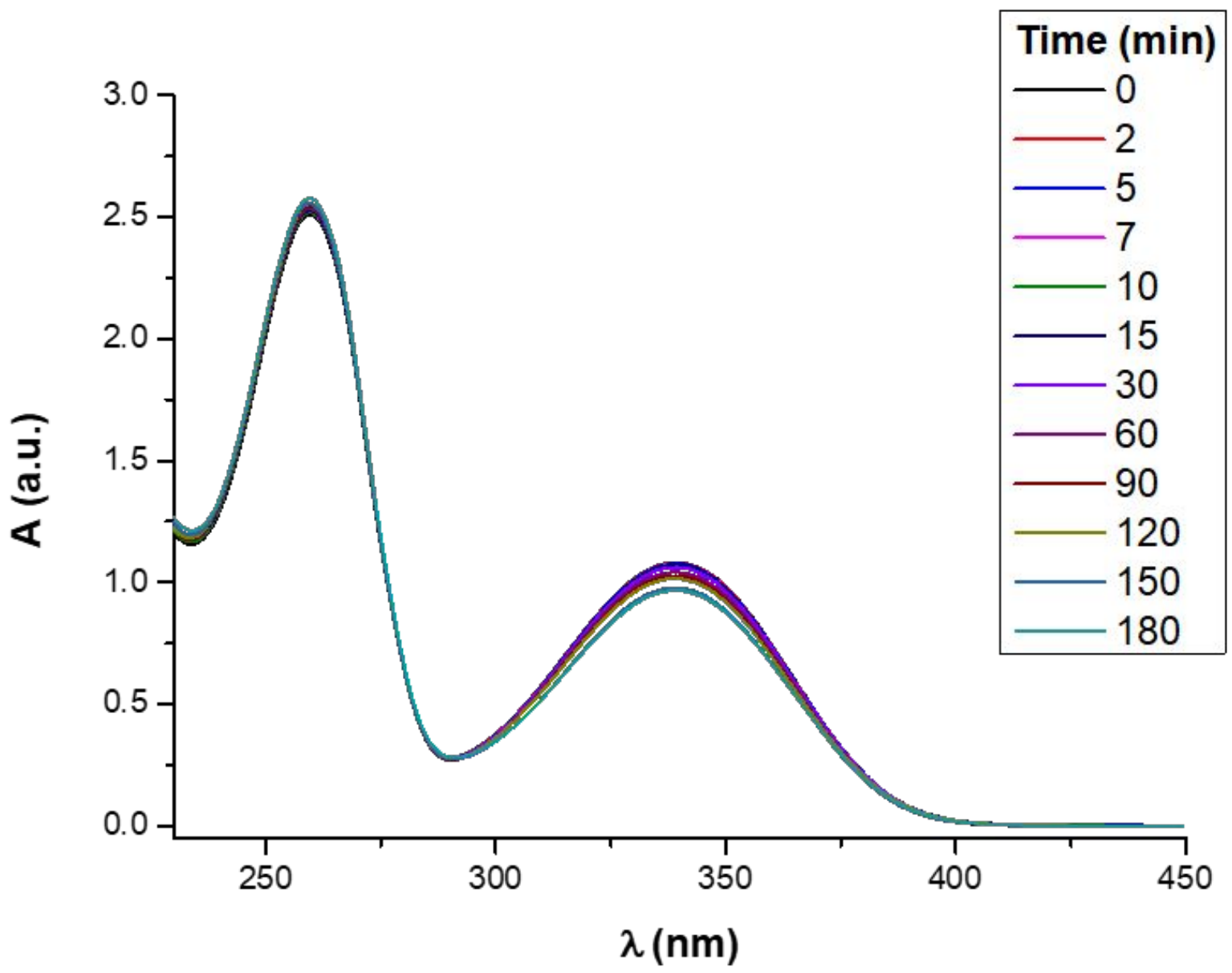

Figure S45. UV-vis spectrum of NADH in presence of catalyst **2** (0.1 μM; 0.05 mol%) under irradiation at 470 nm.

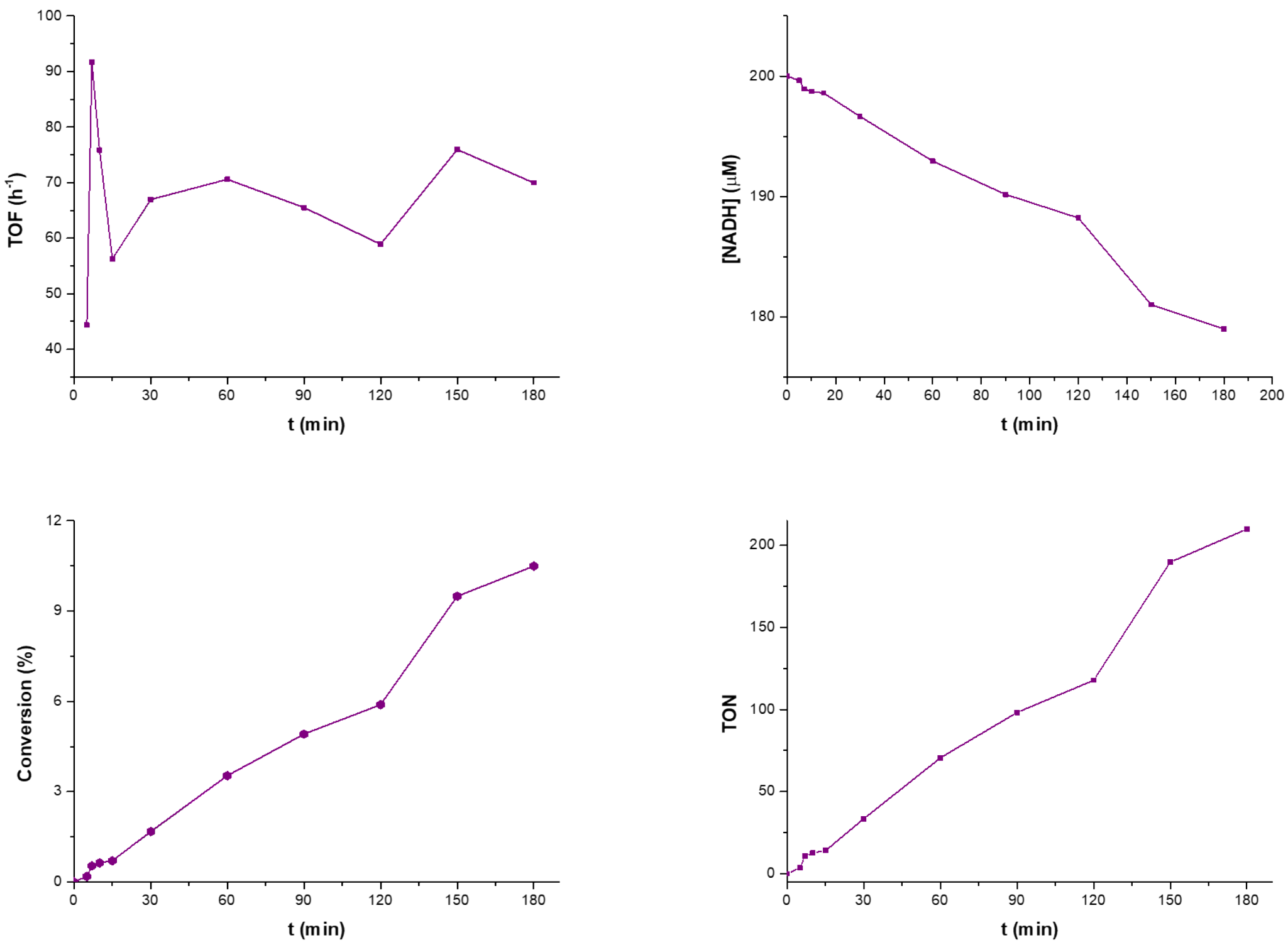

Figure S46. Plots of TOF, [NADH], conversion and TOF for complex **2** (0.1 μM; 0.05 mol%) under irradiation at 470 nm.

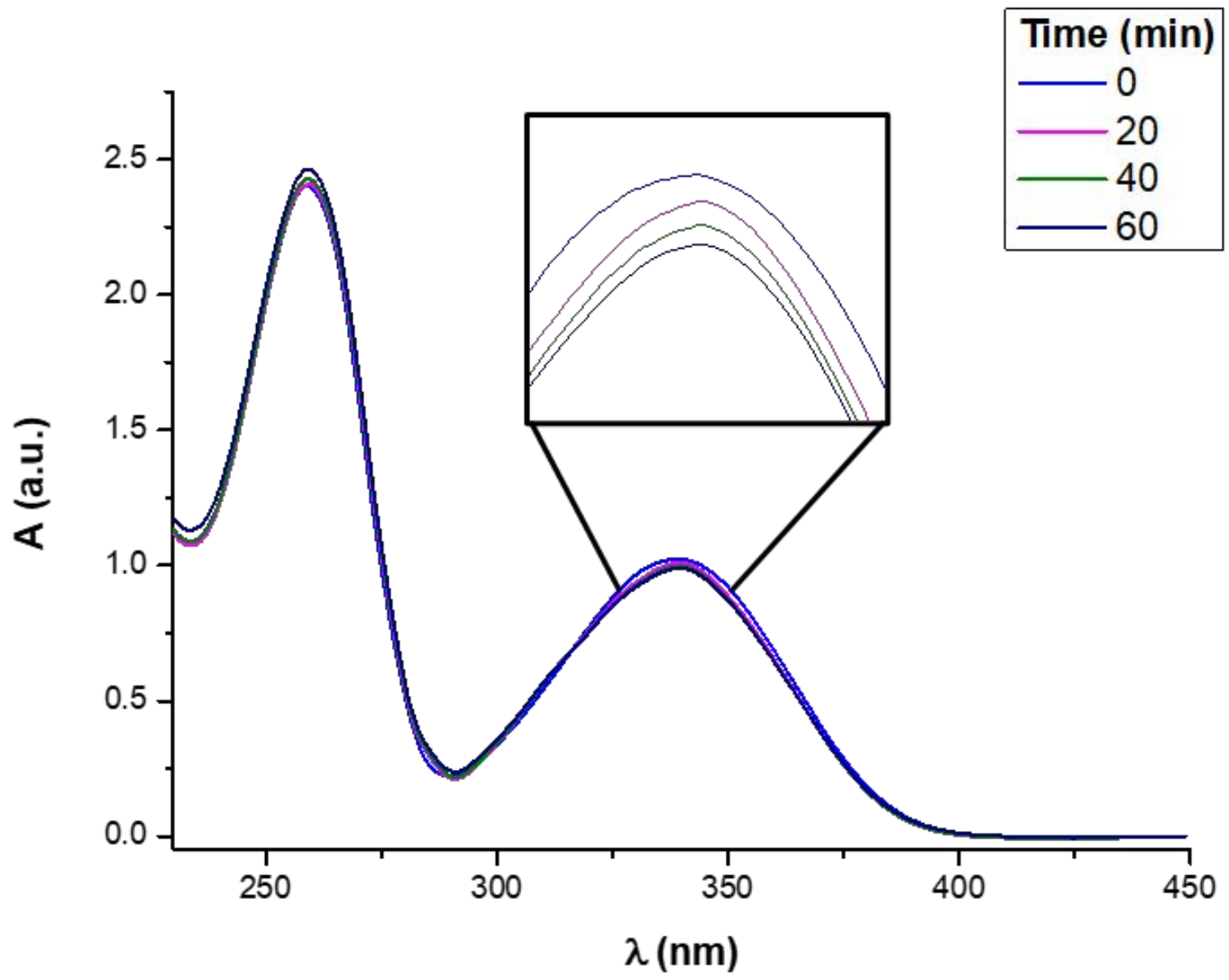

Figure S47. UV-vis spectrum of NADH in presence of catalyst **2** (60 µM; 30 mol%) under irradiation at 600 nm.

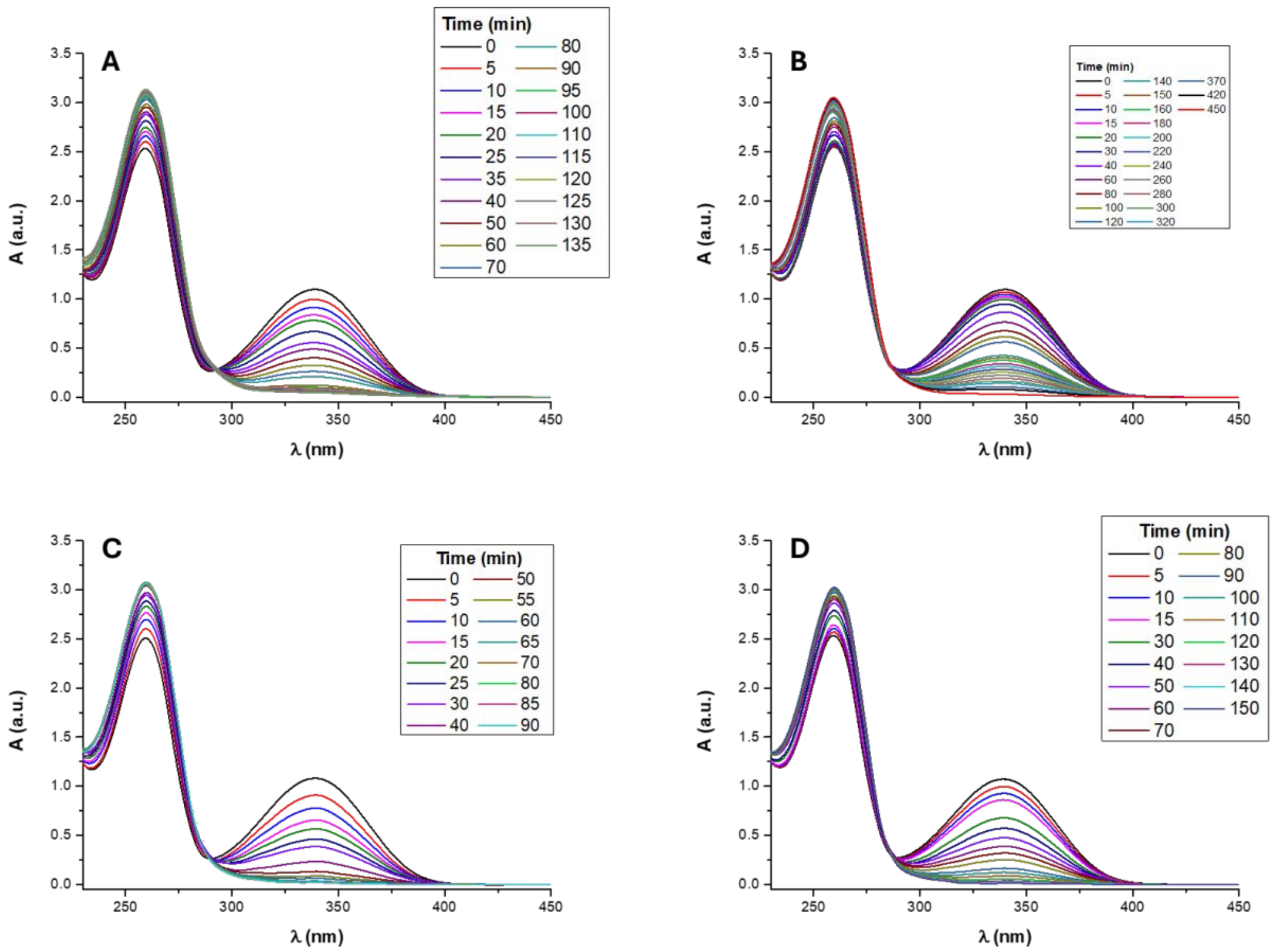

Figure S48. UV-vis of NADH in presence of complexes **1** and **2** (20 µM; 10 mol%) until complete conversion (>95%) under irradiation at 470 and 525 nm. **A** complex **1** under 470 nm irradiation, **B** complex **1** under 525 nm irradiation, **C** complex **2** under 470 nm irradiation, **D** complex **2** under 525 nm irradiation.

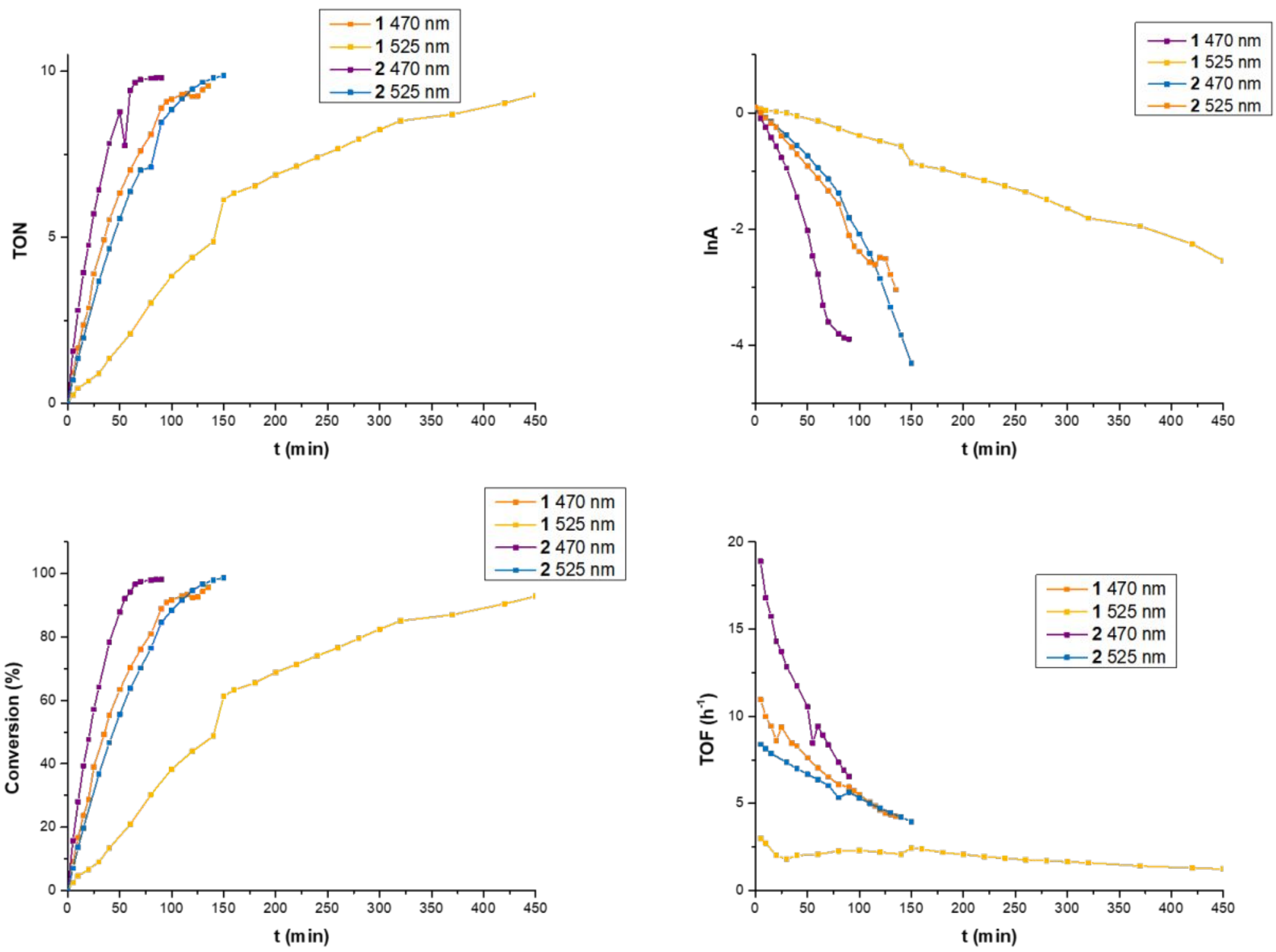

Figure S49. Compared plots of TON, lnA, conversion and TOF for complexes **1** and **2** (20 μM; 10 mol%) until complete conversion (>95%) under irradiation at 470 and 525 nm.

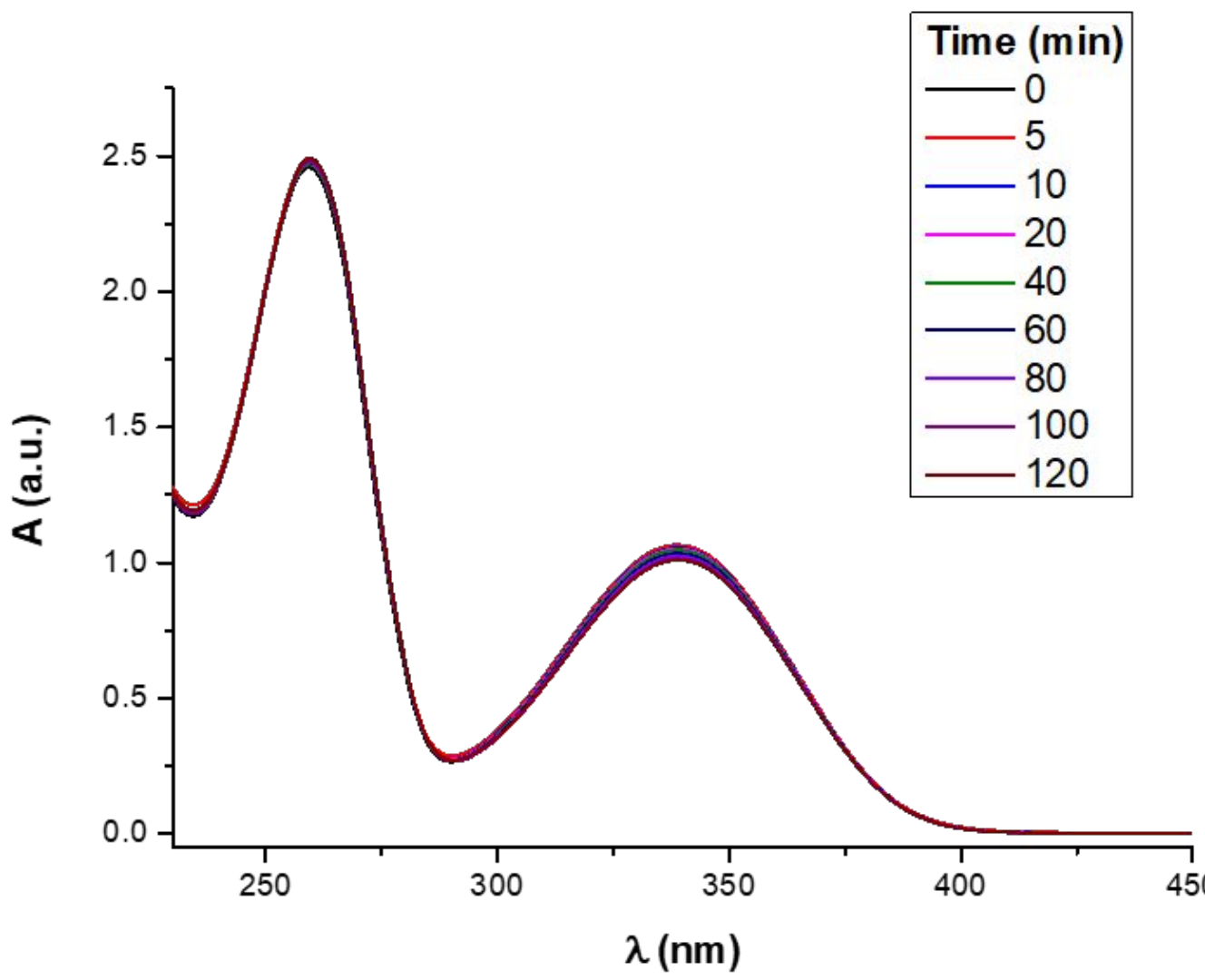

Figure S50. UV-vis spectrum of NADH under irradiation at 470 nm.

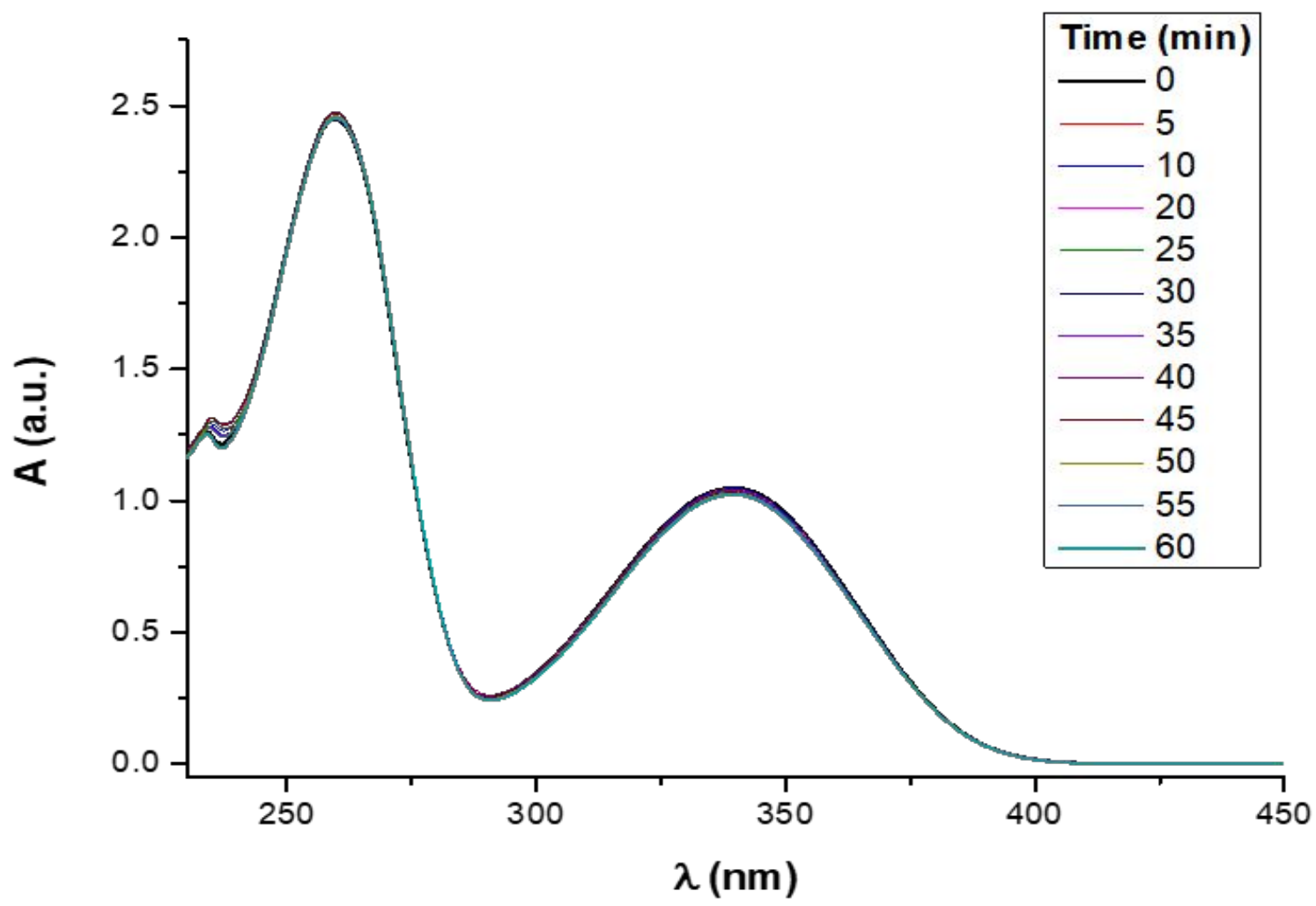

Figure S51. UV-vis spectrum of NADH in presence of ligand **L** (20 µM; 10 mol%) under irradiation at 470 nm.

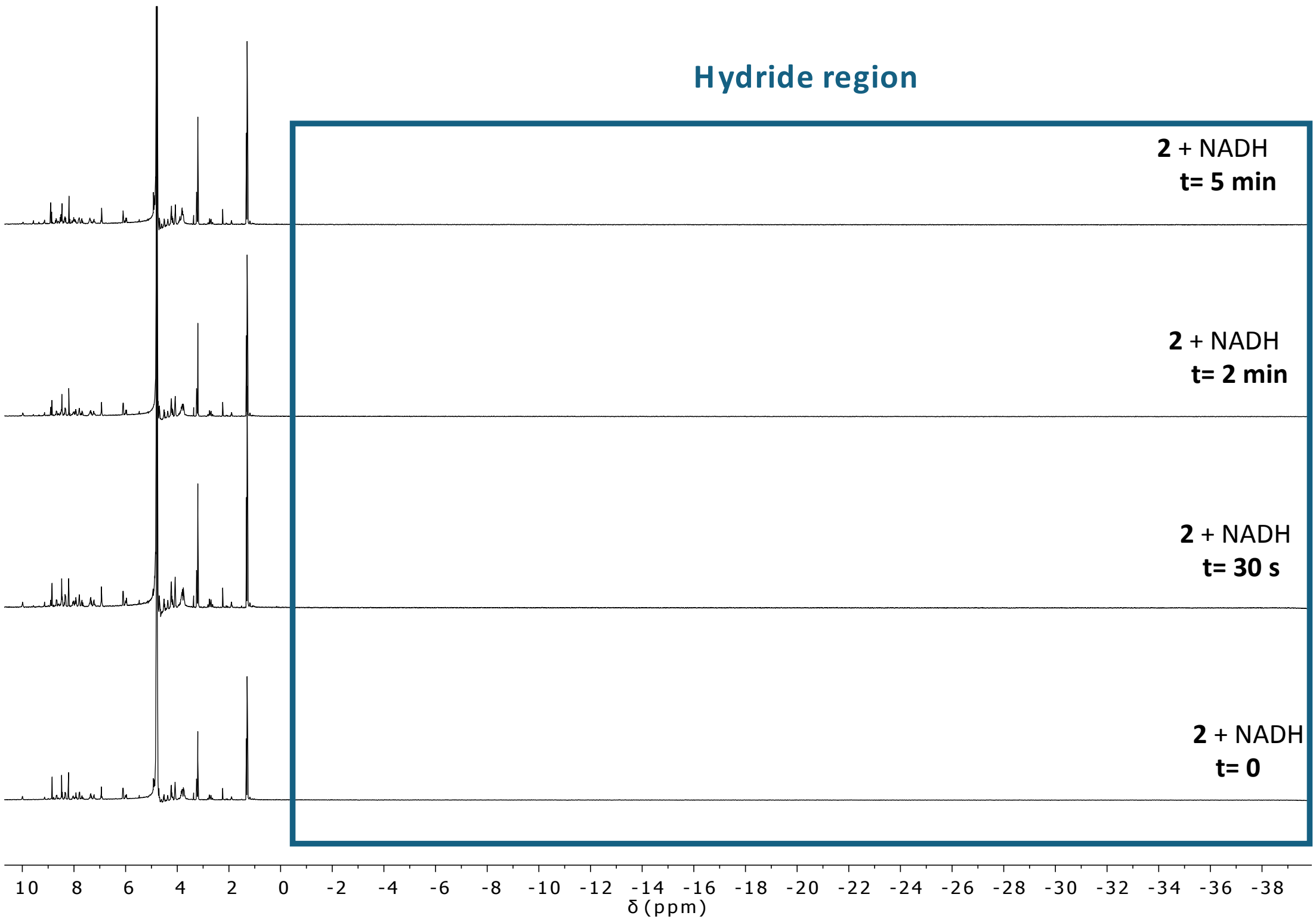

Figure S52. NMR spectra of complex **2** (5 mM) in presence of NADH (5 mM) in D_2O. The hydride region is marked in blue, showing no hydride formation during the catalytic reaction.

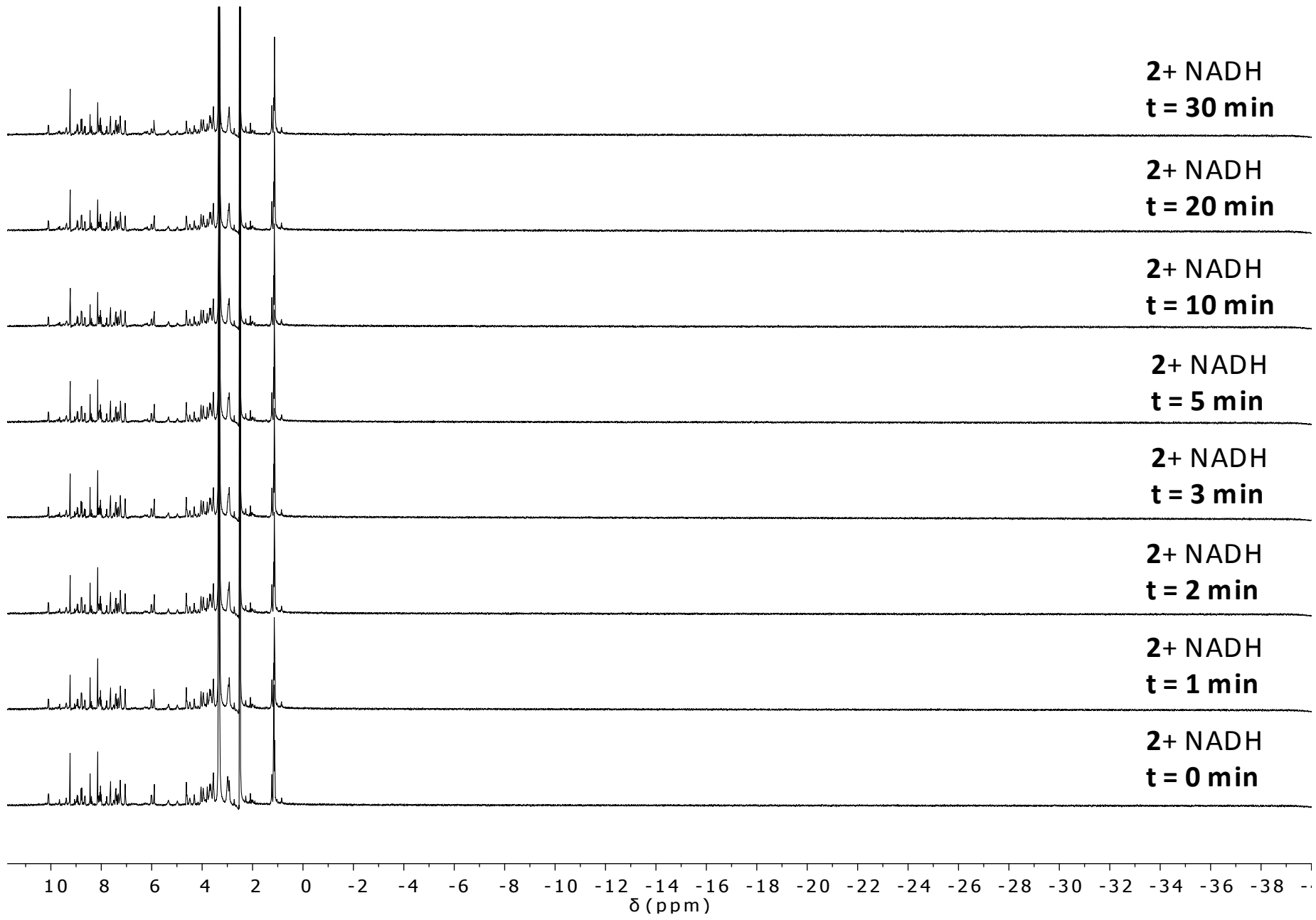

Figure S53. NMR spectra of complex **2** (5 mM) in presence of NADH (5 mM) in DMSO-d_6 and under argon atmosphere.

6.1.2. Mechanistic studies

6.1.2.1. H_2O_2 generation

H_2O_2 generation was detected by Quantofix peroxide test sticks (Sigma-Aldrich) at different irradiation times.

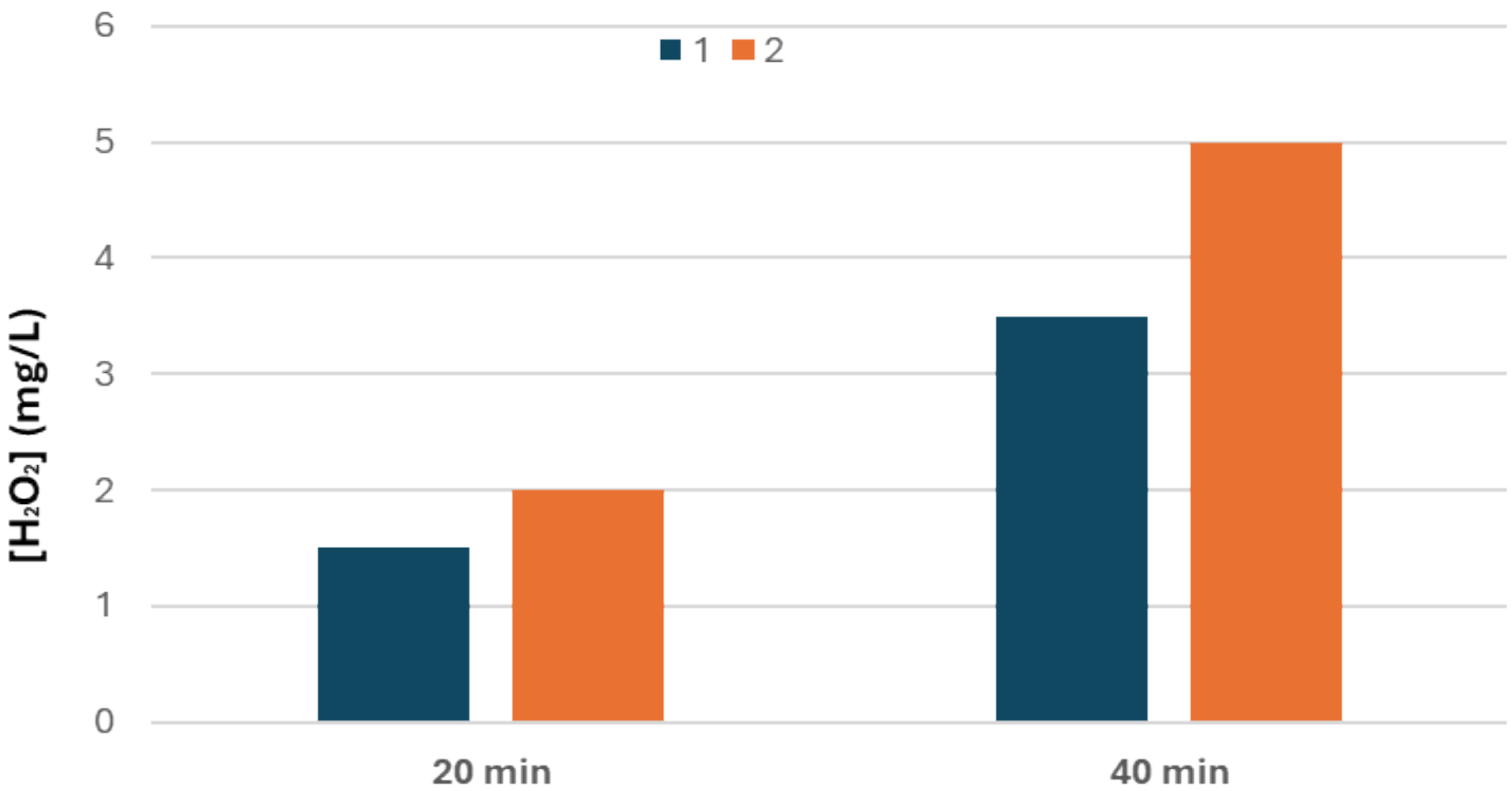

Figure S54. H_2O_2 generation for complexes **1** (blue) and **2** (orange) under irradiation at 470 nm.

6.1.2.2. Influence of O_2

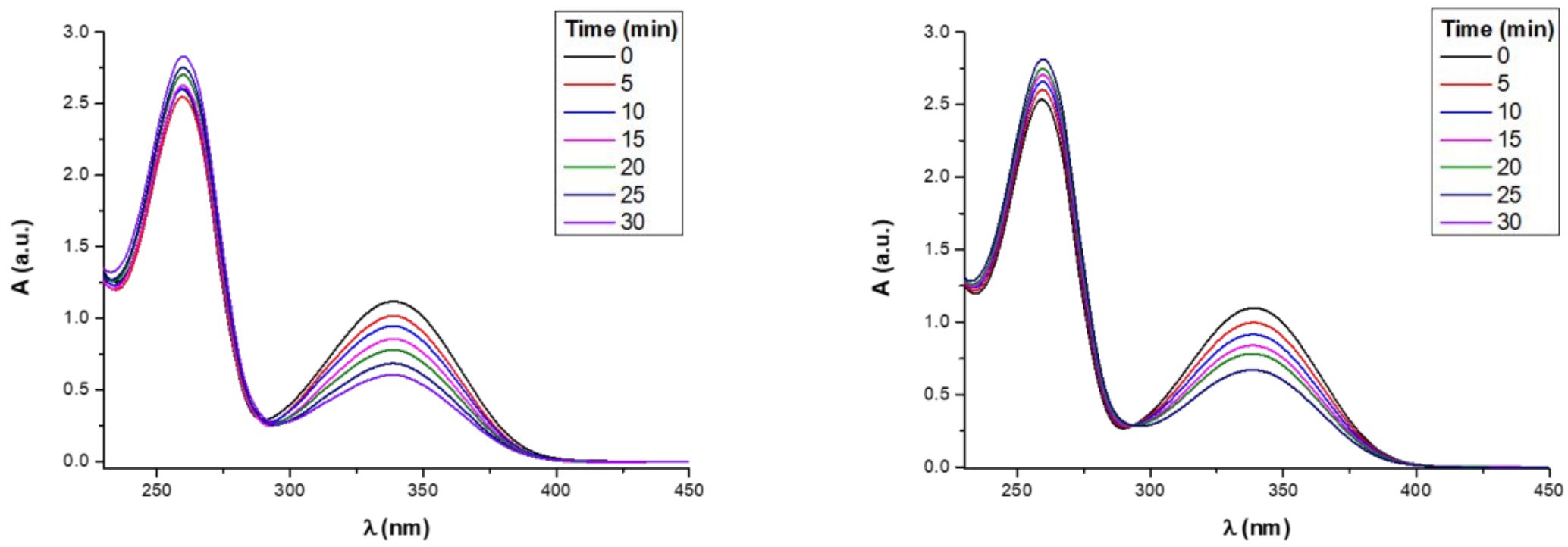

Figure S55. UV-vis spectrum of NADH in presence of complex **2** (20 μM; 10 mol%) under irradiation at 470 nm in air (right) and under Ar atmosphere (left).

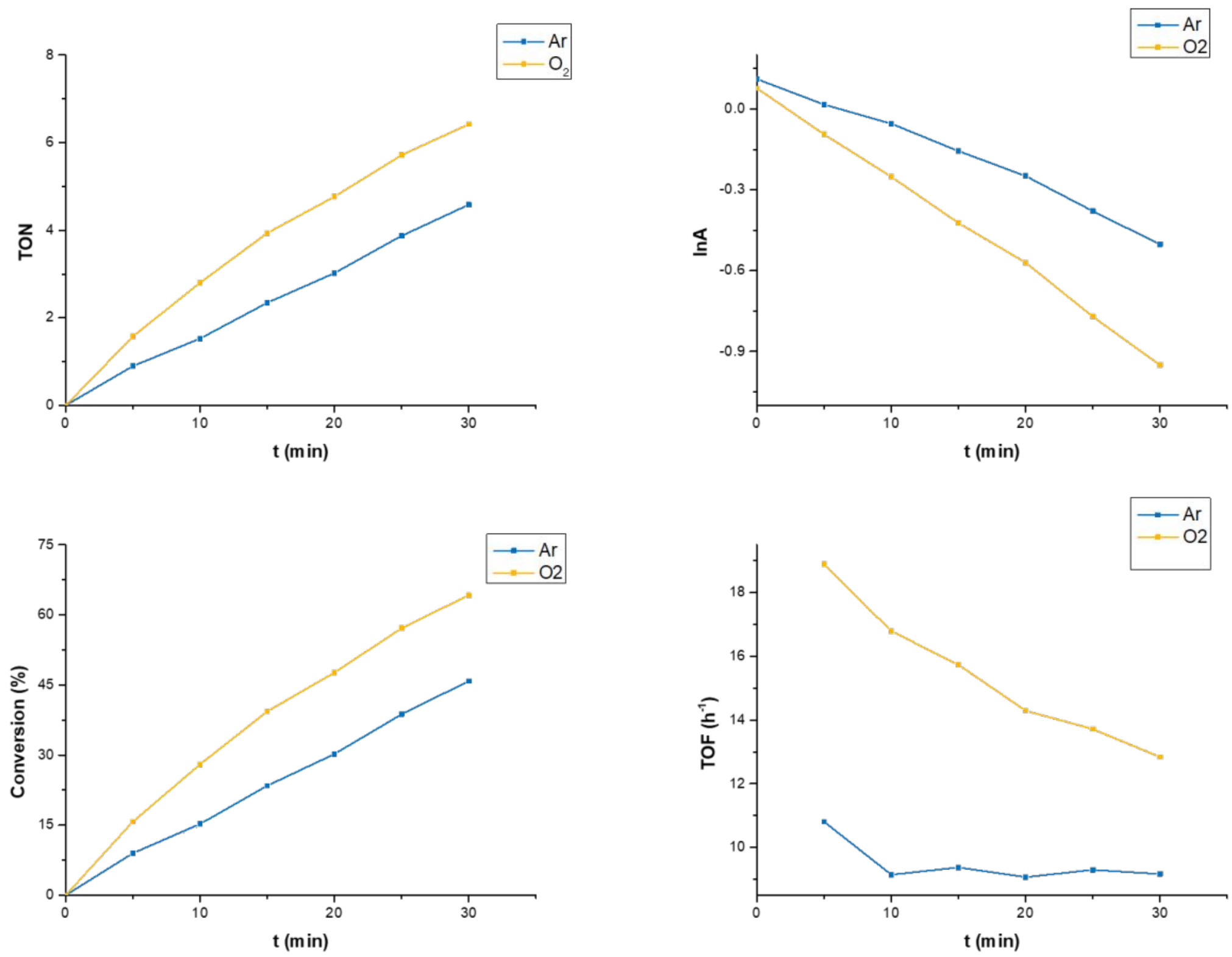

Figure S56. Compared plots of TON, lnA, conversion and TOF for complex **2** under irradiation at 470 nm in air and under Ar atmosphere.

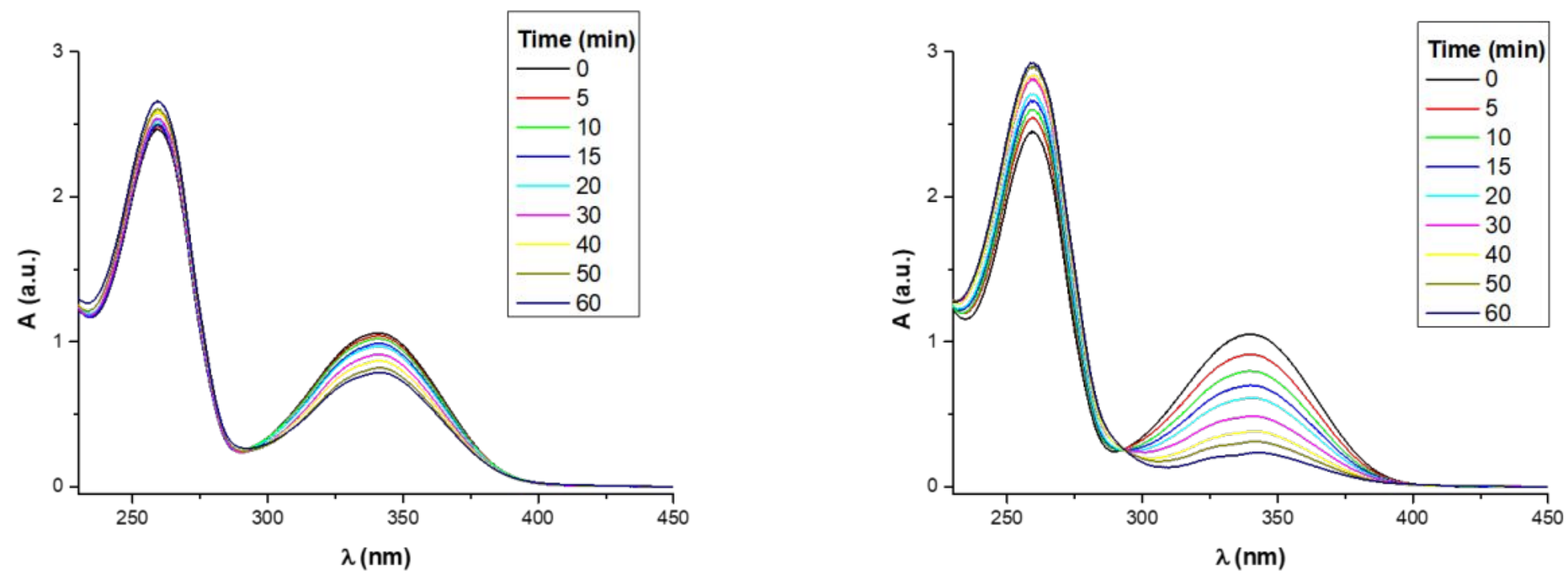

Figure S57. UV-vis spectrum of NADH in presence of complex **1** (20 µM; 10 mol%) under irradiation at 470 nm in air (right) and under Ar atmosphere (left).

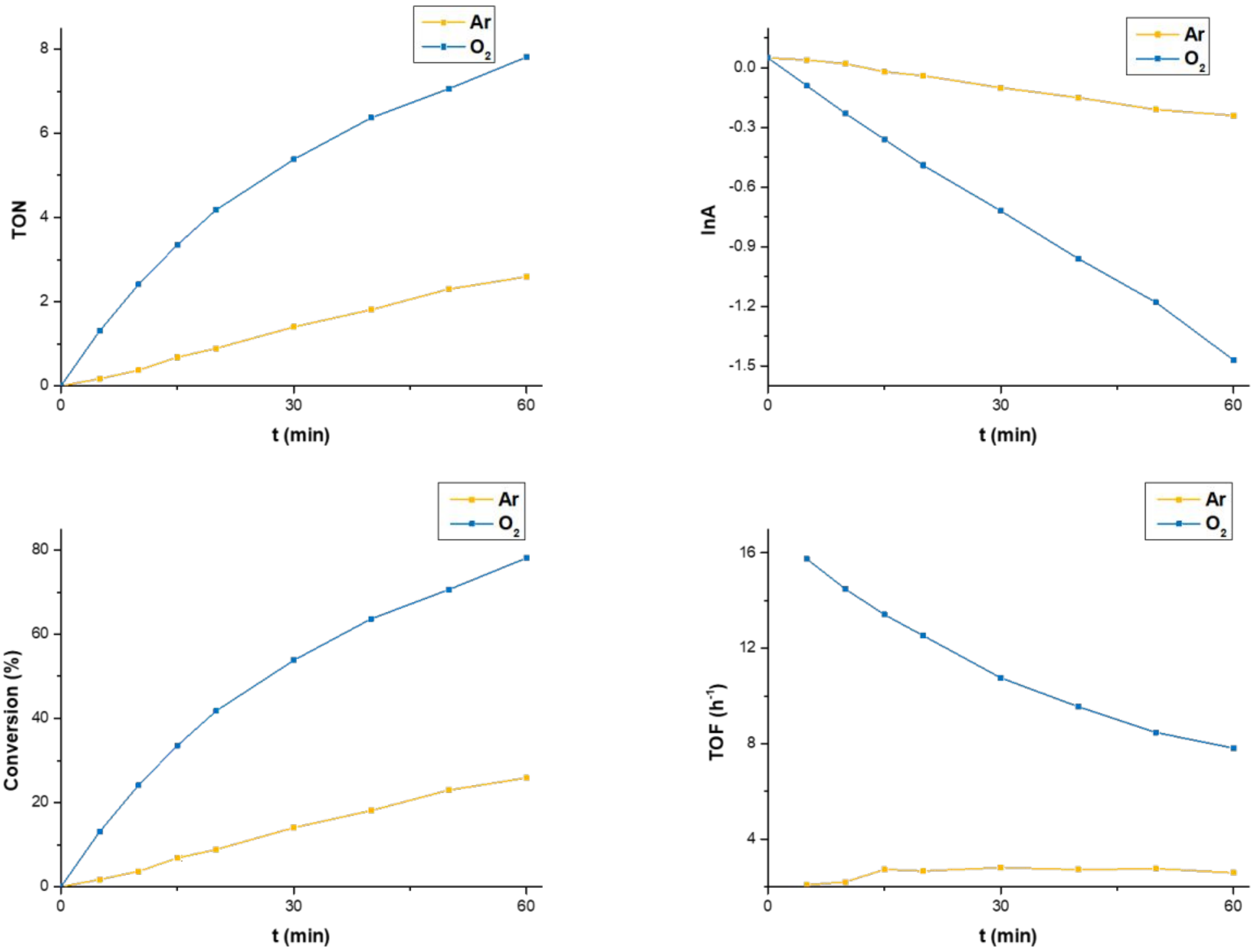

Figure S58. Compared plots of TON, lnA, conversion and TOF for complex **1** under irradiation at 470 nm in air and under Ar atmosphere.

6.1.2.3. <u>ROS scavengers</u>

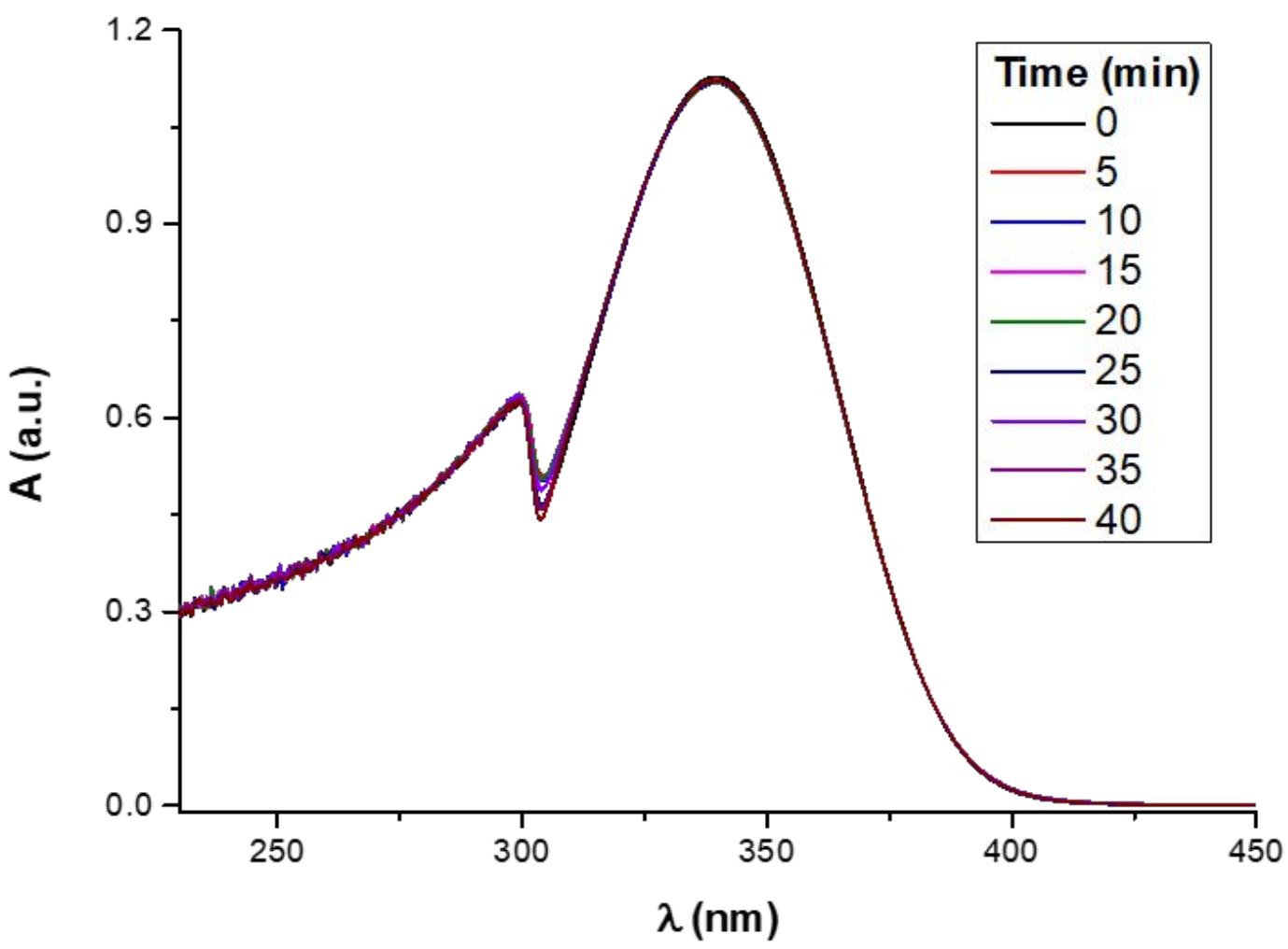

Figure S59. UV-vis spectrum of NADH in presence of complex **1** (10 µM; 5 mol%) and 10 mM sodium ascorbate under irradiation at 470 nm.

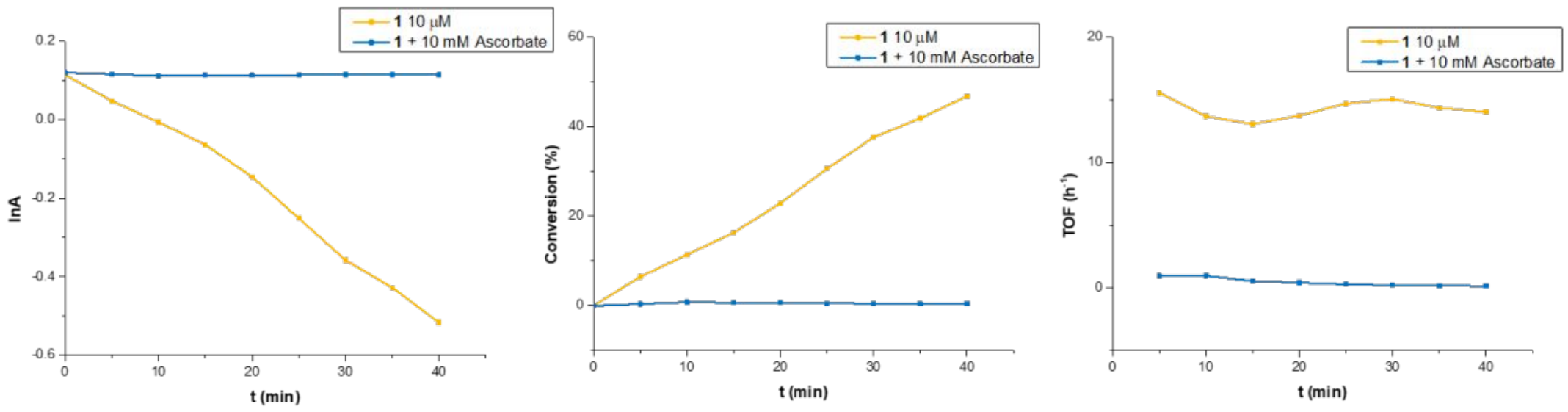

Figure S60. Compared plots of lnA, conversion and TOF for complex **1** (10 μM; 5 mol%) and 10 mM sodium ascorbate under irradiation at 470 nm.

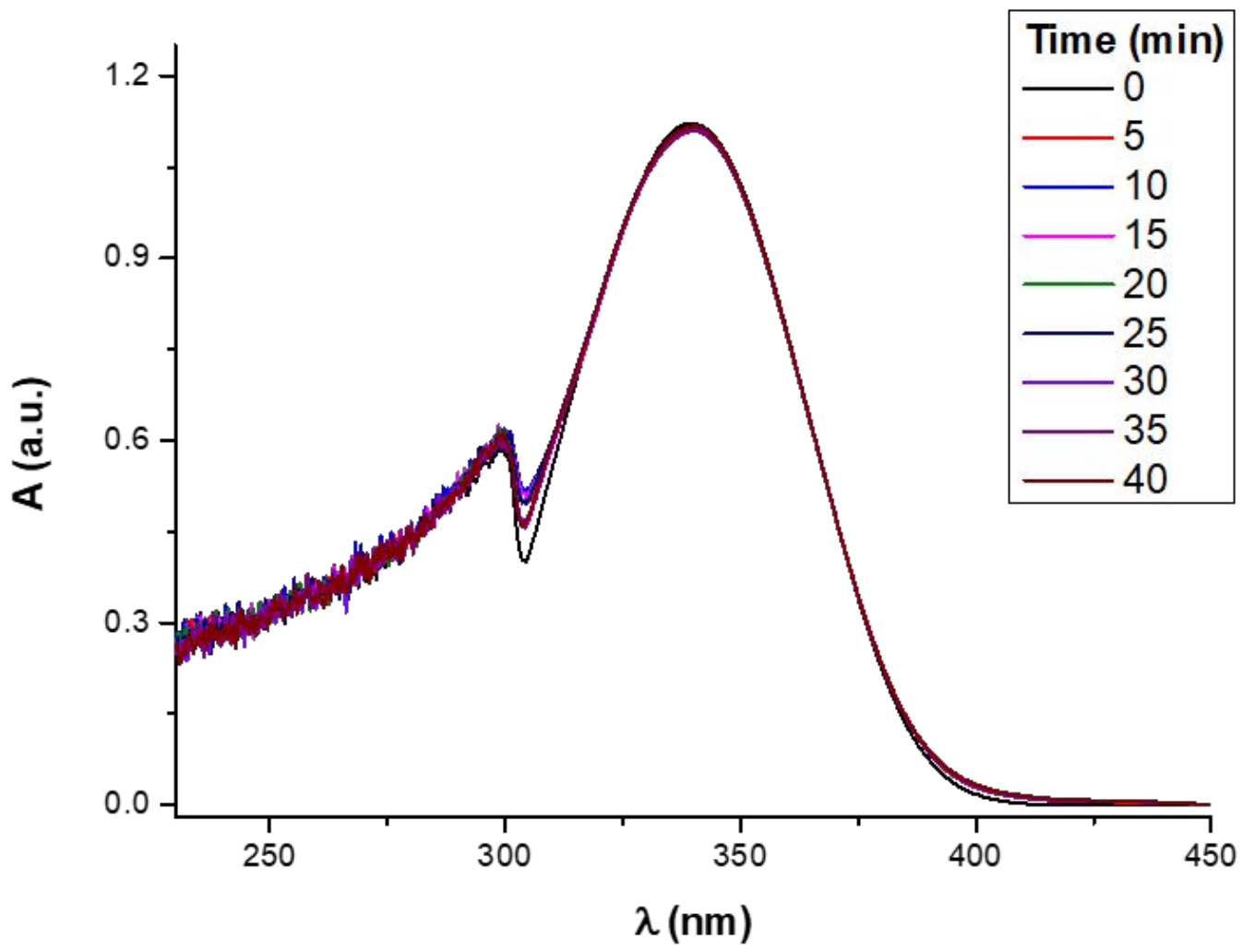

Figure S61. UV-vis spectrum of NADH in presence of complex **2** (10 μM; 5 mol%) and 10 mM sodium ascorbate under irradiation at 470 nm.

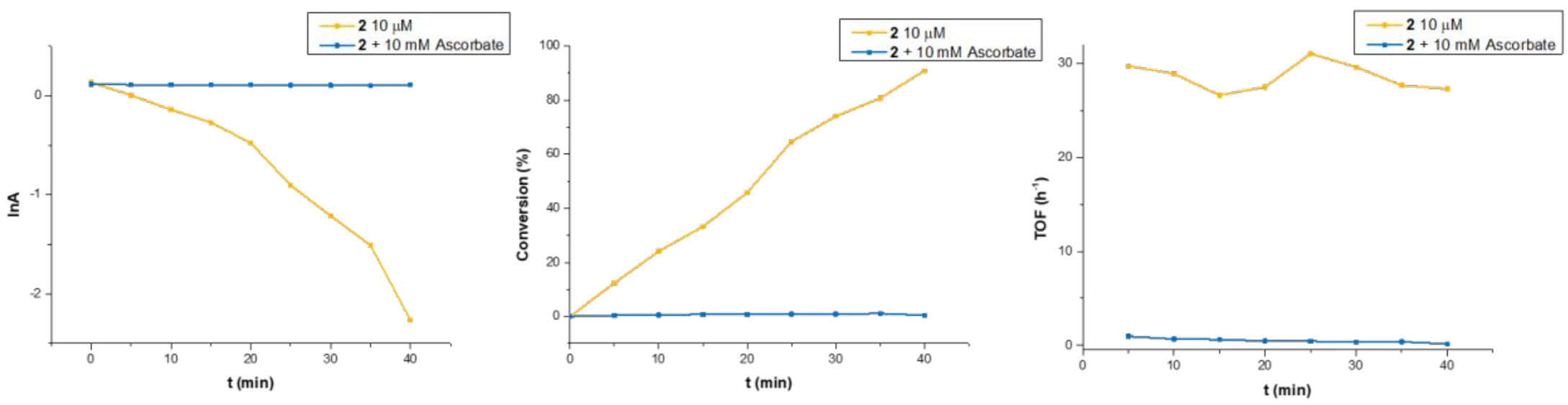

Figure S62. Compared plots of lnA, conversion and TOF for complex **2** (10 μM; 5 mol%) and 10 mM sodium ascorbate under irradiation at 470 nm.

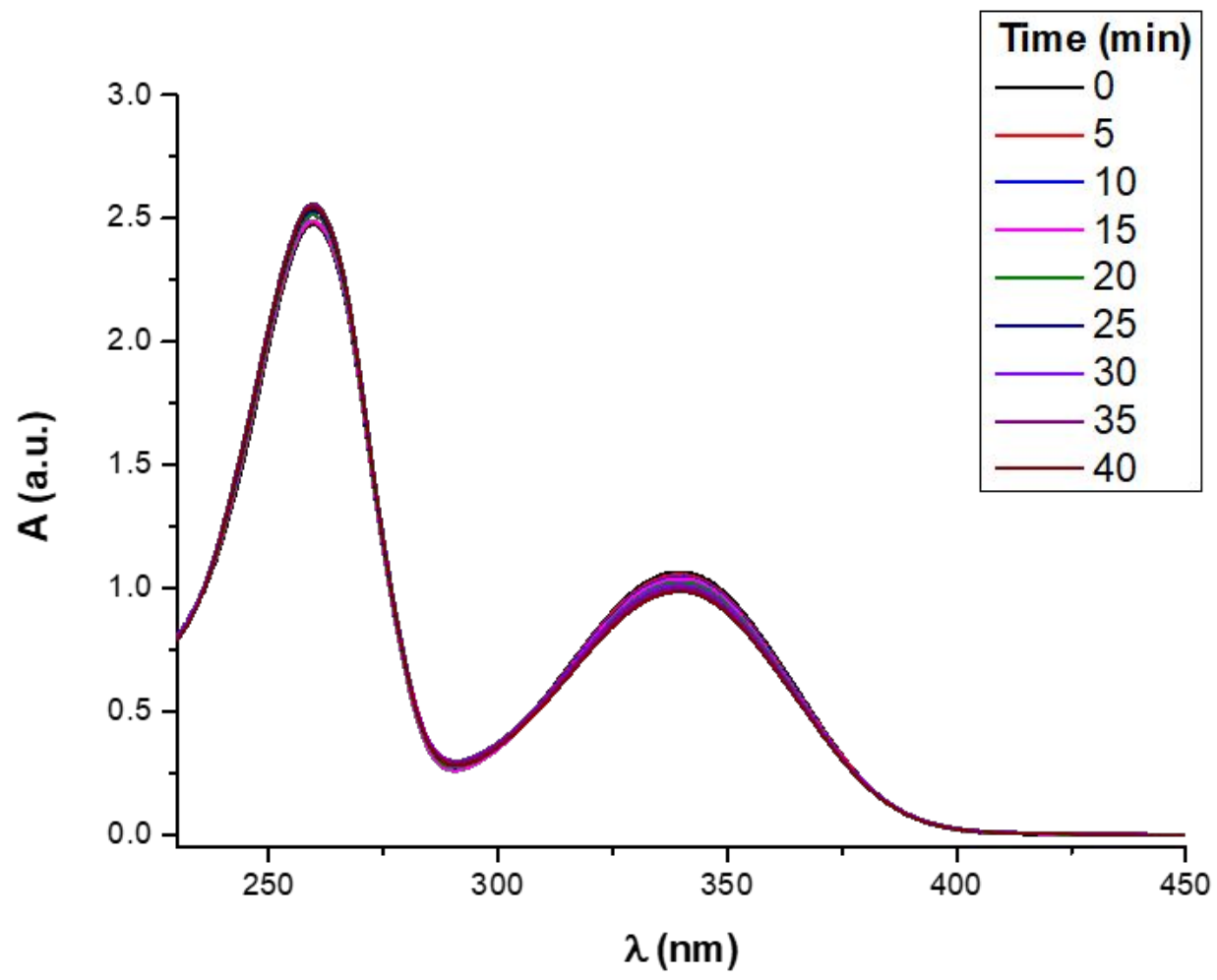

Figure S63. UV-vis spectrum of NADH in presence of complex **1** (10 μM; 5 mol%) and 10 mM sodium azide under irradiation at 470 nm.

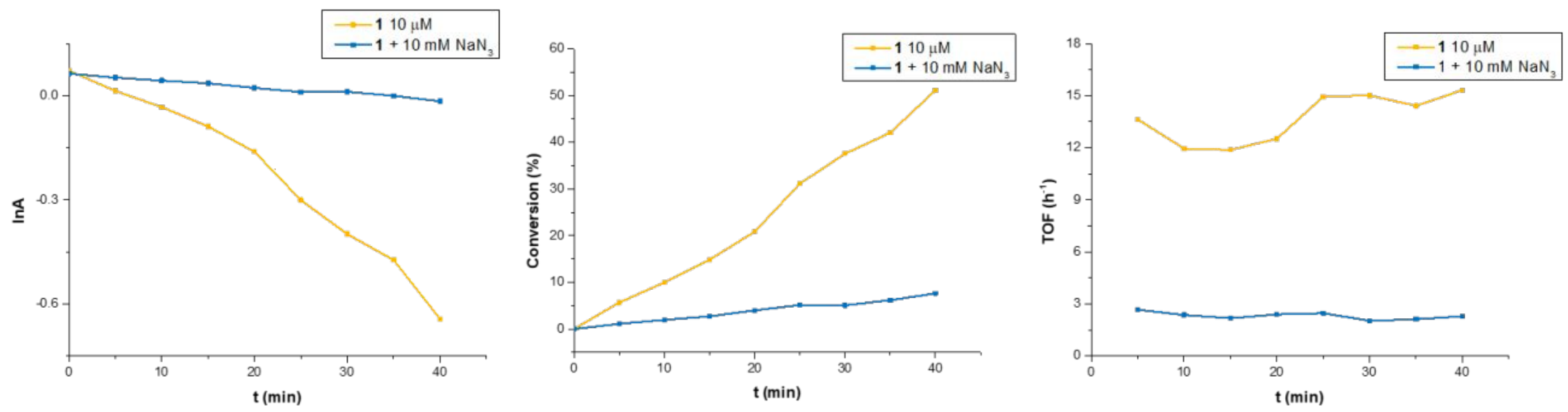

Figure S64. Compared plots of lnA, conversion and TOF for complex **1** (10 μM; 5 mol%) and 10 mM sodium azide under irradiation at 470 nm.

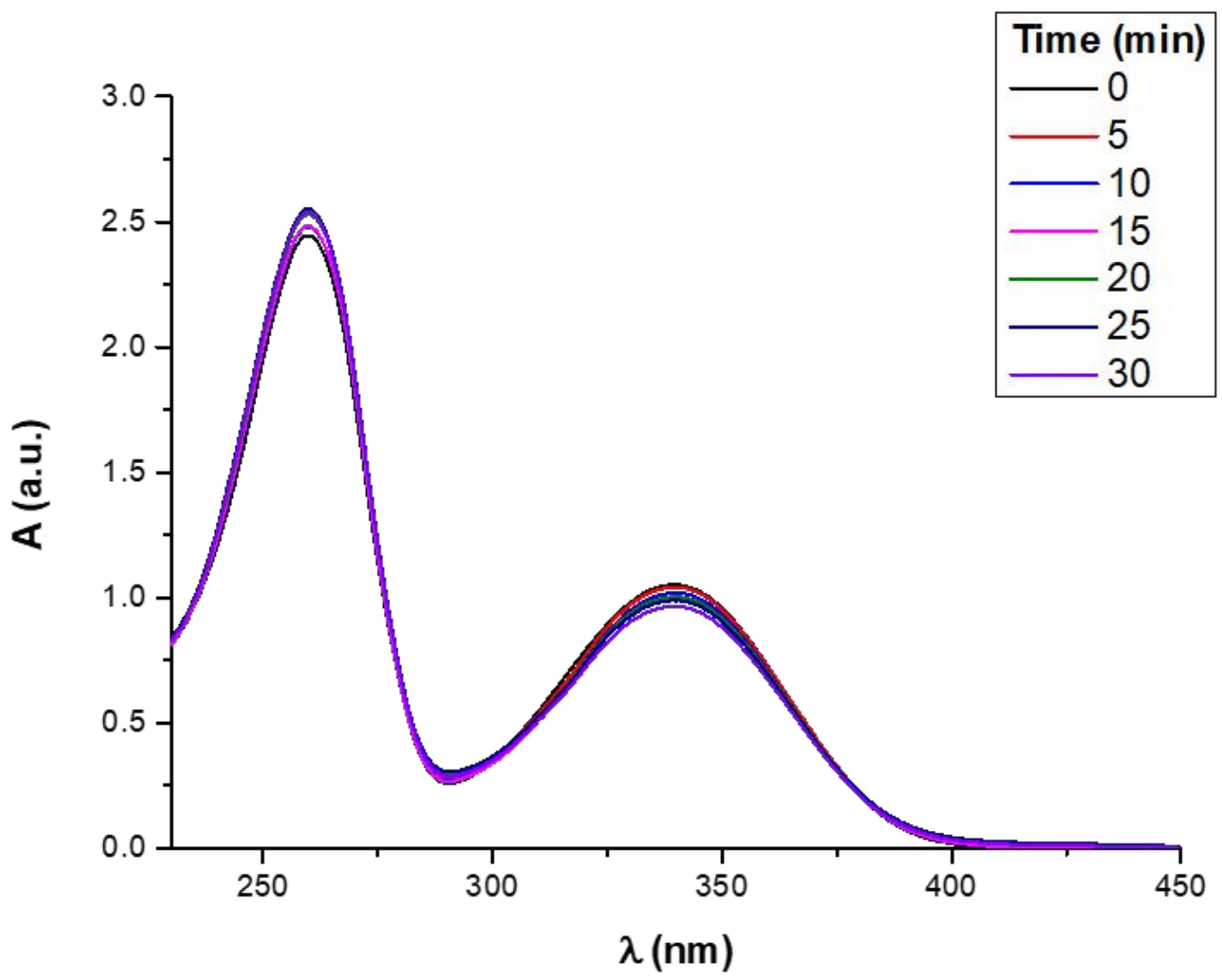

Figure S65. UV-vis spectrum of NADH in presence of complex **2** (10 μM; 5 mol%) and 10 mM sodium azide under irradiation at 470 nm.

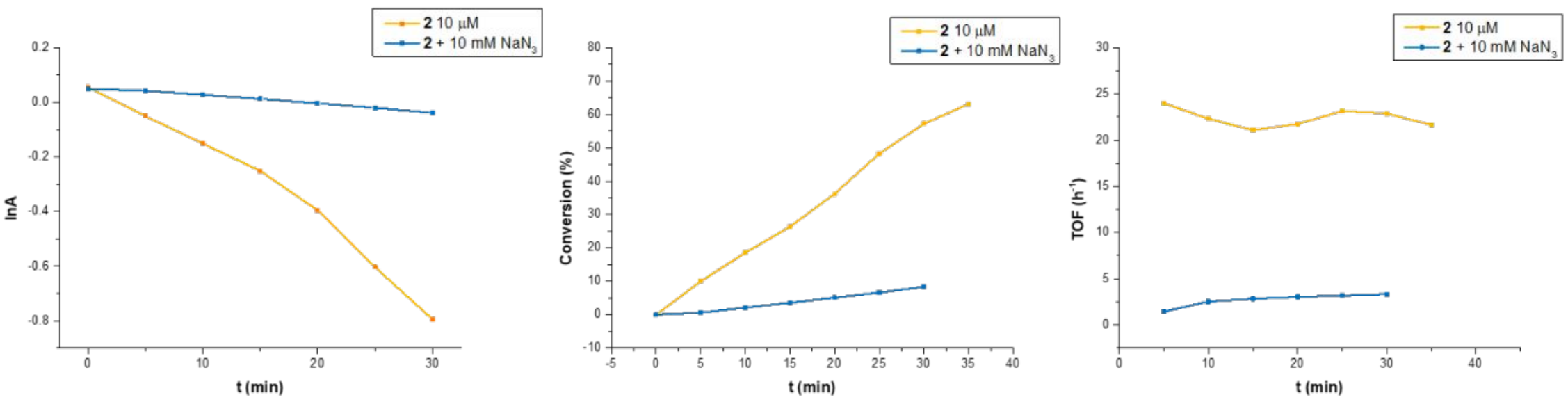

Figure S66. Compared plots of lnA, conversion and TOF for complex **2** (10 µM; 5 mol%) and 10 mM sodium azide under irradiation at 470 nm.

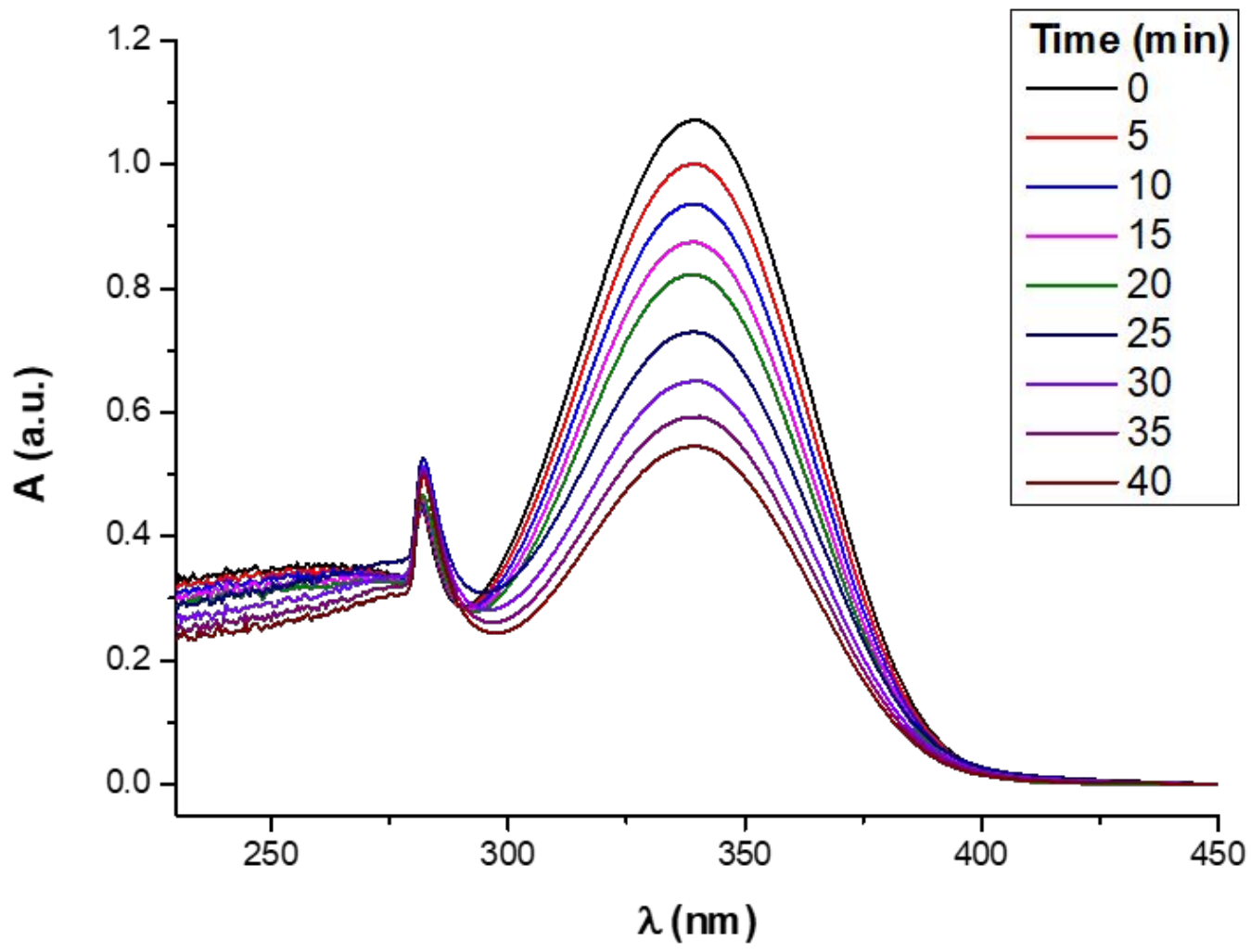

Figure S67. UV-vis spectrum of NADH in presence of complex **1** (10 µM; 5 mol%) and 15 mM sodium benzoate under irradiation at 470 nm.

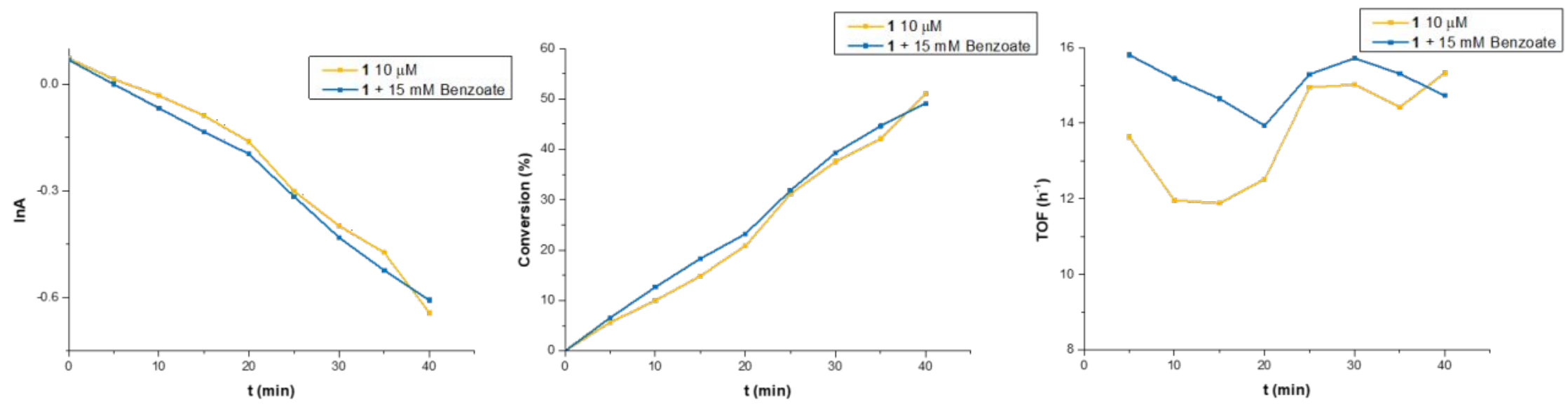

Figure S68. Compared plots of lnA, conversion and TOF for complex **1** (10 µM; 5 mol%) and 15 mM sodium benzoate under irradiation at 470 nm.

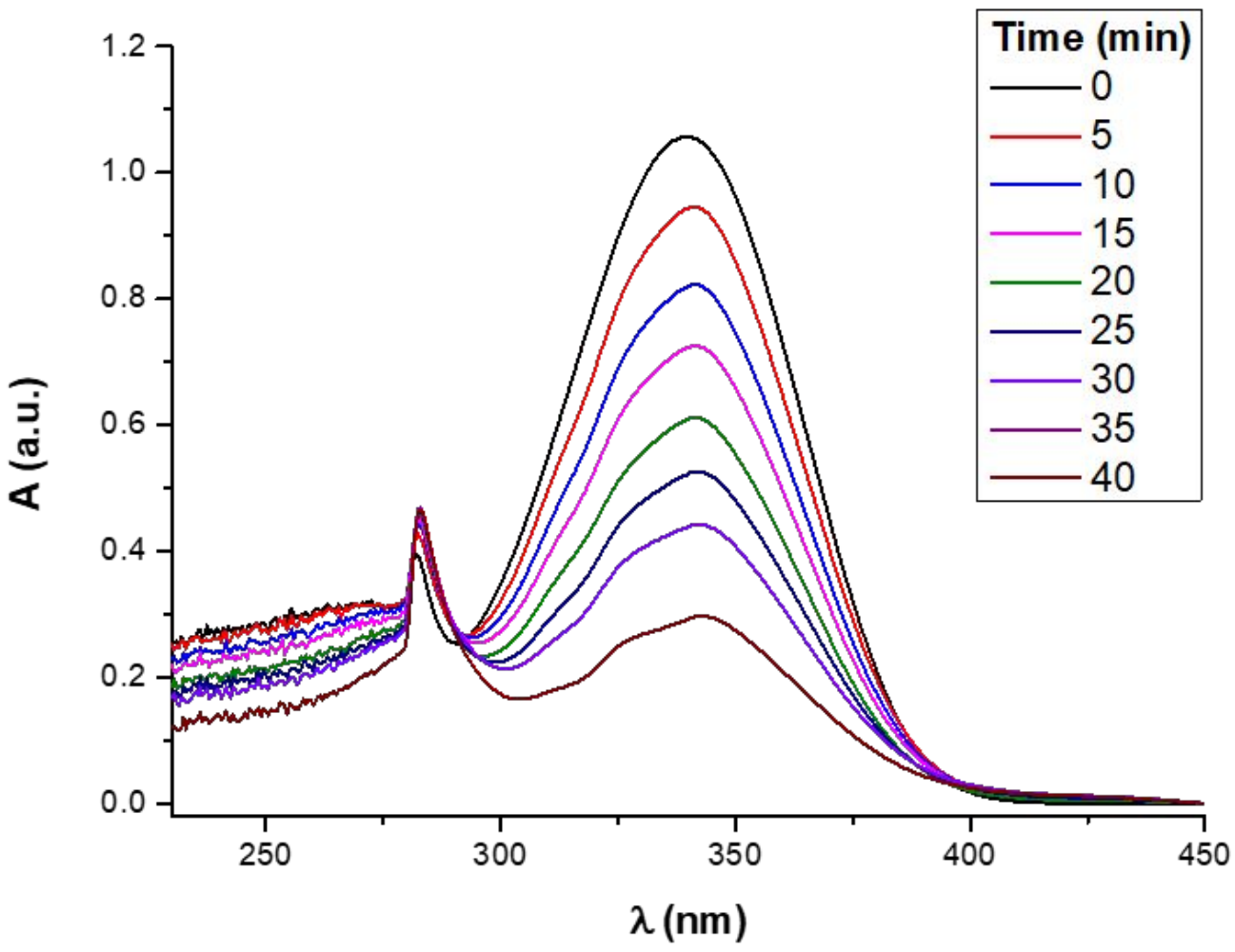

Figure S69. UV-vis spectrum of NADH in presence of complex **2** (10 µM; 5 mol%) and 15 mM sodium benzoate under irradiation at 470 nm.

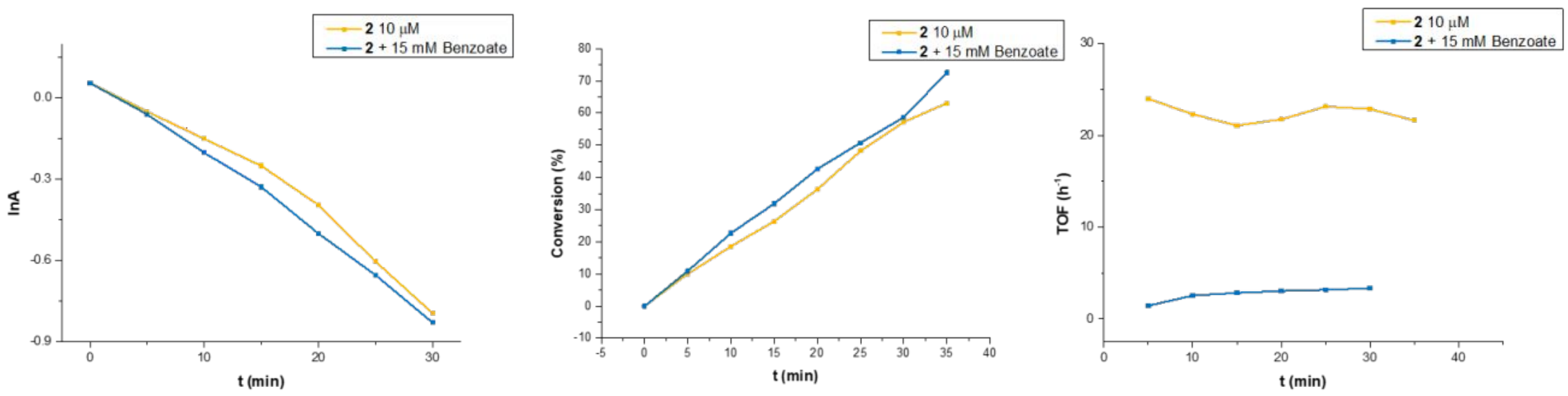

Figure S70. compared plots of lnA, conversion and TOF for complex **2** (10 µM; 5 mol%) and 15 mM sodium benzoate under irradiation at 470 nm.

6.1.2.1. Monitoring of Superoxide generation during photocatalysis using DHE

Catalysts **1** and **2** (20 µM) were mixed with NADH (200 µM) in PBS and the fluorescent probe dihydroethidium (DHE) was added (5 µM, DMSO). Measurements were performed in the absence and presence of SOD (100 U/mL), with the latter serving as an enzymatic control for the detection of superoxide anion (Figure S71-S72). In addition, control experiments were performed in the absence of substrate (GSH or NADH). Fluorescence spectra were recorded after different irradiation times, monitoring the emission band at 610 nm (λ_{ex}= 405 nm).

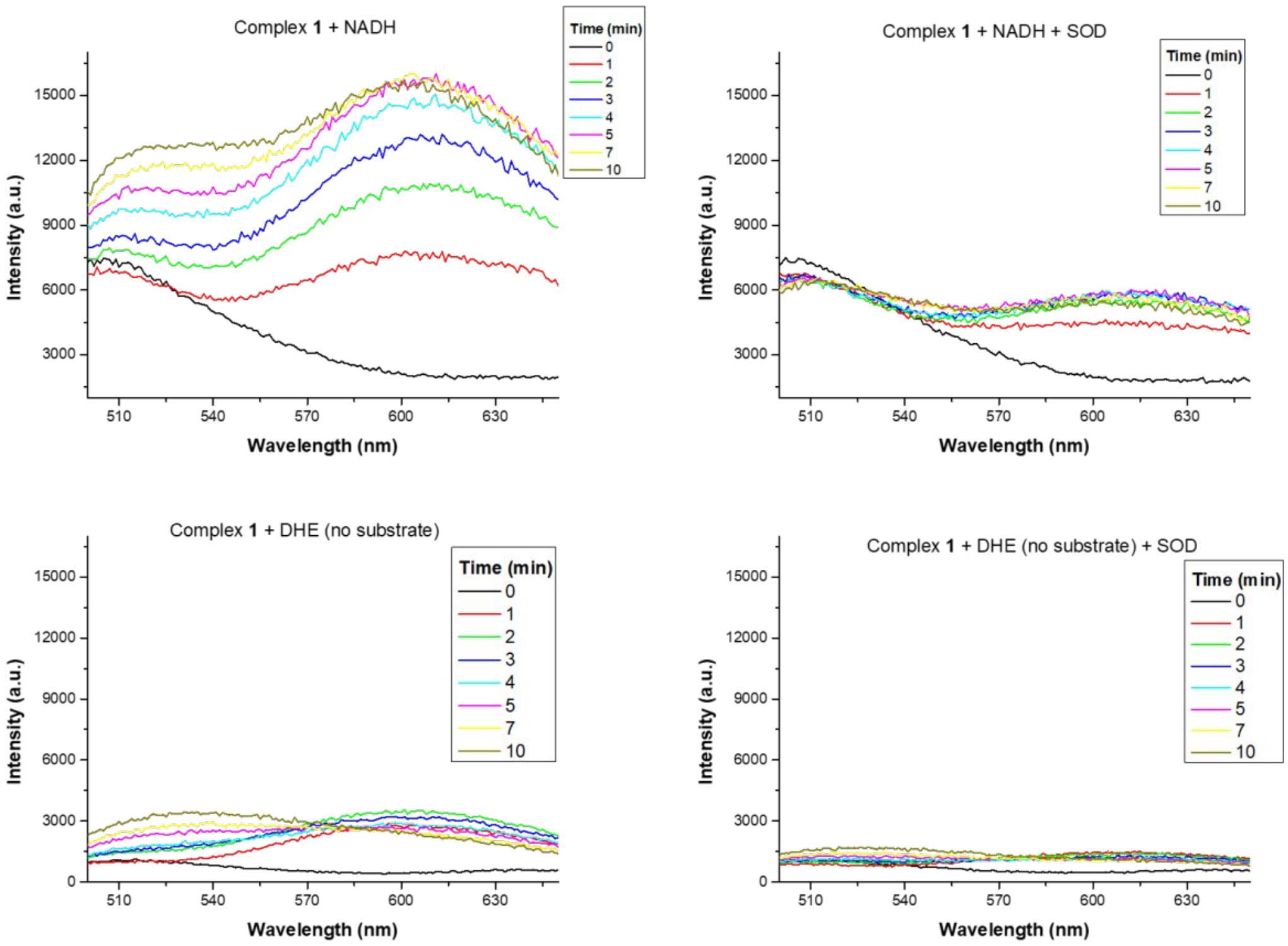

Figure S71. Detection of superoxide generation by DHE fluorescence upon irradiation of complex **1** at 470 nm in the presence of NADH, with SOD (upper right) and without SOD (upper left). Control luminescence spectra recorded in the absence of substrate, with SOD (lower right) and without SOD (lower left).

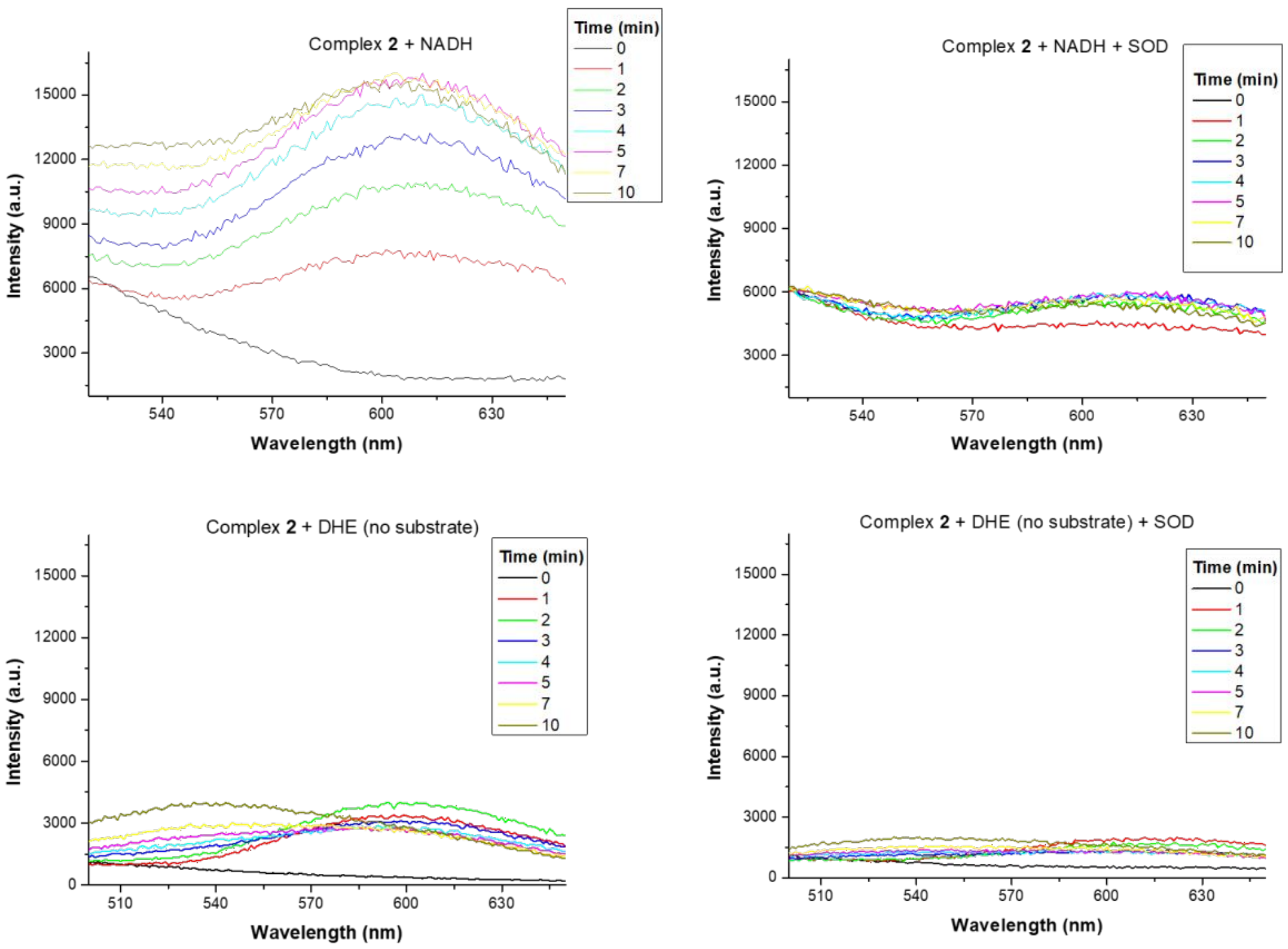

Figure S72. Detection of superoxide generation by DHE fluorescence upon irradiation od complex **2** at 470 nm in the presence of NADH, with SOD (upper left) and without SOD (upper

right). Control luminescence spectra recorded in the absence of substrate, with SOD (lower right) and without SOD (lower left).

6.1.2.2. Catalysis in presence of biomolecules

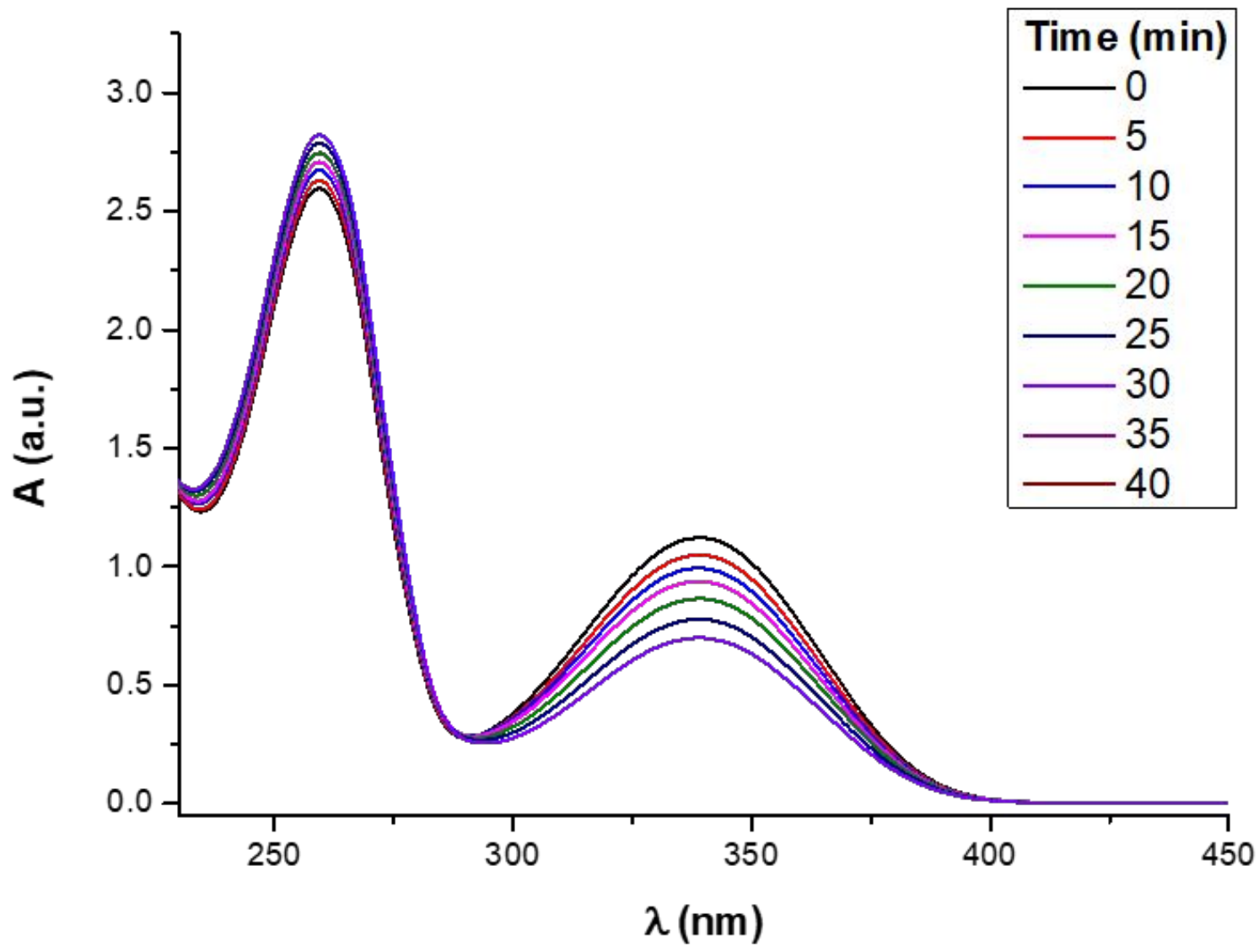

Figure S73. UV-vis spectrum of NADH in presence of complex **1** (10 μM; 5 mol%) and 5 mg/mL BSA under irradiation at 470 nm.

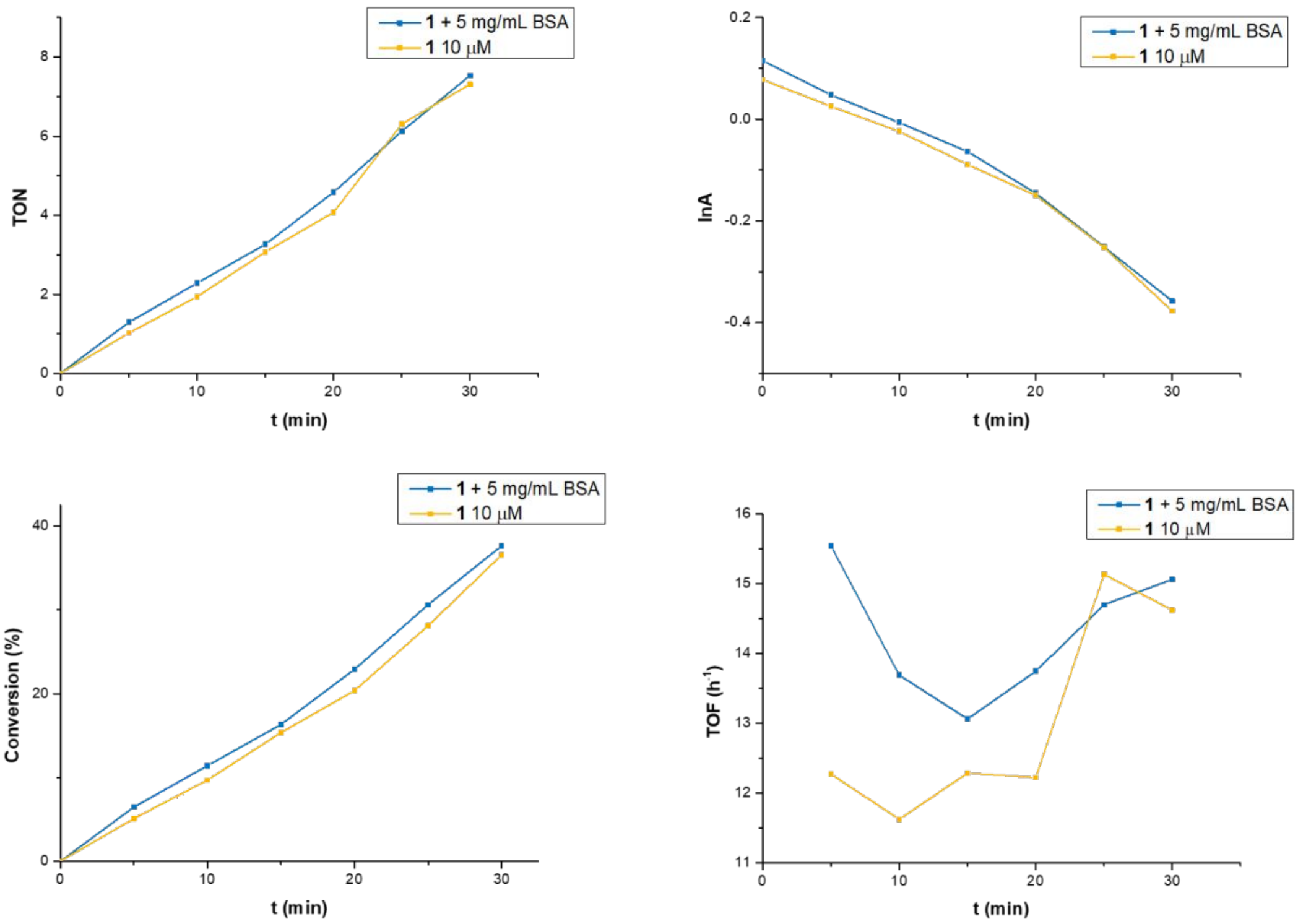

Figure S74. Compared plots of TON, lnA, conversion and TOF for complex **1** (10 μM; 5 mol%) and 5 mg/mL BSA under irradiation at 470 nm.

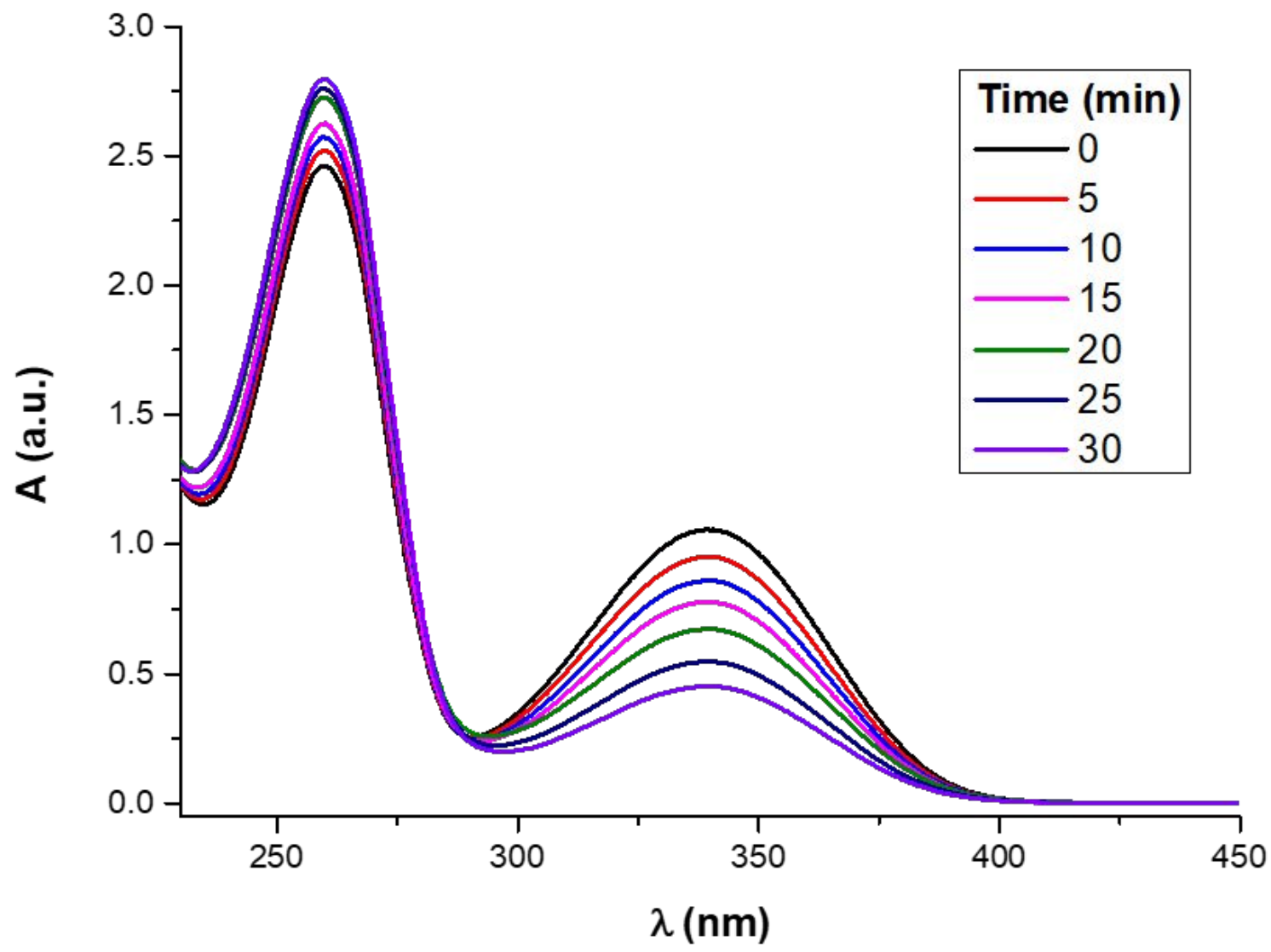

Figure S75. UV-vis spectrum of NADH in presence of complex **2** (10 µM; 5 mol%) and 5 mg/mL BSA under irradiation at 470 nm.

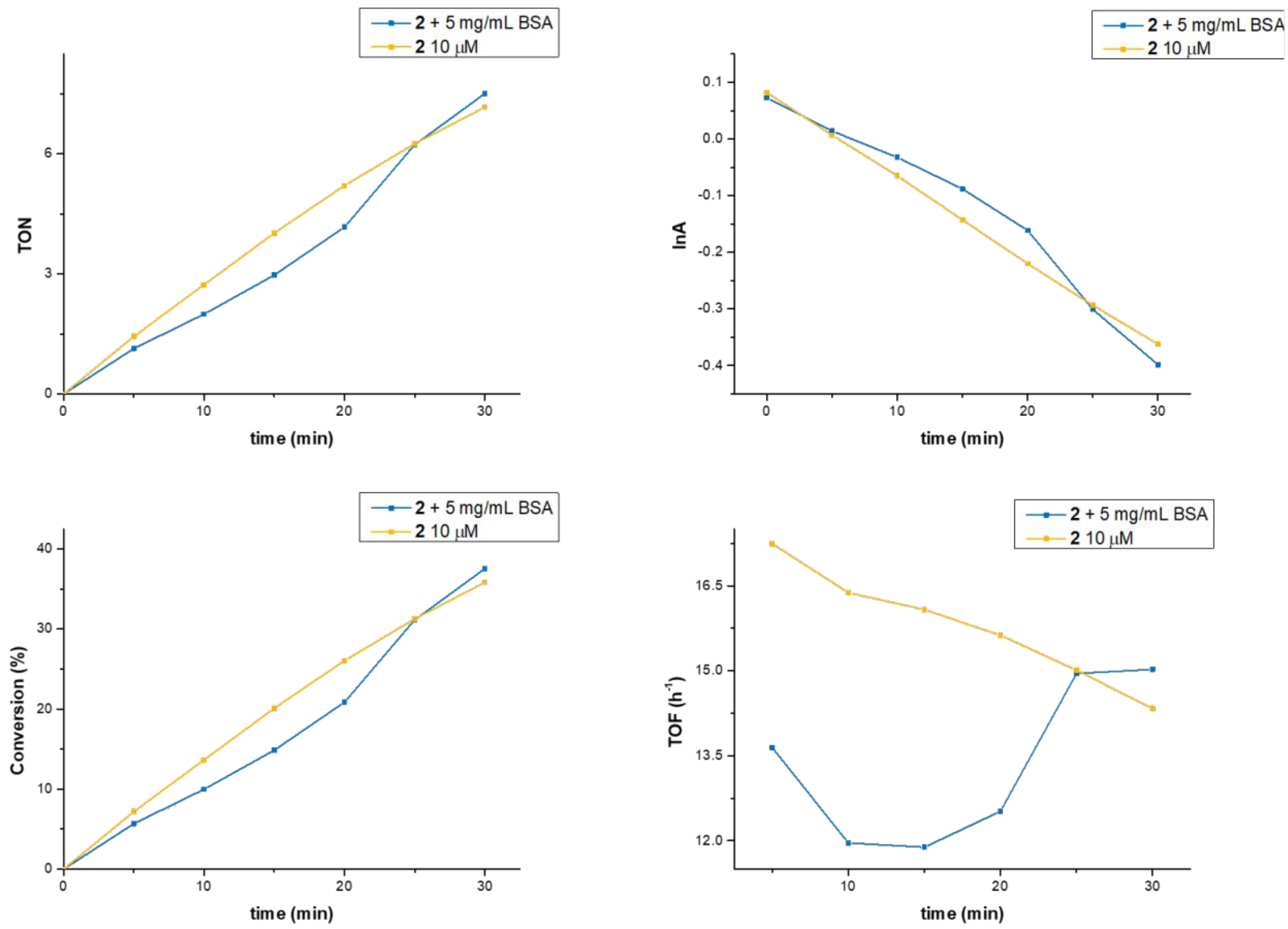

Figure S76. Compared plots of lnA, conversion and TOF for complex **2** (10 µM; 5 mol%) and 5 mg/mL BSA under irradiation at 470 nm.

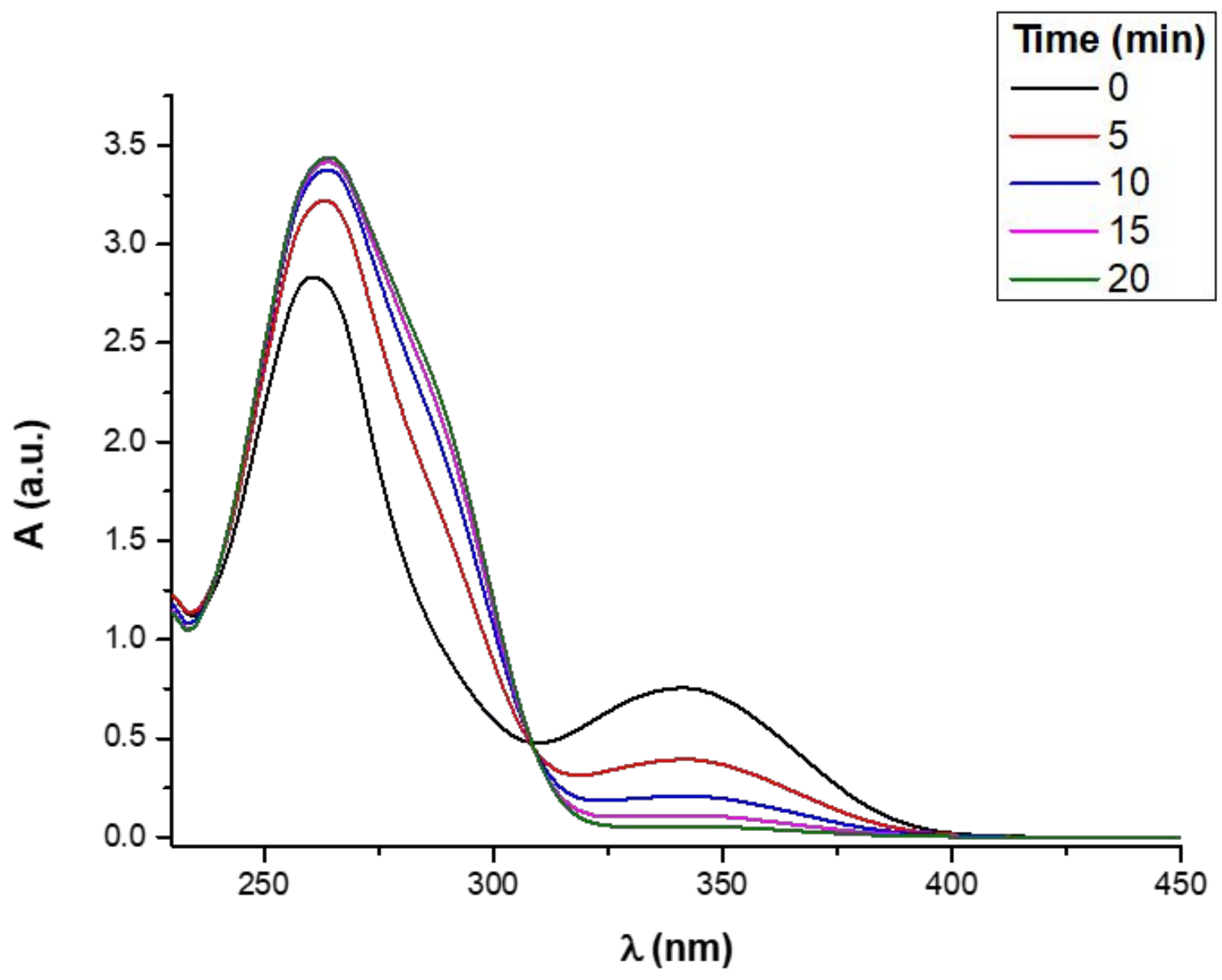

Figure S77. UV-vis spectrum of NADH in presence of complex **1** (10 µM; 5 mol%) and 10 mM GSH under irradiation at 470 nm.

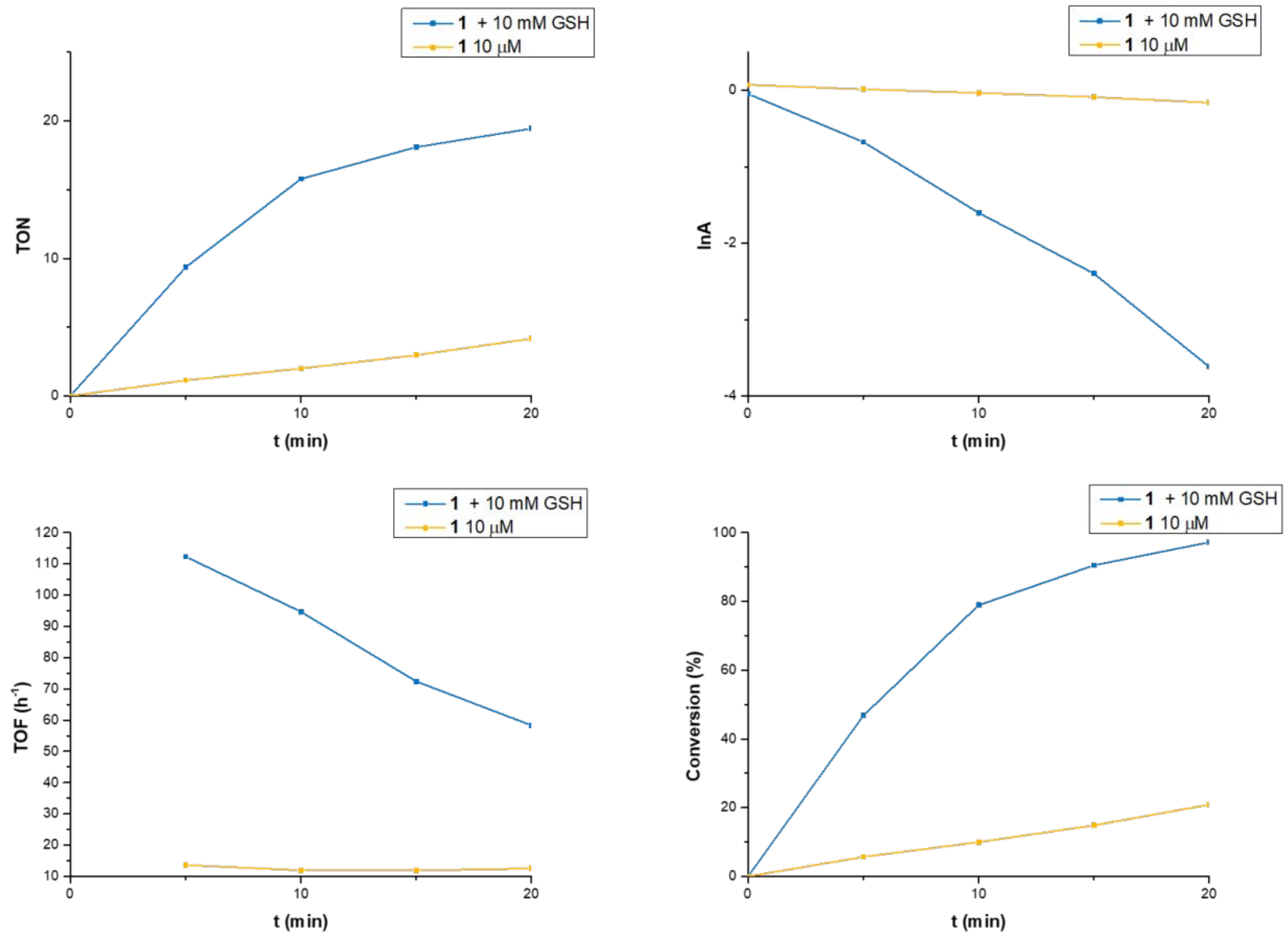

Figure S78. Compared plots of TON, lnA, TOF and conversion for complex **1** (10 µM; 5 mol%) and 10 mM GSH under irradiation at 470 nm.

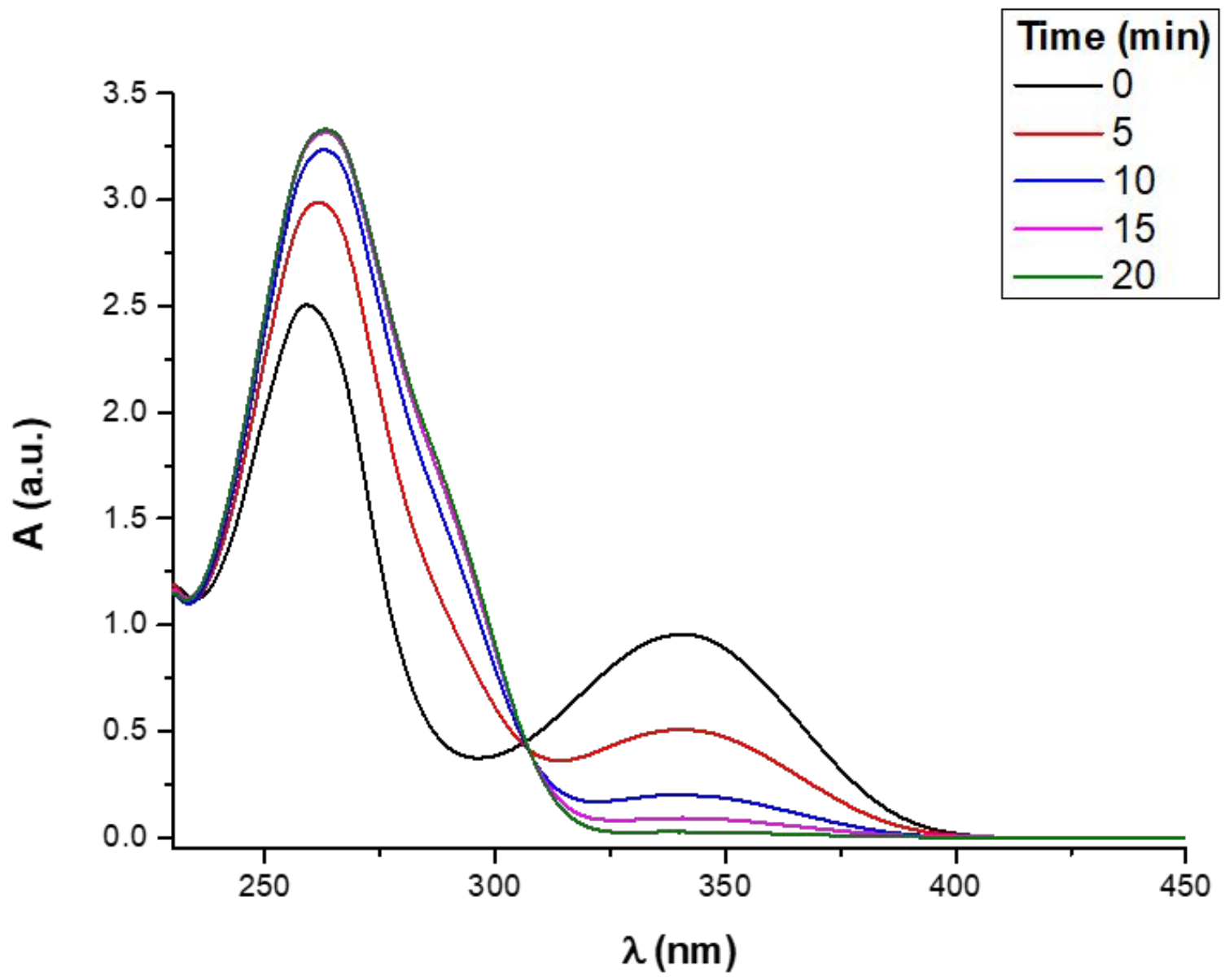

Figure S79. UV-vis spectrum of NADH in presence of complex **2** (10 µM; 5 mol%) and 10 mM GSH under irradiation at 470 nm.

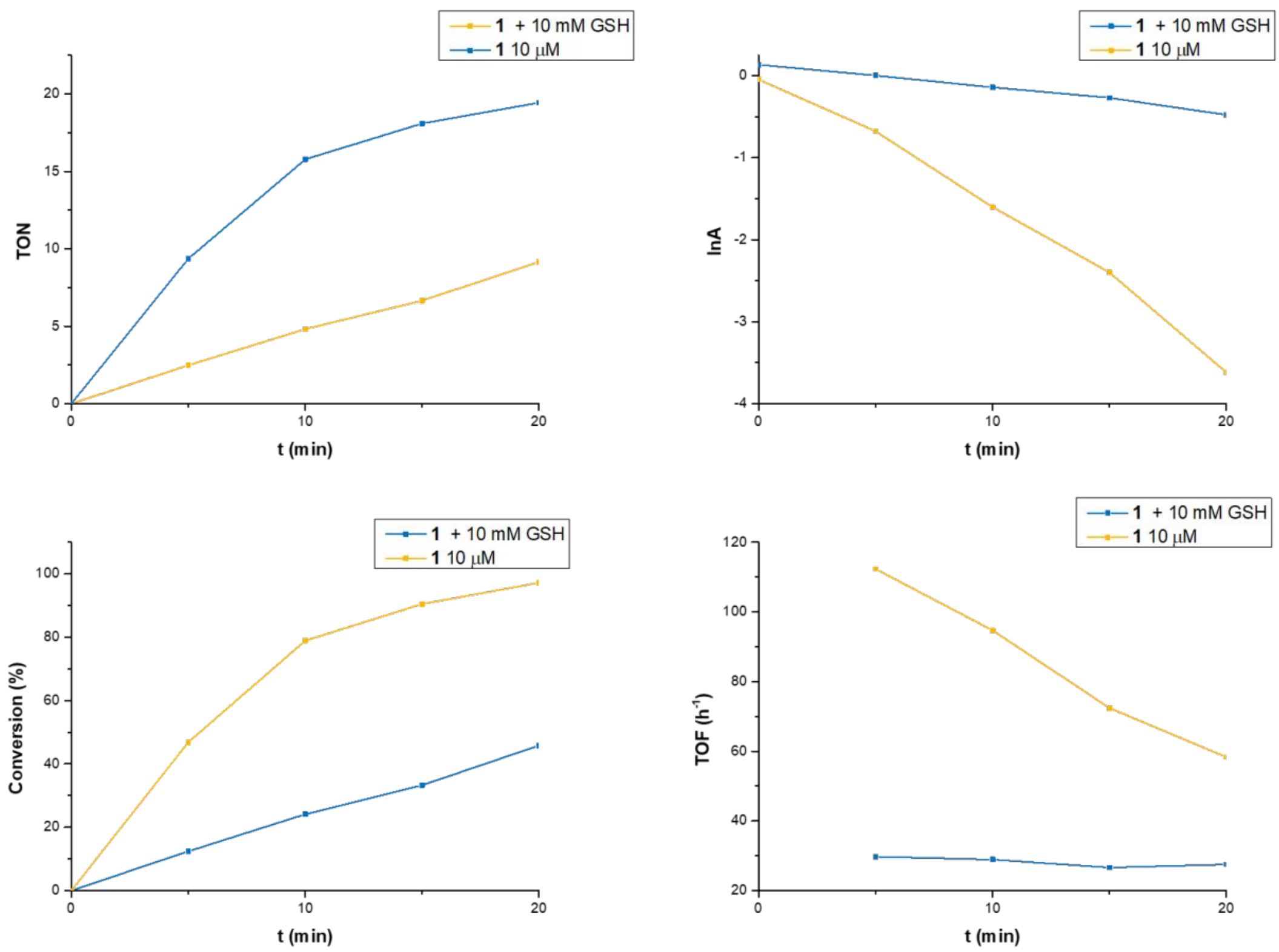

Figure S80. Compared plots of TON, lnA, TOF and conversion for complex **2** (10 µM; 5 mol%) and 10 mM GSH under irradiation at 470 nm.

6.2. GSH photooxidation

Catalyst **1** and **2** (20 µM; 5 mol%) were mixed with GSH (400 µM) in 7 mL PBS. At different irradiation times (at 470 nm, 525 nm or 600 nm), 250 µL of the catalytic reaction solution was added into 2250 µL PBS, and then 10 µL of DTNB (5,5'-dithiobis-(2-nitrobenzoic acid)) (10 mg/mL) were added. The absorbance of this solution was measured by UV-vis spectroscopy at different times, monitoring the band at 412 nm.[5]

6.2.1. Kinetic study

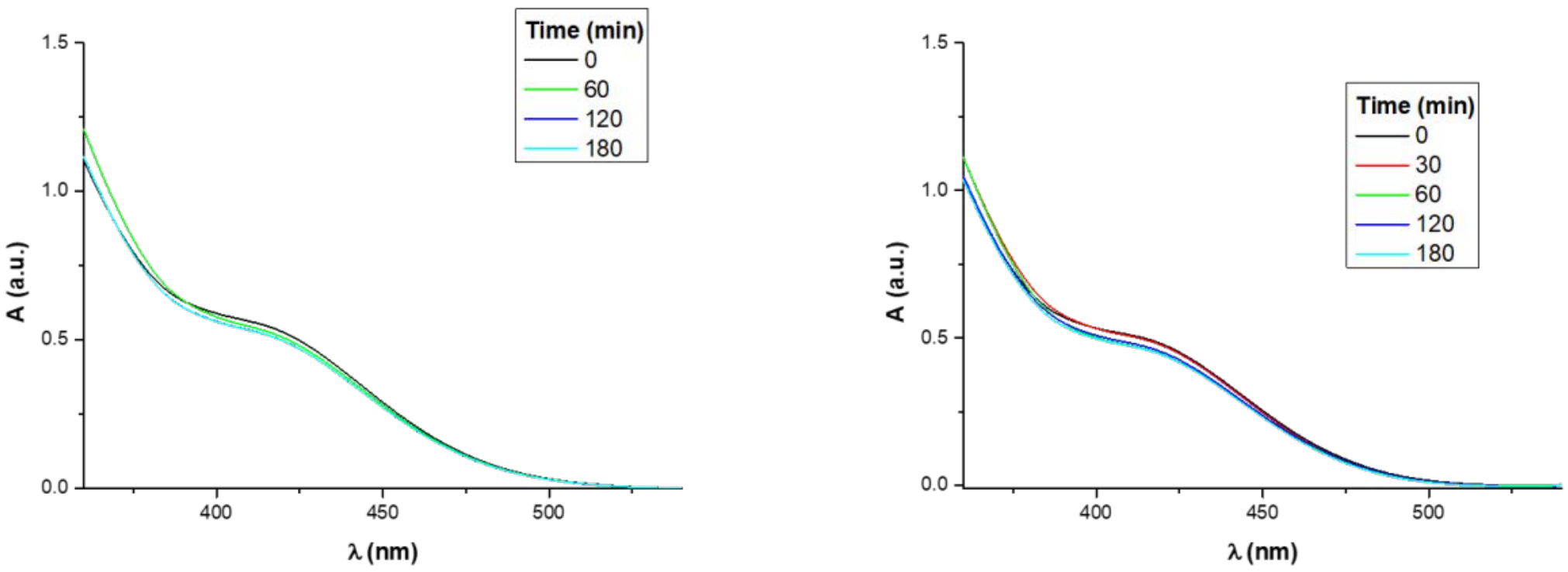

Figure S81. UV-vis spectrum of GSH in presence of **1** (20 µM, 5 mol%, left) and **2** (20 µM, 5 mol%, right) in dark.

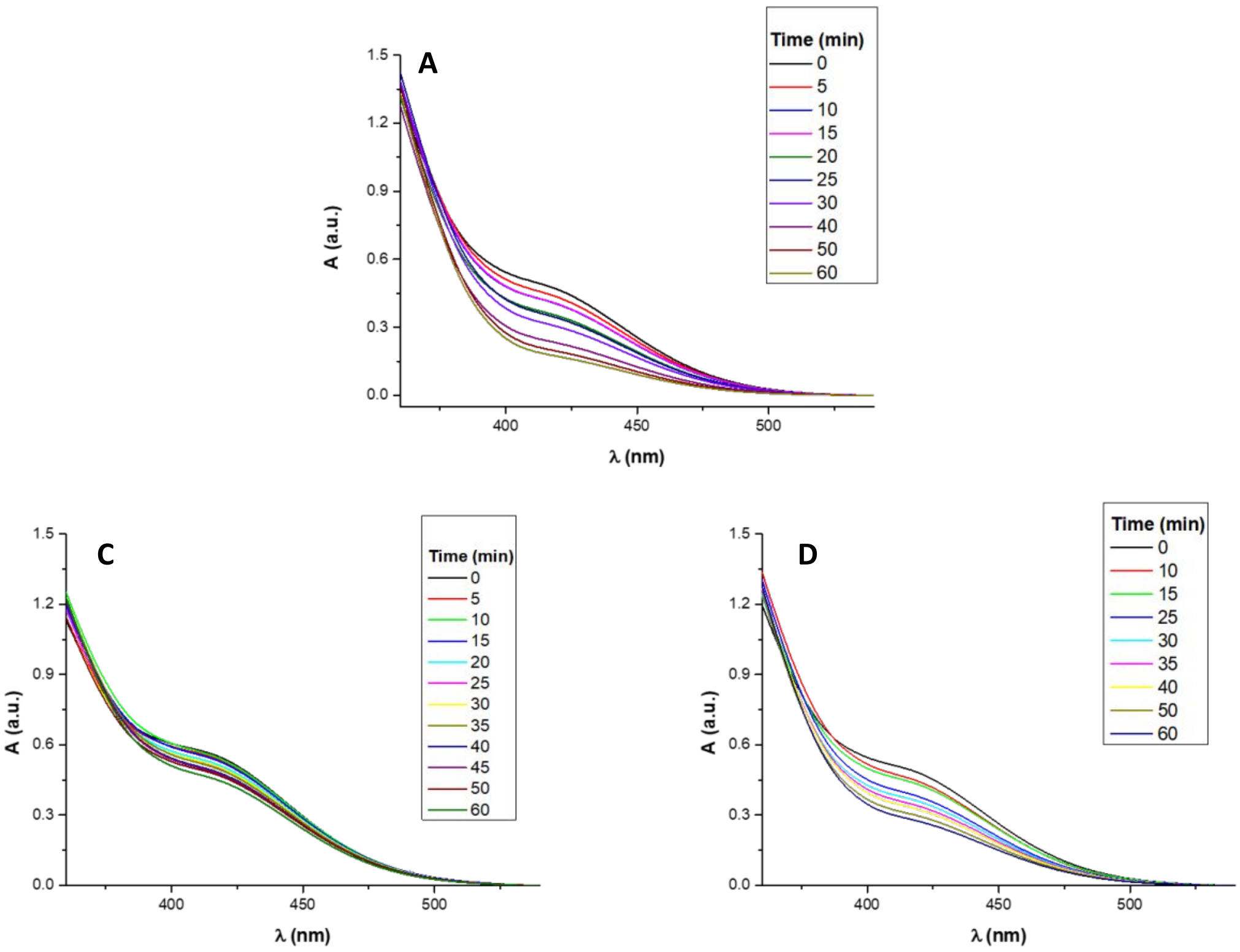

Figure S82. UV-vis spectrum of GSH in presence of catalyst (20 µM; 5 mol%) in different conditions: **A** complex **1** under 470 nm irradiation, **C** complex **1** under 525 nm irradiation, **D** complex **2** under 525 nm irradiation.

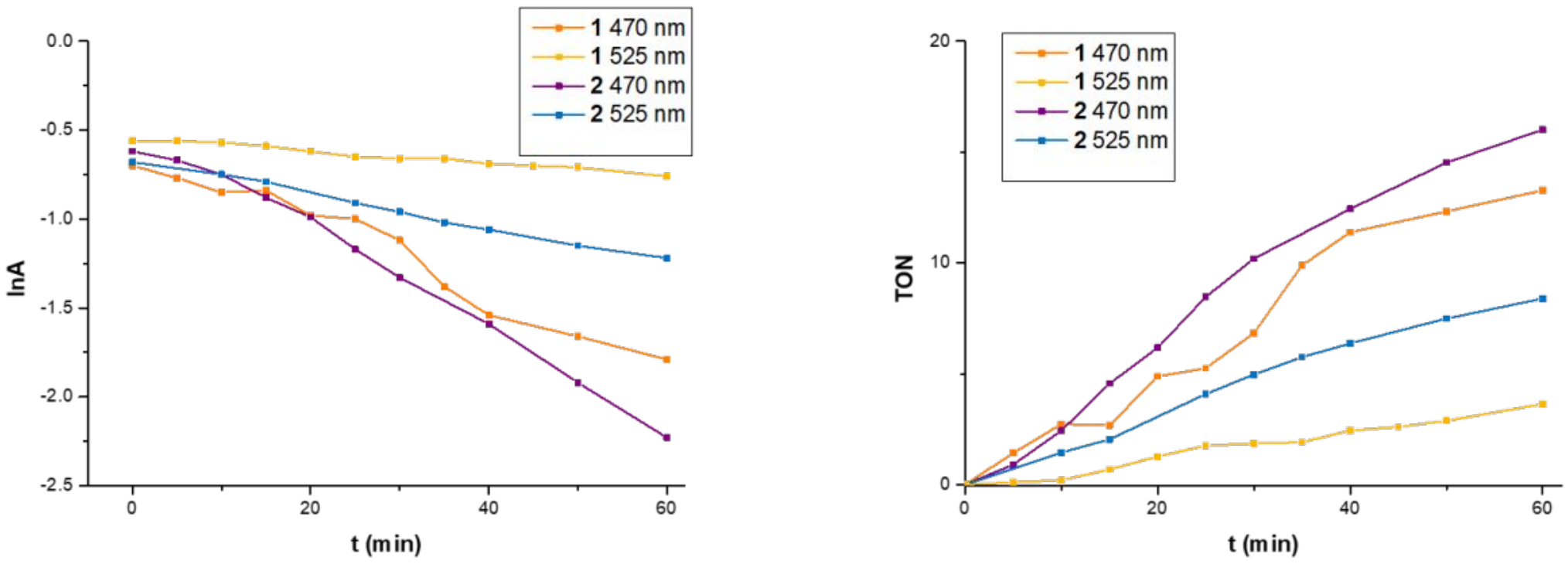

Figure S83. Compared plots of lnA and TON for complexes **1** and **2** (20 mM; 5 mol%) under irradiation at 470 and 525 nm.

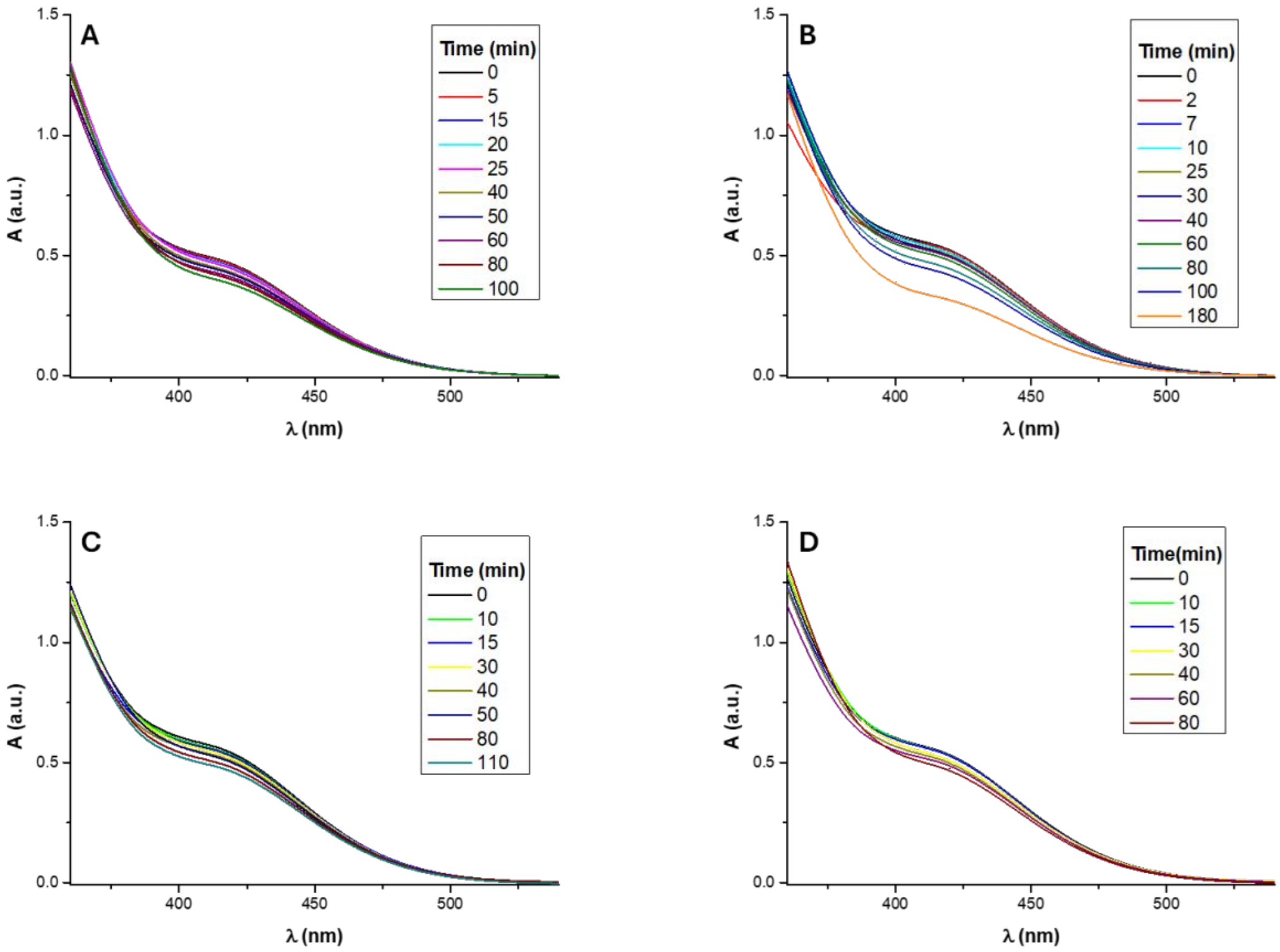

Figure S84. UV-vis spectrum of GSH in presence of catalyst (0.2 μM; 0.05 mol%) in different conditions: **A** complex **1** under 470 nm irradiation, **B** complex **2** under 470 nm irradiation **C** complex **1** under 525 nm irradiation, **D** complex **2** under 525 nm irradiation.

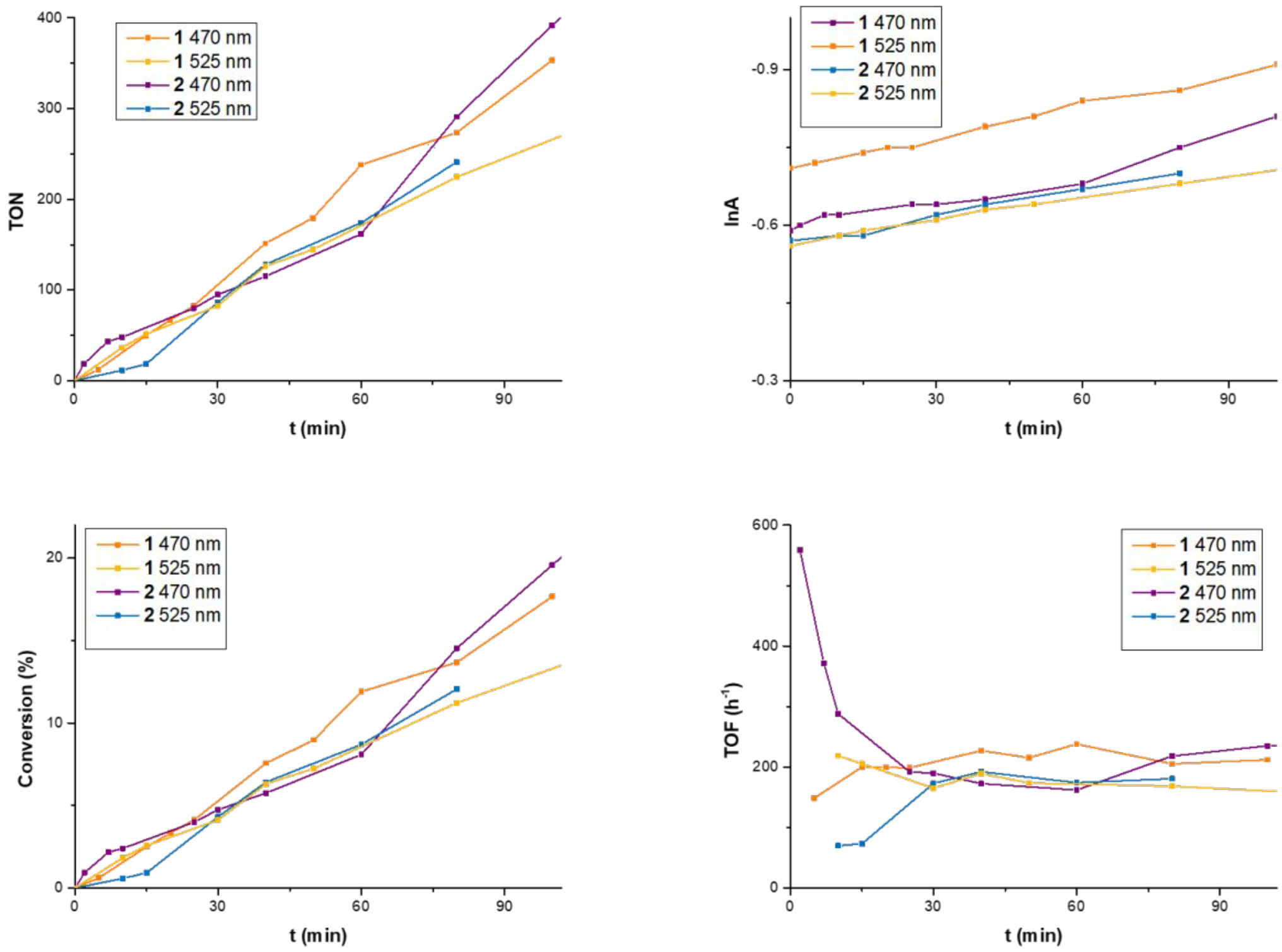

Figure S85. Compared plots of TON, lnA, conversion and TOF for complexes **1** and **2** (0.2 mM; 0.05 mol%) under irradiation at 470 and 525 nm.

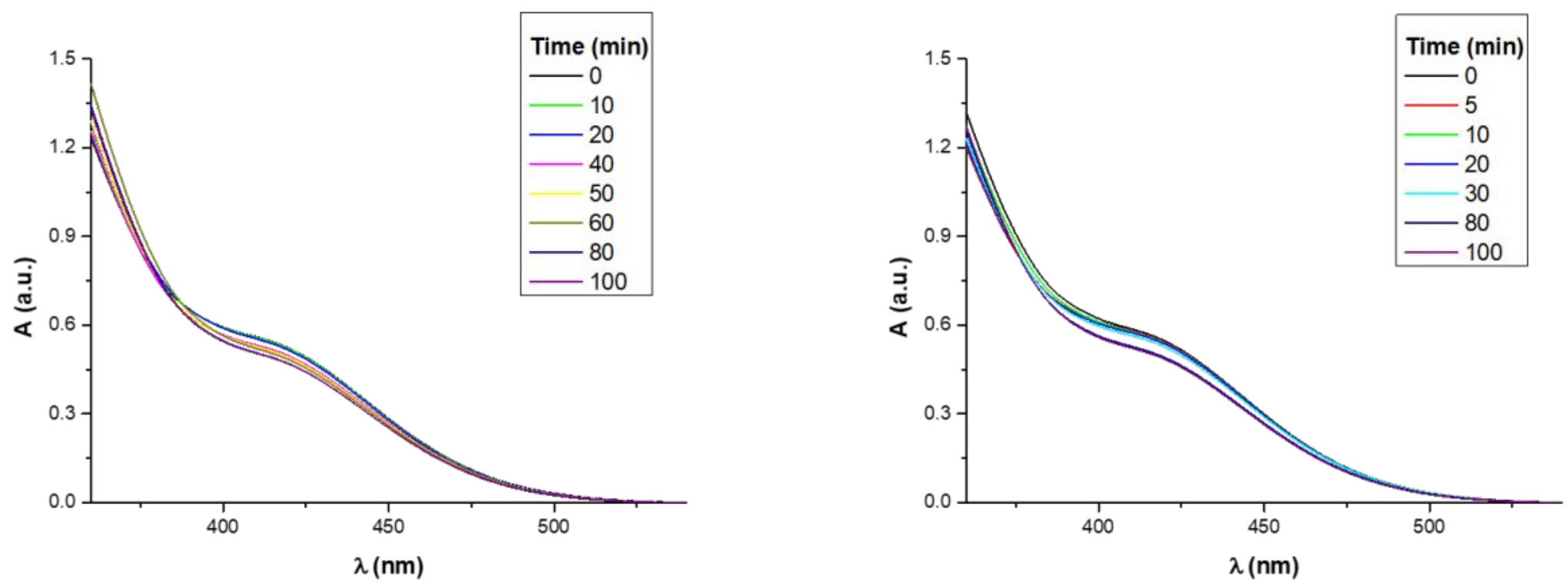

Figure S86. UV-vis spectrum of GSH in presence of **1** (20 μM, 5 mol%, left) and **2** (20 μM, 5 mol%, right) under irradiation at 600 nm.

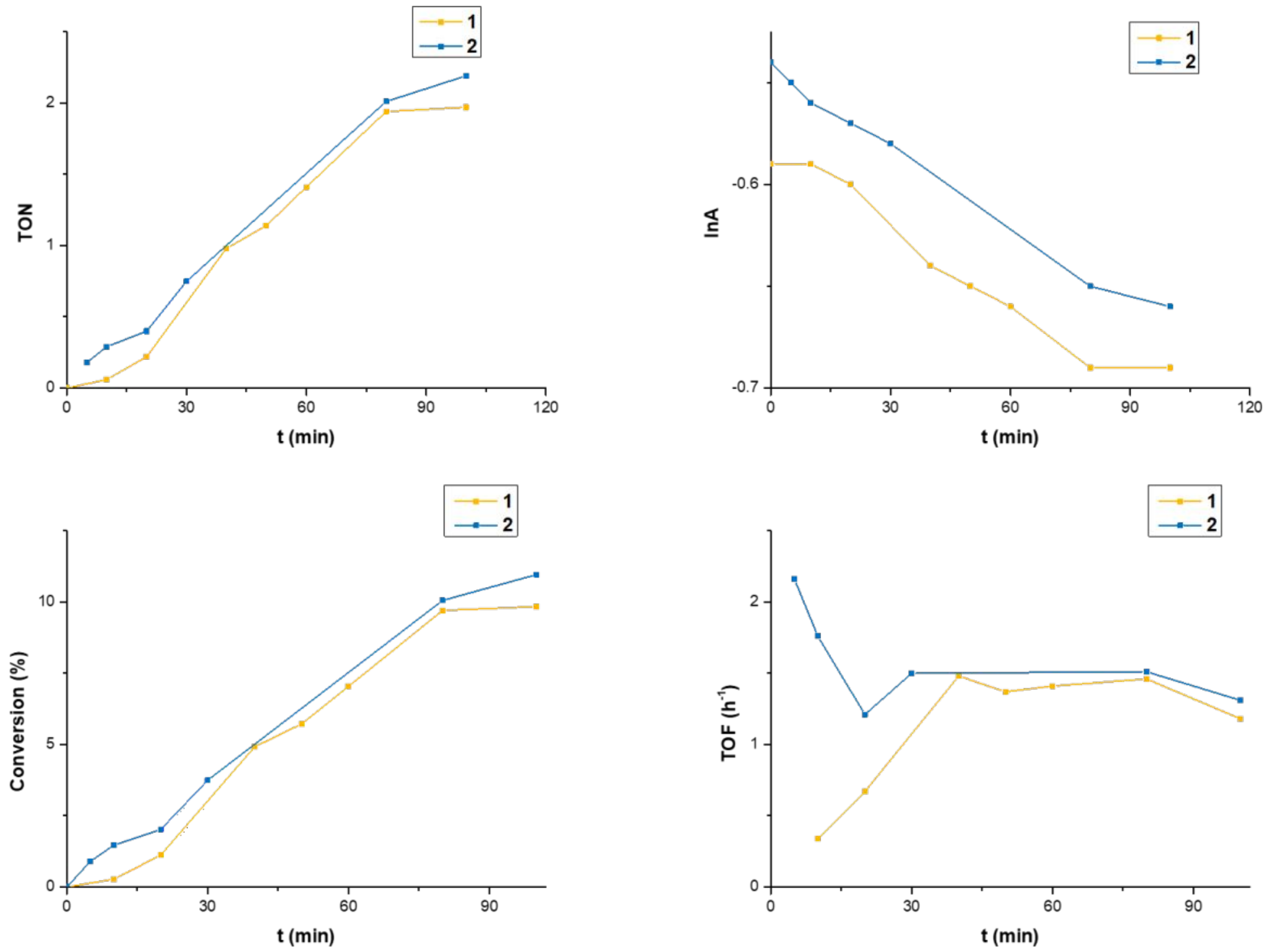

Figure S87. Compared plots of TON, lnA, conversion and TOF for complexes **1** and **2** (20 mM; 5 mol%) under irradiation at 600 nm.

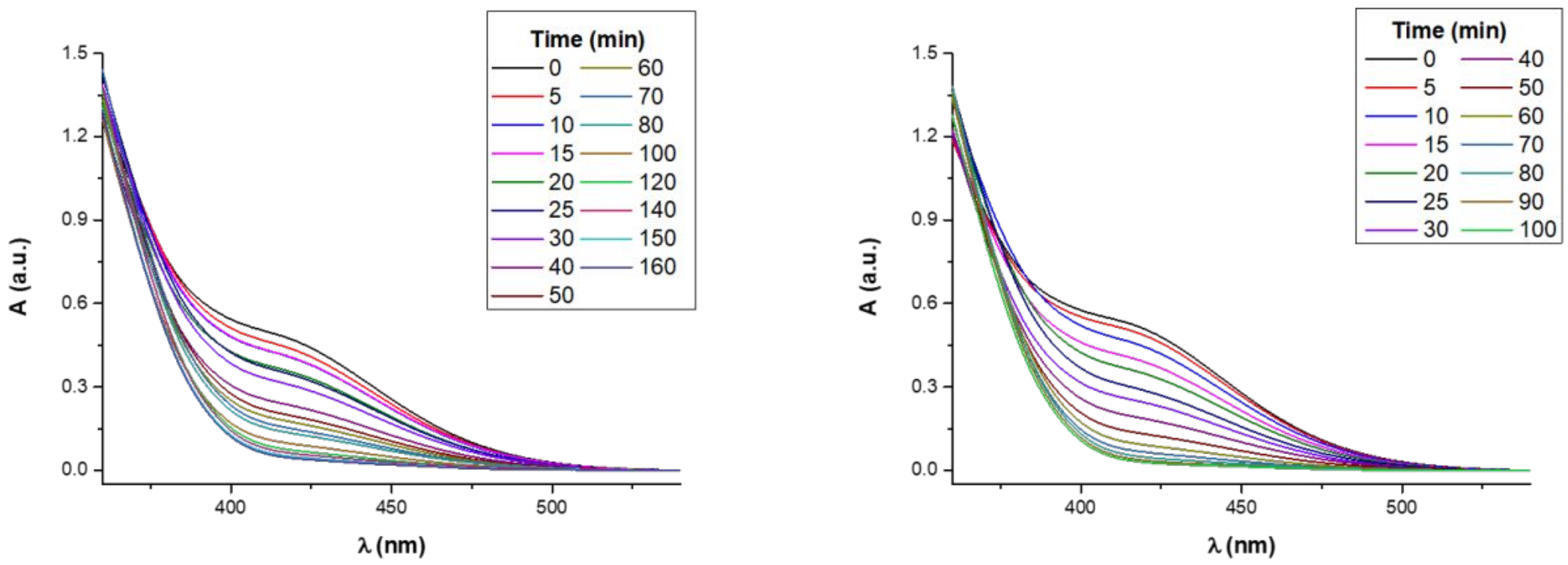

Figure S88. UV-vis of GSH in presence of complexes **1** (left) and **2** (right) (20 μM; 5 mol%) until complete conversion (>92%) under irradiation at 470 nm.

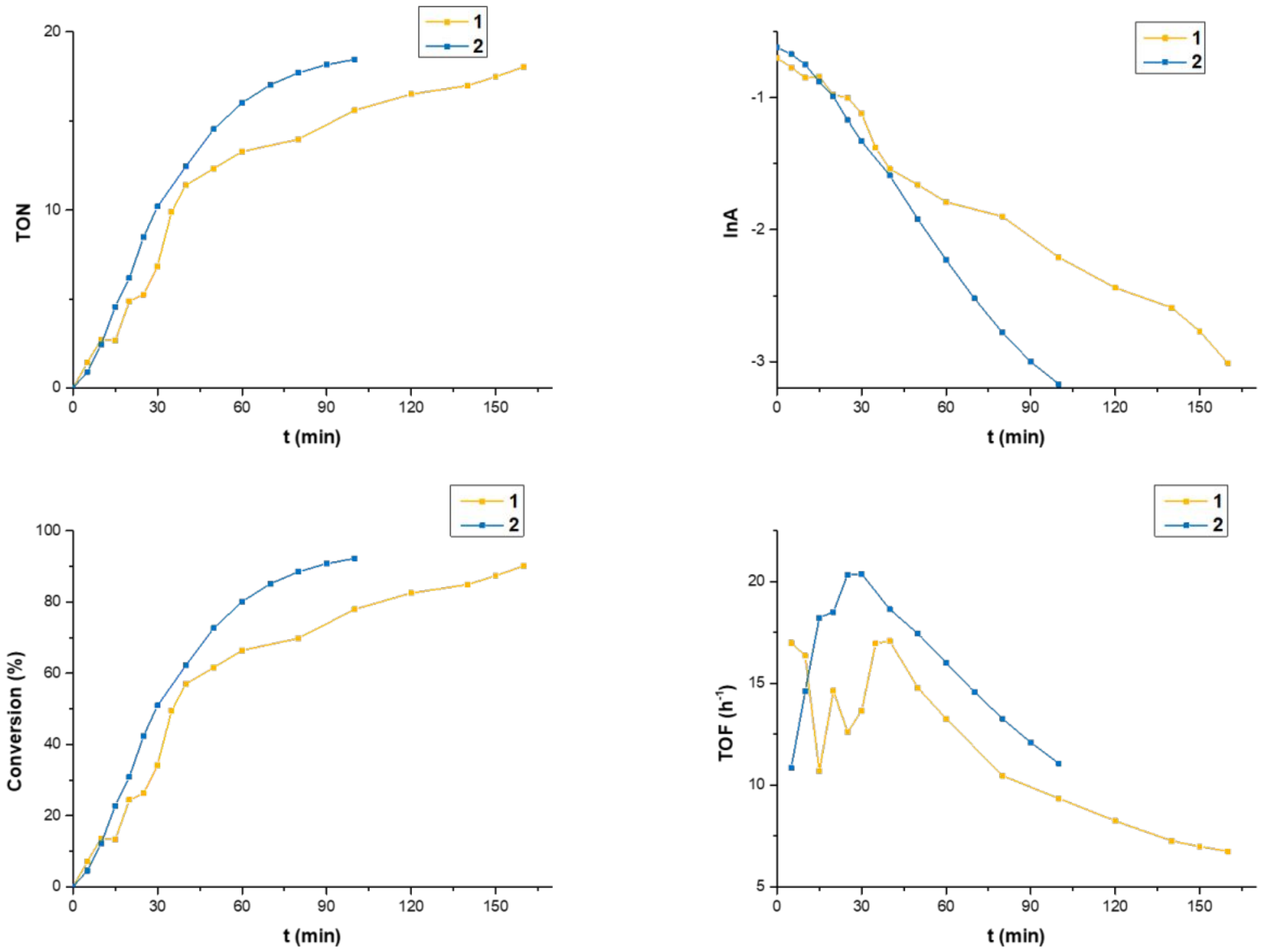

Figure S89. Compared plots of TON, lnA, conversion and TOF for complexes **1** and **2** (20 μM; 5 mol%) until complete conversion (>92%) under irradiation at 470 nm.

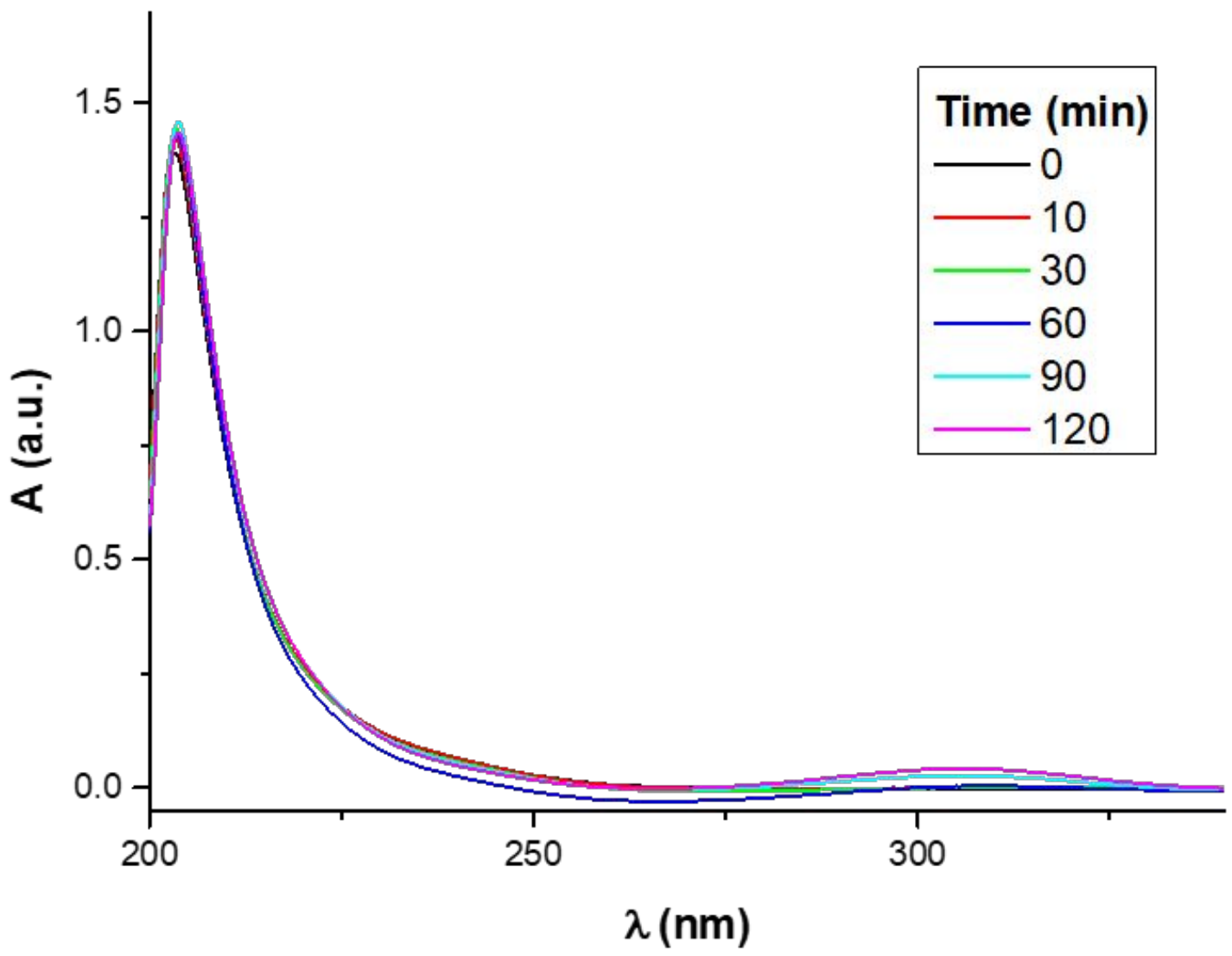

Figure S90. UV-vis spectrum of GSH under irradiation at 470 nm up to 2 h.

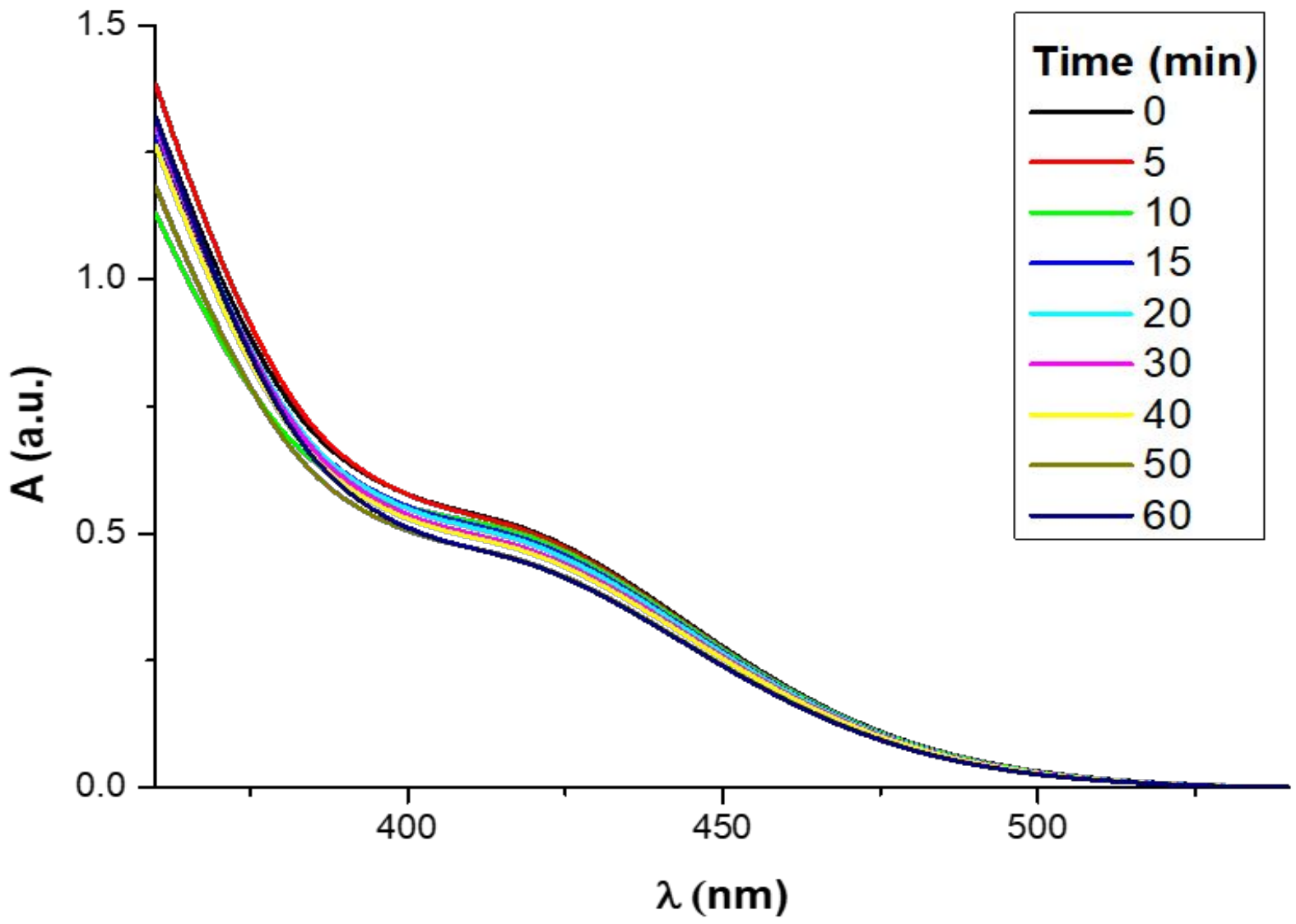

Figure S91. UV-vis spectrum of GSH in presence of ligand **L** (20 μM; 5 mol%) under irradiation at 470 nm.

6.2.2. Mechanistic studies

6.2.2.1. Influence of O_2

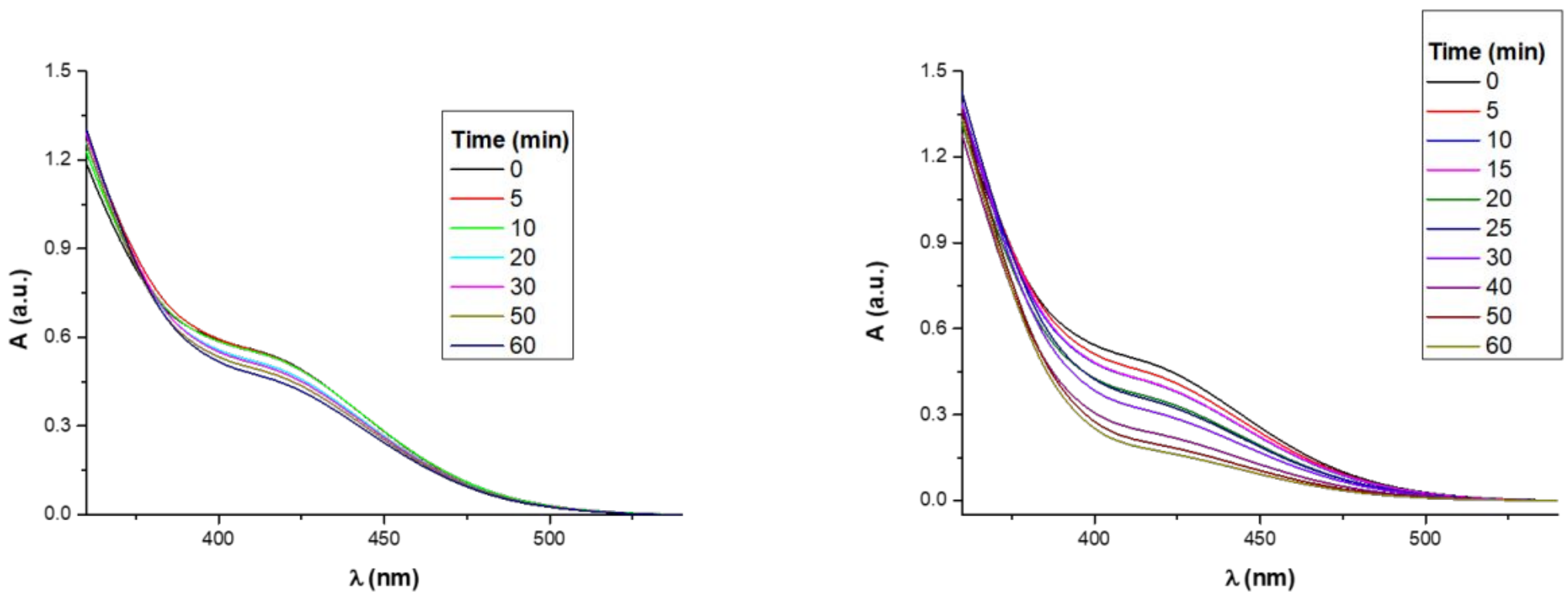

Figure S92. UV-vis spectrum of GSH in presence of complex **1** (20 μM; 5 mol%) under irradiation at 470 nm in air (right) and under Ar atmosphere (left).

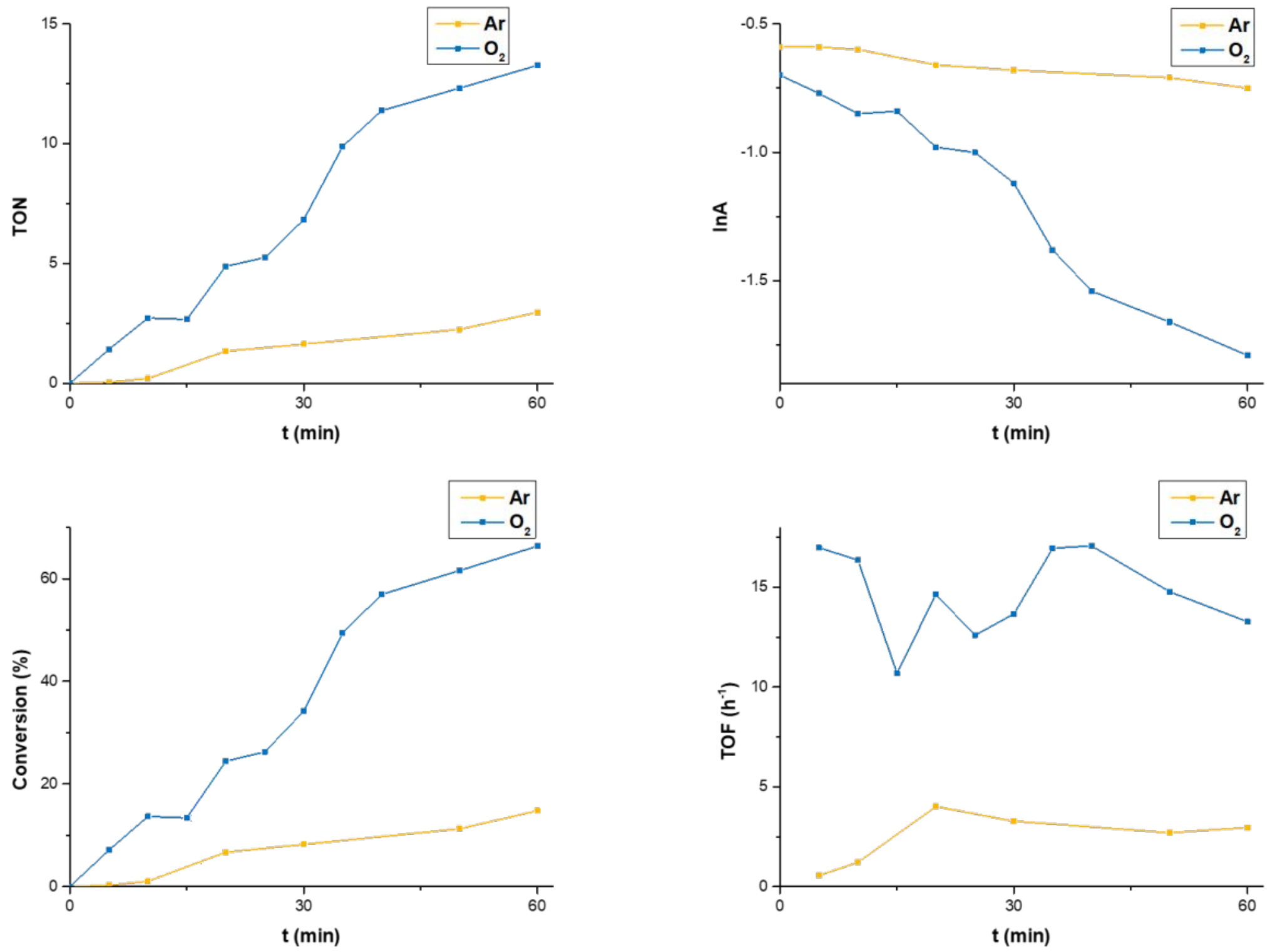

Figure S93. Compared plots of TON, lnA, conversion and TOF for complex **1** under irradiation at 470 nm in air and under Ar atmosphere.

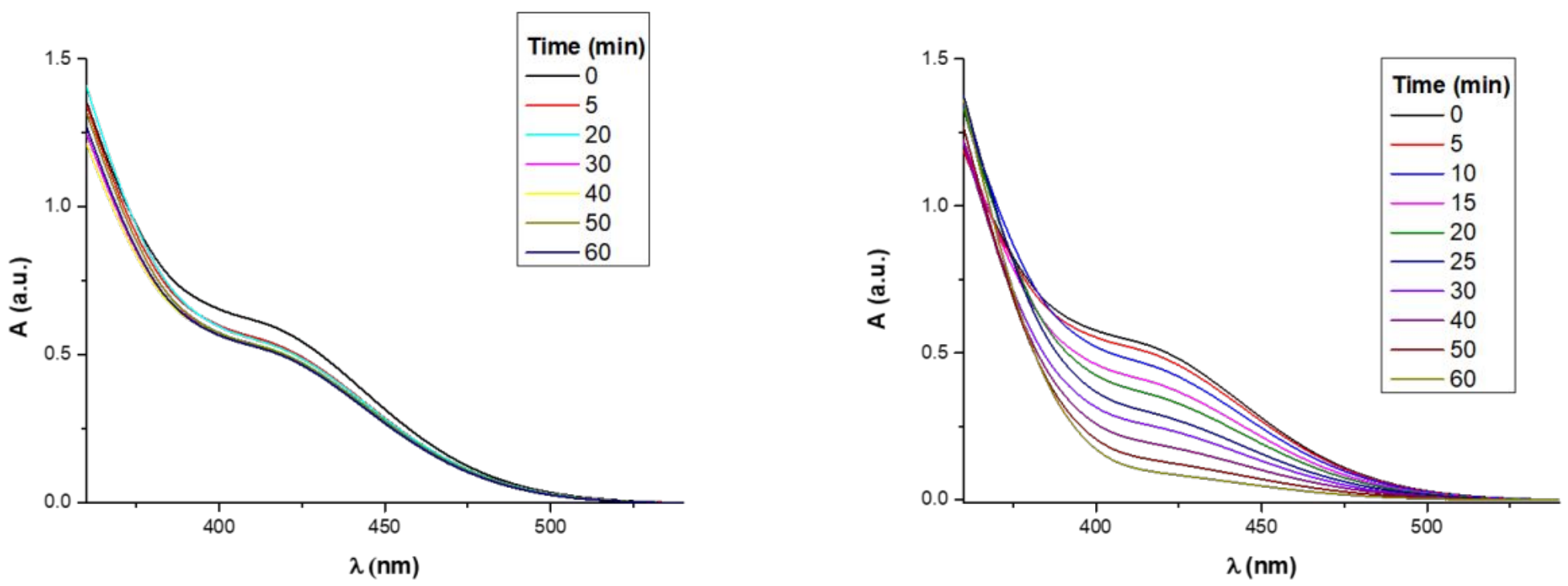

Figure S94. UV-vis spectrum of GSH in presence of complex **2** (20 μM; 5 mol%) under irradiation at 470 nm in air (right) and under Ar atmosphere (left).

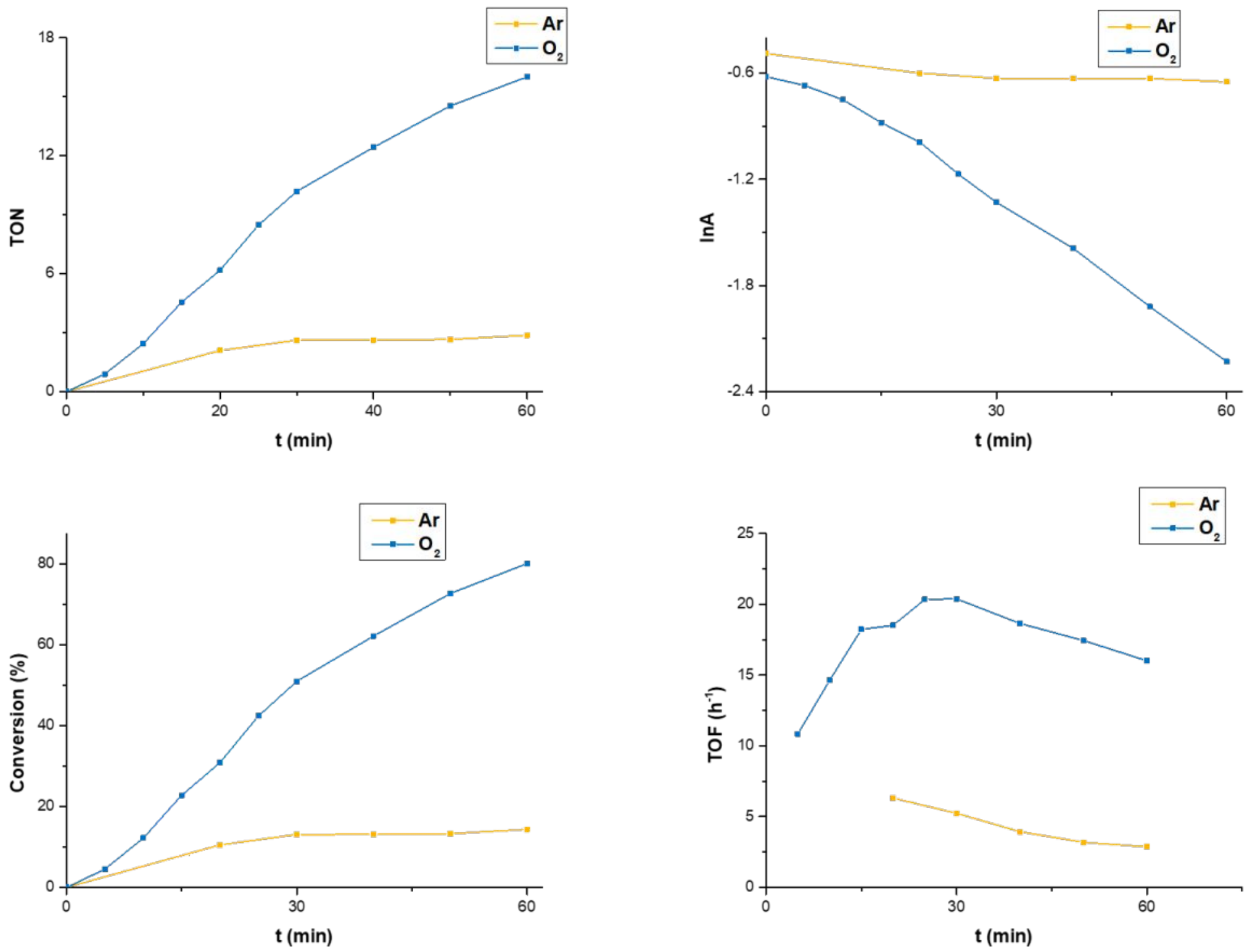

Figure S95. Compared plots of TON, lnA, conversion and TOF for complex **2** under irradiation at 470 nm in air and under Ar atmosphere.

6.2.2.1. Monitoring of superoxide generation during photocatalysis using DHE

Catalysts **1** and **2** (20 µM) were mixed with GSH (200 µM) in PBS and the fluorescent probe dihydroethidium (DHE) was added (5 µM, DMSO). Measurements were performed in the absence and presence of SOD (100 U/mL), with the latter serving as an enzymatic control for the detection of superoxide anion (Figure S96). Fluorescence spectra were recorded after different irradiation times, monitoring the emission band at 610 nm (λ_{ex}= 405 nm).

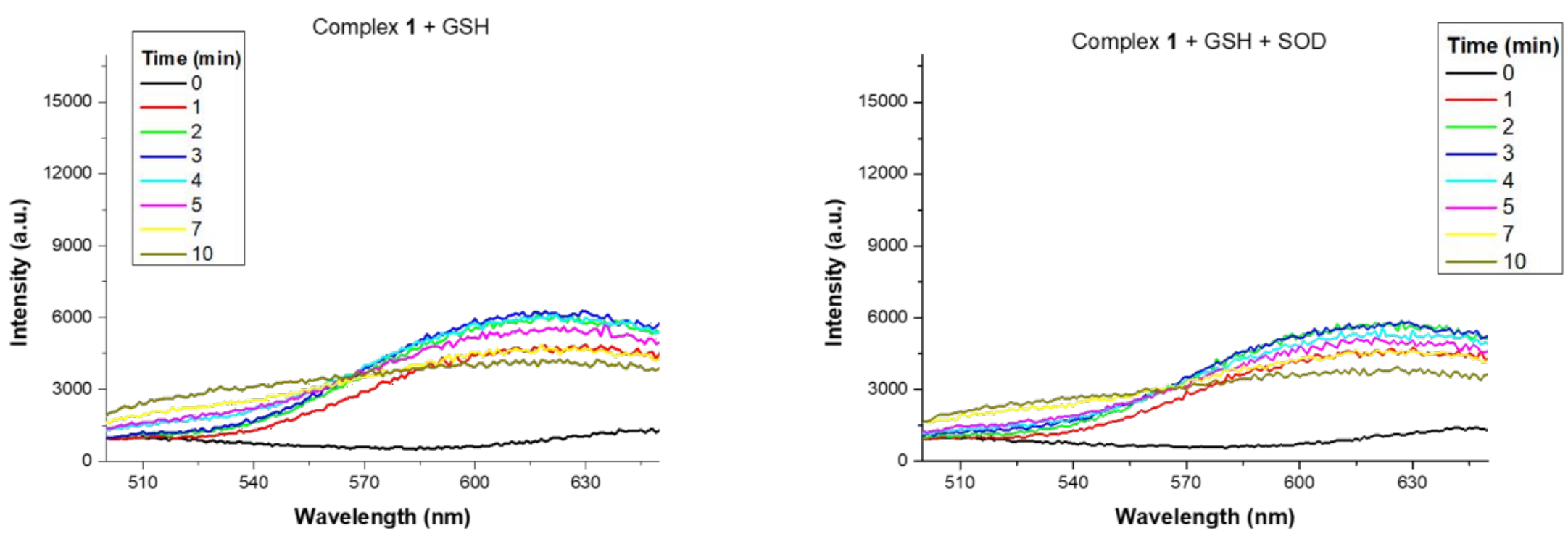

Figure S96. Detection of superoxide generation by DHE fluorescence upon irradiation of complex **1** at 470 nm in the presence of GSH, with SOD (right) and without SOD (left)

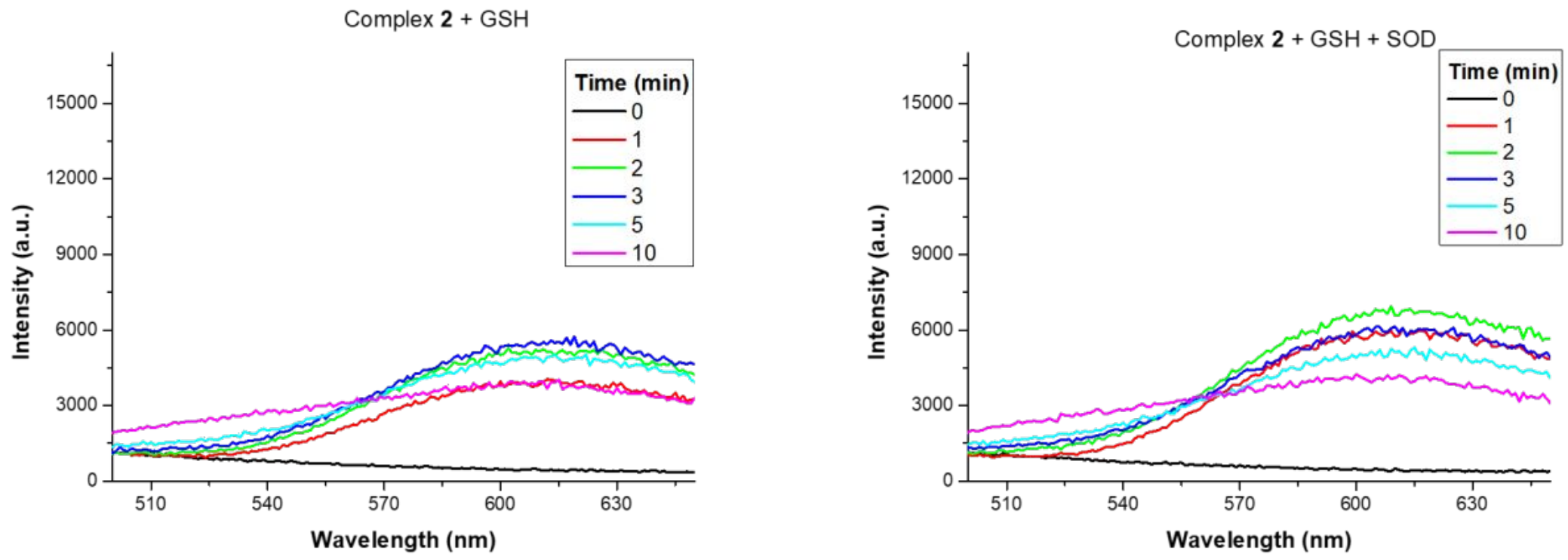

Figure S97. Detection of superoxide generation by DHE fluorescence upon irradiation of complex **2** at 470 nm in the presence of GSH, with SOD (right) and without SOD (left)

7. Biological studies

7.1. DNA Binding

7.1.1. UV-vis

Instrumentation: UV-Vis spectra were recorded with JASCO V-780 spectrophotometer.
Experimental procedure: the complexes were dissolved in H_2O and diluted with the Tris-HCl buffer (10 mM, pH = 7.2). The absorption spectra were performed in fixed concentration of metal complexes (30 μM) while gradually increasing the concentration of CT-DNA with 0 to 100 μM. To obtain the absorption spectra, the required amount of CT-DNA was added to both compound solution and the reference solution to eliminate the absorbance of CT-DNA itself. Each sample solution was allowed to equilibrate 10 min before the spectra was recorded. Using the absorption titration data, the binding constant K_b was determined using the Wolfe-Shimer equation **Eq. S10**:[11,12]

$$\frac{[DNA]}{(\varepsilon_a - \varepsilon_f)} = \frac{[DNA]}{(\varepsilon_a - \varepsilon_f)} + \frac{1}{K_b(\varepsilon_b - \varepsilon_f)} \quad \textbf{Eq. S11}$$

where [DNA] is the concentration of CT-DNA, ε_a corresponds to the extinction coefficient observed (A_{obsd}/[M]), ε_f corresponds to the coefficient of the free compound, ε_b is the extinction coefficient of the compound fully bound to CT-DNA, and K_b is the intrinsic binding constant. The K_b value was determined by the ratio of the slope to the intercept in the plot of [DNA]/(ε_a - εf) versus [DNA].

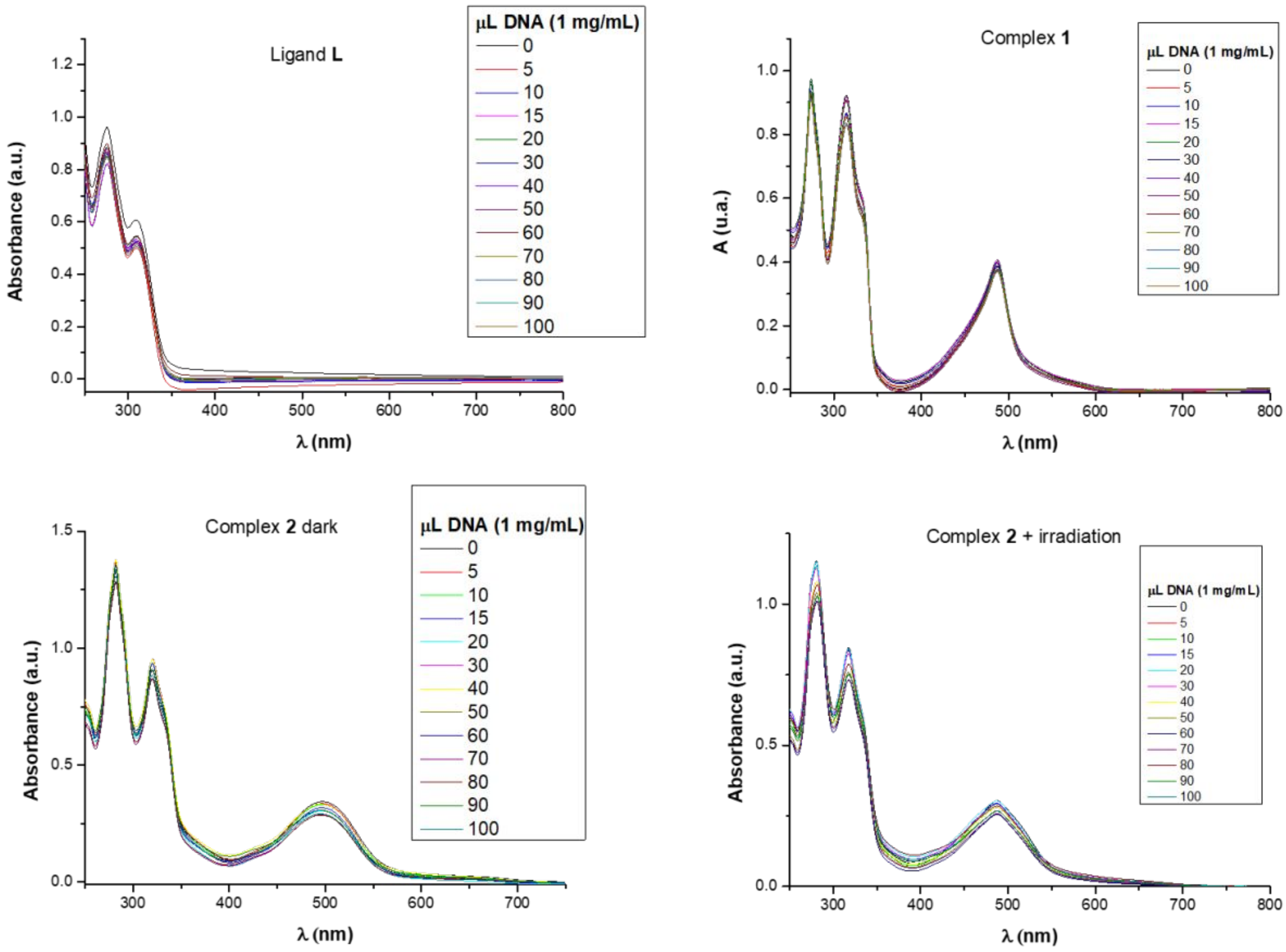

Figure S98. Absorption spectral titration experiment for ligand **L** and complexes **1** and **2**.

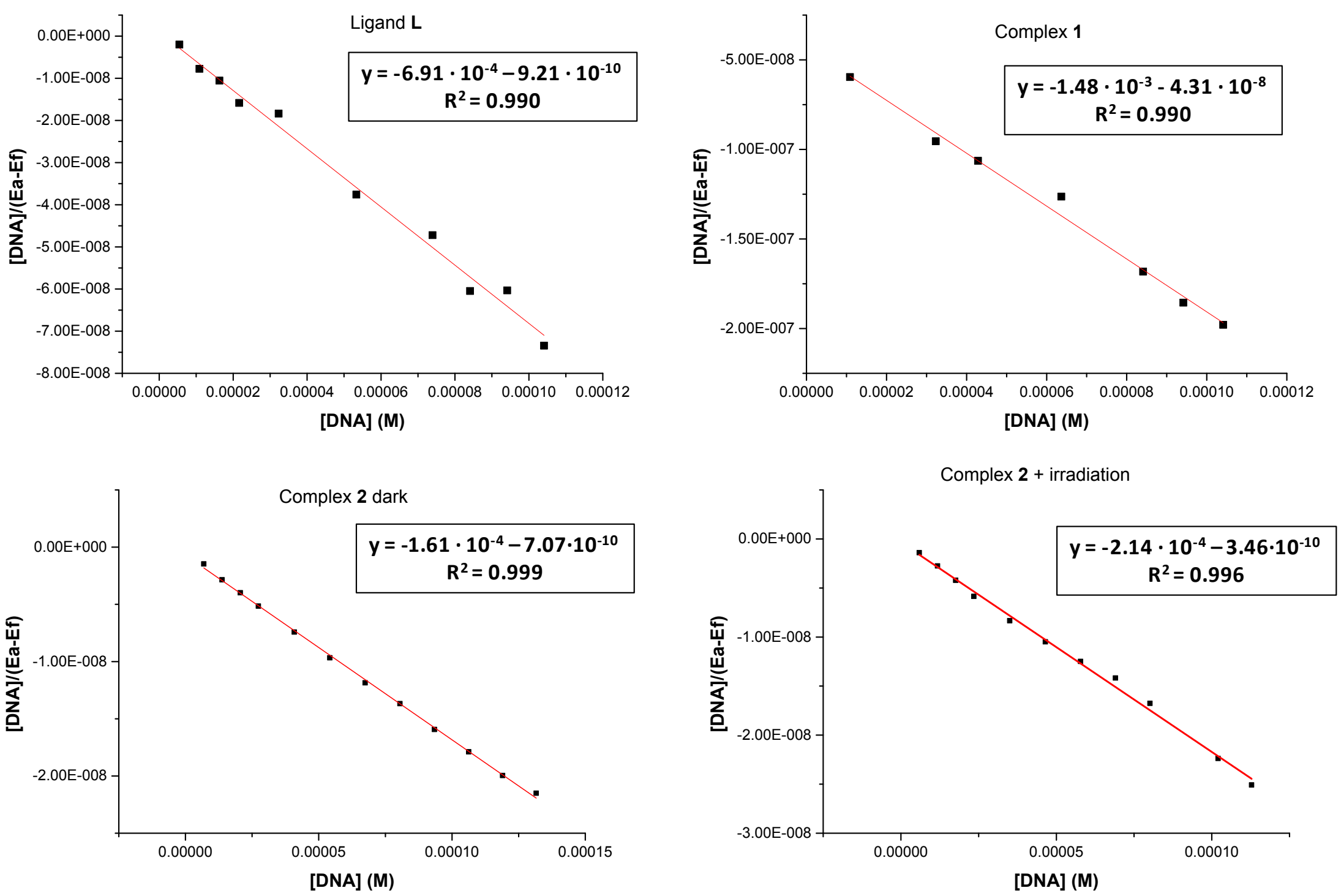

Figure S99. Graphical representation of Wolfe-Shimer equation for ligand **L** and complexes **1** and **2**.

7.1.2. Competitive ethidium bromide (EB) fluorescence displacement assay

A solution containing ethidium bromide (100 µM for **1** and **2** and 55 µM for **L**) and CT-DNA (50 µM) in tris-HCl buffer (10 mM, pH = 7.2) was prepared in a quartz cuvette. Emission

spectra were recorded (λ_{ex} = 525 nm; λ_{em} = 540 - 750 nm) upon addition of increasing amounts of a 2 mM stock solution of the corresponding compound **L**, **1** or **2** (Figure 100). Each sample solution was allowed to equilibrate 10 min before the spectra was recorded.

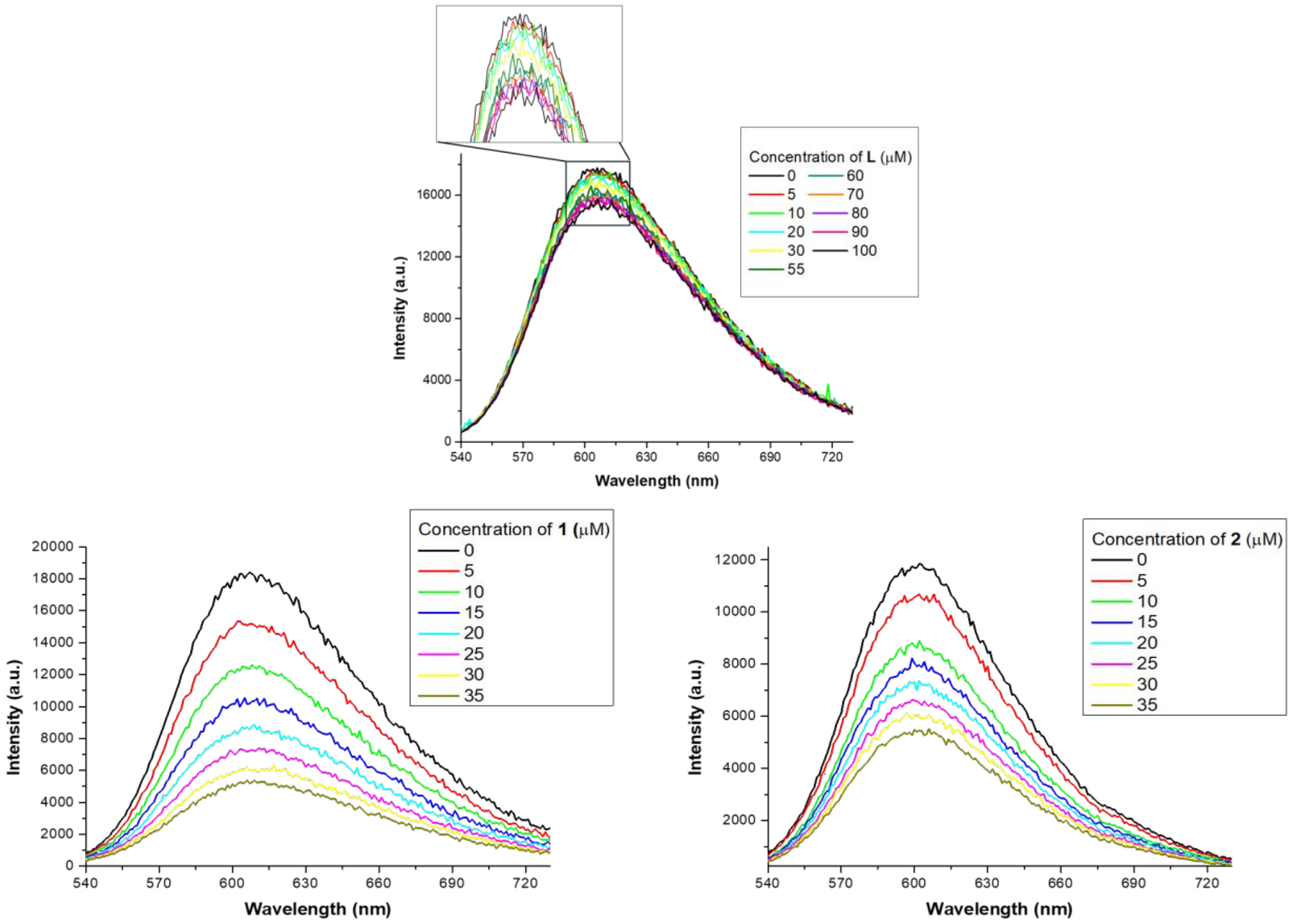

Figure S100. Emission spectra of EB bound to DNA upon addition of increasing concentrations of **L** (up), **1** (lower left) and **2** (lower right).

The k_{app} (apparent binding constant) for each complex was calculated using the following equation (eq. S12).[49manuscrip]

$$k_{app} = k_e(EtBr) \cdot \frac{[EtBr]\ (\mu M)}{C_{50}} \quad \textbf{(Eq. S12)}$$

Where k_e is the binding constant of EtBr ($k_e = 8.8 \cdot 10^6$ M(bp)$^{-1}$) and C_{50} is the concentration needed to induce a 50 % decrease in EtBr fluorescence intensity, determined by plotting the fluorescence intensity at 610 nm against the concentration of compound added (Figure S101).

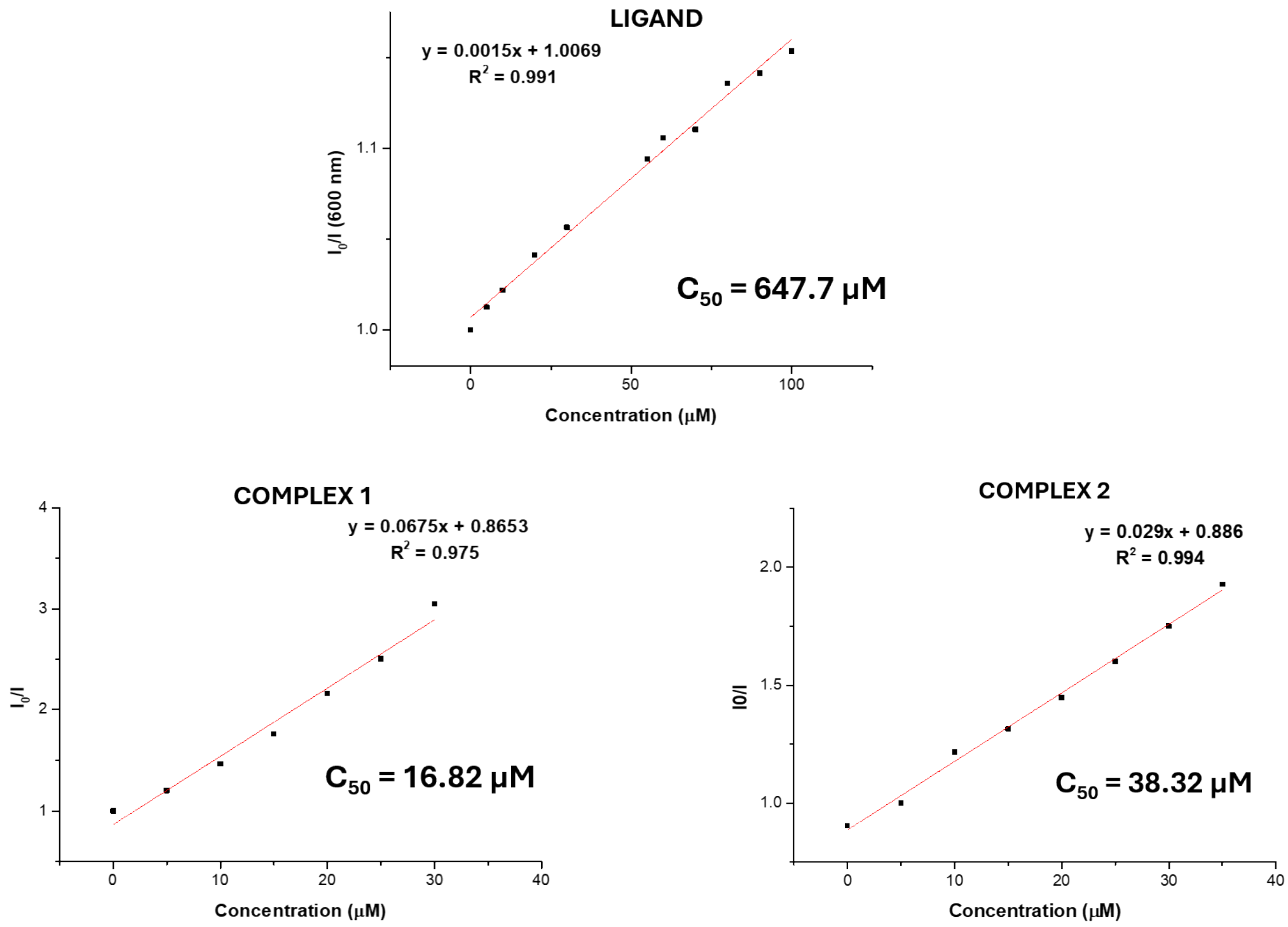

Figure S101. Plot of fluorescence intensity at 610 nm as a function of the added **L** (up), **1** (lower left) or **2** (lower left) concentration. The determined C_{50} value in each case is depicted in the corresponding graph.

7.2. Lipophilicity assay

Instrumentation: UV-Vis spectra were recorded with JASCO V-780 spectrophotometer.

Experimental procedure: complexes **1** and **2** (30 μM) were dissolved in 2 mL of PBS saturated in octanol, and to this solution 2 mL of octanol saturated in PBS were added. The mixture was left to stir for 24 h. Then each phase was measured by UV-Vis spectroscopy.

LogP was calculated using **Eq. S13**

$$logP = \frac{A_{octanol}}{A_{PBS}} \qquad \textbf{Eq. S13}$$

Obtained logP values for **1** and **2**: -2.04 and -1.83.

7.3. Cell culture

The human breast cancer MCF7, breast cancer triple negative MDA-MB-231, lung carcinoma A549 and NHDF-Ad fibroblasts cell lines were cultured in high glucose Dulbecco's modified Eagle's medium (DMEM) supplemented with 5% fetal bovine serum (FBS) (20% for fibroblasts) as well as 100 μg/mL penicillin/streptomycin at 37 °C under a humidified atmosphere of 95% air and 5% CO_2.

7.4. Cellular uptake

Cellular uptake was determined by measuring the ruthenium content inside the cells with inductively coupled plasma optical emission spectroscopy (ICP-OES). Measurements were recorded using a Thermo Scientific i CAP PRO XP Duo spectrometer.

Samples were prepared as follows: MCF7 cells were seeded in 75 cm^2 cell culture flasks (three flasks per condition) and incubated until approximately 90% confluence was reached. Before adding the compounds, the culture medium was removed and then 10 mL of culture medium containing 100 μM of **1** and **2** were added. For the control, 10 mL of compound-free culture medium were added. Cells were incubated with each complex (**1** or **2**) (100 μM) or without complex (control) for 24 h at 37 ˚C. Subsequently, cells were detached with 3 mL of trypsin and incubated for 10 min, after which 7 mL of culture medium were added. Cells were collected and counted, obtaining cell densities of approximately $1x10^6$ cells mL^{-1}. Samples were centrifuged, the supernatant was removed, and the pellets were washed once with PBS. The samples were then digested for 24 h with 1 mL of concentrated nitric acid. Once digested, 9 mL of ultrapure water were added, and ICP-OES measurements were performed in triplicate for each of the samples.

7.5. Cytotoxicity and photocytotoxicity assay

The MTT assay was used to determine cell viability as an indicator for cells sensitivity to the complexes. Exponentially growing cells MCF-7, MDA-MB-231, A549 and NHDF-Ad, were seeded at a density of approximately $7x10^3$ cells per well in 96-well flat-bottomed microplates and incubated for 24h. The complexes were dissolved in H_2O and then diluted with culture medium to obtain the final concentrations ranging from 200 μM to 0.4 μM in quadruplicate. Cells were incubated with compounds for 48 h at 37 ˚C. After 24 h of incubation with the complexes, duplicates of each concentration were irradiated with a LED lamp of 470 nm with a light dose of 15.1 J/cm^2 for 10 min, 525 nm with a light dose of 45.3 J/cm^2 for 30 min and 600nm with a light dose of 58.5 J/cm^2 for 60 min. The cells were cultured for a total of 48 h. 10 μL of MTT (5 mg/mL) were added to each well and plates were incubated for 2 h at 37 ˚C. Then, medium was discarded and DMSO (100 μL per well) was added to dissolve the formazan precipitates in the plates. A 96-well FLUOstar Omega (BMG LABTECH) was used to determine the optical density at 550 nm, and the IC_{50} was calculated. Each experiment was repeated in at least three independent analyses.

7.6. NAD^+/NADH assay

Intracellular NAD+ /NADH quantities were measured using a NAD+ /NADH assay kit (HY-K0313, MedChemExpress, NJ, USA) following the manufacturer´s instructions. NADH standards for calibration curve (0-50 μM) were prepared by serial dilution from a 1 mM stock solution provided in the kit (**Figure S102**).

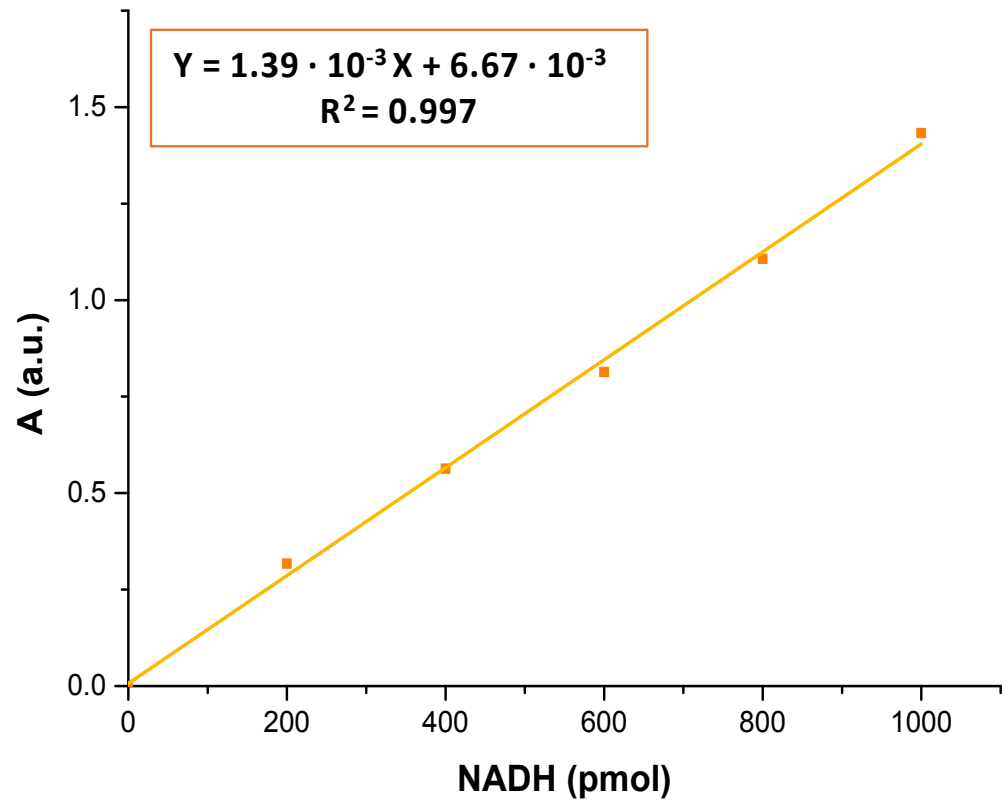

Figure S102. Calibration curve for NADH determination.

Samples were prepared as follows: 5 x 10^5 cells per well were seeded in a 6-well plate and incubated for 24 h. Then, the culture medium was replaced with fresh medium containing 70 μM of **1**. For the controls, it was replaced with compound-free medium. Cells were incubated for 24 h at 37 ˚C. After that, three wells were irradiated (two treated with complex **1** and one compound-free well, used as the irradiated control), and three wells were kept in dark (two treated with complex **1** and one compound-free well, used as the dark control). Irradiation was performed at 600 nm for 90 min (87.8 J/cm^2). Then, cells were detached with 500 μL of trypsin and incubated for 10 min, after which 1.5 mL of culture medium were added. Cells were counted and approximately 1x10^6 cells were collected per sample. Samples were centrifuged, the supernatant removed, and the pellets were washed once with PBS. Subsequently, cells were lysed in cold extraction buffer for 10 min and centrifuged (4 ˚C).
Supernatants were divided into two aliquots: one was used directly to determine total NADt (NADt = NADH + NAD^+), and the other was heated at 60 ˚C for 30 min to selectively degrade NAD^+, enabling the quantification of NADH. For both standards and samples, 20 μL were transferred to a 96-well plate, mixed with 90 μL of ADH enzyme working solution, incubated at 37 ˚C for 10 min, followed by the addition of 10 μL of chromogenic reagent (provided in the kit). After incubation at 37 ˚C for an additional 30 min, absorbance was measured at 450 nm using A 96-well FLUOstar Omega (BMG LABTECH) reader. Measurements were performed in triplicate for each condition independently.
NAD+ quantities and ratio were calculated using equations S14 and S15:

$$[\mathrm{NAD}^+] = [\mathrm{NAD}^+ + \mathrm{NADH}] - [\mathrm{NADH}] \qquad \textbf{(Eq. S14)}$$

$$\frac{[\mathrm{NAD}^+]}{[\mathrm{NADH}]} = \frac{[\mathrm{NAD}^+ + \mathrm{NADH}] - [\mathrm{NADH}]}{[\mathrm{NADH}]} \qquad \textbf{(Eq. S15)}$$

7.7. GSH/GSSG assay

Intracellular GSSG/GSH quantities were measured using a GSH/GSSG assay kit (HY-K0311, MedChemExpress, NJ, USA) following the manufacturer´s instructions. Samples were

prepared as follows: 5 x 10^5 cells per well were seeded in a 6-well plate and incubated for 24 h at 37 ˚C. The culture medium was then replaced with fresh medium containing 70 μM of **1**. For the controls, compound-free medium was used. Cells were incubated for 24 h at 37 ˚C. Three wells were irradiated (two treated with complex **1** and one compound-free well, used as the irradiated control), and three wells were kept in dark (two treated with complex **1** and one compound-free well, used as the dark control). Irradiation was performed at 600 nm for 90 min (87.8 J/cm^2). Then, cells were detached with 500 μL of trypsin and incubated for 10 min, after which 1.5 mL of culture medium were added. Cells were counted and approximately 1x10^6 cells were collected per sample. Samples were centrifuged, the supernatant removed and washed once with PBS.

Subsequently, cells were lysed by sonication in the extraction buffer provided by the kit for 10 min and centrifuged at 4 ˚C. The resulting supernatants were used as samples.

For each sample, two aliquots were prepared for the determination of total glutathione (GSH +GSSG) and oxidized glutathione (GSSG), respectively. For total glutathione, 20 μL of sample were mixed with 65 μL of buffer solution in each well of a 96-well plate. For GSSG, 20 μL of sample were mixed with 60 μL of buffer solution and 5 μL of the quenching solution, both supplied with the kit. Plates were then incubated at 37 ˚C for 10 min under shaking.

Standard curves were constructed in parallel using GSH or GSSG standard solutions provided by the kit, prepared by serial dilution (0-50 μM and 0-25 μM, respectively) and processed under the same conditions as the samples (**Figure S103**).

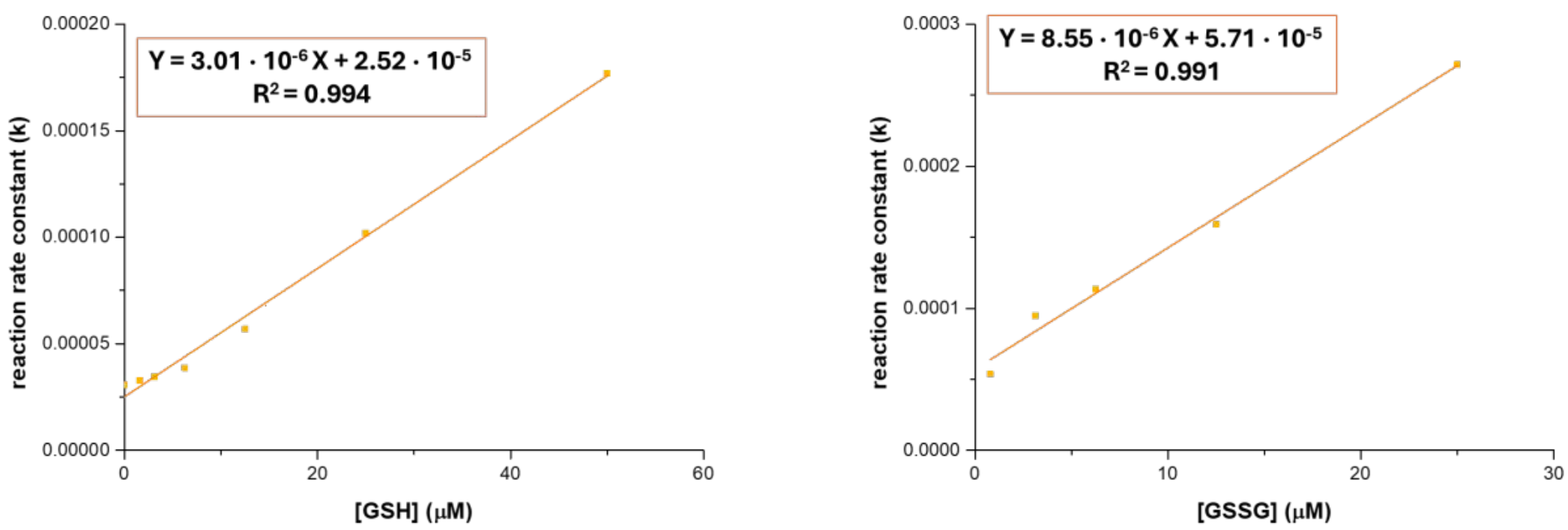

Figure S103. Calibration curves for GSH (left) and GSSG (right) determination.

After incubation, DTNB and enzyme solutions (glutathione reductase) provided by the kit were added rapidly using a multichannel pipette to ensure simultaneous reaction initiation. Absorbance was measured at 412 nm every 10 s for a total of 30 cycles using a 96-well FLUOstar Omega plate reader (BMG LABTECH).

Reaction rate constants (k) were obtained by linear fitting of absorbance versus time for each well, and glutathione concentrations were determined by interpolation from the corresponding standard curves. Reduced glutathione (GSH) quantities were calculated from total glutathione and GSSG quantities, using equation S9 measurements were performed in triplicate for each condition independently.

$$\mathrm{GSH} = (\mathrm{GSH} + \mathrm{GSSG}) - 2 \text{ x } \mathrm{GSH} \qquad \textbf{(Eq. S16)}$$

7.8. Flow cytometry studies: ROS and mitochondrial membrane potential

Experimental procedure: 200000 cells (MCF7/mL were seeded in flat-bottom 12-well plates (1 mL/well) in complete medium and allowed to attach for 24 h. A solution of the complexes **1** and **2** were added at concentration of 200 μM and MCF7 cells were cultured for a total of 48 h. A duplicate was irradiated with a 600 nm LED at a dose of 58.5 J/cm^2 for 60 min after 24 h of incubation and left to incubate for another 24 h. The cells were then de-adhered with 200 μL of trypsin and resuspended in 1 mL of media. This cell suspension has been used for all flow cytometry measurements that have been carried out at cytometer SA3800 Sony (cell cycle) and cytometer GALLIOS Beckman Coulter. Commercial kits were used for all flow cytometry studies and in all cases, they were used as indicated in the manufacturer's instructions. ROS production: CellROX® Green and CellROX® Orange Flow Cytometry Assay Kits (molecular probes by life technologies, catalogue number C10492). Mitochondrial potential: MitoStep™ Flow Cytometry Mitochondrial Membrane Potential Assay (immunostep, reference: MITO-100T).

8. REFERENCES

(1) Athmor, S.; Hernández-Castillo, D.; Maryasin, B.; Seeber, P.; Mengele, A. K.; Gräfe, S.; González, L.; Rau, S. Strong Ligand Stabilization Based on π-Extension in a Series of Ruthenium Terpyridine Water Oxidation Catalysts. *Chem. Eur. J.* **2021**, *27*, 16871–16878.

(2) Pitre, S. P.; McTiernan, C. D.; Vine, W.; DiPucchio, R.; Grenier, M.; Scaiano, J. C. Visible-Light Actinometry and Intermittent Illumination as Convenient Tools to Study $Ru(bpy)_3Cl_2$ Mediated Photoredox Transformations. *Sci. Rep.* **2015**, *5*, 16397.

(3) Saleh, F. S.; Rahman, M. R.; Okajima, T.; Mao, L.; Ohsaka, T. Determination of formal potential of NADH/NAD$^+$ redox couple and catalytic oxidation of NADH using poly (phenosafranin)-modified carbon electrodes. *Bioelectrochemistry* **2011**, *80*, 121–127.

(4) Chen, Y.; Chen, H.; Tian, H. Immobilization of a cobalt catalyst on fullerene in molecular devices for water reduction. *Chem. Commun.* **2015**, *51*, 11508–11511.

(5) Mateyise, N. G. S.; Conradie, M. M.; Conradie, J. Electronic effect of substituent groups on the oxidation and reduction of bis(2,2′:6′,2″-terpyridine)ruthenium. *J. Electroanal. Chem.* **2023**, *947*, 117807.

(6) Schafer, F.Q.; Buettner, G.R. Redox environment of the cells as viewed through the redox state of the glutathione disulfide/glutathione couple. *Free Radic. Biol. Med.* **2001**, 30, 1191–1212.

(7) Romero, N. A.; Nicewicz, D. A. Organic Photoredox Catalysis. *Chem. Rev.* **2016**, *116*, 10075–10166.

(8) Cardona, C. M.; Li, W.; Kaifer, A. E.; Stockdale, D.; Bazan, G. C. Electrochemical Considerations for Determining Absolute Frontier Orbital Energy Levels of Conjugated Polymers for Solar Cell Applications. *Adv. Mater.* **2011**, *23*, 2367−2371

(9) Yan, Y.; Yu, Z.; Liu, C.; Jin, X. Effects of phenyl/thienyl substituents at acetylacetone auxiliary ligands on the properties of cyclometalated platinum(II) complexes. *Dyes Pigm.* **2020**, *173*, 107949.

(10) Yan, Y.; Yu, Z.; Wang, J.; Liu, C. An oxygen-sensitive cyclometalated platinum(II) complex with a triphenylamine modified acetylacetone derivative. *Tetrahedron Lett.* **2023**, *119*, 154427.

(11) Wolfe, A.; Shimer, G. H.; Meehan, T. Polycyclic aromatic hydrocarbons physically intercalate into duplex regions of denatured DNA. *Biochemistry* **1987**, *26*, 6392–6396.

(12) Gil-Moles, M.; Olmos, M. E.; López-de-Luzuriaga, J. M.; Ott, I.; Gimeno, M. C. A dual approach to cancer treatment: gold (I) terpyridine derivatives as DNA binders and inhibitors of mammalian thioredoxin reductase. *Inorg. Chem. Front.* **2024**, *11*, 4802–4814.
